# AI-contextualized self-efficacy and exercise engagement among vocational college students in China: a cross-sectional serial mediation analysis of exercise self-efficacy and exercise outcome expectations

**DOI:** 10.3389/fpsyg.2026.1933716

**Published:** 2026-10-06

**Authors:** Guoliang Hou, Lintong Gan

**Affiliations:** 1 Department of Physical Education, Yunnan Medical Health College, Kunming, Yunnan, China; 2 Pathumthani University, Pathumthani, Thailand; 3 Myongji University, Yongin, Gyeonggi-do, Republic of Korea

**Keywords:** artificial intelligence-contextualized self-efficacy, cross-sectional study, exercise engagement, exercise outcome expectations, exercise self-efficacy, serial mediation, vocational college students

## Abstract

**Background:**

It remains unclear whether artificial intelligence-contextualized self-efficacy, reflecting general efficacy beliefs about coping with difficulties and achieving goals with artificial intelligence support, is associated with exercise engagement. This study examined this association and specific and serial statistical indirect effects involving exercise self-efficacy and exercise outcome expectations.

**Methods:**

A cross-sectional questionnaire survey was conducted among 2,279 students recruited by convenience sampling from one vocational college in China. Participants completed self-report measures of artificial intelligence-contextualized self-efficacy, exercise self-efficacy, exercise outcome expectations, and exercise engagement. Confirmatory factor analysis examined the measurement model. PROCESS Models 4 (preliminary) and 6 (primary) evaluated indirect effects using 5,000 bootstrap resamples and percentile 95% confidence intervals. Supplementary latent-variable structural equation models examined the theory-specified mediator ordering and its reversal. All regression and structural models adjusted for gender, age, and grade.

**Results:**

In the main PROCESS analyses, artificial intelligence-contextualized self-efficacy was positively associated with exercise engagement in the total-association model (*β* = 0.427, *p* < 0.001). After including exercise self-efficacy and exercise outcome expectations, the direct association remained significant (*β* = 0.246, *p* < 0.001). The specific indirect effects through exercise self-efficacy (*B* = 0.047, 95% confidence interval [0.0352, 0.0598]) and exercise outcome expectations (*B* = 0.048, 95% confidence interval [0.0378, 0.0586]) were significant. The serial indirect effect through exercise self-efficacy and exercise outcome expectations in the theory-specified order was also significant (*B* = 0.016, 95% confidence interval [0.0116, 0.0198]). Supplementary models yielded identical fit for the two mediator orderings.

**Conclusion:**

In this convenience sample, artificial intelligence-contextualized self-efficacy was associated with self-reported exercise engagement, with statistical indirect effects involving exercise self-efficacy and exercise outcome expectations. These minimally adjusted cross-sectional findings do not establish temporal ordering, causal mechanisms, or effects of actual artificial intelligence use.

## 1 Introduction

Insufficient physical activity remains an important health concern among college students in China. In a multi-region survey of 11,173 Chinese university students, only 4.8% reported engaging in more than 60 min of moderate-to-vigorous physical activity per day (Deng et al., 2024), while physical inactivity is associated with an increased risk of noncommunicable diseases and premature mortality (World Health Organization, 2024). Exercise engagement among college students is an important psychological construct for understanding the quality and intensity of students’ involvement in physical exercise and the development of healthy lifestyles. In the present study, exercise engagement is operationalized through vigor persistence, focused satisfaction, value cognition, and participation autonomy (Dong, 2017), and is distinguished from objectively measured physical activity and long-term exercise adherence. Previous systematic-review and randomized-trial evidence suggests that wearable activity trackers and smartphone applications can support physical activity, although their effects vary and technology provision alone does not ensure sustained behavioral change (Romeo et al., 2019; Ferguson et al., 2022; Kim et al., 2018). Artificial intelligence (AI)-powered mobile fitness applications have also been examined as goal-supportive technologies related to users’ goal perceptions and continuance usage intentions (Lee and Lin, 2023). However, evidence concerning digital tools does not establish an association between AI-related efficacy beliefs and exercise engagement. In the present study, AI-contextualized self-efficacy (AICSE) denotes general efficacy beliefs about coping with difficulties and achieving goals with AI support, assessed using the GSE-6AI (Morales-García et al., 2024). Exercise-related instructions framed the general items, which were not rewritten as exercise-specific items and do not directly assess AI-specific competence. Actual AI use and intervention exposure were not measured. Whether AICSE is associated with exercise engagement among vocational college students remains insufficiently examined.

Previous research has linked AI self-efficacy to students’ learning engagement, providing adjacent rather than direct evidence for the association examined here (He et al., 2025). Two exercise-related psychological variables were included in the proposed model. First, exercise self-efficacy (ESE) reflects students’ beliefs about their ability to initiate, complete, maintain, and regulate physical exercise; it represents a capability-oriented belief about whether they can perform exercise (Bandura, 1977; Jiang et al., 2018). Second, exercise outcome expectations (EOE) reflect students’ expectations regarding the positive outcomes of physical exercise, rather than the outcomes of using AI (Williams et al., 2005; Resnick, 2005; Bohlen et al., 2022). Previous research has linked ESE with EOE, supporting their joint consideration in the model (Marszalek et al., 2017). The ESE–EOE ordering was specified on theoretical grounds rather than established by the cross-sectional design; reverse or reciprocal associations remain possible. In a convenience sample from a single vocational college in China, the present study aimed to: (1) examine the hypothesized positive association between AICSE and exercise engagement; (2) test the specific statistical indirect effect through ESE; (3) test the specific statistical indirect effect through EOE; and (4) test the serial statistical indirect effect through ESE and EOE in the theory-specified order. The model represents a theory-specified configuration of concurrent associations and statistical indirect effects, not an established causal mechanism.

## 2 Literature review and research hypotheses

### 2.1 AI-contextualized self-efficacy and exercise engagement

In the present study, AI-contextualized self-efficacy (AICSE) refers to general efficacy beliefs about coping with difficulties and achieving goals with AI support, assessed using the GSE-6AI (Morales-García et al., 2024). It is not a direct measure of AI-specific competence, actual AI use, or implementation of AI recommendations. Exercise engagement reflects students’ active participation, effort investment, sustained persistence, and self-regulation in physical exercise and, in the present study, is operationalized through vigor persistence, focused satisfaction, value cognition, and participation autonomy (Dong, 2017). Self-Efficacy Theory emphasizes the relevance of capability beliefs to behavioral choices, effort investment, and persistence (Bandura, 1977). Social Cognitive Theory further situates these beliefs within reciprocal relationships among personal factors, behavior, and the environment (Bandura, 1989). Research among college students has associated AI self-efficacy with AI literacy, AI use, attitudes, and interest (Bewersdorff et al., 2025). Related research among K-12 teachers has linked AI self-efficacy with attitudes, perceived usefulness, prior use, perceived relevance, and readiness to use AI (Bergdahl and Sjöberg, 2025). AI self-efficacy has also been associated with students’ learning engagement (He et al., 2025). These studies provide adjacent evidence from educational settings, but do not establish an AICSE–exercise engagement association. The theoretical relevance of efficacy beliefs to effort and persistence nevertheless motivates testing whether AICSE is positively associated with exercise engagement among vocational college students. The proposed relationship concerns a concurrent association rather than an effect of actual AI use. Therefore, the following hypothesis is proposed:

*H1*: AI-contextualized self-efficacy is positively associated with exercise engagement.

### 2.2 Mediating role of exercise self-efficacy

Exercise self-efficacy (ESE) refers to individuals’ beliefs about their ability to initiate, complete, maintain, and regulate physical exercise. It is particularly reflected in confidence in maintaining exercise when facing insufficient time, physical fatigue, lack of motivation, or environmental constraints. According to Self-Efficacy Theory, individuals’ judgments about their own capabilities are theorized to shape behavioral choices, effort investment, persistence, and coping responses when facing difficulties (Bandura, 1977). In the present study, AICSE concerns general efficacy beliefs contextualized to AI support, whereas ESE concerns capability beliefs specific to physical exercise. Both are efficacy beliefs, but they concern distinct contexts and should not be treated as interchangeable. Research in higher education has associated institutional AI capability with students’ learning-related self-efficacy (Wang et al., 2023). This provides adjacent evidence from a different context, rather than direct evidence for the proposed AICSE–ESE association. The shared focus on coping with difficulties motivates examining whether AICSE and ESE covary, without assuming that confidence with AI automatically transfers to exercise.

In addition, ESE may be closely related to exercise engagement. Students with higher ESE are generally more likely to perceive physical exercise as an activity that can be completed, regulated, and maintained, and they may show higher levels of active participation, effort investment, sustained persistence, and self-regulation during exercise (Jackson and Dimmock, 2012; Lewis et al., 2016; Wang et al., 2022). At the same time, previous studies have further suggested that self-efficacy is closely related to physical activity behavior, exercise motivation, and confidence in overcoming physical activity barriers (Lewis et al., 2016; Wang et al., 2022; Rauff and Kumazawa, 2024). Taken together, these considerations motivate testing a statistical indirect association between AICSE and exercise engagement involving ESE, rather than assuming a temporal or causal sequence. Therefore, the following hypothesis is proposed:

*H2*: Exercise self-efficacy shows a statistical mediating role in the association between AI-contextualized self-efficacy and exercise engagement.

### 2.3 Mediating role of exercise outcome expectations

Exercise outcome expectations (EOE) refer to individuals’ subjective expectations regarding the possible outcomes of physical exercise. In the existing literature, EOE may involve physical health benefits, fitness improvement, emotional regulation, social benefits, exercise-related achievement, and improvements in self-evaluation. Previous physical activity research has regarded outcome expectancy as an important cognitive variable for understanding whether individuals are willing to participate in and maintain physical activity (Williams et al., 2005). Scale-related studies have further shown that exercise outcome expectations can be operationalized as expectations regarding the benefits and, in revised instruments, possible negative outcomes of exercise. Related measures developed mainly in older-adult contexts have differentiated outcome expectations into physical, social, and self-evaluative dimensions (Resnick, 2005; Wójcicki et al., 2009), while review evidence has identified EOE as a relevant but context-sensitive construct in physical activity research (Bohlen et al., 2022). Although the OEE was originally developed mainly in older-adult contexts, its reliability and factorial validity were re-examined in the present student sample. Computer self-efficacy has been theoretically linked to expected outcomes of technology use (Compeau and Higgins, 1995). This provides only a cross-contextual analogy: AICSE concerns efficacy with AI support, whereas EOE concerns anticipated outcomes of exercise. The present model tests whether these distinct beliefs are associated, without attributing exercise expectations to AI use or feedback.

In addition, EOE may be associated with exercise engagement. Review and meta-analytic evidence has linked positive outcome expectations with physical activity (Williams et al., 2005; Bohlen et al., 2022), providing adjacent evidence because physical activity and exercise engagement are not equivalent. Within Social Cognitive Theory, efficacy beliefs and outcome expectations are considered cognitive factors relevant to physical activity (Young et al., 2014; Beauchamp et al., 2019). This perspective motivates the expectation that students anticipating positive exercise outcomes may report greater effort investment and exercise engagement. Taken together, these considerations motivate testing a statistical indirect association between AICSE and exercise engagement involving EOE, rather than assuming a temporal or causal process. Therefore, the following hypothesis is proposed:

*H3*: Exercise outcome expectations show a statistical mediating role in the association between AI-contextualized self-efficacy and exercise engagement.

### 2.4 Serial mediation effect of exercise self-efficacy and exercise outcome expectations

Exercise self-efficacy (ESE) and exercise outcome expectations (EOE) are specified as hypothesized mediators in a serial statistical model of the association between AICSE and exercise engagement. According to Social Cognitive Theory, individual behavior is not determined by a single psychological variable but is shaped by the reciprocal interaction among personal cognition, behavioral experience, and environmental resources (Bandura, 1989, 1998). In the present model, AICSE, ESE, and EOE represent personal cognitive factors, whereas no social or environmental factor is directly modeled. Relevant but unmeasured influences may include teacher guidance, peer support and modeling, social norms, access to digital resources, and campus exercise conditions (Anderson et al., 2006; Young et al., 2014; Beauchamp et al., 2019). ESE concerns perceived capability to perform exercise, whereas EOE concerns anticipated exercise outcomes; the two constructs are conceptually distinct. Previous research based on Social Cognitive Theory has identified efficacy beliefs and outcome expectations as theoretically important cognitive variables for understanding physical activity (Young et al., 2014; Beauchamp et al., 2019). However, the strength and consistency of these constructs may vary across studies, indicating that outcome expectations should be interpreted as a theoretically relevant but context-sensitive construct. Previous research suggests that ESE is associated with more positive expectations of exercise benefits (Marszalek et al., 2017). Theoretically, perceived exercise capability may inform judgments about the attainability of expected outcomes.

Taken together, Self-Efficacy Theory and Social Cognitive Theory provide a rationale for testing a serial statistical association between AICSE and exercise engagement through ESE and EOE. The specified ordering does not establish temporal precedence or causality; reverse or reciprocal associations remain possible. Therefore, the following hypothesis is proposed:

*H4*: Exercise self-efficacy and exercise outcome expectations show a serial statistical mediating role in the association between AI-contextualized self-efficacy and exercise engagement.

In summary, existing theories and related empirical research provide a rationale for testing the association between AICSE and exercise engagement and the specific and serial statistical indirect pathways involving ESE and EOE. Figure 1 presents the conceptual model. The arrows indicate directions specified for statistical testing among concurrently measured variables. Specifically, this study aimed to: (1) examine the positive cross-sectional association between AICSE and exercise engagement; (2) test the statistical indirect pathway through ESE; (3) test the statistical indirect pathway through EOE; and (4) test the theory-specified serial statistical indirect pathway through ESE and EOE.

**Figure 1:**
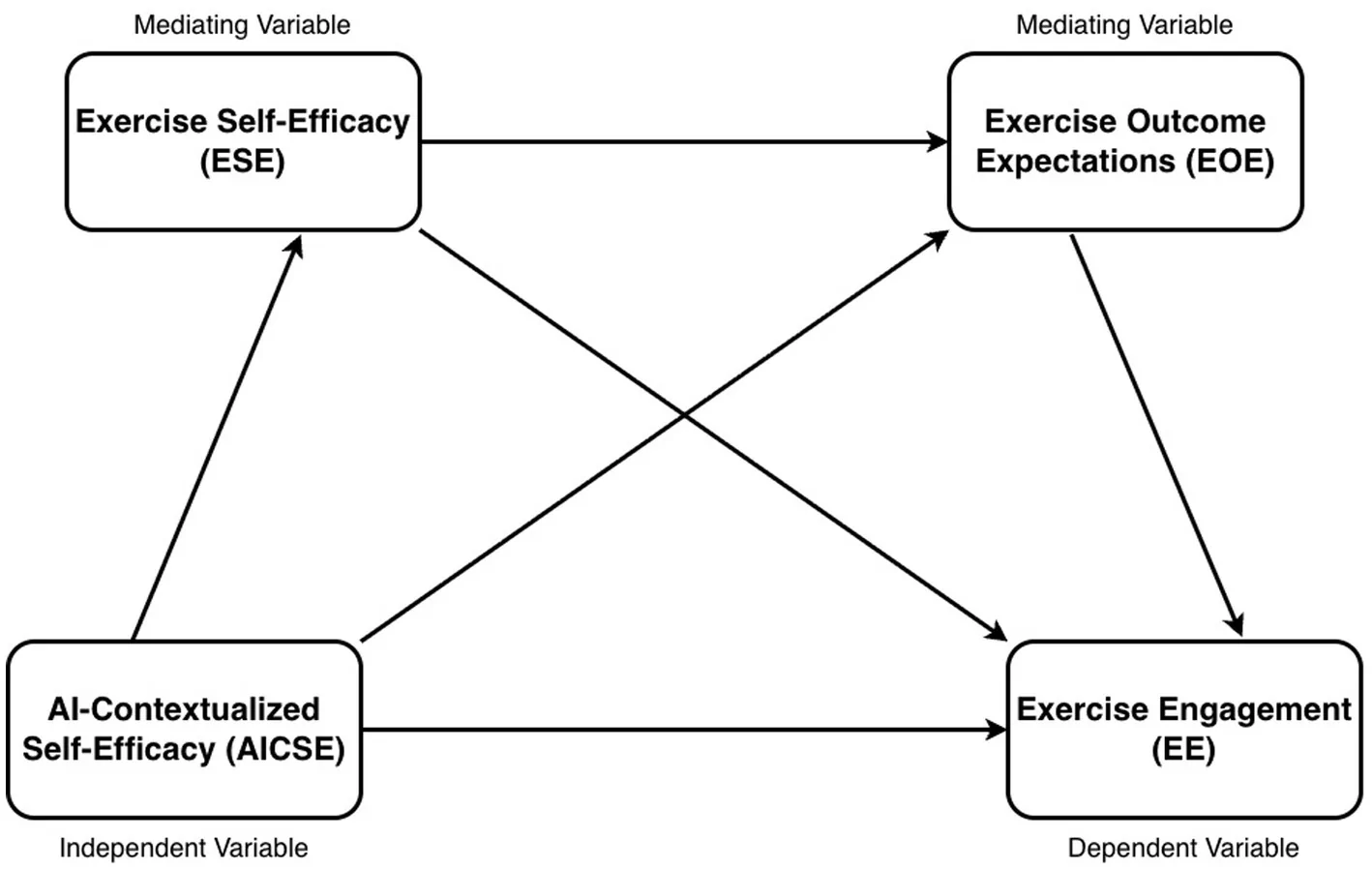
Arrows indicate the theory-specified directions used for statistical testing among concurrently measured variables and should not be interpreted as evidence of temporal precedence or causal effects. AICSE = AI-contextualized self-efficacy; ESE = exercise self-efficacy; EOE = exercise outcome expectations; EE = exercise engagement.

## 3 Materials and methods

### 3.1 Participants and procedure

This study used a cross-sectional questionnaire survey design. The questionnaire survey, data screening, and initial statistical analyses were conducted during June–July 2026. Students were recruited from Yunnan Medical Health College, a three-year vocational college in China, using convenience sampling. Recruitment was facilitated by teachers, counselors, and class advisors through class group chats and in-person invitations during breaks between classes. Specific academic-major information was not collected in the present survey. Accordingly, participants could not be classified at the individual level by academic major, and academic major was not included in the statistical analyses. The questionnaire was administered in both paper-based and online formats, with the administration mode determined by class-level or on-site arrangements. Paper-based questionnaires were distributed in person, whereas the online version was administered through the Wenjuanxing online survey platform. Both formats used identical questionnaire items, instructions, and response options. Students were eligible to participate if they: (1) were enrolled at Yunnan Medical Health College during the study period; (2) were aged 18 years or older; (3) were able to understand the questionnaire content and complete it independently; and (4) provided informed consent and voluntarily agreed to participate in the study. During data screening, questionnaires containing missing responses (*n* = 82) or repeated selection of the same response option across a large number of items (*n* = 15) were excluded from analysis. Online questionnaires with completion times exceeding 5 min were also excluded (*n* = 24). A total of 2,400 questionnaires were received, comprising 1,873 online and 527 paper-based questionnaires. During screening, 121 questionnaires were excluded (27 online and 94 paper-based), leaving 2,279 questionnaires for analysis (1,846 online and 433 paper-based), representing 94.96% of those received. The final analytic dataset contained no missing values on the 45 measurement items or on gender, age, and grade. In the final sample, 934 students reported being male, representing 41.0% of the sample, and 1,345 reported being female, representing 59.0%. Regarding age, 326 students were 18 years old (14.3%), 977 were 19 years old (42.9%), 651 were 20 years old (28.6%), and 325 were 21 years or older (14.3%). Because the participants were recruited from a three-year vocational college, the sample included students from three grade levels: 760 first-year students (33.3%), 570 second-year students (25.0%), and 949 third-year students (41.6%). This study was approved by the Ethics Review Committee of Yunnan Medical Health College (approval number: YNYYJK-LLSL-001). Before completing the questionnaire, all participants were informed of the study purpose, anonymity, confidentiality principles, voluntary participation, and their right to withdraw at any time. Online participants could access the questionnaire items only after providing electronic informed consent; those who declined consent could not proceed. Paper-based participants provided written informed consent before completing the questionnaire. The study did not collect directly identifiable personal information, such as names, national identification numbers, student identification numbers, or mobile phone numbers. All questionnaire data were used only for research analyses, and demographic information and questionnaire responses were coded before being entered into the statistical analysis process.

### 3.2 Measures

#### 3.2.1 AI-contextualized self-efficacy

AI-contextualized self-efficacy (AICSE) was measured using the General Self-Efficacy Scale for Use with Artificial Intelligence (GSE-6AI), which assesses students’ efficacy beliefs regarding their ability to cope with difficulties, handle unexpected situations, and achieve goals with the support of artificial intelligence in AI-use contexts (Schwarzer and Jerusalem, 1995; Romppel et al., 2013; Morales-García et al., 2024). The GSE-6AI is an AI-contextualized version adapted from the General Self-Efficacy Scale-6 (GSE-6). In the present study, the English items were translated into Chinese and subsequently underwent tool-assisted linguistic checking. A contextualized instruction framed responses in relation to AI use associated with physical exercise, while the general AI-related item content was retained rather than rewritten as exercise-specific items. Accordingly, AICSE was interpreted as general efficacy beliefs contextualized to AI support, rather than a direct measure of AI-specific competence, actual AI use, or exercise-specific AI skills. The scale consists of six items with a unidimensional structure. All items were positively worded and scored in the same direction, and no items were reverse-scored. A sample item is “No matter what comes up, I can usually handle it with the support of artificial intelligence.” To maintain consistency in the response format across the questionnaire, the original 4-point response format was adjusted to a 5-point Likert-type scale, ranging from 1 = “does not describe me at all” to 5 = “describes me completely.” Because changes in response options may affect score distributions, psychometric performance, and comparability with the original scale, measurement equivalence with the original 4-point format was not assumed. Equivalence with the source-language version was also not established. Accordingly, the AICSE scores were used to characterize relative levels within the present sample and were not directly compared with scores from the original 4-point version. The internal consistency and factorial validity of the administered Chinese 5-point version were examined in the present sample. For subsequent analyses, a mean composite score across all items was calculated, with higher scores indicating greater AI-contextualized self-efficacy. In the present sample, Cronbach’s α for this scale was 0.882. The scale-specific CFA results indicated good model fit: *χ*^2^/df = 2.755, RMSEA = 0.028, CFI = 0.997, and TLI = 0.996. In the complete measurement model, standardized loadings for the six items ranged from 0.721 to 0.760, with composite reliability (CR) = 0.883 and average variance extracted (AVE) = 0.556 (Supplementary Table S1).

#### 3.2.2 Exercise self-efficacy

Exercise self-efficacy was measured using the Physical Activity Self-Efficacy Scale for College Students (PASS), which assesses students’ efficacy beliefs regarding their ability to persist in and complete physical exercise when facing different exercise barriers (Jiang et al., 2018). The present study used the original Chinese version of the scale. The scale consists of 10 items and includes two dimensions: situational motivation and subjective support. Situational motivation includes items 1–6, and subjective support includes items 7–10. All items were positively worded and scored in the same direction, and no items were reverse-scored. A sample item is “Even if no one accompanies me, I will still stick to my exercise plan.” The scale was rated on a 5-point Likert-type scale, ranging from 1 = “strongly disagree” to 5 = “strongly agree.” For subsequent analyses, a mean composite score across all items was calculated, with higher scores indicating greater exercise self-efficacy. In the present sample, Cronbach’s α values for situational motivation and subjective support were 0.870 and 0.825, respectively. The scale-specific CFA results indicated good model fit: *χ*^2^/df = 1.234, RMSEA = 0.010, CFI = 0.999, and TLI = 0.999. In the complete measurement model, ESE was specified as a higher-order factor reflected by situational motivation and subjective support. Standardized item and higher-order factor loadings, together with CR and AVE at the corresponding measurement levels, are reported in Supplementary Table S1.

#### 3.2.3 Exercise outcome expectations

Exercise outcome expectations were measured using the Outcome Expectations for Exercise Scale (OEE), which was originally developed to assess older adults’ expectations regarding the benefits of exercise (Resnick et al., 2000). In the present study, the English items of the OEE were translated into Chinese and administered to the student sample to assess students’ subjective expectations regarding the positive physical, psychological, and functional outcomes of exercise. The scale consists of nine items with a unidimensional structure. All items were positively worded and scored in the same direction, and no items were reverse-scored. A sample item is “Exercise makes my mood better in general.” The scale was rated on a 5-point Likert-type response format, and responses were coded from 1 = “strongly disagree” to 5 = “strongly agree.” For subsequent analyses, a mean composite score across all items was calculated, with higher scores indicating more positive exercise outcome expectations. The scale concerns anticipated outcomes of engaging in physical exercise rather than anticipated benefits of using AI. In the present sample, Cronbach’s α for this scale was 0.910. The scale-specific CFA results indicated good model fit: *χ*^2^/df = 3.006, RMSEA = 0.030, CFI = 0.995, and TLI = 0.993. In the complete measurement model, standardized item loadings ranged from 0.716 to 0.745, with CR = 0.910 and AVE = 0.528 (Supplementary Table S1). Targeted discriminant-validity evidence concerning EOE and the value cognition dimension of exercise engagement is presented in Supplementary Table S4.

#### 3.2.4 Exercise engagement

Exercise engagement was measured using the Physical Exercise Involvement Scale for College Students (PEIS), which assesses students’ vigor persistence, focused satisfaction, value cognition, and participation autonomy in physical exercise (Dong, 2017). The present study used the original Chinese version of the scale. The scale consists of 20 items and includes four dimensions: vigor persistence, focused satisfaction, value cognition, and participation autonomy. Vigor persistence includes items 1–6, focused satisfaction includes items 7–12, value cognition includes items 13–17, and participation autonomy includes items 18–20. All items were positively worded and scored in the same direction, and no items were reverse-scored. A sample item is “Physical exercise is a meaningful activity for me.” The scale was rated on a 5-point Likert-type scale, ranging from 1 = “does not describe me at all” to 5 = “describes me completely.” For subsequent analyses, a mean composite score across all items was calculated, with higher scores indicating greater exercise engagement. The score was interpreted as self-reported exercise engagement, not objectively measured physical activity or longitudinally assessed exercise adherence. In the present sample, Cronbach’s α values for vigor persistence, focused satisfaction, value cognition, and participation autonomy were 0.873, 0.874, 0.859, and 0.769, respectively. The scale-specific CFA results indicated good model fit: *χ*^2^/df = 1.064, RMSEA = 0.005, CFI = 0.999, and TLI = 0.999. In the complete measurement model, EE was specified as a higher-order factor reflected by its four dimensions. Standardized item and higher-order factor loadings, together with CR and AVE at the corresponding measurement levels, are reported in Supplementary Table S1.

### 3.3 Statistical analysis

Data analyses used IBM SPSS Statistics 26.0, AMOS, and PROCESS 4.1. Descriptive statistics and Cronbach’s α were calculated. Harman’s test examined the first unrotated principal component across all 45 items. CFA assessed the scale-specific and complete measurement models. Factor loadings, CR, and AVE were examined, and discriminant validity was assessed using latent-factor correlations and the Fornell–Larcker criterion (Supplementary Tables S1, S2). Maximum-likelihood model comparisons included the complete versus single-factor 45-item models and the two-factor versus single-factor 14-item EOE–value cognition models (Supplementary Tables S3, S4). Fit was evaluated using *χ*^2^/df, CFI, TLI, and RMSEA, with AIC for model comparisons. Harman’s test and the 45-item single-factor comparison were preliminary diagnostics that could not rule out common method variance. Independent-samples t tests assessed gender differences, one-way analyses of variance assessed age and grade differences, and Pearson correlations assessed unadjusted bivariate associations. Using mean composite scores, PROCESS Model 4 provided preliminary single-mediator analyses, whereas Model 6 was the primary model estimating specific and serial indirect effects between AICSE and EE through ESE and EOE in the theory-specified order. Regression and SEM analyses adjusted for gender, age, and grade; age and grade were entered as single numerically coded covariates (Supplementary Table S5). PROCESS indirect effects were evaluated using 5,000 bootstrap resamples and percentile 95% confidence intervals, with intervals excluding zero indicating significance; direct and total associations used conventional regression intervals. PROCESS results were reported as standardized path coefficients (β) and unstandardized association estimates (B). Supplementary latent-variable SEMs retained the measurement structure and examined both ESE → EOE and EOE → ESE orderings using maximum likelihood (Supplementary Tables S5, S6; Supplementary Figure S1). Standardized SEM indirect, direct, and total estimates were reported with percentile 95% confidence intervals from 5,000 bootstrap samples; serial-specific inference was based on PROCESS Model 6. Significance was set at *p* < 0.05, with two-tailed correlation and coefficient tests. Results were interpreted as minimally adjusted cross-sectional associations, not established temporal or causal mechanisms.

## 4 Results

### 4.1 Descriptive statistics and group differences in AI-contextualized self-efficacy, exercise self-efficacy, exercise outcome expectations, and exercise engagement

Table 1 presents the descriptive statistics and group differences for the study variables. The overall mean scores for AICSE, ESE, EOE, and EE ranged from 3.24 to 3.50, all above the theoretical midpoint of the 5-point scales. Male students reported significantly higher mean scores than female students on all four variables. Significant differences were observed across age and grade groups for all four variables, although the age-group result for ESE was close to the conventional significance threshold.

**Table 1:** Descriptive statistics (M ± SD) and group difference tests for the study variables.

Group	*n*	AI-contextualized self-efficacy	Exercise self-efficacy	Exercise outcome expectations	Exercise engagement
Male	934	3.29 ± 0.81	3.55 ± 0.54	3.39 ± 0.55	3.48 ± 0.48
Female	1,345	3.22 ± 0.83	3.47 ± 0.53	3.32 ± 0.56	3.40 ± 0.51
Overall	2,279	3.24 ± 0.82	3.50 ± 0.53	3.35 ± 0.55	3.43 ± 0.50
Gender differences (*t*)		1.980*	3.549***	2.703**	4.175***
Age differences, *F*(3, 2,275)		6.699***	2.611*	6.000***	5.791***
Grade differences, *F*(2, 2,276)		7.791***	3.127*	4.704**	7.661***

Values are presented as M ± SD. Gender differences were examined using independent-samples t tests, and age and grade differences were examined using one-way analyses of variance. Positive t values indicate higher mean scores in male students. Gender was coded as 1 = male and 2 = female. Age was coded as 1 = 18 years, 2 = 19 years, 3 = 20 years, and 4 = 21 years or older. Grade was coded as 1 = first-year student, 2 = second-year student, and 3 = third-year student. AI = artificial intelligence. *p < 0.05, **p < 0.01, ***p < 0.001.

### 4.2 Common method bias test

Harman’s single-factor test was based on the unrotated principal component results for all 45 items. The results showed that eight components had eigenvalues greater than 1, and the first component explained 29.30% of the total variance, which was below the 40% empirical threshold adopted in prior questionnaire-based studies (Dong et al., 2026). The 45-item single-factor CFA model showed poor fit (*χ*^2^/df = 24.543, RMSEA = 0.102, CFI = 0.546, TLI = 0.524; Supplementary Table S3). However, these preliminary diagnostics cannot rule out common method variance or its influence on the observed associations.

### 4.3 Correlation analysis of AI-contextualized self-efficacy, exercise self-efficacy, exercise outcome expectations, and exercise engagement

Table 2 presents the unadjusted Pearson correlations among the mean composite scores of the four study variables. AICSE was positively correlated with ESE (*r* = 0.470, *p* < 0.001), EOE (*r* = 0.495, *p* < 0.001), and EE (*r* = 0.435, *p* < 0.001). ESE was also positively correlated with EOE (*r* = 0.435, *p* < 0.001) and EE (*r* = 0.381, *p* < 0.001). EOE was likewise positively correlated with EE (*r* = 0.413, *p* < 0.001).

**Table 2:** Means, standard deviations, and correlations among study variables.

Variable	*M*	*SD*	1	2	3	4
1. AI-Contextualized self-efficacy	3.24	0.82	1			
2. Exercise self-efficacy	3.50	0.53	0.470***	1		
3. Exercise outcome expectations	3.35	0.55	0.495***	0.435***	1	
4. Exercise engagement	3.43	0.50	0.435***	0.381***	0.413***	1

*N* = 2,279. M = mean; SD = standard deviation; AI = artificial intelligence. Pearson correlations between mean composite scores are reported. The correlations were not adjusted for gender, age, or grade. All significance tests were two-tailed. ***p < 0.001.

### 4.4 Testing the statistical indirect effects of exercise self-efficacy and exercise outcome expectations in the association between AI-contextualized self-efficacy and exercise engagement

The main analyses used PROCESS Model 6 with gender, age, and grade as covariates; preliminary Model 4 indirect effects through ESE and EOE were also significant. The complete 45-item measurement model examined in the supplementary CFA showed good fit (*χ*^2^/df = 1.110, RMSEA = 0.007, CFI = 0.998, TLI = 0.998; Supplementary Table S3). The Fornell–Larcker criterion supported discriminant validity among the four core constructs (Supplementary Table S2). In the EOE–value cognition comparison, the two-factor model fitted better than the single-factor model (CFI = 0.997 versus 0.743), with a latent correlation of 0.389 (Supplementary Table S4). Table 3 and Figure 2 present standardized path coefficients (*β*), and Table 4 reports unstandardized estimates (B) and confidence intervals. The total AICSE–EE association was significant (*β* = 0.427, *t* = 22.624, *p* < 0.001), as was the direct association after including ESE and EOE (*β* = 0.246, *t* = 11.238, *p* < 0.001). AICSE was positively associated with ESE (*β* = 0.466, *t* = 25.110). AICSE and ESE were positively associated with EOE (*β* = 0.369, *t* = 18.512, and *β* = 0.256, *t* = 12.862, respectively), while ESE and EOE were positively associated with EE (*β* = 0.166, *t* = 7.859, and *β* = 0.212, *t* = 9.895, respectively; all *p* < 0.001). All regression equations were significant, with *R*^2^ = 0.225, 0.299, and 0.266 for ESE, EOE, and EE, respectively, and *R*^2^ = 0.197 for the total-association model. The specific indirect estimates were significant via ESE [*B* = 0.047, 95% CI (0.0352, 0.0598)], via EOE [*B* = 0.048, 95% CI (0.0378, 0.0586)], and serially via ESE and EOE [*B* = 0.016, 95% CI (0.0116, 0.0198)], representing 18.11, 18.34, and 5.95% of the total association, respectively. The total indirect estimate was *B* = 0.111 [95% CI (0.0952, 0.1263)], representing 42.39% of the total association. The direct and total associations were *B* = 0.150 [95% CI (0.1239, 0.1763)] and *B* = 0.261 [95% CI (0.2381, 0.2833)], respectively. The positive AICSE–EE association was consistent with H1, and the three specific indirect estimates were consistent with H2–H4, respectively. Supplementary covariate-adjusted SEMs with ESE → EOE and EOE → ESE orderings showed identical fit (*χ*^2^/df = 1.130, RMSEA = 0.008, CFI = 0.997, TLI = 0.997; Supplementary Table S5; Supplementary Figure S1). In both models, the standardized direct and total indirect estimates were 0.249 [95% CI (0.1884, 0.3091)] and 0.250 (95% CI [0.2082, 0.2922]), respectively (Supplementary Table S6). These minimally adjusted cross-sectional results do not establish temporal order or causal mechanisms, and model fit did not favor either ordering.

**Table 3:** Regression results for the theory-specified cross-sectional model.

Predictor	Exercise self-efficacy		Exercise outcome expectations		Exercise engagementᵃ		Exercise engagementᵇ	
	*β*	*t*	*β*	*t*	*β*	*t*	*β*	*t*
AI-contextualized self-efficacy	0.466	25.110***	0.369	18.512***	0.246	11.238***	0.427	22.624***
Exercise self-efficacy	—	—	0.256	12.862***	0.166	7.859***	—	—
Exercise outcome expectations	—	—	—	—	0.212	9.895***	—	—
*R* ^2^	0.225		0.299		0.266		0.197	
*F*	164.805***		194.060***		137.357***		139.311***	
df₁, df₂	4, 2,274		5, 2,273		6, 2,272		4, 2,274	

*N* = 2,279. Standardized regression coefficients (*β*) based on mean composite scores are reported. The first three equations correspond to PROCESS Model 6; the final equation estimates the total association. ᵃ The exercise engagement equation includes exercise self-efficacy and exercise outcome expectations. ᵇ The exercise engagement equation excludes these two variables. Gender, age, and grade were included as covariates in all equations but are not shown in the table. *R*^2^ and F refer to the full equations, including the covariates. df₁ and df₂ are the numerator and denominator degrees of freedom for the F tests. Dashes indicate predictors not included in the corresponding regression equation. The coefficients represent minimally adjusted cross-sectional associations and do not establish temporal order or causality. AI = artificial intelligence. All coefficient tests were two-tailed. ***p < 0.001.

**Figure 2:**
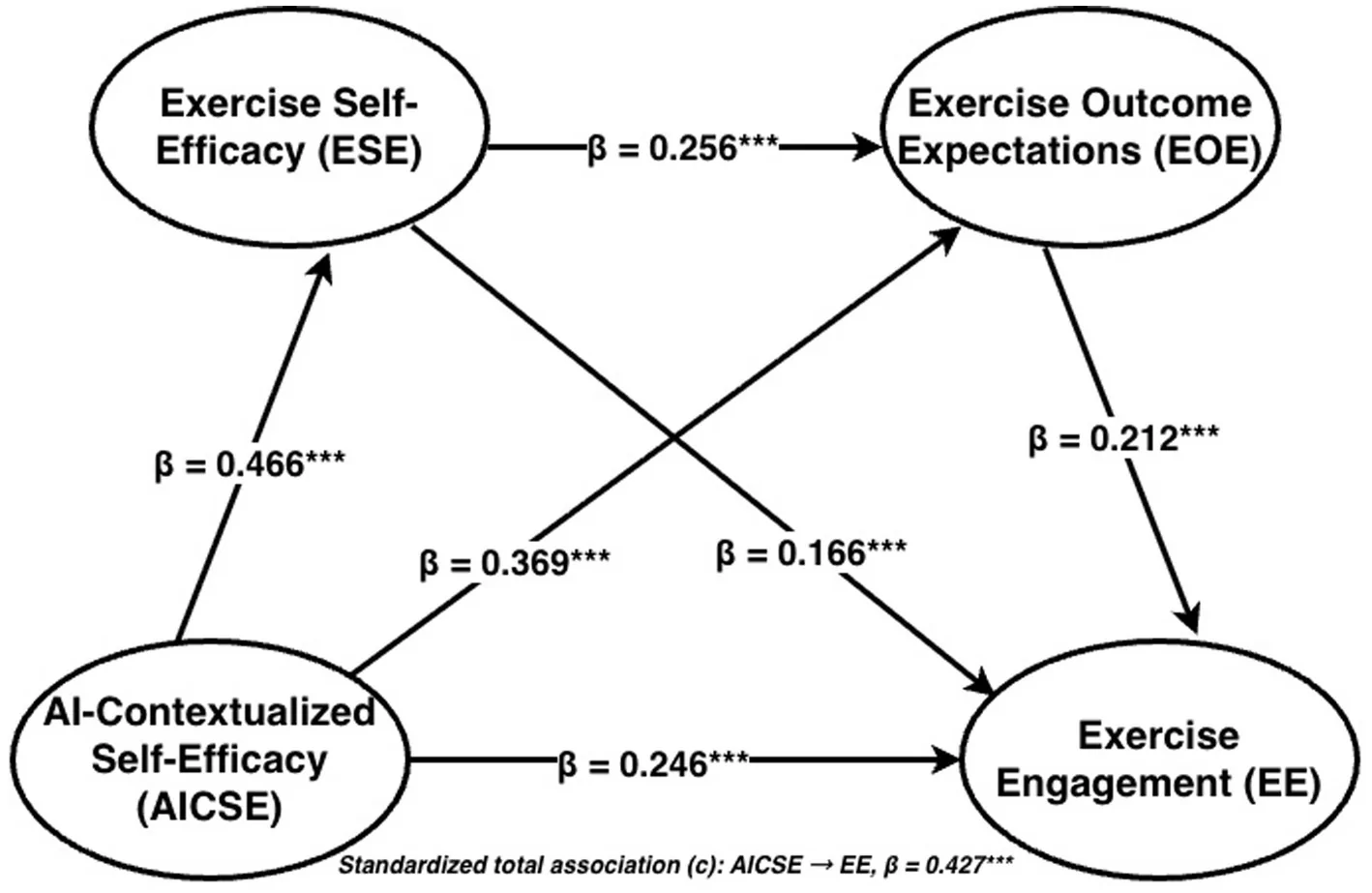
*N* = 2,279. Values shown on the paths are standardized regression coefficients (*β*) estimated using PROCESS Model 6. Gender, age, and grade were included as covariates in all equations but are omitted from the figure for clarity. The coefficient shown on the AICSE → EE path represents the standardized direct association (c’) after ESE and EOE were included in the model, whereas the coefficient shown below the model represents the standardized total association (c) estimated without the mediators. Arrows denote theory-specified directions for statistical estimation and do not indicate established temporal or causal effects. AICSE = Al-contextualized self-efficacy; ESE = exercise self-efficacy; EOE = exercise outcome expectations; EE = exercise engagement. ***p < 0.001.

**Table 4:** Mediation effects and proportions of the total effect.

Path	Unstandardized estimate (*B*)	Proportion of total association	95% CI	
			LL	UL
AICSE → ESE → EE	0.047	18.11%	0.0352	0.0598
AICSE → EOE → EE	0.048	18.34%	0.0378	0.0586
AICSE → ESE → EOE → EE	0.016	5.95%	0.0116	0.0198
Total indirect	**0.111**	**42.39%**	**0.0952**	**0.1263**
Direct association (*c′*)	0.150	—	0.1239	0.1763
Total association (*c*)	0.261	—	0.2381	0.2833

*N* = 2,279. Estimates (B) are unstandardized and were obtained from PROCESS Model 6 using mean composite scores, with AICSE as the predictor and EE as the outcome. Indirect estimates were evaluated using percentile bootstrap 95% confidence intervals based on 5,000 resamples. Confidence intervals for the direct and total associations are conventional regression 95% confidence intervals. Proportions of the total association were computed using unrounded estimates as (indirect association / total association) × 100%. These percentages describe the statistical decomposition of the total association, not explained variance or causal contributions; small discrepancies in their sums may occur because of rounding. Gender, age, and grade were included as covariates in all equations. Completely standardized indirect estimates (*β*_cs), with 95% percentile bootstrap confidence intervals, were: total indirect = 0.1813 [0.1570, 0.2064], via ESE = 0.0774 [0.0579, 0.0979], via EOE = 0.0785 [0.0621, 0.0956], and via ESE and EOE = 0.0254 [0.0191, 0.0324]. Arrows denote theory-specified directions for statistical estimation among concurrently measured variables, not established temporal sequences or causal effects. AICSE = AI-contextualized self-efficacy; ESE = exercise self-efficacy; EOE = exercise outcome expectations; EE = exercise engagement; CI = confidence interval; LL = lower limit; UL = upper limit.

## 5 Discussion

This study examined the association between AI-contextualized self-efficacy (AICSE) and exercise engagement in a convenience sample from one vocational college in China and tested the specific and serial statistical indirect effects involving exercise self-efficacy (ESE) and exercise outcome expectations (EOE). In the main PROCESS model, AICSE was positively associated with exercise engagement, with significant specific indirect effects through ESE, through EOE, and through their theory-specified serial pathway; the direct association remained significant. These findings suggest that general efficacy beliefs contextualized to AI support, exercise-specific efficacy beliefs, and expected exercise outcomes were jointly associated with self-reported exercise engagement. These minimally adjusted cross-sectional findings are interpreted as concurrent associations and statistical indirect effects, not evidence of temporal ordering or causal mechanisms.

### 5.1 AI-contextualized self-efficacy and exercise engagement

The results showed that AICSE was significantly and positively associated with exercise engagement, consistent with H1. In this vocational college sample, stronger beliefs about coping with difficulties and achieving goals with AI support co-occurred with higher self-reported exercise engagement. Prior studies have associated AI self-efficacy with college students’ attitudes toward AI and actual AI use (Chen et al., 2024). Related studies in technology-based exercise and physical education contexts have examined fitness app continuance usage, mobile application engagement, and learning outcomes (Huang and Ren, 2020; Zheng et al., 2026). These studies provide adjacent rather than direct evidence for the AICSE–exercise engagement association. Thus, AICSE may be a relevant psychological correlate of exercise engagement in this sample.

The association concerns concurrent self-reported efficacy beliefs and exercise engagement, not evidence that AI use improves exercise behavior. A systematic review and meta-analysis found modest and mainly short-term effects of smartphone app interventions, whereas a cluster randomized controlled trial among college students reported limited effects of a wearable activity tracker intervention (Romeo et al., 2019; Kim et al., 2018). These findings indicate that technology provision alone should not be assumed to produce sustained behavioral change. Theoretically, the finding broadens the examination of efficacy-related correlates of exercise engagement to include general beliefs contextualized to AI support. AI literacy and feedback-interpretation training could be evaluated in future intervention studies. Longitudinal designs incorporating AI-use logs, fitness app usage records, and wearable device data could further examine the AICSE–exercise engagement association.

### 5.2 Specific indirect effect through exercise self-efficacy

The specific indirect effect through ESE in the main PROCESS model was significant, consistent with H2. ESE statistically accounted for part of the concurrent association between AICSE and exercise engagement. Previous studies have reported structural associations among social support, self-efficacy, mobile-app use, and physical activity among college students (Wang et al., 2019). This provides adjacent rather than direct evidence for the present indirect association. Exercise self-regulatory efficacy among undergraduates has also been associated with exercise engagement (Jackson and Dimmock, 2012). Research on mobile exercise apps and goal setting has examined associations involving ESE, exercise barriers, and physical activity (Litman et al., 2015; Iwasaki et al., 2017). The present study extends this work by examining the specific indirect association involving ESE within a model linking AICSE with exercise engagement.

Self-Efficacy Theory emphasizes the relevance of capability beliefs to effort, persistence, and coping with difficulties (Bandura, 1977). Exercise-related efficacy beliefs are also embedded in social and environmental contexts, including teacher guidance, peer support and modeling, social norms, and opportunities for exercise, none of which were directly measured in the present model (Anderson et al., 2006; Young et al., 2014; Beauchamp et al., 2019). Recent research in college-student samples has linked exercise self-efficacy with exercise motivation, exercise climate, physical activity, and exercise adherence, while broader adult longitudinal evidence has further suggested that barrier self-efficacy may be involved in subsequent physical activity (Sheng et al., 2025; Li et al., 2024; Zhang et al., 2025). These outcomes are related to, but not equivalent to, the exercise engagement measured in the present study. This statistical indirect effect does not show that greater AICSE leads to higher ESE or establish a causal pathway to exercise engagement. Future intervention studies could test whether combining optional digital goal-setting and progress feedback with teacher guidance, peer support, and opportunities for successful exercise experiences supports ESE. Longitudinal research incorporating objective physical activity indicators could further examine associations among AICSE, ESE, and exercise engagement.

### 5.3 Specific indirect effect through exercise outcome expectations

The specific indirect effect through EOE in the main PROCESS model was significant, consistent with H3. EOE, reflecting anticipated positive exercise outcomes, statistically accounted for part of the concurrent association between AICSE and exercise engagement. In fitness-app contexts, self-efficacy, perceived benefits, and performance expectancy have been associated with app adoption, actual app use, or physical activity intention (Wei et al., 2021; Yang and Koenigstorfer, 2021). These studies provide adjacent evidence concerning technology acceptance rather than direct evidence for the AICSE–EOE association. A systematic review and meta-analysis and empirical research have linked positive outcome expectations with physical activity (Bohlen et al., 2022; Wang et al., 2025). The present study extends this inquiry by examining a specific indirect association involving EOE within a model linking AICSE and exercise engagement.

Within Social Cognitive Theory, outcome expectations are considered relevant but context-sensitive cognitive correlates of physical activity (Young et al., 2014; Beauchamp et al., 2019). In addition, previous research suggests that fulfilled emotional outcome expectations may be relevant to physical activity adoption and maintenance (Klusmann et al., 2016). However, sustained participation and maintenance were not measured in the present study and should not be inferred from exercise engagement. EOE reflects anticipated rather than realized exercise benefits. The targeted comparison supported treating EOE and the value cognition dimension of exercise engagement as related but distinct constructs (Supplementary Table S4). This statistical indirect effect does not establish that AICSE or AI feedback changes EOE or exercise engagement. Future intervention studies could test whether combining clearly explained AI-supported information with teacher guidance and actual exercise experiences supports realistic exercise outcome expectations. Longitudinal research could examine changes in EOE alongside documented AI use, exposure to specific AI feedback, and exercise outcome feedback.

### 5.4 Serial indirect effect through exercise self-efficacy and exercise outcome expectations

The serial indirect effect through ESE and EOE in the main PROCESS model was significant, consistent with H4. Figure 1 presents the conceptual model, whereas Figure 2 shows the standardized PROCESS paths. Systematic-review and meta-analytic evidence has identified self-efficacy and outcome expectations as relevant, although not uniformly supported, cognitive correlates of physical activity (Young et al., 2014; Beauchamp et al., 2019). Longitudinal and adult-sample research has also examined associations among self-efficacy, outcome expectations, and physical activity (Gothe, 2018; White et al., 2012). Related studies have examined self-efficacy and outcome expectations in older-adult neighborhood and fitness-app contexts, but not the AICSE–ESE–EOE–exercise engagement model (Ying et al., 2025; Lim and Noh, 2017). The present study extends this work by incorporating AICSE into a serial statistical model involving ESE, EOE, and exercise engagement.

Theoretically, ESE concerns perceived exercise capability, whereas EOE concerns anticipated exercise outcomes. AICSE reflects general efficacy beliefs contextualized to AI support, not observed exercise-related AI use. Previous research among women in cardiac rehabilitation has reported associations of exercise-related self-efficacy and outcome expectations with physical activity over time; however, differences in population, setting, and outcome mean that this study provides only adjacent support for considering the two constructs jointly (Blanchard et al., 2015). The serial component represented 5.95% of the total association in the main PROCESS model, indicating a modest share of its statistical decomposition (Table 4). The context-dependent role of outcome expectations also cautions against a universal interpretation of this pathway (Anderson et al., 2006). Supplementary covariate-adjusted SEMs showed identical fit for the ESE → EOE and EOE → ESE orderings, so model fit did not favor the hypothesized ordering (Supplementary Table S5; Supplementary Figure S1). Both models yielded significant total indirect associations, whereas serial-specific inference rests on PROCESS Model 6 (Supplementary Table S6). Thus, the specified ordering does not establish temporal precedence or causality, and reciprocal associations remain possible. Future intervention studies could test whether optional digital support combined with teacher guidance, peer support, and successful exercise experiences supports ESE and realistic EOE. Longitudinal studies incorporating AI-use logs and wearable device data could further examine temporal relationships and assess long-term exercise adherence directly.

## 6 Practical implications

The findings suggest examining AICSE, ESE, and EOE alongside exercise engagement in future intervention research. The following proposals are directions for testing, rather than applications validated by the present study.

Future intervention studies could: (1) evaluate AI-supported exercise literacy modules in physical education and health education, covering generative AI, exercise apps, wearable devices, and smart physical education platforms, with a focus on critical appraisal of recommendations, feedback interpretation, planning, goal adjustment, and independent exercise decisions; (2) test whether personalized goal setting, staged exercise tasks, progress feedback, and optional exercise-barrier coping prompts support ESE when students face time constraints, fatigue, lack of companions, or limited exercise opportunities; (3) examine whether exercise logs, AI feedback reports, visual progress records, and staged summaries, discussed alongside actual exercise experiences and teacher guidance, support realistic EOE; and (4) incorporate teacher review to explain, screen, and correct AI-generated recommendations rather than treating them as professional exercise prescriptions, alongside safeguards for exercise safety, data privacy, and informed consent.

In addition, future studies could examine AI-supported resources integrated with physical education, mental health education, student health management, extracurricular activities, peer support, and accessible campus exercise opportunities, rather than relying on apps alone. Teachers, counselors, mental health educators, and peers could contribute modeling, encouragement, and exercise self-management support, with attention to students reporting lower AICSE or ESE or less positive EOE. Within such studies, AI resources could be evaluated as optional, gradually removable educational scaffolds, with independent goal setting, self-monitoring, and exercise decision-making as intended outcomes. Intrinsic motivation, autonomous regulation, and long-term exercise adherence should be assessed directly.

## 7 Limitations and future directions

This study provides preliminary evidence on specific and theory-specified serial statistical indirect associations involving AICSE, ESE, EOE, and exercise engagement. Several limitations should be considered.

### 7.1 Measurement limitations

The core variables in this study were measured using self-report questionnaires, which may be subject to social desirability bias, self-perception bias, and common method bias. AICSE reflects general efficacy beliefs contextualized to AI support, not AI-specific competence, actual AI use, feedback exposure, or adoption of AI recommendations. Exercise-related instructions framed general rather than exercise-specific items, limiting claims about exercise-specific AI efficacy. Similarly, exercise engagement should not be treated as equivalent to objectively measured physical activity, exercise habit, or long-term exercise adherence. The GSE-6AI response format was changed from four to five points. Although the administered Chinese version showed acceptable internal consistency and factorial validity, equivalence with the source-language version or the original four-point format was not established; direct score comparisons are therefore unwarranted. Harman’s test and the single-factor CFA comparison remain preliminary diagnostics and cannot rule out common method variance. Future research could: (1) incorporate AI-use logs, fitness app usage records, and wearable device data; (2) combine teacher evaluations, course participation records, and objective physical activity indicators; and (3) examine the stability, measurement equivalence, and exercise-context specificity of the administered AICSE measure in independent samples.

### 7.2 Study design and causal inference

The cross-sectional observational design identifies concurrent associations among AICSE, ESE, EOE, and exercise engagement, not temporal or causal relationships. The theory-specified serial indirect effects do not establish psychological mechanisms or exclude reverse or reciprocal associations. Future research could use longitudinal follow-up designs, cross-lagged panel models, or intervention studies to further examine the directionality and robustness of this pathway.

### 7.3 Limited adjustment for confounding factors

Regression-based and supplementary latent-variable models adjusted only for gender, age, and grade; the findings therefore represent minimally adjusted cross-sectional associations. For example, AI literacy; the frequency, consistency, and purposes of actual AI use; technology acceptance; access to digital technologies; prior exercise habits; intrinsic, autonomous, and controlled motivation; teacher and peer support; campus exercise opportunities; physical health status; academic stress; and mental health status may simultaneously influence the study variables and their observed associations. Future research could measure and adjust for a broader set of relevant individual, technological, and environmental factors.

### 7.4 Model robustness and analytic strategy

The primary PROCESS analyses used composite scores, while supplementary latent-variable SEMs incorporated measurement error under the specified factor structure (Supplementary Tables S5, S6). Measurement error can attenuate or inflate path coefficients and thereby distort indirect estimates (Cole and Preacher, 2014). The SEM estimates remain conditional on model specification. The two mediator orderings yielded identical fit, so model fit did not establish the proposed sequence. Measurement invariance and cross-group stability remain to be established; demographic group comparisons should therefore be interpreted cautiously. Future research could examine measurement and structural stability across gender, grade, AI-use experience, and exercise habits.

### 7.5 Sample characteristics and generalizability

The sample in this study was recruited from Yunnan Medical Health College, a single three-year vocational college, using convenience sampling. The institution has a medical and health education context that may be associated with greater exposure to health-related knowledge and digital health resources. However, specific academic-major information was not collected; participants therefore could not be classified at the individual level as medical, health-related, or other majors, and the possible influence of disciplinary background on the study variables and their associations could not be examined. Caution is therefore needed when generalizing the findings to students from other regions, disciplinary backgrounds, undergraduate institutions, comprehensive universities, or sports universities. Although paper-based and online formats used identical items, instructions, and response options, response-mode differences remain possible. Because class or program-level clustering information was not modeled, potential non-independence among students from the same classes or academic programs could not be fully addressed. Future research could use multi-region, multi-institution, and multi-stage or clustered sampling; collect specific academic-major, class-level, and program-level information; and examine whether the observed associations vary across disciplinary backgrounds.

### 7.6 Future research directions and applications

Future research could explore the following directions. First, longitudinal follow-up studies, cross-lagged panel models, or intervention studies could further examine associations among AICSE, ESE, EOE, and exercise engagement. Second, AI-use logs, fitness app usage records, wearable device data, and physical education course participation records could be combined to document the frequency, consistency, purposes, and feedback exposure of actual AI use and to improve measurement objectivity. Third, multi-institutional studies could compare the roles of generative AI, exercise apps, wearable devices, and smart physical education platforms across academic majors while also examining teacher support, peer influences, motivational regulation, and campus exercise conditions. Fourth, AI-supported exercise literacy interventions could test whether feedback interpretation, goal setting, exercise-barrier coping, and progress visualization, combined with gradually reduced AI prompts, support independent exercise planning and self-management. Such studies should directly assess motivational regulation and long-term exercise adherence rather than using exercise engagement as a substitute for these outcomes.

## 8 Conclusion

This study examined the association between AI-contextualized self-efficacy (AICSE) and self-reported exercise engagement in a convenience sample from one vocational college in China. In the main PROCESS model, AICSE was significantly and positively associated with exercise engagement, with significant specific indirect effects via ESE and via EOE and a significant serial indirect effect in the theory-specified ESE–EOE order. Drawing on Self-Efficacy Theory and Social Cognitive Theory, the study considers general efficacy beliefs contextualized to AI support alongside exercise-specific efficacy beliefs and anticipated exercise outcomes. Future interventions could evaluate AI-supported exercise resources alongside teacher guidance and peer support, with independent exercise self-management as an intended outcome.

These minimally adjusted cross-sectional findings do not establish temporal ordering or causal mechanisms; actual AI use, feedback adoption, objective physical activity, and long-term exercise adherence were not measured. Supplementary SEMs yielded identical fit for the two mediator orderings, so model fit did not favor the hypothesized sequence. Future multi-institutional longitudinal and intervention studies incorporating AI-use records and objective physical activity measures could examine temporal relationships and generalizability.

## Data Availability

The original contributions presented in the study are included in the article/Supplementary material, further inquiries can be directed to the corresponding author.
