# Supplementary material for: AI-contextualized self-efficacy and exercise engagement among vocational college students in China: a cross-sectional serial mediation analysis of exercise self-efficacy and exercise outcome expectations
Source: Front Psychol. 2026 Oct 6;17:1933716. doi: 10.3389/fpsyg.2026.1933716 (PMC13680253; doi:10.3389/fpsyg.2026.1933716)
Supplement: Supplementary file 1 [file Data_Sheet_2.pdf]

Supplementary Material

1 Supplementary Tables

Supplementary Table S1. Standardized factor loadings, composite reliability, and average variance extracted for the measurement model

Construct / Factor	Indicator	Standardized loading (λ)	CR	AVE
Panel A. Item-level measurement model				
**AI-Contextualized Self-Efficacy (AICSE)**	AISE_1	0.721	0.883	0.556
	AISE_2	0.757		
	AISE_3	0.736		
	AISE_4	0.748		
	AISE_5	0.760		
	AISE_6	0.752		
**Exercise Outcome Expectations (EOE)**	EOE_1	0.720	0.910	0.528
	EOE_2	0.727		
	EOE_3	0.721		
	EOE_4	0.724		
	EOE_5	0.738		
	EOE_6	0.716		
	EOE_7	0.745		
	EOE_8	0.728		
	EOE_9	0.718		
**ESE: Situational motivation (Sm)**	ESE_1	0.711	0.870	0.527
	ESE_2	0.733		
	ESE_3	0.713		
	ESE_4	0.739		
	ESE_5	0.739		
	ESE_6	0.721		

Construct / Factor	Indicator	Standardized loading (λ)	CR	AVE
	ESE_7	0.744		
	ESE_8	0.747		
**ESE: Subjective support (Ss)**			0.825	0.541
	ESE_9	0.716		
	ESE_10	0.735		
	EE_1	0.719		
	EE_2	0.722		
	EE_3	0.744		
**EE: Vigor persistence (Vp)**			0.873	0.533
	EE_4	0.732		
	EE_5	0.726		
	EE_6	0.740		
	EE_7	0.729		
	EE_8	0.715		
	EE_9	0.739		
**EE: Focused satisfaction (Fs)**			0.874	0.535
	EE_10	0.737		
	EE_11	0.736		
	EE_12	0.735		
	EE_13	0.750		
	EE_14	0.734		
**EE: Value cognition (Vc)**	EE_15	0.749	0.859	0.550
	EE_16	0.735		
	EE_17	0.738		
	EE_18	0.738		
**EE: Participation autonomy (Pa)**	EE_19	0.714	0.769	0.526
	EE_20	0.723		
Panel B. Higher-order measurement model				
	Situational motivation (Sm)	0.810		
**Exercise Self-Efficacy (ESE)**			0.778	0.637
	Subjective support (Ss)	0.786		

**Construct / Factor**	**Indicator**	**Standardized loading (λ)**	**CR**	**AVE**
**Exercise Engagement (EE)**	Vigor persistence (Vp)	0.807	0.886	0.659
	Focused satisfaction (Fs)	0.814		
	Value cognition (Vc)	0.824		
	Participation autonomy (Pa)	0.802		

Note. N = 2,279. Loadings are standardized estimates from the complete confirmatory factor analysis measurement model. Panel A reports item loadings on first-order factors; Panel B reports first-order factor loadings on the higher-order ESE and EE constructs. CR and AVE are reported once per factor at the corresponding measurement level and were computed from unrounded standardized loadings. The higher-order CR and AVE values in Panel B are not reliability estimates for the observed mean composite scores. Values are rounded to three decimal places. AICSE = AI-contextualized self-efficacy; ESE = exercise self-efficacy; EOE = exercise outcome expectations; EE = exercise engagement; λ = standardized factor loading; CR = composite reliability; AVE = average variance extracted. The construct reported as AICSE is named AISE in the analysis files; AISE_1–AISE_6 are its original item identifiers.

Supplementary Table S2. Latent-factor correlations and Fornell–Larcker discriminant validity

Construct	1	2	3	4
1. AI-Contextualized Self-Efficacy (AICSE)	**0.746**			
2. Exercise Outcome Expectations (EOE)	0.553	**0.726**		
3. Exercise Self-Efficacy (ESE)	0.597	0.541	**0.798**	
4. Exercise Engagement (EE)	0.507	0.476	0.500	**0.812**

Note. N = 2,279. Bold diagonal values are the square roots of AVE; lower-triangular values are latent-factor correlations from the complete CFA measurement model used in Supplementary Table S1. The square roots of AVE were calculated from unrounded standardized loadings before rounding to three decimal places. AICSE and EOE are first-order factors, whereas ESE and EE are higher-order factors; their AVE values correspond to the respective measurement levels reported in Supplementary Table S1. These are not Pearson correlations between observed composite scores. Under the Fornell–Larcker criterion used here, each diagonal value is compared with the absolute values of the corresponding construct's correlations with the other constructs. AICSE = AI-contextualized self-efficacy (named AISE in the analysis files); ESE = exercise self-efficacy; EOE = exercise outcome expectations; EE = exercise engagement; CFA = confirmatory factor analysis; AVE = average variance extracted.

Supplementary Table S3. Model fit comparison of the complete measurement model and the single-factor model

Model	χ^2	*df*	χ^2/df	RMSEA	CFI	TLI	AIC
Complete measurement model	1,035.773	933	1.110	0.007	0.998	0.998	1,239.773
Single-factor model	23,193.606	945	24.543	0.102	0.546	0.524	23,373.606

Note. N = 2,279. Both models were estimated in AMOS using maximum likelihood and the same 45 measurement items. The complete measurement model is the CFA model reported in Supplementary Tables S1 and S2: AICSE and EOE are first-order factors, whereas ESE and EE are higher-order factors reflected by two and four first-order dimensions, respectively; the four core constructs were allowed to correlate. In the single-factor model, all 45 items load on one common factor. This comparison is a supplementary diagnostic of the measurement structure and does not rule out common method variance. χ^2 = model chi-square; df = degrees of freedom; RMSEA = root mean square error of approximation; CFI = comparative fit index; TLI = Tucker–Lewis index; AIC = Akaike information criterion; CFA = confirmatory factor analysis; AICSE = AI-contextualized self-efficacy; ESE = exercise self-efficacy; EOE = exercise outcome expectations; EE = exercise engagement.

Supplementary Table S4. Targeted discriminant-validity analysis of exercise outcome expectations and value cognition

Panel A. Measurement evidence from the two-factor model

Factor	Indicator	Standardized loading (λ)	CR	AVE	√AVE
**Exercise Outcome Expectations (EOE)**	EOE_1	0.720	0.910	0.528	0.726
	EOE_2	0.728			
	EOE_3	0.721			
	EOE_4	0.721			
	EOE_5	0.738			
	EOE_6	0.718			
	EOE_7	0.746			
	EOE_8	0.727			
	EOE_9	0.718			
**Value Cognition (Vc)**	EE_13	0.747	0.859	0.550	0.741
	EE_14	0.735			
	EE_15	0.747			
	EE_16	0.737			
	EE_17	0.740			

Latent-factor correlation between EOE and Vc: r = 0.389.

Panel B. Model fit comparison (14 items)

Model	χ^2	df	χ^2/df	RMSEA	CFI	TLI	AIC
Two-factor model	119.346	76	1.570	0.016	0.997	0.997	177.346
Single-factor model	3,889.447	77	50.512	0.147	0.743	0.696	3,945.447

Note. N = 2,279. Both models were estimated in AMOS using maximum likelihood and the same 14 items. In the two-factor model, EOE_1–EOE_9 load on EOE and EE_13–EE_17 load on Vc; the two factors were allowed to correlate. In the single-factor model, all 14 items load on one factor. Panel A reports estimates from the targeted two-factor model, not the complete measurement model in Supplementary Table S1. CR, AVE, and √AVE were computed from unrounded standardized loadings and rounded to three decimal places. CR and AVE are reported once per factor. For the Fornell–Larcker comparison, each factor's √AVE is compared with the absolute latent-factor correlation. Vc is the value cognition dimension of exercise engagement (EE), not the overall EE construct. EOE = exercise outcome expectations; λ = standardized factor loading; CR = composite reliability; AVE = average variance extracted; χ^2 = model chi-square; df = degrees of freedom; RMSEA = root mean square error of approximation; CFI = comparative fit index; TLI = Tucker–Lewis index; AIC = Akaike information criterion.

Supplementary Table S5. Model fit and structural path estimates for the covariate-adjusted latent-variable models

Panel A. Model fit comparison

Model	χ^2	*df*	χ^2/df	RMSEA	CFI	TLI	AIC
Model A (ESE → EOE)	1,193.609	1056	1.130	0.008	0.997	0.997	1,433.609
Model B (EOE → ESE)	1,193.609	1056	1.130	0.008	0.997	0.997	1,433.609

Panel B. Structural path estimates

Model A: Theory-specified ordering (ESE → EOE)

Path	*B*	SE	C.R.	*p*	β
AICSE → ESE	0.336	0.018	19.001	<0.001	0.593
Gender → ESE	−0.054	0.020	−2.707	0.007	−0.060
Age → ESE	0.005	0.011	0.433	0.665	0.010
Grade → ESE	0.014	0.011	1.192	0.233	0.026
AICSE → EOE	0.235	0.020	11.825	<0.001	0.354
ESE → EOE	0.382	0.040	9.663	<0.001	0.326
Gender → EOE	−0.016	0.020	−0.828	0.408	−0.015
Age → EOE	0.018	0.011	1.652	0.098	0.031
Grade → EOE	0.011	0.011	0.985	0.325	0.018
AICSE → EE	0.142	0.018	7.793	<0.001	0.249
ESE → EE	0.232	0.037	6.334	<0.001	0.231
EOE → EE	0.176	0.025	7.131	<0.001	0.206
Gender → EE	−0.049	0.018	−2.704	0.007	−0.054
Age → EE	0.016	0.010	1.623	0.105	0.032
Grade → EE	0.014	0.010	1.375	0.169	0.027

Model B: Alternative ordering (EOE → ESE)

Path	*B*	SE	C.R.	*p*	β
AICSE → EOE	0.363	0.017	21.213	<0.001	0.547
Gender → EOE	−0.037	0.020	−1.829	0.067	−0.035
Age → EOE	0.020	0.011	1.768	0.077	0.034
Grade → EOE	0.016	0.012	1.406	0.160	0.027
AICSE → ESE	0.242	0.018	13.378	<0.001	0.428
EOE → ESE	0.257	0.025	10.078	<0.001	0.301
Gender → ESE	−0.045	0.019	−2.311	0.021	−0.049
Age → ESE	0.000[a]	0.011	−0.029	0.977	−0.001
Grade → ESE	0.009	0.011	0.855	0.392	0.018
AICSE → EE	0.142	0.018	7.793	<0.001	0.249
ESE → EE	0.232	0.037	6.334	<0.001	0.231
EOE → EE	0.176	0.025	7.131	<0.001	0.206
Gender → EE	−0.049	0.018	−2.704	0.007	−0.054
Age → EE	0.016	0.010	1.623	0.105	0.032
Grade → EE	0.014	0.010	1.375	0.169	0.027

Note. N = 2,279. Both models were estimated by maximum likelihood in AMOS and retained the item–factor structure reported in Supplementary Table S1. Gender, age, and grade were observed covariates predicting ESE, EOE, and EE; AICSE and the three covariates were allowed to covary. Gender was coded 1 = male and 2 = female; age was coded 1 = 18 years, 2 = 19 years, 3 = 20 years, and 4 = 21 years or older; grade was coded 1 = first year, 2 = second year, and 3 = third year. Age and grade were each entered as a single numerically coded covariate, not as dummy variables. B = unstandardized regression coefficient; SE = standard error of B; C.R. = critical ratio (B/SE, calculated from unrounded estimates); β = standardized regression coefficient. The p values are from the maximum-likelihood regression-weight output, not bootstrap tests. These minimally adjusted cross-sectional models are supplementary sensitivity analyses; arrows denote specified statistical directions, not established temporal or causal relationships. χ^2 = model chi-square; df = degrees of freedom; RMSEA = root mean square error of approximation; CFI = comparative fit index; TLI = Tucker–Lewis index; AIC = Akaike information criterion. AICSE = AI-contextualized self-efficacy (AISE in the analysis files); ESE = exercise self-efficacy; EOE = exercise outcome expectations; EE = exercise engagement.

[a] The unstandardized Age → ESE estimate in Model B is −0.00031094; the value 0.000 reflects rounding, not an exact zero.

Supplementary Table S6. Standardized association estimates and bootstrap confidence intervals for the covariate-adjusted latent-variable models

Path / component	Standardized estimate	Bootstrap SE	95% percentile CI		p
			Lower	Upper	
**Model A: Theory-specified ordering (ESE → EOE)**					
AICSE → ESE → EE	0.137	0.022	0.0952	0.1802	<0.001
AICSE → EOE → EE	0.073	0.011	0.0520	0.0953	<0.001
**Total indirect association**	**0.250**	**0.021**	**0.2082**	**0.2922**	**<0.001**
Direct association (c')	0.249	0.031	0.1884	0.3091	<0.001
Total association (c)	0.499	0.019	0.4620	0.5370	<0.001
**Model B: Alternative ordering (EOE → ESE)**					
AICSE → ESE → EE	0.099	0.017	0.0674	0.1329	<0.001
AICSE → EOE → EE	0.113	0.015	0.0838	0.1426	<0.001
**Total indirect association**	**0.250**	**0.021**	**0.2082**	**0.2922**	**<0.001**
Direct association (c')	0.249	0.031	0.1884	0.3091	<0.001
Total association (c)	0.499	0.019	0.4620	0.5370	<0.001

Note. N = 2,279. Models A and B are the covariate-adjusted latent-variable models reported in Supplementary Table S5. Gender, age, and grade were included as covariates in the ESE, EOE, and EE equations in both models. All estimates are standardized and concern the AICSE–EE association. The first two rows of each model are specific indirect estimates from that complete model, not separate single-mediator analyses. The total indirect estimate includes the two displayed specific indirect estimates and the serial component; the two displayed components therefore do not sum to the total indirect estimate. No separate bootstrap CI for the serial-specific component was obtained, and no separate test of that component is reported here. Total association = direct association + total indirect association. Bootstrap SEs, percentile 95% CIs, and bootstrap p values were obtained from the AMOS output using 5,000 usable bootstrap samples per model; this applies to indirect, direct, and total estimates. Estimates and SEs are rounded to three decimal places; CI limits are rounded to four decimal places from unrounded output. Arrows denote specified statistical directions among concurrently measured variables, not established temporal sequences or causal effects. AICSE = AI-contextualized self-efficacy (AISE in the analysis files); ESE = exercise self-efficacy; EOE = exercise outcome expectations; EE = exercise engagement; SE = standard error; CI = confidence interval.
