# Supplementary material for: AI-contextualized self-efficacy and exercise engagement among vocational college students in China: a cross-sectional serial mediation analysis of exercise self-efficacy and exercise outcome expectations
Source: Front Psychol. 2026 Oct 6;17:1933716. doi: 10.3389/fpsyg.2026.1933716 (PMC13680253; doi:10.3389/fpsyg.2026.1933716)
Supplement: Supplementary file 2 [file Data_Sheet_1.zip › Revision_Package_1933716/SEM_Model_2/SEM_Model_2.AmosOutput]

结构方程模型2.amw


# D:\系统默认\桌面\danzi\2025年9月份单子\361.数据分析-220元\结构方程模型2.amw

## Analysis Summary

## Date and Time

Date: 2026年9月13日

Time: 17:44:20

## Title

结构方程模型2: 2026年9月13日 17:44

## Groups

## Group number 1 (Group number 1)

## Notes for Group (Group number 1)

The model is recursive.

Sample size = 2279

## Variable Summary (Group number 1)

## Your model contains the following variables (Group number 1)

Observed, endogenous variables

AISE\_1

AISE\_2

AISE\_3

AISE\_4

AISE\_5

AISE\_6

EOE\_1

EOE\_2

EOE\_3

EOE\_4

EOE\_5

EOE\_6

EOE\_7

EOE\_8

EOE\_9

ESE\_1

ESE\_2

ESE\_3

ESE\_4

ESE\_5

ESE\_6

ESE\_7

ESE\_8

ESE\_9

ESE\_10

EE\_1

EE\_2

EE\_3

EE\_4

EE\_5

EE\_6

EE\_7

EE\_8

EE\_9

EE\_10

EE\_11

EE\_12

EE\_13

EE\_14

EE\_15

EE\_16

EE\_17

EE\_18

EE\_19

EE\_20

Observed, exogenous variables

Gender

Age

Grade

Unobserved, endogenous variables

EOE

Sm

Ss

ESE

Vp

Fs

Vc

Pa

EE

Unobserved, exogenous variables

AISE

e1

e2

e3

e4

e5

e6

e7

e8

e9

e10

e11

e12

e13

e14

e15

e16

e17

e18

e19

e20

e21

e22

e23

e24

e25

e26

e27

e28

e29

e30

e31

e32

e33

e34

e35

e36

e37

e38

e39

e40

e41

e42

e43

e44

e45

e46

e47

e48

e49

e50

e51

e52

e53

e54

## Variable counts (Group number 1)

|  |  |
| --- | --- |
| Number of variables in your model: | 112 |
| Number of observed variables: | 48 |
| Number of unobserved variables: | 64 |
| Number of exogenous variables: | 58 |
| Number of endogenous variables: | 54 |

## Parameter Summary (Group number 1)

|  | Weights | Covariances | Variances | Means | Intercepts | Total |
| --- | --- | --- | --- | --- | --- | --- |
| Fixed | 64 | 0 | 0 | 0 | 0 | 64 |
| Labeled | 0 | 0 | 0 | 0 | 0 | 0 |
| Unlabeled | 56 | 6 | 58 | 0 | 0 | 120 |
| Total | 120 | 6 | 58 | 0 | 0 | 184 |

## Models

## Default model (Default model)

## Notes for Model (Default model)

## Computation of degrees of freedom (Default model)

|  |  |
| --- | --- |
| Number of distinct sample moments: | 1176 |
| Number of distinct parameters to be estimated: | 120 |
| Degrees of freedom (1176 - 120): | 1056 |

## Result (Default model)

Minimum was achieved

Chi-square = 1193.609

Degrees of freedom = 1056

Probability level = .002

## Group number 1 (Group number 1 - Default model)

## Estimates (Group number 1 - Default model)

## Scalar Estimates (Group number 1 - Default model)

## Maximum Likelihood Estimates

## Regression Weights: (Group number 1 - Default model)

|  |  |  | Estimate | S.E. | C.R. | P | Label |
| --- | --- | --- | --- | --- | --- | --- | --- |
| EOE | <--- | AISE | .363 | .017 | 21.213 | \*\*\* |  |
| EOE | <--- | Gender | -.037 | .020 | -1.829 | .067 |  |
| EOE | <--- | Age | .020 | .011 | 1.768 | .077 |  |
| EOE | <--- | Grade | .016 | .012 | 1.406 | .160 |  |
| ESE | <--- | AISE | .242 | .018 | 13.378 | \*\*\* |  |
| ESE | <--- | EOE | .257 | .025 | 10.078 | \*\*\* |  |
| ESE | <--- | Gender | -.045 | .019 | -2.311 | .021 |  |
| ESE | <--- | Age | .000 | .011 | -.029 | .977 |  |
| ESE | <--- | Grade | .009 | .011 | .855 | .392 |  |
| EE | <--- | ESE | .232 | .037 | 6.334 | \*\*\* |  |
| EE | <--- | EOE | .176 | .025 | 7.131 | \*\*\* |  |
| EE | <--- | AISE | .142 | .018 | 7.793 | \*\*\* |  |
| EE | <--- | Gender | -.049 | .018 | -2.704 | .007 |  |
| EE | <--- | Age | .016 | .010 | 1.623 | .105 |  |
| EE | <--- | Grade | .014 | .010 | 1.375 | .169 |  |
| Sm | <--- | ESE | 1.000 |  |  |  |  |
| Ss | <--- | ESE | .993 | .050 | 19.747 | \*\*\* |  |
| Vp | <--- | EE | 1.000 |  |  |  |  |
| Fs | <--- | EE | 1.034 | .042 | 24.495 | \*\*\* |  |
| Vc | <--- | EE | 1.062 | .043 | 24.956 | \*\*\* |  |
| Pa | <--- | EE | .978 | .041 | 23.799 | \*\*\* |  |
| AISE\_1 | <--- | AISE | 1.000 |  |  |  |  |
| AISE\_2 | <--- | AISE | .983 | .029 | 34.120 | \*\*\* |  |
| AISE\_3 | <--- | AISE | .959 | .029 | 33.199 | \*\*\* |  |
| AISE\_4 | <--- | AISE | .991 | .029 | 33.721 | \*\*\* |  |
| AISE\_5 | <--- | AISE | .955 | .028 | 34.273 | \*\*\* |  |
| AISE\_6 | <--- | AISE | .999 | .029 | 33.915 | \*\*\* |  |
| EOE\_1 | <--- | EOE | 1.000 |  |  |  |  |
| EOE\_2 | <--- | EOE | 1.014 | .030 | 33.435 | \*\*\* |  |
| EOE\_3 | <--- | EOE | 1.002 | .030 | 33.154 | \*\*\* |  |
| EOE\_4 | <--- | EOE | .993 | .030 | 33.279 | \*\*\* |  |
| EOE\_5 | <--- | EOE | 1.032 | .030 | 33.927 | \*\*\* |  |
| EOE\_6 | <--- | EOE | .992 | .030 | 32.940 | \*\*\* |  |
| EOE\_7 | <--- | EOE | 1.030 | .030 | 34.275 | \*\*\* |  |
| EOE\_8 | <--- | EOE | 1.024 | .031 | 33.469 | \*\*\* |  |
| EOE\_9 | <--- | EOE | 1.001 | .030 | 33.018 | \*\*\* |  |
| ESE\_1 | <--- | Sm | 1.000 |  |  |  |  |
| ESE\_2 | <--- | Sm | 1.028 | .032 | 32.284 | \*\*\* |  |
| ESE\_3 | <--- | Sm | .982 | .031 | 31.436 | \*\*\* |  |
| ESE\_4 | <--- | Sm | 1.047 | .032 | 32.536 | \*\*\* |  |
| ESE\_5 | <--- | Sm | 1.033 | .032 | 32.522 | \*\*\* |  |
| ESE\_6 | <--- | Sm | 1.028 | .032 | 31.763 | \*\*\* |  |
| ESE\_7 | <--- | Ss | 1.000 |  |  |  |  |
| ESE\_8 | <--- | Ss | 1.005 | .031 | 32.650 | \*\*\* |  |
| ESE\_9 | <--- | Ss | .947 | .030 | 31.413 | \*\*\* |  |
| ESE\_10 | <--- | Ss | .981 | .031 | 32.148 | \*\*\* |  |
| EE\_1 | <--- | Vp | 1.000 |  |  |  |  |
| EE\_2 | <--- | Vp | 1.020 | .032 | 32.390 | \*\*\* |  |
| EE\_3 | <--- | Vp | 1.049 | .031 | 33.351 | \*\*\* |  |
| EE\_4 | <--- | Vp | 1.022 | .031 | 32.836 | \*\*\* |  |
| EE\_5 | <--- | Vp | 1.027 | .032 | 32.568 | \*\*\* |  |
| EE\_6 | <--- | Vp | 1.037 | .031 | 33.201 | \*\*\* |  |
| EE\_7 | <--- | Fs | 1.000 |  |  |  |  |
| EE\_8 | <--- | Fs | .972 | .030 | 32.521 | \*\*\* |  |
| EE\_9 | <--- | Fs | 1.007 | .030 | 33.659 | \*\*\* |  |
| EE\_10 | <--- | Fs | 1.008 | .030 | 33.530 | \*\*\* |  |
| EE\_11 | <--- | Fs | 1.003 | .030 | 33.491 | \*\*\* |  |
| EE\_12 | <--- | Fs | .994 | .030 | 33.452 | \*\*\* |  |
| EE\_13 | <--- | Vc | 1.000 |  |  |  |  |
| EE\_14 | <--- | Vc | .973 | .029 | 34.009 | \*\*\* |  |
| EE\_15 | <--- | Vc | .989 | .028 | 34.724 | \*\*\* |  |
| EE\_16 | <--- | Vc | .977 | .029 | 34.076 | \*\*\* |  |
| EE\_17 | <--- | Vc | .970 | .028 | 34.213 | \*\*\* |  |
| EE\_18 | <--- | Pa | 1.000 |  |  |  |  |
| EE\_19 | <--- | Pa | .973 | .033 | 29.598 | \*\*\* |  |
| EE\_20 | <--- | Pa | .990 | .033 | 29.868 | \*\*\* |  |

## Standardized Regression Weights: (Group number 1 - Default model)

|  |  |  | Estimate |
| --- | --- | --- | --- |
| EOE | <--- | AISE | .547 |
| EOE | <--- | Gender | -.035 |
| EOE | <--- | Age | .034 |
| EOE | <--- | Grade | .027 |
| ESE | <--- | AISE | .428 |
| ESE | <--- | EOE | .301 |
| ESE | <--- | Gender | -.049 |
| ESE | <--- | Age | -.001 |
| ESE | <--- | Grade | .018 |
| EE | <--- | ESE | .231 |
| EE | <--- | EOE | .206 |
| EE | <--- | AISE | .249 |
| EE | <--- | Gender | -.054 |
| EE | <--- | Age | .032 |
| EE | <--- | Grade | .027 |
| Sm | <--- | ESE | .811 |
| Ss | <--- | ESE | .784 |
| Vp | <--- | EE | .807 |
| Fs | <--- | EE | .813 |
| Vc | <--- | EE | .824 |
| Pa | <--- | EE | .803 |
| AISE\_1 | <--- | AISE | .721 |
| AISE\_2 | <--- | AISE | .757 |
| AISE\_3 | <--- | AISE | .736 |
| AISE\_4 | <--- | AISE | .748 |
| AISE\_5 | <--- | AISE | .760 |
| AISE\_6 | <--- | AISE | .752 |
| EOE\_1 | <--- | EOE | .720 |
| EOE\_2 | <--- | EOE | .727 |
| EOE\_3 | <--- | EOE | .721 |
| EOE\_4 | <--- | EOE | .724 |
| EOE\_5 | <--- | EOE | .738 |
| EOE\_6 | <--- | EOE | .716 |
| EOE\_7 | <--- | EOE | .745 |
| EOE\_8 | <--- | EOE | .728 |
| EOE\_9 | <--- | EOE | .718 |
| ESE\_1 | <--- | Sm | .711 |
| ESE\_2 | <--- | Sm | .733 |
| ESE\_3 | <--- | Sm | .713 |
| ESE\_4 | <--- | Sm | .739 |
| ESE\_5 | <--- | Sm | .739 |
| ESE\_6 | <--- | Sm | .721 |
| ESE\_7 | <--- | Ss | .744 |
| ESE\_8 | <--- | Ss | .748 |
| ESE\_9 | <--- | Ss | .716 |
| ESE\_10 | <--- | Ss | .735 |
| EE\_1 | <--- | Vp | .719 |
| EE\_2 | <--- | Vp | .722 |
| EE\_3 | <--- | Vp | .744 |
| EE\_4 | <--- | Vp | .732 |
| EE\_5 | <--- | Vp | .726 |
| EE\_6 | <--- | Vp | .740 |
| EE\_7 | <--- | Fs | .729 |
| EE\_8 | <--- | Fs | .715 |
| EE\_9 | <--- | Fs | .739 |
| EE\_10 | <--- | Fs | .737 |
| EE\_11 | <--- | Fs | .736 |
| EE\_12 | <--- | Fs | .735 |
| EE\_13 | <--- | Vc | .750 |
| EE\_14 | <--- | Vc | .734 |
| EE\_15 | <--- | Vc | .749 |
| EE\_16 | <--- | Vc | .735 |
| EE\_17 | <--- | Vc | .738 |
| EE\_18 | <--- | Pa | .738 |
| EE\_19 | <--- | Pa | .715 |
| EE\_20 | <--- | Pa | .723 |

## Covariances: (Group number 1 - Default model)

|  |  |  | Estimate | S.E. | C.R. | P | Label |
| --- | --- | --- | --- | --- | --- | --- | --- |
| Gender | <--> | Age | .006 | .009 | .593 | .553 |  |
| Gender | <--> | Grade | -.006 | .009 | -.620 | .536 |  |
| Age | <--> | Grade | .017 | .016 | 1.026 | .305 |  |
| AISE | <--> | Gender | -.017 | .009 | -2.016 | .044 |  |
| AISE | <--> | Age | .070 | .016 | 4.373 | \*\*\* |  |
| AISE | <--> | Grade | .015 | .015 | 1.018 | .309 |  |

## Correlations: (Group number 1 - Default model)

|  |  |  | Estimate |
| --- | --- | --- | --- |
| Gender | <--> | Age | .012 |
| Gender | <--> | Grade | -.013 |
| Age | <--> | Grade | .021 |
| AISE | <--> | Gender | -.045 |
| AISE | <--> | Age | .098 |
| AISE | <--> | Grade | .023 |

## Variances: (Group number 1 - Default model)

|  |  |  | Estimate | S.E. | C.R. | P | Label |
| --- | --- | --- | --- | --- | --- | --- | --- |
| AISE |  |  | .623 | .033 | 19.074 | \*\*\* |  |
| Gender |  |  | .242 | .007 | 33.749 | \*\*\* |  |
| Age |  |  | .816 | .024 | 33.749 | \*\*\* |  |
| Grade |  |  | .743 | .022 | 33.749 | \*\*\* |  |
| e52 |  |  | .190 | .010 | 18.567 | \*\*\* |  |
| e53 |  |  | .115 | .009 | 12.099 | \*\*\* |  |
| e54 |  |  | .131 | .009 | 14.763 | \*\*\* |  |
| e46 |  |  | .103 | .010 | 10.792 | \*\*\* |  |
| e47 |  |  | .123 | .010 | 11.924 | \*\*\* |  |
| e48 |  |  | .108 | .007 | 14.500 | \*\*\* |  |
| e49 |  |  | .110 | .008 | 14.458 | \*\*\* |  |
| e50 |  |  | .107 | .008 | 14.096 | \*\*\* |  |
| e51 |  |  | .106 | .008 | 12.674 | \*\*\* |  |
| e1 |  |  | .576 | .020 | 29.266 | \*\*\* |  |
| e2 |  |  | .449 | .016 | 28.182 | \*\*\* |  |
| e3 |  |  | .485 | .017 | 28.848 | \*\*\* |  |
| e4 |  |  | .483 | .017 | 28.485 | \*\*\* |  |
| e5 |  |  | .415 | .015 | 28.060 | \*\*\* |  |
| e6 |  |  | .477 | .017 | 28.341 | \*\*\* |  |
| e7 |  |  | .255 | .008 | 30.319 | \*\*\* |  |
| e8 |  |  | .251 | .008 | 30.167 | \*\*\* |  |
| e9 |  |  | .255 | .008 | 30.291 | \*\*\* |  |
| e10 |  |  | .246 | .008 | 30.237 | \*\*\* |  |
| e11 |  |  | .245 | .008 | 29.935 | \*\*\* |  |
| e12 |  |  | .256 | .008 | 30.382 | \*\*\* |  |
| e13 |  |  | .233 | .008 | 29.758 | \*\*\* |  |
| e14 |  |  | .256 | .008 | 30.151 | \*\*\* |  |
| e15 |  |  | .258 | .009 | 30.349 | \*\*\* |  |
| e16 |  |  | .296 | .010 | 29.041 | \*\*\* |  |
| e17 |  |  | .275 | .010 | 28.398 | \*\*\* |  |
| e18 |  |  | .283 | .010 | 28.999 | \*\*\* |  |
| e19 |  |  | .275 | .010 | 28.200 | \*\*\* |  |
| e20 |  |  | .269 | .010 | 28.211 | \*\*\* |  |
| e21 |  |  | .296 | .010 | 28.779 | \*\*\* |  |
| e22 |  |  | .257 | .010 | 25.802 | \*\*\* |  |
| e23 |  |  | .255 | .010 | 25.651 | \*\*\* |  |
| e24 |  |  | .272 | .010 | 27.032 | \*\*\* |  |
| e25 |  |  | .262 | .010 | 26.261 | \*\*\* |  |
| e26 |  |  | .288 | .010 | 29.165 | \*\*\* |  |
| e27 |  |  | .296 | .010 | 29.103 | \*\*\* |  |
| e28 |  |  | .275 | .010 | 28.455 | \*\*\* |  |
| e29 |  |  | .280 | .010 | 28.817 | \*\*\* |  |
| e30 |  |  | .293 | .010 | 28.992 | \*\*\* |  |
| e31 |  |  | .274 | .010 | 28.565 | \*\*\* |  |
| e32 |  |  | .287 | .010 | 28.955 | \*\*\* |  |
| e33 |  |  | .294 | .010 | 29.336 | \*\*\* |  |
| e34 |  |  | .273 | .010 | 28.639 | \*\*\* |  |
| e35 |  |  | .278 | .010 | 28.725 | \*\*\* |  |
| e36 |  |  | .277 | .010 | 28.751 | \*\*\* |  |
| e37 |  |  | .273 | .010 | 28.776 | \*\*\* |  |
| e38 |  |  | .260 | .009 | 27.700 | \*\*\* |  |
| e39 |  |  | .271 | .010 | 28.270 | \*\*\* |  |
| e40 |  |  | .256 | .009 | 27.754 | \*\*\* |  |
| e41 |  |  | .271 | .010 | 28.224 | \*\*\* |  |
| e42 |  |  | .262 | .009 | 28.129 | \*\*\* |  |
| e43 |  |  | .250 | .010 | 24.270 | \*\*\* |  |
| e44 |  |  | .270 | .011 | 25.504 | \*\*\* |  |
| e45 |  |  | .266 | .011 | 25.075 | \*\*\* |  |

## User-defined estimands: (Group number 1 - Default model)

|  |  |  |  |
| --- | --- | --- | --- |
| MedIndirect1 |  |  | .099 |
| MedDirect1 |  |  | .249 |
| MedTotal1 |  |  | .348 |
| MedIndirect2 |  |  | .113 |
| MedDirect2 |  |  | .249 |
| MedTotal2 |  |  | .362 |
| MedIndirect3 |  |  | .250 |
| MedDirect3 |  |  | .249 |
| MedTotal3 |  |  | .499 |

## Matrices (Group number 1 - Default model)

## Total Effects (Group number 1 - Default model)

|  | Grade | Age | Gender | AISE | EOE | ESE | EE | Pa | Vc | Fs | Vp | Ss | Sm |
| --- | --- | --- | --- | --- | --- | --- | --- | --- | --- | --- | --- | --- | --- |
| EOE | .016 | .020 | -.037 | .363 | .000 | .000 | .000 | .000 | .000 | .000 | .000 | .000 | .000 |
| ESE | .014 | .005 | -.054 | .336 | .257 | .000 | .000 | .000 | .000 | .000 | .000 | .000 | .000 |
| EE | .020 | .021 | -.068 | .283 | .236 | .232 | .000 | .000 | .000 | .000 | .000 | .000 | .000 |
| Pa | .020 | .020 | -.067 | .277 | .231 | .227 | .978 | .000 | .000 | .000 | .000 | .000 | .000 |
| Vc | .021 | .022 | -.072 | .301 | .250 | .246 | 1.062 | .000 | .000 | .000 | .000 | .000 | .000 |
| Fs | .021 | .021 | -.070 | .293 | .244 | .240 | 1.034 | .000 | .000 | .000 | .000 | .000 | .000 |
| Vp | .020 | .021 | -.068 | .283 | .236 | .232 | 1.000 | .000 | .000 | .000 | .000 | .000 | .000 |
| Ss | .014 | .005 | -.054 | .333 | .255 | .993 | .000 | .000 | .000 | .000 | .000 | .000 | .000 |
| Sm | .014 | .005 | -.054 | .336 | .257 | 1.000 | .000 | .000 | .000 | .000 | .000 | .000 | .000 |
| EE\_20 | .020 | .020 | -.066 | .274 | .228 | .225 | .968 | .990 | .000 | .000 | .000 | .000 | .000 |
| EE\_19 | .019 | .020 | -.065 | .270 | .224 | .221 | .952 | .973 | .000 | .000 | .000 | .000 | .000 |
| EE\_18 | .020 | .020 | -.067 | .277 | .231 | .227 | .978 | 1.000 | .000 | .000 | .000 | .000 | .000 |
| EE\_17 | .021 | .021 | -.070 | .292 | .243 | .239 | 1.029 | .000 | .970 | .000 | .000 | .000 | .000 |
| EE\_16 | .021 | .021 | -.071 | .294 | .245 | .241 | 1.037 | .000 | .977 | .000 | .000 | .000 | .000 |
| EE\_15 | .021 | .022 | -.072 | .298 | .248 | .244 | 1.050 | .000 | .989 | .000 | .000 | .000 | .000 |
| EE\_14 | .021 | .021 | -.070 | .293 | .244 | .240 | 1.033 | .000 | .973 | .000 | .000 | .000 | .000 |
| EE\_13 | .021 | .022 | -.072 | .301 | .250 | .246 | 1.062 | .000 | 1.000 | .000 | .000 | .000 | .000 |
| EE\_12 | .021 | .021 | -.070 | .291 | .242 | .238 | 1.027 | .000 | .000 | .994 | .000 | .000 | .000 |
| EE\_11 | .021 | .021 | -.071 | .294 | .245 | .241 | 1.037 | .000 | .000 | 1.003 | .000 | .000 | .000 |
| EE\_10 | .021 | .021 | -.071 | .295 | .246 | .242 | 1.042 | .000 | .000 | 1.008 | .000 | .000 | .000 |
| EE\_9 | .021 | .021 | -.071 | .295 | .246 | .242 | 1.041 | .000 | .000 | 1.007 | .000 | .000 | .000 |
| EE\_8 | .020 | .021 | -.068 | .285 | .237 | .233 | 1.005 | .000 | .000 | .972 | .000 | .000 | .000 |
| EE\_7 | .021 | .021 | -.070 | .293 | .244 | .240 | 1.034 | .000 | .000 | 1.000 | .000 | .000 | .000 |
| EE\_6 | .021 | .021 | -.071 | .294 | .245 | .241 | 1.037 | .000 | .000 | .000 | 1.037 | .000 | .000 |
| EE\_5 | .021 | .021 | -.070 | .291 | .242 | .238 | 1.027 | .000 | .000 | .000 | 1.027 | .000 | .000 |
| EE\_4 | .021 | .021 | -.070 | .290 | .241 | .237 | 1.022 | .000 | .000 | .000 | 1.022 | .000 | .000 |
| EE\_3 | .021 | .022 | -.072 | .297 | .247 | .243 | 1.049 | .000 | .000 | .000 | 1.049 | .000 | .000 |
| EE\_2 | .021 | .021 | -.070 | .289 | .241 | .237 | 1.020 | .000 | .000 | .000 | 1.020 | .000 | .000 |
| EE\_1 | .020 | .021 | -.068 | .283 | .236 | .232 | 1.000 | .000 | .000 | .000 | 1.000 | .000 | .000 |
| ESE\_10 | .013 | .005 | -.053 | .327 | .250 | .974 | .000 | .000 | .000 | .000 | .000 | .981 | .000 |
| ESE\_9 | .013 | .004 | -.051 | .315 | .241 | .940 | .000 | .000 | .000 | .000 | .000 | .947 | .000 |
| ESE\_8 | .014 | .005 | -.054 | .335 | .256 | .998 | .000 | .000 | .000 | .000 | .000 | 1.005 | .000 |
| ESE\_7 | .014 | .005 | -.054 | .333 | .255 | .993 | .000 | .000 | .000 | .000 | .000 | 1.000 | .000 |
| ESE\_6 | .014 | .005 | -.056 | .345 | .264 | 1.028 | .000 | .000 | .000 | .000 | .000 | .000 | 1.028 |
| ESE\_5 | .014 | .005 | -.056 | .347 | .265 | 1.033 | .000 | .000 | .000 | .000 | .000 | .000 | 1.033 |
| ESE\_4 | .014 | .005 | -.057 | .351 | .269 | 1.047 | .000 | .000 | .000 | .000 | .000 | .000 | 1.047 |
| ESE\_3 | .013 | .005 | -.053 | .330 | .252 | .982 | .000 | .000 | .000 | .000 | .000 | .000 | .982 |
| ESE\_2 | .014 | .005 | -.056 | .345 | .264 | 1.028 | .000 | .000 | .000 | .000 | .000 | .000 | 1.028 |
| ESE\_1 | .014 | .005 | -.054 | .336 | .257 | 1.000 | .000 | .000 | .000 | .000 | .000 | .000 | 1.000 |
| EOE\_9 | .016 | .020 | -.037 | .364 | 1.001 | .000 | .000 | .000 | .000 | .000 | .000 | .000 | .000 |
| EOE\_8 | .017 | .020 | -.038 | .372 | 1.024 | .000 | .000 | .000 | .000 | .000 | .000 | .000 | .000 |
| EOE\_7 | .017 | .020 | -.038 | .374 | 1.030 | .000 | .000 | .000 | .000 | .000 | .000 | .000 | .000 |
| EOE\_6 | .016 | .020 | -.037 | .360 | .992 | .000 | .000 | .000 | .000 | .000 | .000 | .000 | .000 |
| EOE\_5 | .017 | .020 | -.038 | .375 | 1.032 | .000 | .000 | .000 | .000 | .000 | .000 | .000 | .000 |
| EOE\_4 | .016 | .020 | -.037 | .361 | .993 | .000 | .000 | .000 | .000 | .000 | .000 | .000 | .000 |
| EOE\_3 | .016 | .020 | -.037 | .364 | 1.002 | .000 | .000 | .000 | .000 | .000 | .000 | .000 | .000 |
| EOE\_2 | .017 | .020 | -.038 | .368 | 1.014 | .000 | .000 | .000 | .000 | .000 | .000 | .000 | .000 |
| EOE\_1 | .016 | .020 | -.037 | .363 | 1.000 | .000 | .000 | .000 | .000 | .000 | .000 | .000 | .000 |
| AISE\_6 | .000 | .000 | .000 | .999 | .000 | .000 | .000 | .000 | .000 | .000 | .000 | .000 | .000 |
| AISE\_5 | .000 | .000 | .000 | .955 | .000 | .000 | .000 | .000 | .000 | .000 | .000 | .000 | .000 |
| AISE\_4 | .000 | .000 | .000 | .991 | .000 | .000 | .000 | .000 | .000 | .000 | .000 | .000 | .000 |
| AISE\_3 | .000 | .000 | .000 | .959 | .000 | .000 | .000 | .000 | .000 | .000 | .000 | .000 | .000 |
| AISE\_2 | .000 | .000 | .000 | .983 | .000 | .000 | .000 | .000 | .000 | .000 | .000 | .000 | .000 |
| AISE\_1 | .000 | .000 | .000 | 1.000 | .000 | .000 | .000 | .000 | .000 | .000 | .000 | .000 | .000 |

## Standardized Total Effects (Group number 1 - Default model)

|  | Grade | Age | Gender | AISE | EOE | ESE | EE | Pa | Vc | Fs | Vp | Ss | Sm |
| --- | --- | --- | --- | --- | --- | --- | --- | --- | --- | --- | --- | --- | --- |
| EOE | .027 | .034 | -.035 | .547 | .000 | .000 | .000 | .000 | .000 | .000 | .000 | .000 | .000 |
| ESE | .026 | .010 | -.060 | .593 | .301 | .000 | .000 | .000 | .000 | .000 | .000 | .000 | .000 |
| EE | .039 | .041 | -.075 | .499 | .275 | .231 | .000 | .000 | .000 | .000 | .000 | .000 | .000 |
| Pa | .031 | .033 | -.060 | .401 | .221 | .186 | .803 | .000 | .000 | .000 | .000 | .000 | .000 |
| Vc | .032 | .034 | -.062 | .411 | .227 | .190 | .824 | .000 | .000 | .000 | .000 | .000 | .000 |
| Fs | .032 | .034 | -.061 | .406 | .224 | .188 | .813 | .000 | .000 | .000 | .000 | .000 | .000 |
| Vp | .031 | .033 | -.060 | .403 | .222 | .187 | .807 | .000 | .000 | .000 | .000 | .000 | .000 |
| Ss | .021 | .008 | -.047 | .465 | .236 | .784 | .000 | .000 | .000 | .000 | .000 | .000 | .000 |
| Sm | .021 | .008 | -.049 | .481 | .244 | .811 | .000 | .000 | .000 | .000 | .000 | .000 | .000 |
| EE\_20 | .023 | .024 | -.043 | .290 | .160 | .134 | .581 | .723 | .000 | .000 | .000 | .000 | .000 |
| EE\_19 | .022 | .024 | -.043 | .286 | .158 | .133 | .574 | .715 | .000 | .000 | .000 | .000 | .000 |
| EE\_18 | .023 | .025 | -.044 | .296 | .163 | .137 | .593 | .738 | .000 | .000 | .000 | .000 | .000 |
| EE\_17 | .024 | .025 | -.045 | .303 | .167 | .141 | .608 | .000 | .738 | .000 | .000 | .000 | .000 |
| EE\_16 | .023 | .025 | -.045 | .302 | .167 | .140 | .606 | .000 | .735 | .000 | .000 | .000 | .000 |
| EE\_15 | .024 | .026 | -.046 | .308 | .170 | .143 | .617 | .000 | .749 | .000 | .000 | .000 | .000 |
| EE\_14 | .023 | .025 | -.045 | .302 | .167 | .140 | .605 | .000 | .734 | .000 | .000 | .000 | .000 |
| EE\_13 | .024 | .026 | -.046 | .308 | .170 | .143 | .618 | .000 | .750 | .000 | .000 | .000 | .000 |
| EE\_12 | .023 | .025 | -.045 | .298 | .165 | .138 | .598 | .000 | .000 | .735 | .000 | .000 | .000 |
| EE\_11 | .023 | .025 | -.045 | .299 | .165 | .138 | .598 | .000 | .000 | .736 | .000 | .000 | .000 |
| EE\_10 | .023 | .025 | -.045 | .299 | .165 | .138 | .599 | .000 | .000 | .737 | .000 | .000 | .000 |
| EE\_9 | .023 | .025 | -.045 | .300 | .166 | .139 | .601 | .000 | .000 | .739 | .000 | .000 | .000 |
| EE\_8 | .023 | .024 | -.043 | .290 | .160 | .134 | .581 | .000 | .000 | .715 | .000 | .000 | .000 |
| EE\_7 | .023 | .025 | -.044 | .296 | .163 | .137 | .593 | .000 | .000 | .729 | .000 | .000 | .000 |
| EE\_6 | .023 | .025 | -.045 | .298 | .165 | .138 | .597 | .000 | .000 | .000 | .740 | .000 | .000 |
| EE\_5 | .023 | .024 | -.044 | .292 | .161 | .135 | .586 | .000 | .000 | .000 | .726 | .000 | .000 |
| EE\_4 | .023 | .024 | -.044 | .295 | .163 | .137 | .591 | .000 | .000 | .000 | .732 | .000 | .000 |
| EE\_3 | .023 | .025 | -.045 | .299 | .165 | .139 | .600 | .000 | .000 | .000 | .744 | .000 | .000 |
| EE\_2 | .023 | .024 | -.044 | .291 | .160 | .135 | .583 | .000 | .000 | .000 | .722 | .000 | .000 |
| EE\_1 | .022 | .024 | -.043 | .290 | .160 | .134 | .581 | .000 | .000 | .000 | .719 | .000 | .000 |
| ESE\_10 | .015 | .006 | -.035 | .342 | .173 | .576 | .000 | .000 | .000 | .000 | .000 | .735 | .000 |
| ESE\_9 | .015 | .005 | -.034 | .333 | .169 | .562 | .000 | .000 | .000 | .000 | .000 | .716 | .000 |
| ESE\_8 | .015 | .006 | -.035 | .348 | .177 | .586 | .000 | .000 | .000 | .000 | .000 | .748 | .000 |
| ESE\_7 | .015 | .006 | -.035 | .346 | .176 | .584 | .000 | .000 | .000 | .000 | .000 | .744 | .000 |
| ESE\_6 | .015 | .006 | -.035 | .347 | .176 | .585 | .000 | .000 | .000 | .000 | .000 | .000 | .721 |
| ESE\_5 | .016 | .006 | -.036 | .356 | .181 | .600 | .000 | .000 | .000 | .000 | .000 | .000 | .739 |
| ESE\_4 | .016 | .006 | -.036 | .356 | .181 | .600 | .000 | .000 | .000 | .000 | .000 | .000 | .739 |
| ESE\_3 | .015 | .006 | -.035 | .343 | .174 | .579 | .000 | .000 | .000 | .000 | .000 | .000 | .713 |
| ESE\_2 | .016 | .006 | -.036 | .353 | .179 | .595 | .000 | .000 | .000 | .000 | .000 | .000 | .733 |
| ESE\_1 | .015 | .006 | -.035 | .342 | .174 | .577 | .000 | .000 | .000 | .000 | .000 | .000 | .711 |
| EOE\_9 | .019 | .024 | -.025 | .393 | .718 | .000 | .000 | .000 | .000 | .000 | .000 | .000 | .000 |
| EOE\_8 | .020 | .025 | -.025 | .398 | .728 | .000 | .000 | .000 | .000 | .000 | .000 | .000 | .000 |
| EOE\_7 | .020 | .025 | -.026 | .408 | .745 | .000 | .000 | .000 | .000 | .000 | .000 | .000 | .000 |
| EOE\_6 | .019 | .024 | -.025 | .392 | .716 | .000 | .000 | .000 | .000 | .000 | .000 | .000 | .000 |
| EOE\_5 | .020 | .025 | -.026 | .404 | .738 | .000 | .000 | .000 | .000 | .000 | .000 | .000 | .000 |
| EOE\_4 | .019 | .025 | -.025 | .396 | .724 | .000 | .000 | .000 | .000 | .000 | .000 | .000 | .000 |
| EOE\_3 | .019 | .025 | -.025 | .394 | .721 | .000 | .000 | .000 | .000 | .000 | .000 | .000 | .000 |
| EOE\_2 | .020 | .025 | -.025 | .398 | .727 | .000 | .000 | .000 | .000 | .000 | .000 | .000 | .000 |
| EOE\_1 | .019 | .024 | -.025 | .394 | .720 | .000 | .000 | .000 | .000 | .000 | .000 | .000 | .000 |
| AISE\_6 | .000 | .000 | .000 | .752 | .000 | .000 | .000 | .000 | .000 | .000 | .000 | .000 | .000 |
| AISE\_5 | .000 | .000 | .000 | .760 | .000 | .000 | .000 | .000 | .000 | .000 | .000 | .000 | .000 |
| AISE\_4 | .000 | .000 | .000 | .748 | .000 | .000 | .000 | .000 | .000 | .000 | .000 | .000 | .000 |
| AISE\_3 | .000 | .000 | .000 | .736 | .000 | .000 | .000 | .000 | .000 | .000 | .000 | .000 | .000 |
| AISE\_2 | .000 | .000 | .000 | .757 | .000 | .000 | .000 | .000 | .000 | .000 | .000 | .000 | .000 |
| AISE\_1 | .000 | .000 | .000 | .721 | .000 | .000 | .000 | .000 | .000 | .000 | .000 | .000 | .000 |

## Direct Effects (Group number 1 - Default model)

|  | Grade | Age | Gender | AISE | EOE | ESE | EE | Pa | Vc | Fs | Vp | Ss | Sm |
| --- | --- | --- | --- | --- | --- | --- | --- | --- | --- | --- | --- | --- | --- |
| EOE | .016 | .020 | -.037 | .363 | .000 | .000 | .000 | .000 | .000 | .000 | .000 | .000 | .000 |
| ESE | .009 | .000 | -.045 | .242 | .257 | .000 | .000 | .000 | .000 | .000 | .000 | .000 | .000 |
| EE | .014 | .016 | -.049 | .142 | .176 | .232 | .000 | .000 | .000 | .000 | .000 | .000 | .000 |
| Pa | .000 | .000 | .000 | .000 | .000 | .000 | .978 | .000 | .000 | .000 | .000 | .000 | .000 |
| Vc | .000 | .000 | .000 | .000 | .000 | .000 | 1.062 | .000 | .000 | .000 | .000 | .000 | .000 |
| Fs | .000 | .000 | .000 | .000 | .000 | .000 | 1.034 | .000 | .000 | .000 | .000 | .000 | .000 |
| Vp | .000 | .000 | .000 | .000 | .000 | .000 | 1.000 | .000 | .000 | .000 | .000 | .000 | .000 |
| Ss | .000 | .000 | .000 | .000 | .000 | .993 | .000 | .000 | .000 | .000 | .000 | .000 | .000 |
| Sm | .000 | .000 | .000 | .000 | .000 | 1.000 | .000 | .000 | .000 | .000 | .000 | .000 | .000 |
| EE\_20 | .000 | .000 | .000 | .000 | .000 | .000 | .000 | .990 | .000 | .000 | .000 | .000 | .000 |
| EE\_19 | .000 | .000 | .000 | .000 | .000 | .000 | .000 | .973 | .000 | .000 | .000 | .000 | .000 |
| EE\_18 | .000 | .000 | .000 | .000 | .000 | .000 | .000 | 1.000 | .000 | .000 | .000 | .000 | .000 |
| EE\_17 | .000 | .000 | .000 | .000 | .000 | .000 | .000 | .000 | .970 | .000 | .000 | .000 | .000 |
| EE\_16 | .000 | .000 | .000 | .000 | .000 | .000 | .000 | .000 | .977 | .000 | .000 | .000 | .000 |
| EE\_15 | .000 | .000 | .000 | .000 | .000 | .000 | .000 | .000 | .989 | .000 | .000 | .000 | .000 |
| EE\_14 | .000 | .000 | .000 | .000 | .000 | .000 | .000 | .000 | .973 | .000 | .000 | .000 | .000 |
| EE\_13 | .000 | .000 | .000 | .000 | .000 | .000 | .000 | .000 | 1.000 | .000 | .000 | .000 | .000 |
| EE\_12 | .000 | .000 | .000 | .000 | .000 | .000 | .000 | .000 | .000 | .994 | .000 | .000 | .000 |
| EE\_11 | .000 | .000 | .000 | .000 | .000 | .000 | .000 | .000 | .000 | 1.003 | .000 | .000 | .000 |
| EE\_10 | .000 | .000 | .000 | .000 | .000 | .000 | .000 | .000 | .000 | 1.008 | .000 | .000 | .000 |
| EE\_9 | .000 | .000 | .000 | .000 | .000 | .000 | .000 | .000 | .000 | 1.007 | .000 | .000 | .000 |
| EE\_8 | .000 | .000 | .000 | .000 | .000 | .000 | .000 | .000 | .000 | .972 | .000 | .000 | .000 |
| EE\_7 | .000 | .000 | .000 | .000 | .000 | .000 | .000 | .000 | .000 | 1.000 | .000 | .000 | .000 |
| EE\_6 | .000 | .000 | .000 | .000 | .000 | .000 | .000 | .000 | .000 | .000 | 1.037 | .000 | .000 |
| EE\_5 | .000 | .000 | .000 | .000 | .000 | .000 | .000 | .000 | .000 | .000 | 1.027 | .000 | .000 |
| EE\_4 | .000 | .000 | .000 | .000 | .000 | .000 | .000 | .000 | .000 | .000 | 1.022 | .000 | .000 |
| EE\_3 | .000 | .000 | .000 | .000 | .000 | .000 | .000 | .000 | .000 | .000 | 1.049 | .000 | .000 |
| EE\_2 | .000 | .000 | .000 | .000 | .000 | .000 | .000 | .000 | .000 | .000 | 1.020 | .000 | .000 |
| EE\_1 | .000 | .000 | .000 | .000 | .000 | .000 | .000 | .000 | .000 | .000 | 1.000 | .000 | .000 |
| ESE\_10 | .000 | .000 | .000 | .000 | .000 | .000 | .000 | .000 | .000 | .000 | .000 | .981 | .000 |
| ESE\_9 | .000 | .000 | .000 | .000 | .000 | .000 | .000 | .000 | .000 | .000 | .000 | .947 | .000 |
| ESE\_8 | .000 | .000 | .000 | .000 | .000 | .000 | .000 | .000 | .000 | .000 | .000 | 1.005 | .000 |
| ESE\_7 | .000 | .000 | .000 | .000 | .000 | .000 | .000 | .000 | .000 | .000 | .000 | 1.000 | .000 |
| ESE\_6 | .000 | .000 | .000 | .000 | .000 | .000 | .000 | .000 | .000 | .000 | .000 | .000 | 1.028 |
| ESE\_5 | .000 | .000 | .000 | .000 | .000 | .000 | .000 | .000 | .000 | .000 | .000 | .000 | 1.033 |
| ESE\_4 | .000 | .000 | .000 | .000 | .000 | .000 | .000 | .000 | .000 | .000 | .000 | .000 | 1.047 |
| ESE\_3 | .000 | .000 | .000 | .000 | .000 | .000 | .000 | .000 | .000 | .000 | .000 | .000 | .982 |
| ESE\_2 | .000 | .000 | .000 | .000 | .000 | .000 | .000 | .000 | .000 | .000 | .000 | .000 | 1.028 |
| ESE\_1 | .000 | .000 | .000 | .000 | .000 | .000 | .000 | .000 | .000 | .000 | .000 | .000 | 1.000 |
| EOE\_9 | .000 | .000 | .000 | .000 | 1.001 | .000 | .000 | .000 | .000 | .000 | .000 | .000 | .000 |
| EOE\_8 | .000 | .000 | .000 | .000 | 1.024 | .000 | .000 | .000 | .000 | .000 | .000 | .000 | .000 |
| EOE\_7 | .000 | .000 | .000 | .000 | 1.030 | .000 | .000 | .000 | .000 | .000 | .000 | .000 | .000 |
| EOE\_6 | .000 | .000 | .000 | .000 | .992 | .000 | .000 | .000 | .000 | .000 | .000 | .000 | .000 |
| EOE\_5 | .000 | .000 | .000 | .000 | 1.032 | .000 | .000 | .000 | .000 | .000 | .000 | .000 | .000 |
| EOE\_4 | .000 | .000 | .000 | .000 | .993 | .000 | .000 | .000 | .000 | .000 | .000 | .000 | .000 |
| EOE\_3 | .000 | .000 | .000 | .000 | 1.002 | .000 | .000 | .000 | .000 | .000 | .000 | .000 | .000 |
| EOE\_2 | .000 | .000 | .000 | .000 | 1.014 | .000 | .000 | .000 | .000 | .000 | .000 | .000 | .000 |
| EOE\_1 | .000 | .000 | .000 | .000 | 1.000 | .000 | .000 | .000 | .000 | .000 | .000 | .000 | .000 |
| AISE\_6 | .000 | .000 | .000 | .999 | .000 | .000 | .000 | .000 | .000 | .000 | .000 | .000 | .000 |
| AISE\_5 | .000 | .000 | .000 | .955 | .000 | .000 | .000 | .000 | .000 | .000 | .000 | .000 | .000 |
| AISE\_4 | .000 | .000 | .000 | .991 | .000 | .000 | .000 | .000 | .000 | .000 | .000 | .000 | .000 |
| AISE\_3 | .000 | .000 | .000 | .959 | .000 | .000 | .000 | .000 | .000 | .000 | .000 | .000 | .000 |
| AISE\_2 | .000 | .000 | .000 | .983 | .000 | .000 | .000 | .000 | .000 | .000 | .000 | .000 | .000 |
| AISE\_1 | .000 | .000 | .000 | 1.000 | .000 | .000 | .000 | .000 | .000 | .000 | .000 | .000 | .000 |

## Standardized Direct Effects (Group number 1 - Default model)

|  | Grade | Age | Gender | AISE | EOE | ESE | EE | Pa | Vc | Fs | Vp | Ss | Sm |
| --- | --- | --- | --- | --- | --- | --- | --- | --- | --- | --- | --- | --- | --- |
| EOE | .027 | .034 | -.035 | .547 | .000 | .000 | .000 | .000 | .000 | .000 | .000 | .000 | .000 |
| ESE | .018 | -.001 | -.049 | .428 | .301 | .000 | .000 | .000 | .000 | .000 | .000 | .000 | .000 |
| EE | .027 | .032 | -.054 | .249 | .206 | .231 | .000 | .000 | .000 | .000 | .000 | .000 | .000 |
| Pa | .000 | .000 | .000 | .000 | .000 | .000 | .803 | .000 | .000 | .000 | .000 | .000 | .000 |
| Vc | .000 | .000 | .000 | .000 | .000 | .000 | .824 | .000 | .000 | .000 | .000 | .000 | .000 |
| Fs | .000 | .000 | .000 | .000 | .000 | .000 | .813 | .000 | .000 | .000 | .000 | .000 | .000 |
| Vp | .000 | .000 | .000 | .000 | .000 | .000 | .807 | .000 | .000 | .000 | .000 | .000 | .000 |
| Ss | .000 | .000 | .000 | .000 | .000 | .784 | .000 | .000 | .000 | .000 | .000 | .000 | .000 |
| Sm | .000 | .000 | .000 | .000 | .000 | .811 | .000 | .000 | .000 | .000 | .000 | .000 | .000 |
| EE\_20 | .000 | .000 | .000 | .000 | .000 | .000 | .000 | .723 | .000 | .000 | .000 | .000 | .000 |
| EE\_19 | .000 | .000 | .000 | .000 | .000 | .000 | .000 | .715 | .000 | .000 | .000 | .000 | .000 |
| EE\_18 | .000 | .000 | .000 | .000 | .000 | .000 | .000 | .738 | .000 | .000 | .000 | .000 | .000 |
| EE\_17 | .000 | .000 | .000 | .000 | .000 | .000 | .000 | .000 | .738 | .000 | .000 | .000 | .000 |
| EE\_16 | .000 | .000 | .000 | .000 | .000 | .000 | .000 | .000 | .735 | .000 | .000 | .000 | .000 |
| EE\_15 | .000 | .000 | .000 | .000 | .000 | .000 | .000 | .000 | .749 | .000 | .000 | .000 | .000 |
| EE\_14 | .000 | .000 | .000 | .000 | .000 | .000 | .000 | .000 | .734 | .000 | .000 | .000 | .000 |
| EE\_13 | .000 | .000 | .000 | .000 | .000 | .000 | .000 | .000 | .750 | .000 | .000 | .000 | .000 |
| EE\_12 | .000 | .000 | .000 | .000 | .000 | .000 | .000 | .000 | .000 | .735 | .000 | .000 | .000 |
| EE\_11 | .000 | .000 | .000 | .000 | .000 | .000 | .000 | .000 | .000 | .736 | .000 | .000 | .000 |
| EE\_10 | .000 | .000 | .000 | .000 | .000 | .000 | .000 | .000 | .000 | .737 | .000 | .000 | .000 |
| EE\_9 | .000 | .000 | .000 | .000 | .000 | .000 | .000 | .000 | .000 | .739 | .000 | .000 | .000 |
| EE\_8 | .000 | .000 | .000 | .000 | .000 | .000 | .000 | .000 | .000 | .715 | .000 | .000 | .000 |
| EE\_7 | .000 | .000 | .000 | .000 | .000 | .000 | .000 | .000 | .000 | .729 | .000 | .000 | .000 |
| EE\_6 | .000 | .000 | .000 | .000 | .000 | .000 | .000 | .000 | .000 | .000 | .740 | .000 | .000 |
| EE\_5 | .000 | .000 | .000 | .000 | .000 | .000 | .000 | .000 | .000 | .000 | .726 | .000 | .000 |
| EE\_4 | .000 | .000 | .000 | .000 | .000 | .000 | .000 | .000 | .000 | .000 | .732 | .000 | .000 |
| EE\_3 | .000 | .000 | .000 | .000 | .000 | .000 | .000 | .000 | .000 | .000 | .744 | .000 | .000 |
| EE\_2 | .000 | .000 | .000 | .000 | .000 | .000 | .000 | .000 | .000 | .000 | .722 | .000 | .000 |
| EE\_1 | .000 | .000 | .000 | .000 | .000 | .000 | .000 | .000 | .000 | .000 | .719 | .000 | .000 |
| ESE\_10 | .000 | .000 | .000 | .000 | .000 | .000 | .000 | .000 | .000 | .000 | .000 | .735 | .000 |
| ESE\_9 | .000 | .000 | .000 | .000 | .000 | .000 | .000 | .000 | .000 | .000 | .000 | .716 | .000 |
| ESE\_8 | .000 | .000 | .000 | .000 | .000 | .000 | .000 | .000 | .000 | .000 | .000 | .748 | .000 |
| ESE\_7 | .000 | .000 | .000 | .000 | .000 | .000 | .000 | .000 | .000 | .000 | .000 | .744 | .000 |
| ESE\_6 | .000 | .000 | .000 | .000 | .000 | .000 | .000 | .000 | .000 | .000 | .000 | .000 | .721 |
| ESE\_5 | .000 | .000 | .000 | .000 | .000 | .000 | .000 | .000 | .000 | .000 | .000 | .000 | .739 |
| ESE\_4 | .000 | .000 | .000 | .000 | .000 | .000 | .000 | .000 | .000 | .000 | .000 | .000 | .739 |
| ESE\_3 | .000 | .000 | .000 | .000 | .000 | .000 | .000 | .000 | .000 | .000 | .000 | .000 | .713 |
| ESE\_2 | .000 | .000 | .000 | .000 | .000 | .000 | .000 | .000 | .000 | .000 | .000 | .000 | .733 |
| ESE\_1 | .000 | .000 | .000 | .000 | .000 | .000 | .000 | .000 | .000 | .000 | .000 | .000 | .711 |
| EOE\_9 | .000 | .000 | .000 | .000 | .718 | .000 | .000 | .000 | .000 | .000 | .000 | .000 | .000 |
| EOE\_8 | .000 | .000 | .000 | .000 | .728 | .000 | .000 | .000 | .000 | .000 | .000 | .000 | .000 |
| EOE\_7 | .000 | .000 | .000 | .000 | .745 | .000 | .000 | .000 | .000 | .000 | .000 | .000 | .000 |
| EOE\_6 | .000 | .000 | .000 | .000 | .716 | .000 | .000 | .000 | .000 | .000 | .000 | .000 | .000 |
| EOE\_5 | .000 | .000 | .000 | .000 | .738 | .000 | .000 | .000 | .000 | .000 | .000 | .000 | .000 |
| EOE\_4 | .000 | .000 | .000 | .000 | .724 | .000 | .000 | .000 | .000 | .000 | .000 | .000 | .000 |
| EOE\_3 | .000 | .000 | .000 | .000 | .721 | .000 | .000 | .000 | .000 | .000 | .000 | .000 | .000 |
| EOE\_2 | .000 | .000 | .000 | .000 | .727 | .000 | .000 | .000 | .000 | .000 | .000 | .000 | .000 |
| EOE\_1 | .000 | .000 | .000 | .000 | .720 | .000 | .000 | .000 | .000 | .000 | .000 | .000 | .000 |
| AISE\_6 | .000 | .000 | .000 | .752 | .000 | .000 | .000 | .000 | .000 | .000 | .000 | .000 | .000 |
| AISE\_5 | .000 | .000 | .000 | .760 | .000 | .000 | .000 | .000 | .000 | .000 | .000 | .000 | .000 |
| AISE\_4 | .000 | .000 | .000 | .748 | .000 | .000 | .000 | .000 | .000 | .000 | .000 | .000 | .000 |
| AISE\_3 | .000 | .000 | .000 | .736 | .000 | .000 | .000 | .000 | .000 | .000 | .000 | .000 | .000 |
| AISE\_2 | .000 | .000 | .000 | .757 | .000 | .000 | .000 | .000 | .000 | .000 | .000 | .000 | .000 |
| AISE\_1 | .000 | .000 | .000 | .721 | .000 | .000 | .000 | .000 | .000 | .000 | .000 | .000 | .000 |

## Indirect Effects (Group number 1 - Default model)

|  | Grade | Age | Gender | AISE | EOE | ESE | EE | Pa | Vc | Fs | Vp | Ss | Sm |
| --- | --- | --- | --- | --- | --- | --- | --- | --- | --- | --- | --- | --- | --- |
| EOE | .000 | .000 | .000 | .000 | .000 | .000 | .000 | .000 | .000 | .000 | .000 | .000 | .000 |
| ESE | .004 | .005 | -.010 | .093 | .000 | .000 | .000 | .000 | .000 | .000 | .000 | .000 | .000 |
| EE | .006 | .005 | -.019 | .142 | .060 | .000 | .000 | .000 | .000 | .000 | .000 | .000 | .000 |
| Pa | .020 | .020 | -.067 | .277 | .231 | .227 | .000 | .000 | .000 | .000 | .000 | .000 | .000 |
| Vc | .021 | .022 | -.072 | .301 | .250 | .246 | .000 | .000 | .000 | .000 | .000 | .000 | .000 |
| Fs | .021 | .021 | -.070 | .293 | .244 | .240 | .000 | .000 | .000 | .000 | .000 | .000 | .000 |
| Vp | .020 | .021 | -.068 | .283 | .236 | .232 | .000 | .000 | .000 | .000 | .000 | .000 | .000 |
| Ss | .014 | .005 | -.054 | .333 | .255 | .000 | .000 | .000 | .000 | .000 | .000 | .000 | .000 |
| Sm | .014 | .005 | -.054 | .336 | .257 | .000 | .000 | .000 | .000 | .000 | .000 | .000 | .000 |
| EE\_20 | .020 | .020 | -.066 | .274 | .228 | .225 | .968 | .000 | .000 | .000 | .000 | .000 | .000 |
| EE\_19 | .019 | .020 | -.065 | .270 | .224 | .221 | .952 | .000 | .000 | .000 | .000 | .000 | .000 |
| EE\_18 | .020 | .020 | -.067 | .277 | .231 | .227 | .978 | .000 | .000 | .000 | .000 | .000 | .000 |
| EE\_17 | .021 | .021 | -.070 | .292 | .243 | .239 | 1.029 | .000 | .000 | .000 | .000 | .000 | .000 |
| EE\_16 | .021 | .021 | -.071 | .294 | .245 | .241 | 1.037 | .000 | .000 | .000 | .000 | .000 | .000 |
| EE\_15 | .021 | .022 | -.072 | .298 | .248 | .244 | 1.050 | .000 | .000 | .000 | .000 | .000 | .000 |
| EE\_14 | .021 | .021 | -.070 | .293 | .244 | .240 | 1.033 | .000 | .000 | .000 | .000 | .000 | .000 |
| EE\_13 | .021 | .022 | -.072 | .301 | .250 | .246 | 1.062 | .000 | .000 | .000 | .000 | .000 | .000 |
| EE\_12 | .021 | .021 | -.070 | .291 | .242 | .238 | 1.027 | .000 | .000 | .000 | .000 | .000 | .000 |
| EE\_11 | .021 | .021 | -.071 | .294 | .245 | .241 | 1.037 | .000 | .000 | .000 | .000 | .000 | .000 |
| EE\_10 | .021 | .021 | -.071 | .295 | .246 | .242 | 1.042 | .000 | .000 | .000 | .000 | .000 | .000 |
| EE\_9 | .021 | .021 | -.071 | .295 | .246 | .242 | 1.041 | .000 | .000 | .000 | .000 | .000 | .000 |
| EE\_8 | .020 | .021 | -.068 | .285 | .237 | .233 | 1.005 | .000 | .000 | .000 | .000 | .000 | .000 |
| EE\_7 | .021 | .021 | -.070 | .293 | .244 | .240 | 1.034 | .000 | .000 | .000 | .000 | .000 | .000 |
| EE\_6 | .021 | .021 | -.071 | .294 | .245 | .241 | 1.037 | .000 | .000 | .000 | .000 | .000 | .000 |
| EE\_5 | .021 | .021 | -.070 | .291 | .242 | .238 | 1.027 | .000 | .000 | .000 | .000 | .000 | .000 |
| EE\_4 | .021 | .021 | -.070 | .290 | .241 | .237 | 1.022 | .000 | .000 | .000 | .000 | .000 | .000 |
| EE\_3 | .021 | .022 | -.072 | .297 | .247 | .243 | 1.049 | .000 | .000 | .000 | .000 | .000 | .000 |
| EE\_2 | .021 | .021 | -.070 | .289 | .241 | .237 | 1.020 | .000 | .000 | .000 | .000 | .000 | .000 |
| EE\_1 | .020 | .021 | -.068 | .283 | .236 | .232 | 1.000 | .000 | .000 | .000 | .000 | .000 | .000 |
| ESE\_10 | .013 | .005 | -.053 | .327 | .250 | .974 | .000 | .000 | .000 | .000 | .000 | .000 | .000 |
| ESE\_9 | .013 | .004 | -.051 | .315 | .241 | .940 | .000 | .000 | .000 | .000 | .000 | .000 | .000 |
| ESE\_8 | .014 | .005 | -.054 | .335 | .256 | .998 | .000 | .000 | .000 | .000 | .000 | .000 | .000 |
| ESE\_7 | .014 | .005 | -.054 | .333 | .255 | .993 | .000 | .000 | .000 | .000 | .000 | .000 | .000 |
| ESE\_6 | .014 | .005 | -.056 | .345 | .264 | 1.028 | .000 | .000 | .000 | .000 | .000 | .000 | .000 |
| ESE\_5 | .014 | .005 | -.056 | .347 | .265 | 1.033 | .000 | .000 | .000 | .000 | .000 | .000 | .000 |
| ESE\_4 | .014 | .005 | -.057 | .351 | .269 | 1.047 | .000 | .000 | .000 | .000 | .000 | .000 | .000 |
| ESE\_3 | .013 | .005 | -.053 | .330 | .252 | .982 | .000 | .000 | .000 | .000 | .000 | .000 | .000 |
| ESE\_2 | .014 | .005 | -.056 | .345 | .264 | 1.028 | .000 | .000 | .000 | .000 | .000 | .000 | .000 |
| ESE\_1 | .014 | .005 | -.054 | .336 | .257 | 1.000 | .000 | .000 | .000 | .000 | .000 | .000 | .000 |
| EOE\_9 | .016 | .020 | -.037 | .364 | .000 | .000 | .000 | .000 | .000 | .000 | .000 | .000 | .000 |
| EOE\_8 | .017 | .020 | -.038 | .372 | .000 | .000 | .000 | .000 | .000 | .000 | .000 | .000 | .000 |
| EOE\_7 | .017 | .020 | -.038 | .374 | .000 | .000 | .000 | .000 | .000 | .000 | .000 | .000 | .000 |
| EOE\_6 | .016 | .020 | -.037 | .360 | .000 | .000 | .000 | .000 | .000 | .000 | .000 | .000 | .000 |
| EOE\_5 | .017 | .020 | -.038 | .375 | .000 | .000 | .000 | .000 | .000 | .000 | .000 | .000 | .000 |
| EOE\_4 | .016 | .020 | -.037 | .361 | .000 | .000 | .000 | .000 | .000 | .000 | .000 | .000 | .000 |
| EOE\_3 | .016 | .020 | -.037 | .364 | .000 | .000 | .000 | .000 | .000 | .000 | .000 | .000 | .000 |
| EOE\_2 | .017 | .020 | -.038 | .368 | .000 | .000 | .000 | .000 | .000 | .000 | .000 | .000 | .000 |
| EOE\_1 | .016 | .020 | -.037 | .363 | .000 | .000 | .000 | .000 | .000 | .000 | .000 | .000 | .000 |
| AISE\_6 | .000 | .000 | .000 | .000 | .000 | .000 | .000 | .000 | .000 | .000 | .000 | .000 | .000 |
| AISE\_5 | .000 | .000 | .000 | .000 | .000 | .000 | .000 | .000 | .000 | .000 | .000 | .000 | .000 |
| AISE\_4 | .000 | .000 | .000 | .000 | .000 | .000 | .000 | .000 | .000 | .000 | .000 | .000 | .000 |
| AISE\_3 | .000 | .000 | .000 | .000 | .000 | .000 | .000 | .000 | .000 | .000 | .000 | .000 | .000 |
| AISE\_2 | .000 | .000 | .000 | .000 | .000 | .000 | .000 | .000 | .000 | .000 | .000 | .000 | .000 |
| AISE\_1 | .000 | .000 | .000 | .000 | .000 | .000 | .000 | .000 | .000 | .000 | .000 | .000 | .000 |

## Standardized Indirect Effects (Group number 1 - Default model)

|  | Grade | Age | Gender | AISE | EOE | ESE | EE | Pa | Vc | Fs | Vp | Ss | Sm |
| --- | --- | --- | --- | --- | --- | --- | --- | --- | --- | --- | --- | --- | --- |
| EOE | .000 | .000 | .000 | .000 | .000 | .000 | .000 | .000 | .000 | .000 | .000 | .000 | .000 |
| ESE | .008 | .010 | -.011 | .165 | .000 | .000 | .000 | .000 | .000 | .000 | .000 | .000 | .000 |
| EE | .012 | .009 | -.021 | .250 | .070 | .000 | .000 | .000 | .000 | .000 | .000 | .000 | .000 |
| Pa | .031 | .033 | -.060 | .401 | .221 | .186 | .000 | .000 | .000 | .000 | .000 | .000 | .000 |
| Vc | .032 | .034 | -.062 | .411 | .227 | .190 | .000 | .000 | .000 | .000 | .000 | .000 | .000 |
| Fs | .032 | .034 | -.061 | .406 | .224 | .188 | .000 | .000 | .000 | .000 | .000 | .000 | .000 |
| Vp | .031 | .033 | -.060 | .403 | .222 | .187 | .000 | .000 | .000 | .000 | .000 | .000 | .000 |
| Ss | .021 | .008 | -.047 | .465 | .236 | .000 | .000 | .000 | .000 | .000 | .000 | .000 | .000 |
| Sm | .021 | .008 | -.049 | .481 | .244 | .000 | .000 | .000 | .000 | .000 | .000 | .000 | .000 |
| EE\_20 | .023 | .024 | -.043 | .290 | .160 | .134 | .581 | .000 | .000 | .000 | .000 | .000 | .000 |
| EE\_19 | .022 | .024 | -.043 | .286 | .158 | .133 | .574 | .000 | .000 | .000 | .000 | .000 | .000 |
| EE\_18 | .023 | .025 | -.044 | .296 | .163 | .137 | .593 | .000 | .000 | .000 | .000 | .000 | .000 |
| EE\_17 | .024 | .025 | -.045 | .303 | .167 | .141 | .608 | .000 | .000 | .000 | .000 | .000 | .000 |
| EE\_16 | .023 | .025 | -.045 | .302 | .167 | .140 | .606 | .000 | .000 | .000 | .000 | .000 | .000 |
| EE\_15 | .024 | .026 | -.046 | .308 | .170 | .143 | .617 | .000 | .000 | .000 | .000 | .000 | .000 |
| EE\_14 | .023 | .025 | -.045 | .302 | .167 | .140 | .605 | .000 | .000 | .000 | .000 | .000 | .000 |
| EE\_13 | .024 | .026 | -.046 | .308 | .170 | .143 | .618 | .000 | .000 | .000 | .000 | .000 | .000 |
| EE\_12 | .023 | .025 | -.045 | .298 | .165 | .138 | .598 | .000 | .000 | .000 | .000 | .000 | .000 |
| EE\_11 | .023 | .025 | -.045 | .299 | .165 | .138 | .598 | .000 | .000 | .000 | .000 | .000 | .000 |
| EE\_10 | .023 | .025 | -.045 | .299 | .165 | .138 | .599 | .000 | .000 | .000 | .000 | .000 | .000 |
| EE\_9 | .023 | .025 | -.045 | .300 | .166 | .139 | .601 | .000 | .000 | .000 | .000 | .000 | .000 |
| EE\_8 | .023 | .024 | -.043 | .290 | .160 | .134 | .581 | .000 | .000 | .000 | .000 | .000 | .000 |
| EE\_7 | .023 | .025 | -.044 | .296 | .163 | .137 | .593 | .000 | .000 | .000 | .000 | .000 | .000 |
| EE\_6 | .023 | .025 | -.045 | .298 | .165 | .138 | .597 | .000 | .000 | .000 | .000 | .000 | .000 |
| EE\_5 | .023 | .024 | -.044 | .292 | .161 | .135 | .586 | .000 | .000 | .000 | .000 | .000 | .000 |
| EE\_4 | .023 | .024 | -.044 | .295 | .163 | .137 | .591 | .000 | .000 | .000 | .000 | .000 | .000 |
| EE\_3 | .023 | .025 | -.045 | .299 | .165 | .139 | .600 | .000 | .000 | .000 | .000 | .000 | .000 |
| EE\_2 | .023 | .024 | -.044 | .291 | .160 | .135 | .583 | .000 | .000 | .000 | .000 | .000 | .000 |
| EE\_1 | .022 | .024 | -.043 | .290 | .160 | .134 | .581 | .000 | .000 | .000 | .000 | .000 | .000 |
| ESE\_10 | .015 | .006 | -.035 | .342 | .173 | .576 | .000 | .000 | .000 | .000 | .000 | .000 | .000 |
| ESE\_9 | .015 | .005 | -.034 | .333 | .169 | .562 | .000 | .000 | .000 | .000 | .000 | .000 | .000 |
| ESE\_8 | .015 | .006 | -.035 | .348 | .177 | .586 | .000 | .000 | .000 | .000 | .000 | .000 | .000 |
| ESE\_7 | .015 | .006 | -.035 | .346 | .176 | .584 | .000 | .000 | .000 | .000 | .000 | .000 | .000 |
| ESE\_6 | .015 | .006 | -.035 | .347 | .176 | .585 | .000 | .000 | .000 | .000 | .000 | .000 | .000 |
| ESE\_5 | .016 | .006 | -.036 | .356 | .181 | .600 | .000 | .000 | .000 | .000 | .000 | .000 | .000 |
| ESE\_4 | .016 | .006 | -.036 | .356 | .181 | .600 | .000 | .000 | .000 | .000 | .000 | .000 | .000 |
| ESE\_3 | .015 | .006 | -.035 | .343 | .174 | .579 | .000 | .000 | .000 | .000 | .000 | .000 | .000 |
| ESE\_2 | .016 | .006 | -.036 | .353 | .179 | .595 | .000 | .000 | .000 | .000 | .000 | .000 | .000 |
| ESE\_1 | .015 | .006 | -.035 | .342 | .174 | .577 | .000 | .000 | .000 | .000 | .000 | .000 | .000 |
| EOE\_9 | .019 | .024 | -.025 | .393 | .000 | .000 | .000 | .000 | .000 | .000 | .000 | .000 | .000 |
| EOE\_8 | .020 | .025 | -.025 | .398 | .000 | .000 | .000 | .000 | .000 | .000 | .000 | .000 | .000 |
| EOE\_7 | .020 | .025 | -.026 | .408 | .000 | .000 | .000 | .000 | .000 | .000 | .000 | .000 | .000 |
| EOE\_6 | .019 | .024 | -.025 | .392 | .000 | .000 | .000 | .000 | .000 | .000 | .000 | .000 | .000 |
| EOE\_5 | .020 | .025 | -.026 | .404 | .000 | .000 | .000 | .000 | .000 | .000 | .000 | .000 | .000 |
| EOE\_4 | .019 | .025 | -.025 | .396 | .000 | .000 | .000 | .000 | .000 | .000 | .000 | .000 | .000 |
| EOE\_3 | .019 | .025 | -.025 | .394 | .000 | .000 | .000 | .000 | .000 | .000 | .000 | .000 | .000 |
| EOE\_2 | .020 | .025 | -.025 | .398 | .000 | .000 | .000 | .000 | .000 | .000 | .000 | .000 | .000 |
| EOE\_1 | .019 | .024 | -.025 | .394 | .000 | .000 | .000 | .000 | .000 | .000 | .000 | .000 | .000 |
| AISE\_6 | .000 | .000 | .000 | .000 | .000 | .000 | .000 | .000 | .000 | .000 | .000 | .000 | .000 |
| AISE\_5 | .000 | .000 | .000 | .000 | .000 | .000 | .000 | .000 | .000 | .000 | .000 | .000 | .000 |
| AISE\_4 | .000 | .000 | .000 | .000 | .000 | .000 | .000 | .000 | .000 | .000 | .000 | .000 | .000 |
| AISE\_3 | .000 | .000 | .000 | .000 | .000 | .000 | .000 | .000 | .000 | .000 | .000 | .000 | .000 |
| AISE\_2 | .000 | .000 | .000 | .000 | .000 | .000 | .000 | .000 | .000 | .000 | .000 | .000 | .000 |
| AISE\_1 | .000 | .000 | .000 | .000 | .000 | .000 | .000 | .000 | .000 | .000 | .000 | .000 | .000 |

## Modification Indices (Group number 1 - Default model)

## Covariances: (Group number 1 - Default model)

|  |  |  | M.I. | Par Change |
| --- | --- | --- | --- | --- |
| e51 | <--> | Gender | 5.690 | -.012 |
| e44 | <--> | e52 | 4.498 | .012 |
| e41 | <--> | AISE | 4.711 | -.021 |
| e40 | <--> | e46 | 6.089 | -.013 |
| e40 | <--> | e44 | 5.848 | .016 |
| e39 | <--> | Age | 4.353 | -.022 |
| e39 | <--> | AISE | 4.185 | .020 |
| e39 | <--> | e51 | 5.047 | -.013 |
| e38 | <--> | Age | 4.820 | .023 |
| e38 | <--> | e46 | 4.656 | .012 |
| e38 | <--> | e42 | 4.830 | -.014 |
| e37 | <--> | Age | 5.145 | .024 |
| e36 | <--> | e46 | 4.005 | -.011 |
| e33 | <--> | e41 | 4.726 | .015 |
| e31 | <--> | Grade | 6.426 | -.026 |
| e31 | <--> | Gender | 5.829 | -.014 |
| e31 | <--> | e36 | 6.773 | .017 |
| e30 | <--> | Age | 5.730 | .026 |
| e30 | <--> | e51 | 6.199 | .014 |
| e30 | <--> | e46 | 4.300 | -.012 |
| e29 | <--> | e53 | 4.965 | -.012 |
| e29 | <--> | e50 | 4.800 | -.012 |
| e29 | <--> | e48 | 4.145 | .010 |
| e28 | <--> | e47 | 8.233 | .017 |
| e27 | <--> | e52 | 7.114 | .015 |
| e26 | <--> | e43 | 5.985 | -.017 |
| e25 | <--> | AISE | 5.709 | .024 |
| e25 | <--> | e35 | 4.025 | -.013 |
| e24 | <--> | Age | 6.662 | .028 |
| e24 | <--> | e45 | 6.562 | .018 |
| e24 | <--> | e30 | 4.049 | .014 |
| e23 | <--> | e52 | 4.289 | -.012 |
| e23 | <--> | e41 | 5.745 | -.016 |
| e23 | <--> | e37 | 6.420 | .017 |
| e23 | <--> | e28 | 4.973 | .015 |
| e22 | <--> | AISE | 5.614 | -.023 |
| e21 | <--> | e37 | 6.504 | .017 |
| e20 | <--> | e39 | 5.255 | -.015 |
| e20 | <--> | e24 | 4.750 | -.015 |
| e20 | <--> | e23 | 6.939 | .017 |
| e19 | <--> | e33 | 4.132 | .014 |
| e18 | <--> | e45 | 4.288 | .014 |
| e17 | <--> | Age | 4.770 | .023 |
| e16 | <--> | Age | 11.990 | -.038 |
| e16 | <--> | e45 | 4.534 | -.015 |
| e16 | <--> | e27 | 4.714 | -.015 |
| e16 | <--> | e26 | 6.200 | .017 |
| e16 | <--> | e23 | 5.253 | -.016 |
| e15 | <--> | e21 | 5.601 | .015 |
| e15 | <--> | e18 | 11.273 | -.021 |
| e14 | <--> | e18 | 7.470 | .017 |
| e13 | <--> | e32 | 4.114 | -.012 |
| e13 | <--> | e29 | 7.813 | .017 |
| e12 | <--> | e27 | 4.523 | -.014 |
| e12 | <--> | e21 | 5.133 | .015 |
| e11 | <--> | e47 | 5.491 | -.013 |
| e11 | <--> | e46 | 4.649 | .011 |
| e11 | <--> | e23 | 4.169 | -.013 |
| e11 | <--> | e15 | 4.889 | .013 |
| e11 | <--> | e12 | 6.019 | .014 |
| e10 | <--> | Age | 6.869 | .026 |
| e10 | <--> | e42 | 5.484 | -.014 |
| e10 | <--> | e33 | 4.752 | -.014 |
| e10 | <--> | e15 | 10.446 | -.019 |
| e10 | <--> | e12 | 6.186 | -.014 |
| e9 | <--> | e21 | 8.375 | -.019 |
| e9 | <--> | e20 | 8.761 | .018 |
| e9 | <--> | e11 | 9.188 | -.017 |
| e9 | <--> | e10 | 6.011 | .014 |
| e8 | <--> | e32 | 5.376 | .015 |
| e8 | <--> | e28 | 6.793 | -.016 |
| e8 | <--> | e21 | 6.241 | -.016 |
| e8 | <--> | e12 | 4.831 | -.013 |
| e8 | <--> | e10 | 8.288 | .016 |
| e8 | <--> | e9 | 5.734 | .014 |
| e7 | <--> | e11 | 5.560 | -.014 |
| e7 | <--> | e8 | 5.643 | .014 |
| e6 | <--> | e37 | 4.694 | -.019 |
| e6 | <--> | e25 | 5.184 | .020 |
| e6 | <--> | e22 | 7.450 | -.024 |
| e6 | <--> | e13 | 4.879 | -.018 |
| e5 | <--> | e13 | 4.241 | .015 |
| e5 | <--> | e6 | 6.857 | .028 |
| e4 | <--> | e50 | 7.725 | -.020 |
| e4 | <--> | e41 | 9.698 | -.027 |
| e3 | <--> | e30 | 4.055 | .018 |
| e3 | <--> | e27 | 9.121 | -.027 |
| e2 | <--> | e37 | 4.339 | .018 |
| e1 | <--> | e24 | 5.422 | -.022 |
| e1 | <--> | e5 | 9.281 | -.036 |

## Variances: (Group number 1 - Default model)

|  |  |  | M.I. | Par Change |
| --- | --- | --- | --- | --- |

## Regression Weights: (Group number 1 - Default model)

|  |  |  | M.I. | Par Change |
| --- | --- | --- | --- | --- |
| Pa | <--- | Gender | 5.706 | -.049 |
| EE\_20 | <--- | ESE\_1 | 4.018 | -.031 |
| EE\_19 | <--- | EOE | 4.737 | .053 |
| EE\_19 | <--- | EOE\_9 | 4.254 | .034 |
| EE\_19 | <--- | EOE\_6 | 6.696 | .043 |
| EE\_19 | <--- | EOE\_5 | 6.112 | .041 |
| EE\_16 | <--- | AISE | 4.896 | -.035 |
| EE\_16 | <--- | AISE\_6 | 4.716 | -.024 |
| EE\_16 | <--- | AISE\_4 | 12.133 | -.039 |
| EE\_16 | <--- | AISE\_1 | 4.055 | -.022 |
| EE\_15 | <--- | EE\_19 | 4.548 | .033 |
| EE\_15 | <--- | ESE\_6 | 5.703 | -.035 |
| EE\_15 | <--- | ESE\_3 | 5.267 | -.035 |
| EE\_14 | <--- | AISE\_6 | 4.072 | .023 |
| EE\_13 | <--- | Age | 4.964 | .029 |
| EE\_13 | <--- | ESE\_5 | 5.463 | .035 |
| EE\_13 | <--- | ESE\_4 | 4.928 | .033 |
| EE\_13 | <--- | ESE\_3 | 5.299 | .035 |
| EE\_12 | <--- | Age | 5.748 | .031 |
| EE\_12 | <--- | ESE\_8 | 4.459 | .033 |
| EE\_12 | <--- | ESE\_6 | 4.703 | .032 |
| EE\_12 | <--- | EOE\_5 | 4.719 | .035 |
| EE\_12 | <--- | EOE\_3 | 4.275 | .033 |
| EE\_12 | <--- | AISE\_2 | 4.878 | .025 |
| EE\_11 | <--- | Sm | 4.780 | -.050 |
| EE\_11 | <--- | ESE\_6 | 4.299 | -.031 |
| EE\_11 | <--- | ESE\_2 | 5.798 | -.037 |
| EE\_11 | <--- | EOE\_9 | 6.295 | -.041 |
| EE\_11 | <--- | EOE\_5 | 5.130 | -.037 |
| EE\_6 | <--- | Grade | 6.273 | -.034 |
| EE\_6 | <--- | Gender | 5.913 | -.058 |
| EE\_6 | <--- | EE\_11 | 6.328 | .038 |
| EE\_6 | <--- | EE\_8 | 4.237 | .031 |
| EE\_5 | <--- | Age | 5.345 | .031 |
| EE\_4 | <--- | Ss | 4.455 | -.048 |
| EE\_4 | <--- | EE\_17 | 4.259 | -.032 |
| EE\_4 | <--- | EE\_13 | 4.155 | -.031 |
| EE\_4 | <--- | ESE\_9 | 4.759 | -.035 |
| EE\_3 | <--- | Ss | 4.515 | .048 |
| EE\_3 | <--- | ESE\_8 | 7.771 | .043 |
| EE\_3 | <--- | ESE\_7 | 5.510 | .037 |
| EE\_2 | <--- | EOE\_9 | 4.816 | .037 |
| EE\_2 | <--- | EOE\_7 | 4.419 | .035 |
| EE\_2 | <--- | EOE\_5 | 6.389 | .042 |
| EE\_2 | <--- | EOE\_2 | 5.679 | .040 |
| EE\_2 | <--- | AISE\_3 | 5.180 | -.027 |
| EE\_1 | <--- | ESE\_1 | 6.902 | .041 |
| ESE\_10 | <--- | AISE | 4.761 | .035 |
| ESE\_10 | <--- | Vc | 4.124 | .044 |
| ESE\_10 | <--- | EE\_16 | 6.588 | .040 |
| ESE\_10 | <--- | EE\_14 | 5.859 | .037 |
| ESE\_10 | <--- | ESE\_6 | 4.830 | .033 |
| ESE\_10 | <--- | AISE\_6 | 8.647 | .033 |
| ESE\_10 | <--- | AISE\_2 | 4.127 | .024 |
| ESE\_10 | <--- | AISE\_1 | 7.673 | .030 |
| ESE\_9 | <--- | Age | 6.679 | .034 |
| ESE\_8 | <--- | EE\_12 | 7.341 | .041 |
| ESE\_8 | <--- | EE\_3 | 5.896 | .037 |
| ESE\_8 | <--- | EOE\_5 | 4.590 | -.035 |
| ESE\_7 | <--- | AISE | 5.831 | -.038 |
| ESE\_7 | <--- | AISE\_6 | 11.441 | -.038 |
| ESE\_7 | <--- | AISE\_3 | 4.709 | -.025 |
| ESE\_7 | <--- | AISE\_2 | 5.418 | -.027 |
| ESE\_5 | <--- | ESE\_8 | 4.431 | .032 |
| ESE\_3 | <--- | EOE\_9 | 4.531 | -.035 |
| ESE\_2 | <--- | Age | 4.279 | .027 |
| ESE\_1 | <--- | Age | 11.448 | -.046 |
| ESE\_1 | <--- | EE\_20 | 4.416 | -.034 |
| ESE\_1 | <--- | EE\_2 | 5.471 | -.036 |
| EOE\_9 | <--- | ESE\_3 | 6.136 | -.037 |
| EOE\_9 | <--- | EOE\_4 | 4.551 | -.033 |
| EOE\_8 | <--- | ESE\_3 | 4.577 | .031 |
| EOE\_7 | <--- | EE\_4 | 6.174 | .034 |
| EOE\_7 | <--- | ESE\_2 | 4.035 | -.028 |
| EOE\_6 | <--- | EE\_2 | 4.080 | -.029 |
| EOE\_5 | <--- | EOE\_3 | 4.047 | -.030 |
| EOE\_4 | <--- | Age | 7.518 | .033 |
| EOE\_4 | <--- | EE\_5 | 4.115 | .028 |
| EOE\_4 | <--- | EE\_4 | 5.740 | .034 |
| EOE\_4 | <--- | EOE\_9 | 4.644 | -.032 |
| EOE\_4 | <--- | AISE\_1 | 4.958 | .022 |
| EOE\_2 | <--- | EE\_3 | 5.552 | -.033 |
| AISE\_6 | <--- | EOE\_7 | 4.674 | -.047 |
| AISE\_5 | <--- | AISE\_1 | 4.027 | -.027 |
| AISE\_4 | <--- | EE\_16 | 7.488 | -.056 |
| AISE\_3 | <--- | EE\_2 | 5.000 | -.044 |

## Bootstrap (Group number 1 - Default model)

## Bootstrap standard errors (Group number 1 - Default model)

## Scalar Estimates (Group number 1 - Default model)

## Regression Weights: (Group number 1 - Default model)

| Parameter | | | SE | SE-SE | Mean | Bias | SE-Bias |
| --- | --- | --- | --- | --- | --- | --- | --- |
| EOE | <--- | AISE | .017 | .000 | .363 | .000 | .000 |
| EOE | <--- | Gender | .021 | .000 | -.037 | .000 | .000 |
| EOE | <--- | Age | .011 | .000 | .020 | .000 | .000 |
| EOE | <--- | Grade | .011 | .000 | .016 | .000 | .000 |
| ESE | <--- | AISE | .017 | .000 | .242 | .000 | .000 |
| ESE | <--- | EOE | .026 | .000 | .257 | .001 | .000 |
| ESE | <--- | Gender | .020 | .000 | -.045 | .000 | .000 |
| ESE | <--- | Age | .011 | .000 | .000 | .000 | .000 |
| ESE | <--- | Grade | .011 | .000 | .009 | .000 | .000 |
| EE | <--- | ESE | .038 | .000 | .232 | .000 | .001 |
| EE | <--- | EOE | .024 | .000 | .176 | .000 | .000 |
| EE | <--- | AISE | .019 | .000 | .142 | .000 | .000 |
| EE | <--- | Gender | .018 | .000 | -.049 | .000 | .000 |
| EE | <--- | Age | .010 | .000 | .016 | .000 | .000 |
| EE | <--- | Grade | .010 | .000 | .014 | .000 | .000 |
| Sm | <--- | ESE | .000 | .000 | 1.000 | .000 | .000 |
| Ss | <--- | ESE | .050 | .000 | .994 | .001 | .001 |
| Vp | <--- | EE | .000 | .000 | 1.000 | .000 | .000 |
| Fs | <--- | EE | .044 | .000 | 1.036 | .002 | .001 |
| Vc | <--- | EE | .045 | .000 | 1.063 | .002 | .001 |
| Pa | <--- | EE | .043 | .000 | .980 | .002 | .001 |
| AISE\_1 | <--- | AISE | .000 | .000 | 1.000 | .000 | .000 |
| AISE\_2 | <--- | AISE | .026 | .000 | .983 | .000 | .000 |
| AISE\_3 | <--- | AISE | .026 | .000 | .960 | .001 | .000 |
| AISE\_4 | <--- | AISE | .027 | .000 | .992 | .000 | .000 |
| AISE\_5 | <--- | AISE | .026 | .000 | .955 | .000 | .000 |
| AISE\_6 | <--- | AISE | .027 | .000 | .999 | .000 | .000 |
| EOE\_1 | <--- | EOE | .000 | .000 | 1.000 | .000 | .000 |
| EOE\_2 | <--- | EOE | .029 | .000 | 1.014 | .001 | .000 |
| EOE\_3 | <--- | EOE | .028 | .000 | 1.003 | .001 | .000 |
| EOE\_4 | <--- | EOE | .029 | .000 | .994 | .000 | .000 |
| EOE\_5 | <--- | EOE | .030 | .000 | 1.033 | .001 | .000 |
| EOE\_6 | <--- | EOE | .029 | .000 | .992 | .000 | .000 |
| EOE\_7 | <--- | EOE | .030 | .000 | 1.031 | .001 | .000 |
| EOE\_8 | <--- | EOE | .030 | .000 | 1.025 | .001 | .000 |
| EOE\_9 | <--- | EOE | .030 | .000 | 1.002 | .000 | .000 |
| ESE\_1 | <--- | Sm | .000 | .000 | 1.000 | .000 | .000 |
| ESE\_2 | <--- | Sm | .031 | .000 | 1.028 | .001 | .000 |
| ESE\_3 | <--- | Sm | .031 | .000 | .982 | .000 | .000 |
| ESE\_4 | <--- | Sm | .031 | .000 | 1.048 | .001 | .000 |
| ESE\_5 | <--- | Sm | .032 | .000 | 1.034 | .001 | .000 |
| ESE\_6 | <--- | Sm | .031 | .000 | 1.028 | .000 | .000 |
| ESE\_7 | <--- | Ss | .000 | .000 | 1.000 | .000 | .000 |
| ESE\_8 | <--- | Ss | .028 | .000 | 1.005 | .000 | .000 |
| ESE\_9 | <--- | Ss | .029 | .000 | .946 | .000 | .000 |
| ESE\_10 | <--- | Ss | .030 | .000 | .981 | .000 | .000 |
| EE\_1 | <--- | Vp | .000 | .000 | 1.000 | .000 | .000 |
| EE\_2 | <--- | Vp | .031 | .000 | 1.021 | .001 | .000 |
| EE\_3 | <--- | Vp | .030 | .000 | 1.049 | .000 | .000 |
| EE\_4 | <--- | Vp | .032 | .000 | 1.023 | .001 | .000 |
| EE\_5 | <--- | Vp | .032 | .000 | 1.027 | .000 | .000 |
| EE\_6 | <--- | Vp | .032 | .000 | 1.038 | .001 | .000 |
| EE\_7 | <--- | Fs | .000 | .000 | 1.000 | .000 | .000 |
| EE\_8 | <--- | Fs | .030 | .000 | .972 | .000 | .000 |
| EE\_9 | <--- | Fs | .030 | .000 | 1.007 | .000 | .000 |
| EE\_10 | <--- | Fs | .030 | .000 | 1.008 | .000 | .000 |
| EE\_11 | <--- | Fs | .030 | .000 | 1.004 | .001 | .000 |
| EE\_12 | <--- | Fs | .030 | .000 | .994 | .000 | .000 |
| EE\_13 | <--- | Vc | .000 | .000 | 1.000 | .000 | .000 |
| EE\_14 | <--- | Vc | .027 | .000 | .973 | .000 | .000 |
| EE\_15 | <--- | Vc | .028 | .000 | .989 | .000 | .000 |
| EE\_16 | <--- | Vc | .028 | .000 | .977 | .000 | .000 |
| EE\_17 | <--- | Vc | .030 | .000 | .969 | .000 | .000 |
| EE\_18 | <--- | Pa | .000 | .000 | 1.000 | .000 | .000 |
| EE\_19 | <--- | Pa | .031 | .000 | .973 | .000 | .000 |
| EE\_20 | <--- | Pa | .031 | .000 | .990 | .000 | .000 |

## Standardized Regression Weights: (Group number 1 - Default model)

| Parameter | | | SE | SE-SE | Mean | Bias | SE-Bias |
| --- | --- | --- | --- | --- | --- | --- | --- |
| EOE | <--- | AISE | .017 | .000 | .547 | .000 | .000 |
| EOE | <--- | Gender | .019 | .000 | -.035 | .000 | .000 |
| EOE | <--- | Age | .020 | .000 | .034 | .000 | .000 |
| EOE | <--- | Grade | .019 | .000 | .027 | .000 | .000 |
| ESE | <--- | AISE | .027 | .000 | .428 | .000 | .000 |
| ESE | <--- | EOE | .027 | .000 | .302 | .000 | .000 |
| ESE | <--- | Gender | .022 | .000 | -.050 | .000 | .000 |
| ESE | <--- | Age | .022 | .000 | -.001 | .000 | .000 |
| ESE | <--- | Grade | .021 | .000 | .018 | .000 | .000 |
| EE | <--- | ESE | .035 | .000 | .231 | .000 | .001 |
| EE | <--- | EOE | .027 | .000 | .205 | .000 | .000 |
| EE | <--- | AISE | .031 | .000 | .249 | .000 | .000 |
| EE | <--- | Gender | .020 | .000 | -.053 | .000 | .000 |
| EE | <--- | Age | .020 | .000 | .032 | .000 | .000 |
| EE | <--- | Grade | .020 | .000 | .027 | .000 | .000 |
| Sm | <--- | ESE | .017 | .000 | .812 | .000 | .000 |
| Ss | <--- | ESE | .018 | .000 | .784 | .000 | .000 |
| Vp | <--- | EE | .012 | .000 | .807 | .000 | .000 |
| Fs | <--- | EE | .012 | .000 | .813 | .000 | .000 |
| Vc | <--- | EE | .012 | .000 | .823 | .000 | .000 |
| Pa | <--- | EE | .014 | .000 | .804 | .000 | .000 |
| AISE\_1 | <--- | AISE | .011 | .000 | .721 | .000 | .000 |
| AISE\_2 | <--- | AISE | .010 | .000 | .757 | .000 | .000 |
| AISE\_3 | <--- | AISE | .011 | .000 | .736 | .000 | .000 |
| AISE\_4 | <--- | AISE | .011 | .000 | .748 | .000 | .000 |
| AISE\_5 | <--- | AISE | .010 | .000 | .760 | .000 | .000 |
| AISE\_6 | <--- | AISE | .011 | .000 | .752 | .000 | .000 |
| EOE\_1 | <--- | EOE | .011 | .000 | .720 | .000 | .000 |
| EOE\_2 | <--- | EOE | .011 | .000 | .727 | .000 | .000 |
| EOE\_3 | <--- | EOE | .011 | .000 | .721 | .000 | .000 |
| EOE\_4 | <--- | EOE | .011 | .000 | .724 | .000 | .000 |
| EOE\_5 | <--- | EOE | .010 | .000 | .738 | .000 | .000 |
| EOE\_6 | <--- | EOE | .011 | .000 | .716 | .000 | .000 |
| EOE\_7 | <--- | EOE | .010 | .000 | .745 | .000 | .000 |
| EOE\_8 | <--- | EOE | .011 | .000 | .728 | .000 | .000 |
| EOE\_9 | <--- | EOE | .011 | .000 | .718 | .000 | .000 |
| ESE\_1 | <--- | Sm | .012 | .000 | .711 | .000 | .000 |
| ESE\_2 | <--- | Sm | .012 | .000 | .734 | .000 | .000 |
| ESE\_3 | <--- | Sm | .012 | .000 | .713 | .000 | .000 |
| ESE\_4 | <--- | Sm | .011 | .000 | .740 | .000 | .000 |
| ESE\_5 | <--- | Sm | .011 | .000 | .739 | .000 | .000 |
| ESE\_6 | <--- | Sm | .011 | .000 | .721 | .000 | .000 |
| ESE\_7 | <--- | Ss | .011 | .000 | .745 | .000 | .000 |
| ESE\_8 | <--- | Ss | .012 | .000 | .747 | .000 | .000 |
| ESE\_9 | <--- | Ss | .012 | .000 | .716 | .000 | .000 |
| ESE\_10 | <--- | Ss | .013 | .000 | .734 | .000 | .000 |
| EE\_1 | <--- | Vp | .012 | .000 | .719 | .000 | .000 |
| EE\_2 | <--- | Vp | .012 | .000 | .721 | .000 | .000 |
| EE\_3 | <--- | Vp | .011 | .000 | .743 | .000 | .000 |
| EE\_4 | <--- | Vp | .011 | .000 | .732 | .000 | .000 |
| EE\_5 | <--- | Vp | .012 | .000 | .725 | .000 | .000 |
| EE\_6 | <--- | Vp | .011 | .000 | .740 | .000 | .000 |
| EE\_7 | <--- | Fs | .011 | .000 | .729 | .000 | .000 |
| EE\_8 | <--- | Fs | .012 | .000 | .715 | .000 | .000 |
| EE\_9 | <--- | Fs | .011 | .000 | .739 | .000 | .000 |
| EE\_10 | <--- | Fs | .011 | .000 | .737 | .000 | .000 |
| EE\_11 | <--- | Fs | .011 | .000 | .736 | .000 | .000 |
| EE\_12 | <--- | Fs | .011 | .000 | .735 | .000 | .000 |
| EE\_13 | <--- | Vc | .011 | .000 | .750 | .000 | .000 |
| EE\_14 | <--- | Vc | .011 | .000 | .734 | .000 | .000 |
| EE\_15 | <--- | Vc | .011 | .000 | .748 | .000 | .000 |
| EE\_16 | <--- | Vc | .011 | .000 | .735 | .000 | .000 |
| EE\_17 | <--- | Vc | .012 | .000 | .738 | .000 | .000 |
| EE\_18 | <--- | Pa | .013 | .000 | .738 | .000 | .000 |
| EE\_19 | <--- | Pa | .013 | .000 | .715 | .000 | .000 |
| EE\_20 | <--- | Pa | .013 | .000 | .723 | .000 | .000 |

## Covariances: (Group number 1 - Default model)

| Parameter | | | SE | SE-SE | Mean | Bias | SE-Bias |
| --- | --- | --- | --- | --- | --- | --- | --- |
| Gender | <--> | Age | .009 | .000 | .005 | .000 | .000 |
| Gender | <--> | Grade | .009 | .000 | -.006 | .000 | .000 |
| Age | <--> | Grade | .016 | .000 | .017 | .000 | .000 |
| AISE | <--> | Gender | .009 | .000 | -.018 | .000 | .000 |
| AISE | <--> | Age | .016 | .000 | .070 | .000 | .000 |
| AISE | <--> | Grade | .015 | .000 | .015 | .000 | .000 |

## Correlations: (Group number 1 - Default model)

| Parameter | | | SE | SE-SE | Mean | Bias | SE-Bias |
| --- | --- | --- | --- | --- | --- | --- | --- |
| Gender | <--> | Age | .020 | .000 | .012 | .000 | .000 |
| Gender | <--> | Grade | .021 | .000 | -.013 | .000 | .000 |
| Age | <--> | Grade | .021 | .000 | .021 | .000 | .000 |
| AISE | <--> | Gender | .023 | .000 | -.046 | -.001 | .000 |
| AISE | <--> | Age | .022 | .000 | .099 | .000 | .000 |
| AISE | <--> | Grade | .022 | .000 | .023 | .000 | .000 |

## Variances: (Group number 1 - Default model)

| Parameter | | | SE | SE-SE | Mean | Bias | SE-Bias |
| --- | --- | --- | --- | --- | --- | --- | --- |
| AISE |  |  | .029 | .000 | .623 | .000 | .000 |
| Gender |  |  | .002 | .000 | .242 | .000 | .000 |
| Age |  |  | .020 | .000 | .816 | .000 | .000 |
| Grade |  |  | .009 | .000 | .743 | .000 | .000 |
| e52 |  |  | .010 | .000 | .189 | .000 | .000 |
| e53 |  |  | .009 | .000 | .115 | .000 | .000 |
| e54 |  |  | .009 | .000 | .130 | -.001 | .000 |
| e46 |  |  | .010 | .000 | .103 | .000 | .000 |
| e47 |  |  | .010 | .000 | .123 | .000 | .000 |
| e48 |  |  | .007 | .000 | .107 | .000 | .000 |
| e49 |  |  | .008 | .000 | .110 | .000 | .000 |
| e50 |  |  | .008 | .000 | .108 | .000 | .000 |
| e51 |  |  | .008 | .000 | .106 | .000 | .000 |
| e1 |  |  | .019 | .000 | .576 | -.001 | .000 |
| e2 |  |  | .015 | .000 | .449 | -.001 | .000 |
| e3 |  |  | .016 | .000 | .485 | .000 | .000 |
| e4 |  |  | .017 | .000 | .483 | .000 | .000 |
| e5 |  |  | .014 | .000 | .414 | -.001 | .000 |
| e6 |  |  | .017 | .000 | .477 | .000 | .000 |
| e7 |  |  | .008 | .000 | .255 | .000 | .000 |
| e8 |  |  | .008 | .000 | .251 | .000 | .000 |
| e9 |  |  | .008 | .000 | .255 | .000 | .000 |
| e10 |  |  | .008 | .000 | .246 | .000 | .000 |
| e11 |  |  | .008 | .000 | .244 | .000 | .000 |
| e12 |  |  | .008 | .000 | .256 | .000 | .000 |
| e13 |  |  | .007 | .000 | .233 | .000 | .000 |
| e14 |  |  | .008 | .000 | .255 | .000 | .000 |
| e15 |  |  | .008 | .000 | .258 | .000 | .000 |
| e16 |  |  | .010 | .000 | .295 | .000 | .000 |
| e17 |  |  | .010 | .000 | .274 | -.001 | .000 |
| e18 |  |  | .010 | .000 | .283 | .000 | .000 |
| e19 |  |  | .009 | .000 | .275 | .000 | .000 |
| e20 |  |  | .009 | .000 | .268 | .000 | .000 |
| e21 |  |  | .010 | .000 | .296 | .000 | .000 |
| e22 |  |  | .009 | .000 | .257 | .000 | .000 |
| e23 |  |  | .010 | .000 | .255 | .000 | .000 |
| e24 |  |  | .010 | .000 | .271 | .000 | .000 |
| e25 |  |  | .010 | .000 | .262 | .000 | .000 |
| e26 |  |  | .010 | .000 | .288 | .000 | .000 |
| e27 |  |  | .010 | .000 | .296 | .000 | .000 |
| e28 |  |  | .009 | .000 | .275 | .000 | .000 |
| e29 |  |  | .009 | .000 | .280 | .000 | .000 |
| e30 |  |  | .010 | .000 | .292 | .000 | .000 |
| e31 |  |  | .010 | .000 | .274 | .000 | .000 |
| e32 |  |  | .009 | .000 | .287 | .000 | .000 |
| e33 |  |  | .009 | .000 | .294 | .000 | .000 |
| e34 |  |  | .009 | .000 | .273 | .000 | .000 |
| e35 |  |  | .009 | .000 | .278 | .000 | .000 |
| e36 |  |  | .010 | .000 | .277 | .000 | .000 |
| e37 |  |  | .009 | .000 | .273 | .000 | .000 |
| e38 |  |  | .009 | .000 | .259 | .000 | .000 |
| e39 |  |  | .009 | .000 | .270 | .000 | .000 |
| e40 |  |  | .009 | .000 | .256 | .000 | .000 |
| e41 |  |  | .009 | .000 | .271 | .000 | .000 |
| e42 |  |  | .009 | .000 | .262 | .000 | .000 |
| e43 |  |  | .010 | .000 | .249 | .000 | .000 |
| e44 |  |  | .010 | .000 | .270 | .000 | .000 |
| e45 |  |  | .011 | .000 | .266 | .000 | .000 |

## User-defined estimands: (Group number 1 - Default model)

| Parameter | | | SE | SE-SE | Mean | Bias | SE-Bias |
| --- | --- | --- | --- | --- | --- | --- | --- |
| MedIndirect1 |  |  | .017 | .000 | .099 | .000 | .000 |
| MedDirect1 |  |  | .031 | .000 | .249 | .000 | .000 |
| MedTotal1 |  |  | .026 | .000 | .348 | .000 | .000 |
| MedIndirect2 |  |  | .015 | .000 | .112 | .000 | .000 |
| MedDirect2 |  |  | .031 | .000 | .249 | .000 | .000 |
| MedTotal2 |  |  | .030 | .000 | .362 | .000 | .000 |
| MedIndirect3 |  |  | .021 | .000 | .249 | .000 | .000 |
| MedDirect3 |  |  | .031 | .000 | .249 | .000 | .000 |
| MedTotal3 |  |  | .019 | .000 | .499 | .000 | .000 |

## Matrices (Group number 1 - Default model)

## Total Effects - Standard Errors (Group number 1 - Default model)

|  | Grade | Age | Gender | AISE | EOE | ESE | EE | Pa | Vc | Fs | Vp | Ss | Sm |
| --- | --- | --- | --- | --- | --- | --- | --- | --- | --- | --- | --- | --- | --- |
| EOE | .011 | .011 | .021 | .017 | .000 | .000 | .000 | .000 | .000 | .000 | .000 | .000 | .000 |
| ESE | .011 | .011 | .021 | .017 | .026 | .000 | .000 | .000 | .000 | .000 | .000 | .000 | .000 |
| EE | .011 | .010 | .019 | .016 | .023 | .038 | .000 | .000 | .000 | .000 | .000 | .000 | .000 |
| Pa | .011 | .010 | .019 | .015 | .023 | .037 | .043 | .000 | .000 | .000 | .000 | .000 | .000 |
| Vc | .011 | .011 | .020 | .016 | .025 | .040 | .045 | .000 | .000 | .000 | .000 | .000 | .000 |
| Fs | .011 | .010 | .020 | .016 | .024 | .040 | .044 | .000 | .000 | .000 | .000 | .000 | .000 |
| Vp | .011 | .010 | .019 | .016 | .023 | .038 | .000 | .000 | .000 | .000 | .000 | .000 | .000 |
| Ss | .011 | .011 | .020 | .017 | .025 | .050 | .000 | .000 | .000 | .000 | .000 | .000 | .000 |
| Sm | .011 | .011 | .021 | .017 | .026 | .000 | .000 | .000 | .000 | .000 | .000 | .000 | .000 |
| EE\_20 | .010 | .010 | .019 | .015 | .023 | .037 | .041 | .031 | .000 | .000 | .000 | .000 | .000 |
| EE\_19 | .010 | .010 | .018 | .015 | .023 | .036 | .040 | .031 | .000 | .000 | .000 | .000 | .000 |
| EE\_18 | .011 | .010 | .019 | .015 | .023 | .037 | .043 | .000 | .000 | .000 | .000 | .000 | .000 |
| EE\_17 | .011 | .010 | .020 | .016 | .024 | .039 | .044 | .000 | .030 | .000 | .000 | .000 | .000 |
| EE\_16 | .011 | .011 | .020 | .016 | .025 | .039 | .044 | .000 | .028 | .000 | .000 | .000 | .000 |
| EE\_15 | .011 | .011 | .020 | .016 | .024 | .040 | .044 | .000 | .028 | .000 | .000 | .000 | .000 |
| EE\_14 | .011 | .010 | .020 | .016 | .024 | .039 | .042 | .000 | .027 | .000 | .000 | .000 | .000 |
| EE\_13 | .011 | .011 | .020 | .016 | .025 | .040 | .045 | .000 | .000 | .000 | .000 | .000 | .000 |
| EE\_12 | .011 | .010 | .020 | .016 | .024 | .039 | .044 | .000 | .000 | .030 | .000 | .000 | .000 |
| EE\_11 | .011 | .011 | .020 | .016 | .024 | .040 | .044 | .000 | .000 | .030 | .000 | .000 | .000 |
| EE\_10 | .011 | .011 | .020 | .016 | .024 | .040 | .045 | .000 | .000 | .030 | .000 | .000 | .000 |
| EE\_9 | .011 | .011 | .020 | .016 | .024 | .040 | .044 | .000 | .000 | .030 | .000 | .000 | .000 |
| EE\_8 | .011 | .010 | .019 | .016 | .024 | .038 | .044 | .000 | .000 | .030 | .000 | .000 | .000 |
| EE\_7 | .011 | .010 | .020 | .016 | .024 | .040 | .044 | .000 | .000 | .000 | .000 | .000 | .000 |
| EE\_6 | .011 | .011 | .020 | .016 | .024 | .039 | .032 | .000 | .000 | .000 | .032 | .000 | .000 |
| EE\_5 | .011 | .010 | .020 | .016 | .024 | .039 | .032 | .000 | .000 | .000 | .032 | .000 | .000 |
| EE\_4 | .011 | .010 | .020 | .016 | .024 | .038 | .032 | .000 | .000 | .000 | .032 | .000 | .000 |
| EE\_3 | .011 | .011 | .020 | .016 | .024 | .040 | .030 | .000 | .000 | .000 | .030 | .000 | .000 |
| EE\_2 | .011 | .010 | .019 | .016 | .024 | .039 | .031 | .000 | .000 | .000 | .031 | .000 | .000 |
| EE\_1 | .011 | .010 | .019 | .016 | .023 | .038 | .000 | .000 | .000 | .000 | .000 | .000 | .000 |
| ESE\_10 | .011 | .011 | .020 | .018 | .024 | .051 | .000 | .000 | .000 | .000 | .000 | .030 | .000 |
| ESE\_9 | .011 | .010 | .019 | .016 | .023 | .048 | .000 | .000 | .000 | .000 | .000 | .029 | .000 |
| ESE\_8 | .011 | .011 | .021 | .017 | .025 | .051 | .000 | .000 | .000 | .000 | .000 | .028 | .000 |
| ESE\_7 | .011 | .011 | .020 | .017 | .025 | .050 | .000 | .000 | .000 | .000 | .000 | .000 | .000 |
| ESE\_6 | .012 | .011 | .022 | .017 | .026 | .031 | .000 | .000 | .000 | .000 | .000 | .000 | .031 |
| ESE\_5 | .012 | .012 | .022 | .018 | .026 | .032 | .000 | .000 | .000 | .000 | .000 | .000 | .032 |
| ESE\_4 | .012 | .012 | .022 | .017 | .027 | .031 | .000 | .000 | .000 | .000 | .000 | .000 | .031 |
| ESE\_3 | .011 | .011 | .021 | .017 | .025 | .031 | .000 | .000 | .000 | .000 | .000 | .000 | .031 |
| ESE\_2 | .012 | .011 | .022 | .017 | .026 | .031 | .000 | .000 | .000 | .000 | .000 | .000 | .031 |
| ESE\_1 | .011 | .011 | .021 | .017 | .026 | .000 | .000 | .000 | .000 | .000 | .000 | .000 | .000 |
| EOE\_9 | .011 | .011 | .021 | .017 | .030 | .000 | .000 | .000 | .000 | .000 | .000 | .000 | .000 |
| EOE\_8 | .012 | .012 | .021 | .017 | .030 | .000 | .000 | .000 | .000 | .000 | .000 | .000 | .000 |
| EOE\_7 | .012 | .012 | .021 | .017 | .030 | .000 | .000 | .000 | .000 | .000 | .000 | .000 | .000 |
| EOE\_6 | .011 | .011 | .020 | .016 | .029 | .000 | .000 | .000 | .000 | .000 | .000 | .000 | .000 |
| EOE\_5 | .012 | .012 | .021 | .017 | .030 | .000 | .000 | .000 | .000 | .000 | .000 | .000 | .000 |
| EOE\_4 | .011 | .011 | .020 | .017 | .029 | .000 | .000 | .000 | .000 | .000 | .000 | .000 | .000 |
| EOE\_3 | .011 | .011 | .021 | .017 | .028 | .000 | .000 | .000 | .000 | .000 | .000 | .000 | .000 |
| EOE\_2 | .011 | .012 | .021 | .017 | .029 | .000 | .000 | .000 | .000 | .000 | .000 | .000 | .000 |
| EOE\_1 | .011 | .011 | .021 | .017 | .000 | .000 | .000 | .000 | .000 | .000 | .000 | .000 | .000 |
| AISE\_6 | .000 | .000 | .000 | .027 | .000 | .000 | .000 | .000 | .000 | .000 | .000 | .000 | .000 |
| AISE\_5 | .000 | .000 | .000 | .026 | .000 | .000 | .000 | .000 | .000 | .000 | .000 | .000 | .000 |
| AISE\_4 | .000 | .000 | .000 | .027 | .000 | .000 | .000 | .000 | .000 | .000 | .000 | .000 | .000 |
| AISE\_3 | .000 | .000 | .000 | .026 | .000 | .000 | .000 | .000 | .000 | .000 | .000 | .000 | .000 |
| AISE\_2 | .000 | .000 | .000 | .026 | .000 | .000 | .000 | .000 | .000 | .000 | .000 | .000 | .000 |
| AISE\_1 | .000 | .000 | .000 | .000 | .000 | .000 | .000 | .000 | .000 | .000 | .000 | .000 | .000 |

## Standardized Total Effects - Standard Errors (Group number 1 - Default model)

|  | Grade | Age | Gender | AISE | EOE | ESE | EE | Pa | Vc | Fs | Vp | Ss | Sm |
| --- | --- | --- | --- | --- | --- | --- | --- | --- | --- | --- | --- | --- | --- |
| EOE | .019 | .020 | .019 | .017 | .000 | .000 | .000 | .000 | .000 | .000 | .000 | .000 | .000 |
| ESE | .022 | .022 | .023 | .020 | .027 | .000 | .000 | .000 | .000 | .000 | .000 | .000 | .000 |
| EE | .021 | .020 | .021 | .019 | .025 | .035 | .000 | .000 | .000 | .000 | .000 | .000 | .000 |
| Pa | .017 | .016 | .017 | .017 | .021 | .029 | .014 | .000 | .000 | .000 | .000 | .000 | .000 |
| Vc | .017 | .017 | .017 | .017 | .021 | .029 | .012 | .000 | .000 | .000 | .000 | .000 | .000 |
| Fs | .017 | .017 | .017 | .017 | .021 | .029 | .012 | .000 | .000 | .000 | .000 | .000 | .000 |
| Vp | .017 | .016 | .017 | .017 | .021 | .029 | .012 | .000 | .000 | .000 | .000 | .000 | .000 |
| Ss | .017 | .018 | .018 | .019 | .021 | .018 | .000 | .000 | .000 | .000 | .000 | .000 | .000 |
| Sm | .018 | .018 | .019 | .018 | .023 | .017 | .000 | .000 | .000 | .000 | .000 | .000 | .000 |
| EE\_20 | .012 | .012 | .012 | .014 | .016 | .021 | .015 | .013 | .000 | .000 | .000 | .000 | .000 |
| EE\_19 | .012 | .012 | .012 | .014 | .016 | .021 | .015 | .013 | .000 | .000 | .000 | .000 | .000 |
| EE\_18 | .012 | .012 | .012 | .014 | .016 | .021 | .015 | .013 | .000 | .000 | .000 | .000 | .000 |
| EE\_17 | .013 | .012 | .013 | .014 | .016 | .022 | .013 | .000 | .012 | .000 | .000 | .000 | .000 |
| EE\_16 | .013 | .012 | .013 | .014 | .016 | .022 | .014 | .000 | .011 | .000 | .000 | .000 | .000 |
| EE\_15 | .013 | .013 | .013 | .014 | .016 | .022 | .013 | .000 | .011 | .000 | .000 | .000 | .000 |
| EE\_14 | .013 | .012 | .013 | .014 | .016 | .022 | .013 | .000 | .011 | .000 | .000 | .000 | .000 |
| EE\_13 | .013 | .013 | .013 | .014 | .016 | .022 | .013 | .000 | .011 | .000 | .000 | .000 | .000 |
| EE\_12 | .012 | .012 | .012 | .014 | .016 | .021 | .014 | .000 | .000 | .011 | .000 | .000 | .000 |
| EE\_11 | .012 | .012 | .012 | .014 | .016 | .021 | .014 | .000 | .000 | .011 | .000 | .000 | .000 |
| EE\_10 | .012 | .012 | .012 | .014 | .016 | .022 | .013 | .000 | .000 | .011 | .000 | .000 | .000 |
| EE\_9 | .012 | .012 | .013 | .014 | .016 | .022 | .014 | .000 | .000 | .011 | .000 | .000 | .000 |
| EE\_8 | .012 | .012 | .012 | .014 | .015 | .021 | .014 | .000 | .000 | .012 | .000 | .000 | .000 |
| EE\_7 | .012 | .012 | .012 | .014 | .016 | .021 | .013 | .000 | .000 | .011 | .000 | .000 | .000 |
| EE\_6 | .012 | .012 | .012 | .014 | .016 | .021 | .014 | .000 | .000 | .000 | .011 | .000 | .000 |
| EE\_5 | .012 | .012 | .012 | .014 | .015 | .021 | .014 | .000 | .000 | .000 | .012 | .000 | .000 |
| EE\_4 | .012 | .012 | .012 | .014 | .015 | .021 | .013 | .000 | .000 | .000 | .011 | .000 | .000 |
| EE\_3 | .012 | .012 | .013 | .014 | .016 | .021 | .014 | .000 | .000 | .000 | .011 | .000 | .000 |
| EE\_2 | .012 | .012 | .012 | .014 | .015 | .021 | .014 | .000 | .000 | .000 | .012 | .000 | .000 |
| EE\_1 | .012 | .012 | .012 | .014 | .015 | .021 | .014 | .000 | .000 | .000 | .012 | .000 | .000 |
| ESE\_10 | .013 | .013 | .013 | .016 | .016 | .017 | .000 | .000 | .000 | .000 | .000 | .013 | .000 |
| ESE\_9 | .012 | .013 | .013 | .015 | .016 | .016 | .000 | .000 | .000 | .000 | .000 | .012 | .000 |
| ESE\_8 | .013 | .013 | .013 | .016 | .016 | .017 | .000 | .000 | .000 | .000 | .000 | .012 | .000 |
| ESE\_7 | .013 | .013 | .013 | .015 | .016 | .016 | .000 | .000 | .000 | .000 | .000 | .011 | .000 |
| ESE\_6 | .013 | .013 | .013 | .015 | .017 | .016 | .000 | .000 | .000 | .000 | .000 | .000 | .011 |
| ESE\_5 | .013 | .014 | .014 | .015 | .017 | .016 | .000 | .000 | .000 | .000 | .000 | .000 | .011 |
| ESE\_4 | .013 | .014 | .014 | .015 | .017 | .016 | .000 | .000 | .000 | .000 | .000 | .000 | .011 |
| ESE\_3 | .013 | .013 | .013 | .015 | .017 | .016 | .000 | .000 | .000 | .000 | .000 | .000 | .012 |
| ESE\_2 | .013 | .013 | .014 | .015 | .017 | .017 | .000 | .000 | .000 | .000 | .000 | .000 | .012 |
| ESE\_1 | .013 | .013 | .013 | .015 | .017 | .016 | .000 | .000 | .000 | .000 | .000 | .000 | .012 |
| EOE\_9 | .013 | .014 | .014 | .015 | .011 | .000 | .000 | .000 | .000 | .000 | .000 | .000 | .000 |
| EOE\_8 | .014 | .014 | .014 | .015 | .011 | .000 | .000 | .000 | .000 | .000 | .000 | .000 | .000 |
| EOE\_7 | .014 | .015 | .014 | .015 | .010 | .000 | .000 | .000 | .000 | .000 | .000 | .000 | .000 |
| EOE\_6 | .013 | .014 | .014 | .014 | .011 | .000 | .000 | .000 | .000 | .000 | .000 | .000 | .000 |
| EOE\_5 | .014 | .014 | .014 | .015 | .010 | .000 | .000 | .000 | .000 | .000 | .000 | .000 | .000 |
| EOE\_4 | .013 | .014 | .014 | .015 | .011 | .000 | .000 | .000 | .000 | .000 | .000 | .000 | .000 |
| EOE\_3 | .013 | .014 | .014 | .014 | .011 | .000 | .000 | .000 | .000 | .000 | .000 | .000 | .000 |
| EOE\_2 | .014 | .014 | .014 | .015 | .011 | .000 | .000 | .000 | .000 | .000 | .000 | .000 | .000 |
| EOE\_1 | .013 | .014 | .014 | .015 | .011 | .000 | .000 | .000 | .000 | .000 | .000 | .000 | .000 |
| AISE\_6 | .000 | .000 | .000 | .011 | .000 | .000 | .000 | .000 | .000 | .000 | .000 | .000 | .000 |
| AISE\_5 | .000 | .000 | .000 | .010 | .000 | .000 | .000 | .000 | .000 | .000 | .000 | .000 | .000 |
| AISE\_4 | .000 | .000 | .000 | .011 | .000 | .000 | .000 | .000 | .000 | .000 | .000 | .000 | .000 |
| AISE\_3 | .000 | .000 | .000 | .011 | .000 | .000 | .000 | .000 | .000 | .000 | .000 | .000 | .000 |
| AISE\_2 | .000 | .000 | .000 | .010 | .000 | .000 | .000 | .000 | .000 | .000 | .000 | .000 | .000 |
| AISE\_1 | .000 | .000 | .000 | .011 | .000 | .000 | .000 | .000 | .000 | .000 | .000 | .000 | .000 |

## Direct Effects - Standard Errors (Group number 1 - Default model)

|  | Grade | Age | Gender | AISE | EOE | ESE | EE | Pa | Vc | Fs | Vp | Ss | Sm |
| --- | --- | --- | --- | --- | --- | --- | --- | --- | --- | --- | --- | --- | --- |
| EOE | .011 | .011 | .021 | .017 | .000 | .000 | .000 | .000 | .000 | .000 | .000 | .000 | .000 |
| ESE | .011 | .011 | .020 | .017 | .026 | .000 | .000 | .000 | .000 | .000 | .000 | .000 | .000 |
| EE | .010 | .010 | .018 | .019 | .024 | .038 | .000 | .000 | .000 | .000 | .000 | .000 | .000 |
| Pa | .000 | .000 | .000 | .000 | .000 | .000 | .043 | .000 | .000 | .000 | .000 | .000 | .000 |
| Vc | .000 | .000 | .000 | .000 | .000 | .000 | .045 | .000 | .000 | .000 | .000 | .000 | .000 |
| Fs | .000 | .000 | .000 | .000 | .000 | .000 | .044 | .000 | .000 | .000 | .000 | .000 | .000 |
| Vp | .000 | .000 | .000 | .000 | .000 | .000 | .000 | .000 | .000 | .000 | .000 | .000 | .000 |
| Ss | .000 | .000 | .000 | .000 | .000 | .050 | .000 | .000 | .000 | .000 | .000 | .000 | .000 |
| Sm | .000 | .000 | .000 | .000 | .000 | .000 | .000 | .000 | .000 | .000 | .000 | .000 | .000 |
| EE\_20 | .000 | .000 | .000 | .000 | .000 | .000 | .000 | .031 | .000 | .000 | .000 | .000 | .000 |
| EE\_19 | .000 | .000 | .000 | .000 | .000 | .000 | .000 | .031 | .000 | .000 | .000 | .000 | .000 |
| EE\_18 | .000 | .000 | .000 | .000 | .000 | .000 | .000 | .000 | .000 | .000 | .000 | .000 | .000 |
| EE\_17 | .000 | .000 | .000 | .000 | .000 | .000 | .000 | .000 | .030 | .000 | .000 | .000 | .000 |
| EE\_16 | .000 | .000 | .000 | .000 | .000 | .000 | .000 | .000 | .028 | .000 | .000 | .000 | .000 |
| EE\_15 | .000 | .000 | .000 | .000 | .000 | .000 | .000 | .000 | .028 | .000 | .000 | .000 | .000 |
| EE\_14 | .000 | .000 | .000 | .000 | .000 | .000 | .000 | .000 | .027 | .000 | .000 | .000 | .000 |
| EE\_13 | .000 | .000 | .000 | .000 | .000 | .000 | .000 | .000 | .000 | .000 | .000 | .000 | .000 |
| EE\_12 | .000 | .000 | .000 | .000 | .000 | .000 | .000 | .000 | .000 | .030 | .000 | .000 | .000 |
| EE\_11 | .000 | .000 | .000 | .000 | .000 | .000 | .000 | .000 | .000 | .030 | .000 | .000 | .000 |
| EE\_10 | .000 | .000 | .000 | .000 | .000 | .000 | .000 | .000 | .000 | .030 | .000 | .000 | .000 |
| EE\_9 | .000 | .000 | .000 | .000 | .000 | .000 | .000 | .000 | .000 | .030 | .000 | .000 | .000 |
| EE\_8 | .000 | .000 | .000 | .000 | .000 | .000 | .000 | .000 | .000 | .030 | .000 | .000 | .000 |
| EE\_7 | .000 | .000 | .000 | .000 | .000 | .000 | .000 | .000 | .000 | .000 | .000 | .000 | .000 |
| EE\_6 | .000 | .000 | .000 | .000 | .000 | .000 | .000 | .000 | .000 | .000 | .032 | .000 | .000 |
| EE\_5 | .000 | .000 | .000 | .000 | .000 | .000 | .000 | .000 | .000 | .000 | .032 | .000 | .000 |
| EE\_4 | .000 | .000 | .000 | .000 | .000 | .000 | .000 | .000 | .000 | .000 | .032 | .000 | .000 |
| EE\_3 | .000 | .000 | .000 | .000 | .000 | .000 | .000 | .000 | .000 | .000 | .030 | .000 | .000 |
| EE\_2 | .000 | .000 | .000 | .000 | .000 | .000 | .000 | .000 | .000 | .000 | .031 | .000 | .000 |
| EE\_1 | .000 | .000 | .000 | .000 | .000 | .000 | .000 | .000 | .000 | .000 | .000 | .000 | .000 |
| ESE\_10 | .000 | .000 | .000 | .000 | .000 | .000 | .000 | .000 | .000 | .000 | .000 | .030 | .000 |
| ESE\_9 | .000 | .000 | .000 | .000 | .000 | .000 | .000 | .000 | .000 | .000 | .000 | .029 | .000 |
| ESE\_8 | .000 | .000 | .000 | .000 | .000 | .000 | .000 | .000 | .000 | .000 | .000 | .028 | .000 |
| ESE\_7 | .000 | .000 | .000 | .000 | .000 | .000 | .000 | .000 | .000 | .000 | .000 | .000 | .000 |
| ESE\_6 | .000 | .000 | .000 | .000 | .000 | .000 | .000 | .000 | .000 | .000 | .000 | .000 | .031 |
| ESE\_5 | .000 | .000 | .000 | .000 | .000 | .000 | .000 | .000 | .000 | .000 | .000 | .000 | .032 |
| ESE\_4 | .000 | .000 | .000 | .000 | .000 | .000 | .000 | .000 | .000 | .000 | .000 | .000 | .031 |
| ESE\_3 | .000 | .000 | .000 | .000 | .000 | .000 | .000 | .000 | .000 | .000 | .000 | .000 | .031 |
| ESE\_2 | .000 | .000 | .000 | .000 | .000 | .000 | .000 | .000 | .000 | .000 | .000 | .000 | .031 |
| ESE\_1 | .000 | .000 | .000 | .000 | .000 | .000 | .000 | .000 | .000 | .000 | .000 | .000 | .000 |
| EOE\_9 | .000 | .000 | .000 | .000 | .030 | .000 | .000 | .000 | .000 | .000 | .000 | .000 | .000 |
| EOE\_8 | .000 | .000 | .000 | .000 | .030 | .000 | .000 | .000 | .000 | .000 | .000 | .000 | .000 |
| EOE\_7 | .000 | .000 | .000 | .000 | .030 | .000 | .000 | .000 | .000 | .000 | .000 | .000 | .000 |
| EOE\_6 | .000 | .000 | .000 | .000 | .029 | .000 | .000 | .000 | .000 | .000 | .000 | .000 | .000 |
| EOE\_5 | .000 | .000 | .000 | .000 | .030 | .000 | .000 | .000 | .000 | .000 | .000 | .000 | .000 |
| EOE\_4 | .000 | .000 | .000 | .000 | .029 | .000 | .000 | .000 | .000 | .000 | .000 | .000 | .000 |
| EOE\_3 | .000 | .000 | .000 | .000 | .028 | .000 | .000 | .000 | .000 | .000 | .000 | .000 | .000 |
| EOE\_2 | .000 | .000 | .000 | .000 | .029 | .000 | .000 | .000 | .000 | .000 | .000 | .000 | .000 |
| EOE\_1 | .000 | .000 | .000 | .000 | .000 | .000 | .000 | .000 | .000 | .000 | .000 | .000 | .000 |
| AISE\_6 | .000 | .000 | .000 | .027 | .000 | .000 | .000 | .000 | .000 | .000 | .000 | .000 | .000 |
| AISE\_5 | .000 | .000 | .000 | .026 | .000 | .000 | .000 | .000 | .000 | .000 | .000 | .000 | .000 |
| AISE\_4 | .000 | .000 | .000 | .027 | .000 | .000 | .000 | .000 | .000 | .000 | .000 | .000 | .000 |
| AISE\_3 | .000 | .000 | .000 | .026 | .000 | .000 | .000 | .000 | .000 | .000 | .000 | .000 | .000 |
| AISE\_2 | .000 | .000 | .000 | .026 | .000 | .000 | .000 | .000 | .000 | .000 | .000 | .000 | .000 |
| AISE\_1 | .000 | .000 | .000 | .000 | .000 | .000 | .000 | .000 | .000 | .000 | .000 | .000 | .000 |

## Standardized Direct Effects - Standard Errors (Group number 1 - Default model)

|  | Grade | Age | Gender | AISE | EOE | ESE | EE | Pa | Vc | Fs | Vp | Ss | Sm |
| --- | --- | --- | --- | --- | --- | --- | --- | --- | --- | --- | --- | --- | --- |
| EOE | .019 | .020 | .019 | .017 | .000 | .000 | .000 | .000 | .000 | .000 | .000 | .000 | .000 |
| ESE | .021 | .022 | .022 | .027 | .027 | .000 | .000 | .000 | .000 | .000 | .000 | .000 | .000 |
| EE | .020 | .020 | .020 | .031 | .027 | .035 | .000 | .000 | .000 | .000 | .000 | .000 | .000 |
| Pa | .000 | .000 | .000 | .000 | .000 | .000 | .014 | .000 | .000 | .000 | .000 | .000 | .000 |
| Vc | .000 | .000 | .000 | .000 | .000 | .000 | .012 | .000 | .000 | .000 | .000 | .000 | .000 |
| Fs | .000 | .000 | .000 | .000 | .000 | .000 | .012 | .000 | .000 | .000 | .000 | .000 | .000 |
| Vp | .000 | .000 | .000 | .000 | .000 | .000 | .012 | .000 | .000 | .000 | .000 | .000 | .000 |
| Ss | .000 | .000 | .000 | .000 | .000 | .018 | .000 | .000 | .000 | .000 | .000 | .000 | .000 |
| Sm | .000 | .000 | .000 | .000 | .000 | .017 | .000 | .000 | .000 | .000 | .000 | .000 | .000 |
| EE\_20 | .000 | .000 | .000 | .000 | .000 | .000 | .000 | .013 | .000 | .000 | .000 | .000 | .000 |
| EE\_19 | .000 | .000 | .000 | .000 | .000 | .000 | .000 | .013 | .000 | .000 | .000 | .000 | .000 |
| EE\_18 | .000 | .000 | .000 | .000 | .000 | .000 | .000 | .013 | .000 | .000 | .000 | .000 | .000 |
| EE\_17 | .000 | .000 | .000 | .000 | .000 | .000 | .000 | .000 | .012 | .000 | .000 | .000 | .000 |
| EE\_16 | .000 | .000 | .000 | .000 | .000 | .000 | .000 | .000 | .011 | .000 | .000 | .000 | .000 |
| EE\_15 | .000 | .000 | .000 | .000 | .000 | .000 | .000 | .000 | .011 | .000 | .000 | .000 | .000 |
| EE\_14 | .000 | .000 | .000 | .000 | .000 | .000 | .000 | .000 | .011 | .000 | .000 | .000 | .000 |
| EE\_13 | .000 | .000 | .000 | .000 | .000 | .000 | .000 | .000 | .011 | .000 | .000 | .000 | .000 |
| EE\_12 | .000 | .000 | .000 | .000 | .000 | .000 | .000 | .000 | .000 | .011 | .000 | .000 | .000 |
| EE\_11 | .000 | .000 | .000 | .000 | .000 | .000 | .000 | .000 | .000 | .011 | .000 | .000 | .000 |
| EE\_10 | .000 | .000 | .000 | .000 | .000 | .000 | .000 | .000 | .000 | .011 | .000 | .000 | .000 |
| EE\_9 | .000 | .000 | .000 | .000 | .000 | .000 | .000 | .000 | .000 | .011 | .000 | .000 | .000 |
| EE\_8 | .000 | .000 | .000 | .000 | .000 | .000 | .000 | .000 | .000 | .012 | .000 | .000 | .000 |
| EE\_7 | .000 | .000 | .000 | .000 | .000 | .000 | .000 | .000 | .000 | .011 | .000 | .000 | .000 |
| EE\_6 | .000 | .000 | .000 | .000 | .000 | .000 | .000 | .000 | .000 | .000 | .011 | .000 | .000 |
| EE\_5 | .000 | .000 | .000 | .000 | .000 | .000 | .000 | .000 | .000 | .000 | .012 | .000 | .000 |
| EE\_4 | .000 | .000 | .000 | .000 | .000 | .000 | .000 | .000 | .000 | .000 | .011 | .000 | .000 |
| EE\_3 | .000 | .000 | .000 | .000 | .000 | .000 | .000 | .000 | .000 | .000 | .011 | .000 | .000 |
| EE\_2 | .000 | .000 | .000 | .000 | .000 | .000 | .000 | .000 | .000 | .000 | .012 | .000 | .000 |
| EE\_1 | .000 | .000 | .000 | .000 | .000 | .000 | .000 | .000 | .000 | .000 | .012 | .000 | .000 |
| ESE\_10 | .000 | .000 | .000 | .000 | .000 | .000 | .000 | .000 | .000 | .000 | .000 | .013 | .000 |
| ESE\_9 | .000 | .000 | .000 | .000 | .000 | .000 | .000 | .000 | .000 | .000 | .000 | .012 | .000 |
| ESE\_8 | .000 | .000 | .000 | .000 | .000 | .000 | .000 | .000 | .000 | .000 | .000 | .012 | .000 |
| ESE\_7 | .000 | .000 | .000 | .000 | .000 | .000 | .000 | .000 | .000 | .000 | .000 | .011 | .000 |
| ESE\_6 | .000 | .000 | .000 | .000 | .000 | .000 | .000 | .000 | .000 | .000 | .000 | .000 | .011 |
| ESE\_5 | .000 | .000 | .000 | .000 | .000 | .000 | .000 | .000 | .000 | .000 | .000 | .000 | .011 |
| ESE\_4 | .000 | .000 | .000 | .000 | .000 | .000 | .000 | .000 | .000 | .000 | .000 | .000 | .011 |
| ESE\_3 | .000 | .000 | .000 | .000 | .000 | .000 | .000 | .000 | .000 | .000 | .000 | .000 | .012 |
| ESE\_2 | .000 | .000 | .000 | .000 | .000 | .000 | .000 | .000 | .000 | .000 | .000 | .000 | .012 |
| ESE\_1 | .000 | .000 | .000 | .000 | .000 | .000 | .000 | .000 | .000 | .000 | .000 | .000 | .012 |
| EOE\_9 | .000 | .000 | .000 | .000 | .011 | .000 | .000 | .000 | .000 | .000 | .000 | .000 | .000 |
| EOE\_8 | .000 | .000 | .000 | .000 | .011 | .000 | .000 | .000 | .000 | .000 | .000 | .000 | .000 |
| EOE\_7 | .000 | .000 | .000 | .000 | .010 | .000 | .000 | .000 | .000 | .000 | .000 | .000 | .000 |
| EOE\_6 | .000 | .000 | .000 | .000 | .011 | .000 | .000 | .000 | .000 | .000 | .000 | .000 | .000 |
| EOE\_5 | .000 | .000 | .000 | .000 | .010 | .000 | .000 | .000 | .000 | .000 | .000 | .000 | .000 |
| EOE\_4 | .000 | .000 | .000 | .000 | .011 | .000 | .000 | .000 | .000 | .000 | .000 | .000 | .000 |
| EOE\_3 | .000 | .000 | .000 | .000 | .011 | .000 | .000 | .000 | .000 | .000 | .000 | .000 | .000 |
| EOE\_2 | .000 | .000 | .000 | .000 | .011 | .000 | .000 | .000 | .000 | .000 | .000 | .000 | .000 |
| EOE\_1 | .000 | .000 | .000 | .000 | .011 | .000 | .000 | .000 | .000 | .000 | .000 | .000 | .000 |
| AISE\_6 | .000 | .000 | .000 | .011 | .000 | .000 | .000 | .000 | .000 | .000 | .000 | .000 | .000 |
| AISE\_5 | .000 | .000 | .000 | .010 | .000 | .000 | .000 | .000 | .000 | .000 | .000 | .000 | .000 |
| AISE\_4 | .000 | .000 | .000 | .011 | .000 | .000 | .000 | .000 | .000 | .000 | .000 | .000 | .000 |
| AISE\_3 | .000 | .000 | .000 | .011 | .000 | .000 | .000 | .000 | .000 | .000 | .000 | .000 | .000 |
| AISE\_2 | .000 | .000 | .000 | .010 | .000 | .000 | .000 | .000 | .000 | .000 | .000 | .000 | .000 |
| AISE\_1 | .000 | .000 | .000 | .011 | .000 | .000 | .000 | .000 | .000 | .000 | .000 | .000 | .000 |

## Indirect Effects - Standard Errors (Group number 1 - Default model)

|  | Grade | Age | Gender | AISE | EOE | ESE | EE | Pa | Vc | Fs | Vp | Ss | Sm |
| --- | --- | --- | --- | --- | --- | --- | --- | --- | --- | --- | --- | --- | --- |
| EOE | .000 | .000 | .000 | .000 | .000 | .000 | .000 | .000 | .000 | .000 | .000 | .000 | .000 |
| ESE | .003 | .003 | .005 | .010 | .000 | .000 | .000 | .000 | .000 | .000 | .000 | .000 | .000 |
| EE | .004 | .004 | .007 | .013 | .011 | .000 | .000 | .000 | .000 | .000 | .000 | .000 | .000 |
| Pa | .011 | .010 | .019 | .015 | .023 | .037 | .000 | .000 | .000 | .000 | .000 | .000 | .000 |
| Vc | .011 | .011 | .020 | .016 | .025 | .040 | .000 | .000 | .000 | .000 | .000 | .000 | .000 |
| Fs | .011 | .010 | .020 | .016 | .024 | .040 | .000 | .000 | .000 | .000 | .000 | .000 | .000 |
| Vp | .011 | .010 | .019 | .016 | .023 | .038 | .000 | .000 | .000 | .000 | .000 | .000 | .000 |
| Ss | .011 | .011 | .020 | .017 | .025 | .000 | .000 | .000 | .000 | .000 | .000 | .000 | .000 |
| Sm | .011 | .011 | .021 | .017 | .026 | .000 | .000 | .000 | .000 | .000 | .000 | .000 | .000 |
| EE\_20 | .010 | .010 | .019 | .015 | .023 | .037 | .041 | .000 | .000 | .000 | .000 | .000 | .000 |
| EE\_19 | .010 | .010 | .018 | .015 | .023 | .036 | .040 | .000 | .000 | .000 | .000 | .000 | .000 |
| EE\_18 | .011 | .010 | .019 | .015 | .023 | .037 | .043 | .000 | .000 | .000 | .000 | .000 | .000 |
| EE\_17 | .011 | .010 | .020 | .016 | .024 | .039 | .044 | .000 | .000 | .000 | .000 | .000 | .000 |
| EE\_16 | .011 | .011 | .020 | .016 | .025 | .039 | .044 | .000 | .000 | .000 | .000 | .000 | .000 |
| EE\_15 | .011 | .011 | .020 | .016 | .024 | .040 | .044 | .000 | .000 | .000 | .000 | .000 | .000 |
| EE\_14 | .011 | .010 | .020 | .016 | .024 | .039 | .042 | .000 | .000 | .000 | .000 | .000 | .000 |
| EE\_13 | .011 | .011 | .020 | .016 | .025 | .040 | .045 | .000 | .000 | .000 | .000 | .000 | .000 |
| EE\_12 | .011 | .010 | .020 | .016 | .024 | .039 | .044 | .000 | .000 | .000 | .000 | .000 | .000 |
| EE\_11 | .011 | .011 | .020 | .016 | .024 | .040 | .044 | .000 | .000 | .000 | .000 | .000 | .000 |
| EE\_10 | .011 | .011 | .020 | .016 | .024 | .040 | .045 | .000 | .000 | .000 | .000 | .000 | .000 |
| EE\_9 | .011 | .011 | .020 | .016 | .024 | .040 | .044 | .000 | .000 | .000 | .000 | .000 | .000 |
| EE\_8 | .011 | .010 | .019 | .016 | .024 | .038 | .044 | .000 | .000 | .000 | .000 | .000 | .000 |
| EE\_7 | .011 | .010 | .020 | .016 | .024 | .040 | .044 | .000 | .000 | .000 | .000 | .000 | .000 |
| EE\_6 | .011 | .011 | .020 | .016 | .024 | .039 | .032 | .000 | .000 | .000 | .000 | .000 | .000 |
| EE\_5 | .011 | .010 | .020 | .016 | .024 | .039 | .032 | .000 | .000 | .000 | .000 | .000 | .000 |
| EE\_4 | .011 | .010 | .020 | .016 | .024 | .038 | .032 | .000 | .000 | .000 | .000 | .000 | .000 |
| EE\_3 | .011 | .011 | .020 | .016 | .024 | .040 | .030 | .000 | .000 | .000 | .000 | .000 | .000 |
| EE\_2 | .011 | .010 | .019 | .016 | .024 | .039 | .031 | .000 | .000 | .000 | .000 | .000 | .000 |
| EE\_1 | .011 | .010 | .019 | .016 | .023 | .038 | .000 | .000 | .000 | .000 | .000 | .000 | .000 |
| ESE\_10 | .011 | .011 | .020 | .018 | .024 | .051 | .000 | .000 | .000 | .000 | .000 | .000 | .000 |
| ESE\_9 | .011 | .010 | .019 | .016 | .023 | .048 | .000 | .000 | .000 | .000 | .000 | .000 | .000 |
| ESE\_8 | .011 | .011 | .021 | .017 | .025 | .051 | .000 | .000 | .000 | .000 | .000 | .000 | .000 |
| ESE\_7 | .011 | .011 | .020 | .017 | .025 | .050 | .000 | .000 | .000 | .000 | .000 | .000 | .000 |
| ESE\_6 | .012 | .011 | .022 | .017 | .026 | .031 | .000 | .000 | .000 | .000 | .000 | .000 | .000 |
| ESE\_5 | .012 | .012 | .022 | .018 | .026 | .032 | .000 | .000 | .000 | .000 | .000 | .000 | .000 |
| ESE\_4 | .012 | .012 | .022 | .017 | .027 | .031 | .000 | .000 | .000 | .000 | .000 | .000 | .000 |
| ESE\_3 | .011 | .011 | .021 | .017 | .025 | .031 | .000 | .000 | .000 | .000 | .000 | .000 | .000 |
| ESE\_2 | .012 | .011 | .022 | .017 | .026 | .031 | .000 | .000 | .000 | .000 | .000 | .000 | .000 |
| ESE\_1 | .011 | .011 | .021 | .017 | .026 | .000 | .000 | .000 | .000 | .000 | .000 | .000 | .000 |
| EOE\_9 | .011 | .011 | .021 | .017 | .000 | .000 | .000 | .000 | .000 | .000 | .000 | .000 | .000 |
| EOE\_8 | .012 | .012 | .021 | .017 | .000 | .000 | .000 | .000 | .000 | .000 | .000 | .000 | .000 |
| EOE\_7 | .012 | .012 | .021 | .017 | .000 | .000 | .000 | .000 | .000 | .000 | .000 | .000 | .000 |
| EOE\_6 | .011 | .011 | .020 | .016 | .000 | .000 | .000 | .000 | .000 | .000 | .000 | .000 | .000 |
| EOE\_5 | .012 | .012 | .021 | .017 | .000 | .000 | .000 | .000 | .000 | .000 | .000 | .000 | .000 |
| EOE\_4 | .011 | .011 | .020 | .017 | .000 | .000 | .000 | .000 | .000 | .000 | .000 | .000 | .000 |
| EOE\_3 | .011 | .011 | .021 | .017 | .000 | .000 | .000 | .000 | .000 | .000 | .000 | .000 | .000 |
| EOE\_2 | .011 | .012 | .021 | .017 | .000 | .000 | .000 | .000 | .000 | .000 | .000 | .000 | .000 |
| EOE\_1 | .011 | .011 | .021 | .017 | .000 | .000 | .000 | .000 | .000 | .000 | .000 | .000 | .000 |
| AISE\_6 | .000 | .000 | .000 | .000 | .000 | .000 | .000 | .000 | .000 | .000 | .000 | .000 | .000 |
| AISE\_5 | .000 | .000 | .000 | .000 | .000 | .000 | .000 | .000 | .000 | .000 | .000 | .000 | .000 |
| AISE\_4 | .000 | .000 | .000 | .000 | .000 | .000 | .000 | .000 | .000 | .000 | .000 | .000 | .000 |
| AISE\_3 | .000 | .000 | .000 | .000 | .000 | .000 | .000 | .000 | .000 | .000 | .000 | .000 | .000 |
| AISE\_2 | .000 | .000 | .000 | .000 | .000 | .000 | .000 | .000 | .000 | .000 | .000 | .000 | .000 |
| AISE\_1 | .000 | .000 | .000 | .000 | .000 | .000 | .000 | .000 | .000 | .000 | .000 | .000 | .000 |

## Standardized Indirect Effects - Standard Errors (Group number 1 - Default model)

|  | Grade | Age | Gender | AISE | EOE | ESE | EE | Pa | Vc | Fs | Vp | Ss | Sm |
| --- | --- | --- | --- | --- | --- | --- | --- | --- | --- | --- | --- | --- | --- |
| EOE | .000 | .000 | .000 | .000 | .000 | .000 | .000 | .000 | .000 | .000 | .000 | .000 | .000 |
| ESE | .006 | .006 | .006 | .016 | .000 | .000 | .000 | .000 | .000 | .000 | .000 | .000 | .000 |
| EE | .007 | .008 | .008 | .021 | .012 | .000 | .000 | .000 | .000 | .000 | .000 | .000 | .000 |
| Pa | .017 | .016 | .017 | .017 | .021 | .029 | .000 | .000 | .000 | .000 | .000 | .000 | .000 |
| Vc | .017 | .017 | .017 | .017 | .021 | .029 | .000 | .000 | .000 | .000 | .000 | .000 | .000 |
| Fs | .017 | .017 | .017 | .017 | .021 | .029 | .000 | .000 | .000 | .000 | .000 | .000 | .000 |
| Vp | .017 | .016 | .017 | .017 | .021 | .029 | .000 | .000 | .000 | .000 | .000 | .000 | .000 |
| Ss | .017 | .018 | .018 | .019 | .021 | .000 | .000 | .000 | .000 | .000 | .000 | .000 | .000 |
| Sm | .018 | .018 | .019 | .018 | .023 | .000 | .000 | .000 | .000 | .000 | .000 | .000 | .000 |
| EE\_20 | .012 | .012 | .012 | .014 | .016 | .021 | .015 | .000 | .000 | .000 | .000 | .000 | .000 |
| EE\_19 | .012 | .012 | .012 | .014 | .016 | .021 | .015 | .000 | .000 | .000 | .000 | .000 | .000 |
| EE\_18 | .012 | .012 | .012 | .014 | .016 | .021 | .015 | .000 | .000 | .000 | .000 | .000 | .000 |
| EE\_17 | .013 | .012 | .013 | .014 | .016 | .022 | .013 | .000 | .000 | .000 | .000 | .000 | .000 |
| EE\_16 | .013 | .012 | .013 | .014 | .016 | .022 | .014 | .000 | .000 | .000 | .000 | .000 | .000 |
| EE\_15 | .013 | .013 | .013 | .014 | .016 | .022 | .013 | .000 | .000 | .000 | .000 | .000 | .000 |
| EE\_14 | .013 | .012 | .013 | .014 | .016 | .022 | .013 | .000 | .000 | .000 | .000 | .000 | .000 |
| EE\_13 | .013 | .013 | .013 | .014 | .016 | .022 | .013 | .000 | .000 | .000 | .000 | .000 | .000 |
| EE\_12 | .012 | .012 | .012 | .014 | .016 | .021 | .014 | .000 | .000 | .000 | .000 | .000 | .000 |
| EE\_11 | .012 | .012 | .012 | .014 | .016 | .021 | .014 | .000 | .000 | .000 | .000 | .000 | .000 |
| EE\_10 | .012 | .012 | .012 | .014 | .016 | .022 | .013 | .000 | .000 | .000 | .000 | .000 | .000 |
| EE\_9 | .012 | .012 | .013 | .014 | .016 | .022 | .014 | .000 | .000 | .000 | .000 | .000 | .000 |
| EE\_8 | .012 | .012 | .012 | .014 | .015 | .021 | .014 | .000 | .000 | .000 | .000 | .000 | .000 |
| EE\_7 | .012 | .012 | .012 | .014 | .016 | .021 | .013 | .000 | .000 | .000 | .000 | .000 | .000 |
| EE\_6 | .012 | .012 | .012 | .014 | .016 | .021 | .014 | .000 | .000 | .000 | .000 | .000 | .000 |
| EE\_5 | .012 | .012 | .012 | .014 | .015 | .021 | .014 | .000 | .000 | .000 | .000 | .000 | .000 |
| EE\_4 | .012 | .012 | .012 | .014 | .015 | .021 | .013 | .000 | .000 | .000 | .000 | .000 | .000 |
| EE\_3 | .012 | .012 | .013 | .014 | .016 | .021 | .014 | .000 | .000 | .000 | .000 | .000 | .000 |
| EE\_2 | .012 | .012 | .012 | .014 | .015 | .021 | .014 | .000 | .000 | .000 | .000 | .000 | .000 |
| EE\_1 | .012 | .012 | .012 | .014 | .015 | .021 | .014 | .000 | .000 | .000 | .000 | .000 | .000 |
| ESE\_10 | .013 | .013 | .013 | .016 | .016 | .017 | .000 | .000 | .000 | .000 | .000 | .000 | .000 |
| ESE\_9 | .012 | .013 | .013 | .015 | .016 | .016 | .000 | .000 | .000 | .000 | .000 | .000 | .000 |
| ESE\_8 | .013 | .013 | .013 | .016 | .016 | .017 | .000 | .000 | .000 | .000 | .000 | .000 | .000 |
| ESE\_7 | .013 | .013 | .013 | .015 | .016 | .016 | .000 | .000 | .000 | .000 | .000 | .000 | .000 |
| ESE\_6 | .013 | .013 | .013 | .015 | .017 | .016 | .000 | .000 | .000 | .000 | .000 | .000 | .000 |
| ESE\_5 | .013 | .014 | .014 | .015 | .017 | .016 | .000 | .000 | .000 | .000 | .000 | .000 | .000 |
| ESE\_4 | .013 | .014 | .014 | .015 | .017 | .016 | .000 | .000 | .000 | .000 | .000 | .000 | .000 |
| ESE\_3 | .013 | .013 | .013 | .015 | .017 | .016 | .000 | .000 | .000 | .000 | .000 | .000 | .000 |
| ESE\_2 | .013 | .013 | .014 | .015 | .017 | .017 | .000 | .000 | .000 | .000 | .000 | .000 | .000 |
| ESE\_1 | .013 | .013 | .013 | .015 | .017 | .016 | .000 | .000 | .000 | .000 | .000 | .000 | .000 |
| EOE\_9 | .013 | .014 | .014 | .015 | .000 | .000 | .000 | .000 | .000 | .000 | .000 | .000 | .000 |
| EOE\_8 | .014 | .014 | .014 | .015 | .000 | .000 | .000 | .000 | .000 | .000 | .000 | .000 | .000 |
| EOE\_7 | .014 | .015 | .014 | .015 | .000 | .000 | .000 | .000 | .000 | .000 | .000 | .000 | .000 |
| EOE\_6 | .013 | .014 | .014 | .014 | .000 | .000 | .000 | .000 | .000 | .000 | .000 | .000 | .000 |
| EOE\_5 | .014 | .014 | .014 | .015 | .000 | .000 | .000 | .000 | .000 | .000 | .000 | .000 | .000 |
| EOE\_4 | .013 | .014 | .014 | .015 | .000 | .000 | .000 | .000 | .000 | .000 | .000 | .000 | .000 |
| EOE\_3 | .013 | .014 | .014 | .014 | .000 | .000 | .000 | .000 | .000 | .000 | .000 | .000 | .000 |
| EOE\_2 | .014 | .014 | .014 | .015 | .000 | .000 | .000 | .000 | .000 | .000 | .000 | .000 | .000 |
| EOE\_1 | .013 | .014 | .014 | .015 | .000 | .000 | .000 | .000 | .000 | .000 | .000 | .000 | .000 |
| AISE\_6 | .000 | .000 | .000 | .000 | .000 | .000 | .000 | .000 | .000 | .000 | .000 | .000 | .000 |
| AISE\_5 | .000 | .000 | .000 | .000 | .000 | .000 | .000 | .000 | .000 | .000 | .000 | .000 | .000 |
| AISE\_4 | .000 | .000 | .000 | .000 | .000 | .000 | .000 | .000 | .000 | .000 | .000 | .000 | .000 |
| AISE\_3 | .000 | .000 | .000 | .000 | .000 | .000 | .000 | .000 | .000 | .000 | .000 | .000 | .000 |
| AISE\_2 | .000 | .000 | .000 | .000 | .000 | .000 | .000 | .000 | .000 | .000 | .000 | .000 | .000 |
| AISE\_1 | .000 | .000 | .000 | .000 | .000 | .000 | .000 | .000 | .000 | .000 | .000 | .000 | .000 |

## Bootstrap Confidence (Group number 1 - Default model)

## Percentile method (Group number 1 - Default model)

## 95% confidence intervals (percentile method)

## Scalar Estimates (Group number 1 - Default model)

## Regression Weights: (Group number 1 - Default model)

| Parameter | | | Estimate | Lower | Upper | P |
| --- | --- | --- | --- | --- | --- | --- |
| EOE | <--- | AISE | .363 | .332 | .396 | .000 |
| EOE | <--- | Gender | -.037 | -.078 | .004 | .075 |
| EOE | <--- | Age | .020 | -.002 | .042 | .084 |
| EOE | <--- | Grade | .016 | -.006 | .039 | .148 |
| ESE | <--- | AISE | .242 | .208 | .277 | .000 |
| ESE | <--- | EOE | .257 | .208 | .309 | .000 |
| ESE | <--- | Gender | -.045 | -.085 | -.005 | .027 |
| ESE | <--- | Age | .000 | -.021 | .020 | .971 |
| ESE | <--- | Grade | .009 | -.013 | .031 | .396 |
| EE | <--- | ESE | .232 | .160 | .308 | .000 |
| EE | <--- | EOE | .176 | .129 | .224 | .000 |
| EE | <--- | AISE | .142 | .106 | .179 | .000 |
| EE | <--- | Gender | -.049 | -.084 | -.013 | .007 |
| EE | <--- | Age | .016 | -.003 | .035 | .095 |
| EE | <--- | Grade | .014 | -.006 | .034 | .170 |
| Sm | <--- | ESE | 1.000 | 1.000 | 1.000 | ... |
| Ss | <--- | ESE | .993 | .901 | 1.095 | .000 |
| Vp | <--- | EE | 1.000 | 1.000 | 1.000 | ... |
| Fs | <--- | EE | 1.034 | .954 | 1.124 | .000 |
| Vc | <--- | EE | 1.062 | .980 | 1.156 | .000 |
| Pa | <--- | EE | .978 | .896 | 1.067 | .000 |
| AISE\_1 | <--- | AISE | 1.000 | 1.000 | 1.000 | ... |
| AISE\_2 | <--- | AISE | .983 | .932 | 1.036 | .000 |
| AISE\_3 | <--- | AISE | .959 | .909 | 1.012 | .000 |
| AISE\_4 | <--- | AISE | .991 | .940 | 1.046 | .000 |
| AISE\_5 | <--- | AISE | .955 | .905 | 1.009 | .000 |
| AISE\_6 | <--- | AISE | .999 | .948 | 1.052 | .000 |
| EOE\_1 | <--- | EOE | 1.000 | 1.000 | 1.000 | ... |
| EOE\_2 | <--- | EOE | 1.014 | .960 | 1.070 | .000 |
| EOE\_3 | <--- | EOE | 1.002 | .949 | 1.059 | .000 |
| EOE\_4 | <--- | EOE | .993 | .938 | 1.051 | .000 |
| EOE\_5 | <--- | EOE | 1.032 | .975 | 1.093 | .000 |
| EOE\_6 | <--- | EOE | .992 | .937 | 1.051 | .000 |
| EOE\_7 | <--- | EOE | 1.030 | .974 | 1.092 | .000 |
| EOE\_8 | <--- | EOE | 1.024 | .968 | 1.085 | .000 |
| EOE\_9 | <--- | EOE | 1.001 | .943 | 1.061 | .000 |
| ESE\_1 | <--- | Sm | 1.000 | 1.000 | 1.000 | ... |
| ESE\_2 | <--- | Sm | 1.028 | .969 | 1.091 | .000 |
| ESE\_3 | <--- | Sm | .982 | .923 | 1.046 | .000 |
| ESE\_4 | <--- | Sm | 1.047 | .987 | 1.111 | .000 |
| ESE\_5 | <--- | Sm | 1.033 | .973 | 1.097 | .000 |
| ESE\_6 | <--- | Sm | 1.028 | .968 | 1.092 | .000 |
| ESE\_7 | <--- | Ss | 1.000 | 1.000 | 1.000 | ... |
| ESE\_8 | <--- | Ss | 1.005 | .950 | 1.063 | .000 |
| ESE\_9 | <--- | Ss | .947 | .891 | 1.004 | .000 |
| ESE\_10 | <--- | Ss | .981 | .925 | 1.042 | .000 |
| EE\_1 | <--- | Vp | 1.000 | 1.000 | 1.000 | ... |
| EE\_2 | <--- | Vp | 1.020 | .962 | 1.084 | .000 |
| EE\_3 | <--- | Vp | 1.049 | .995 | 1.110 | .000 |
| EE\_4 | <--- | Vp | 1.022 | .963 | 1.088 | .000 |
| EE\_5 | <--- | Vp | 1.027 | .967 | 1.090 | .000 |
| EE\_6 | <--- | Vp | 1.037 | .979 | 1.102 | .000 |
| EE\_7 | <--- | Fs | 1.000 | 1.000 | 1.000 | ... |
| EE\_8 | <--- | Fs | .972 | .916 | 1.035 | .000 |
| EE\_9 | <--- | Fs | 1.007 | .952 | 1.065 | .000 |
| EE\_10 | <--- | Fs | 1.008 | .949 | 1.068 | .000 |
| EE\_11 | <--- | Fs | 1.003 | .945 | 1.063 | .000 |
| EE\_12 | <--- | Fs | .994 | .936 | 1.056 | .000 |
| EE\_13 | <--- | Vc | 1.000 | 1.000 | 1.000 | ... |
| EE\_14 | <--- | Vc | .973 | .921 | 1.028 | .000 |
| EE\_15 | <--- | Vc | .989 | .936 | 1.044 | .000 |
| EE\_16 | <--- | Vc | .977 | .925 | 1.033 | .000 |
| EE\_17 | <--- | Vc | .970 | .913 | 1.028 | .000 |
| EE\_18 | <--- | Pa | 1.000 | 1.000 | 1.000 | ... |
| EE\_19 | <--- | Pa | .973 | .916 | 1.036 | .000 |
| EE\_20 | <--- | Pa | .990 | .930 | 1.053 | .000 |

## Standardized Regression Weights: (Group number 1 - Default model)

| Parameter | | | Estimate | Lower | Upper | P |
| --- | --- | --- | --- | --- | --- | --- |
| EOE | <--- | AISE | .547 | .513 | .581 | .000 |
| EOE | <--- | Gender | -.035 | -.073 | .003 | .075 |
| EOE | <--- | Age | .034 | -.003 | .073 | .084 |
| EOE | <--- | Grade | .027 | -.011 | .064 | .148 |
| ESE | <--- | AISE | .428 | .374 | .480 | .000 |
| ESE | <--- | EOE | .301 | .248 | .355 | .000 |
| ESE | <--- | Gender | -.049 | -.093 | -.006 | .027 |
| ESE | <--- | Age | -.001 | -.042 | .041 | .971 |
| ESE | <--- | Grade | .018 | -.024 | .060 | .396 |
| EE | <--- | ESE | .231 | .163 | .300 | .000 |
| EE | <--- | EOE | .206 | .153 | .260 | .000 |
| EE | <--- | AISE | .249 | .188 | .309 | .000 |
| EE | <--- | Gender | -.054 | -.092 | -.014 | .007 |
| EE | <--- | Age | .032 | -.006 | .071 | .095 |
| EE | <--- | Grade | .027 | -.012 | .066 | .170 |
| Sm | <--- | ESE | .811 | .777 | .845 | .000 |
| Ss | <--- | ESE | .784 | .748 | .819 | .000 |
| Vp | <--- | EE | .807 | .783 | .830 | .000 |
| Fs | <--- | EE | .813 | .789 | .836 | .000 |
| Vc | <--- | EE | .824 | .801 | .846 | .000 |
| Pa | <--- | EE | .803 | .775 | .831 | .000 |
| AISE\_1 | <--- | AISE | .721 | .698 | .743 | .000 |
| AISE\_2 | <--- | AISE | .757 | .736 | .776 | .000 |
| AISE\_3 | <--- | AISE | .736 | .714 | .757 | .000 |
| AISE\_4 | <--- | AISE | .748 | .726 | .768 | .000 |
| AISE\_5 | <--- | AISE | .760 | .739 | .780 | .000 |
| AISE\_6 | <--- | AISE | .752 | .731 | .773 | .000 |
| EOE\_1 | <--- | EOE | .720 | .698 | .741 | .000 |
| EOE\_2 | <--- | EOE | .727 | .705 | .749 | .000 |
| EOE\_3 | <--- | EOE | .721 | .699 | .742 | .000 |
| EOE\_4 | <--- | EOE | .724 | .703 | .744 | .000 |
| EOE\_5 | <--- | EOE | .738 | .717 | .758 | .000 |
| EOE\_6 | <--- | EOE | .716 | .694 | .738 | .000 |
| EOE\_7 | <--- | EOE | .745 | .726 | .765 | .000 |
| EOE\_8 | <--- | EOE | .728 | .707 | .749 | .000 |
| EOE\_9 | <--- | EOE | .718 | .697 | .740 | .000 |
| ESE\_1 | <--- | Sm | .711 | .688 | .735 | .000 |
| ESE\_2 | <--- | Sm | .733 | .710 | .757 | .000 |
| ESE\_3 | <--- | Sm | .713 | .688 | .736 | .000 |
| ESE\_4 | <--- | Sm | .739 | .716 | .760 | .000 |
| ESE\_5 | <--- | Sm | .739 | .717 | .760 | .000 |
| ESE\_6 | <--- | Sm | .721 | .697 | .743 | .000 |
| ESE\_7 | <--- | Ss | .744 | .722 | .766 | .000 |
| ESE\_8 | <--- | Ss | .748 | .724 | .772 | .000 |
| ESE\_9 | <--- | Ss | .716 | .692 | .740 | .000 |
| ESE\_10 | <--- | Ss | .735 | .708 | .758 | .000 |
| EE\_1 | <--- | Vp | .719 | .694 | .742 | .000 |
| EE\_2 | <--- | Vp | .722 | .698 | .744 | .000 |
| EE\_3 | <--- | Vp | .744 | .722 | .764 | .000 |
| EE\_4 | <--- | Vp | .732 | .710 | .753 | .000 |
| EE\_5 | <--- | Vp | .726 | .703 | .747 | .000 |
| EE\_6 | <--- | Vp | .740 | .718 | .761 | .000 |
| EE\_7 | <--- | Fs | .729 | .707 | .750 | .000 |
| EE\_8 | <--- | Fs | .715 | .691 | .737 | .000 |
| EE\_9 | <--- | Fs | .739 | .717 | .761 | .000 |
| EE\_10 | <--- | Fs | .737 | .715 | .757 | .000 |
| EE\_11 | <--- | Fs | .736 | .713 | .758 | .000 |
| EE\_12 | <--- | Fs | .735 | .712 | .756 | .000 |
| EE\_13 | <--- | Vc | .750 | .728 | .771 | .000 |
| EE\_14 | <--- | Vc | .734 | .711 | .755 | .000 |
| EE\_15 | <--- | Vc | .749 | .726 | .769 | .000 |
| EE\_16 | <--- | Vc | .735 | .713 | .757 | .000 |
| EE\_17 | <--- | Vc | .738 | .714 | .760 | .000 |
| EE\_18 | <--- | Pa | .738 | .711 | .762 | .000 |
| EE\_19 | <--- | Pa | .715 | .688 | .740 | .000 |
| EE\_20 | <--- | Pa | .723 | .696 | .748 | .000 |

## Covariances: (Group number 1 - Default model)

| Parameter | | | Estimate | Lower | Upper | P |
| --- | --- | --- | --- | --- | --- | --- |
| Gender | <--> | Age | .006 | -.013 | .023 | .560 |
| Gender | <--> | Grade | -.006 | -.023 | .012 | .539 |
| Age | <--> | Grade | .017 | -.016 | .049 | .308 |
| AISE | <--> | Gender | -.017 | -.035 | .000 | .046 |
| AISE | <--> | Age | .070 | .040 | .101 | .000 |
| AISE | <--> | Grade | .015 | -.014 | .045 | .308 |

## Correlations: (Group number 1 - Default model)

| Parameter | | | Estimate | Lower | Upper | P |
| --- | --- | --- | --- | --- | --- | --- |
| Gender | <--> | Age | .012 | -.029 | .052 | .560 |
| Gender | <--> | Grade | -.013 | -.054 | .029 | .539 |
| Age | <--> | Grade | .021 | -.020 | .062 | .309 |
| AISE | <--> | Gender | -.045 | -.090 | -.001 | .046 |
| AISE | <--> | Age | .098 | .056 | .140 | .000 |
| AISE | <--> | Grade | .023 | -.021 | .066 | .308 |

## Variances: (Group number 1 - Default model)

| Parameter | | | Estimate | Lower | Upper | P |
| --- | --- | --- | --- | --- | --- | --- |
| AISE |  |  | .623 | .567 | .680 | .000 |
| Gender |  |  | .242 | .238 | .245 | .000 |
| Age |  |  | .816 | .778 | .854 | .000 |
| Grade |  |  | .743 | .724 | .761 | .000 |
| e52 |  |  | .190 | .170 | .209 | .000 |
| e53 |  |  | .115 | .098 | .133 | .000 |
| e54 |  |  | .131 | .113 | .148 | .000 |
| e46 |  |  | .103 | .084 | .123 | .000 |
| e47 |  |  | .123 | .104 | .143 | .000 |
| e48 |  |  | .108 | .093 | .122 | .000 |
| e49 |  |  | .110 | .096 | .125 | .000 |
| e50 |  |  | .107 | .093 | .123 | .000 |
| e51 |  |  | .106 | .090 | .122 | .000 |
| e1 |  |  | .576 | .537 | .614 | .000 |
| e2 |  |  | .449 | .419 | .479 | .000 |
| e3 |  |  | .485 | .454 | .518 | .000 |
| e4 |  |  | .483 | .450 | .517 | .000 |
| e5 |  |  | .415 | .386 | .443 | .000 |
| e6 |  |  | .477 | .445 | .510 | .000 |
| e7 |  |  | .255 | .240 | .272 | .000 |
| e8 |  |  | .251 | .235 | .267 | .000 |
| e9 |  |  | .255 | .239 | .270 | .000 |
| e10 |  |  | .246 | .231 | .261 | .000 |
| e11 |  |  | .245 | .229 | .260 | .000 |
| e12 |  |  | .256 | .241 | .272 | .000 |
| e13 |  |  | .233 | .218 | .247 | .000 |
| e14 |  |  | .256 | .240 | .271 | .000 |
| e15 |  |  | .258 | .242 | .274 | .000 |
| e16 |  |  | .296 | .275 | .315 | .000 |
| e17 |  |  | .275 | .255 | .294 | .000 |
| e18 |  |  | .283 | .264 | .302 | .000 |
| e19 |  |  | .275 | .258 | .293 | .000 |
| e20 |  |  | .269 | .251 | .286 | .000 |
| e21 |  |  | .296 | .277 | .316 | .000 |
| e22 |  |  | .257 | .238 | .275 | .000 |
| e23 |  |  | .255 | .235 | .275 | .000 |
| e24 |  |  | .272 | .253 | .290 | .000 |
| e25 |  |  | .262 | .243 | .282 | .000 |
| e26 |  |  | .288 | .269 | .307 | .000 |
| e27 |  |  | .296 | .276 | .315 | .000 |
| e28 |  |  | .275 | .256 | .293 | .000 |
| e29 |  |  | .280 | .261 | .298 | .000 |
| e30 |  |  | .293 | .272 | .314 | .000 |
| e31 |  |  | .274 | .256 | .293 | .000 |
| e32 |  |  | .287 | .269 | .306 | .000 |
| e33 |  |  | .294 | .275 | .312 | .000 |
| e34 |  |  | .273 | .255 | .292 | .000 |
| e35 |  |  | .278 | .260 | .296 | .000 |
| e36 |  |  | .277 | .258 | .296 | .000 |
| e37 |  |  | .273 | .254 | .292 | .000 |
| e38 |  |  | .260 | .242 | .278 | .000 |
| e39 |  |  | .271 | .252 | .289 | .000 |
| e40 |  |  | .256 | .239 | .274 | .000 |
| e41 |  |  | .271 | .252 | .289 | .000 |
| e42 |  |  | .262 | .244 | .281 | .000 |
| e43 |  |  | .250 | .230 | .269 | .000 |
| e44 |  |  | .270 | .250 | .291 | .000 |
| e45 |  |  | .266 | .245 | .287 | .000 |

## User-defined estimands: (Group number 1 - Default model)

| Parameter | | | Estimate | Lower | Upper | P |
| --- | --- | --- | --- | --- | --- | --- |
| MedIndirect1 |  |  | .099 | .067 | .133 | .000 |
| MedDirect1 |  |  | .249 | .188 | .309 | .000 |
| MedTotal1 |  |  | .348 | .298 | .399 | .000 |
| MedIndirect2 |  |  | .113 | .084 | .143 | .000 |
| MedDirect2 |  |  | .249 | .188 | .309 | .000 |
| MedTotal2 |  |  | .362 | .300 | .420 | .000 |
| MedIndirect3 |  |  | .250 | .208 | .292 | .000 |
| MedDirect3 |  |  | .249 | .188 | .309 | .000 |
| MedTotal3 |  |  | .499 | .462 | .537 | .000 |

## Matrices (Group number 1 - Default model)

## Total Effects (Group number 1 - Default model)

## Total Effects - Lower Bounds (PC) (Group number 1 - Default model)

|  | Grade | Age | Gender | AISE | EOE | ESE | EE | Pa | Vc | Fs | Vp | Ss | Sm |
| --- | --- | --- | --- | --- | --- | --- | --- | --- | --- | --- | --- | --- | --- |
| EOE | -.006 | -.002 | -.078 | .332 | .000 | .000 | .000 | .000 | .000 | .000 | .000 | .000 | .000 |
| ESE | -.009 | -.017 | -.096 | .302 | .208 | .000 | .000 | .000 | .000 | .000 | .000 | .000 | .000 |
| EE | -.001 | .001 | -.104 | .252 | .189 | .160 | .000 | .000 | .000 | .000 | .000 | .000 | .000 |
| Pa | -.001 | .001 | -.103 | .248 | .185 | .157 | .896 | .000 | .000 | .000 | .000 | .000 | .000 |
| Vc | -.001 | .001 | -.112 | .271 | .203 | .170 | .980 | .000 | .000 | .000 | .000 | .000 | .000 |
| Fs | -.001 | .001 | -.108 | .262 | .197 | .166 | .954 | .000 | .000 | .000 | .000 | .000 | .000 |
| Vp | -.001 | .001 | -.104 | .252 | .189 | .160 | 1.000 | .000 | .000 | .000 | .000 | .000 | .000 |
| Ss | -.009 | -.017 | -.094 | .301 | .208 | .901 | .000 | .000 | .000 | .000 | .000 | .000 | .000 |
| Sm | -.009 | -.017 | -.096 | .302 | .208 | 1.000 | .000 | .000 | .000 | .000 | .000 | .000 | .000 |
| EE\_20 | -.001 | .001 | -.102 | .246 | .184 | .155 | .891 | .930 | .000 | .000 | .000 | .000 | .000 |
| EE\_19 | -.001 | .001 | -.100 | .242 | .180 | .153 | .878 | .916 | .000 | .000 | .000 | .000 | .000 |
| EE\_18 | -.001 | .001 | -.103 | .248 | .185 | .157 | .896 | 1.000 | .000 | .000 | .000 | .000 | .000 |
| EE\_17 | -.001 | .001 | -.108 | .262 | .196 | .166 | .947 | .000 | .913 | .000 | .000 | .000 | .000 |
| EE\_16 | -.001 | .001 | -.108 | .265 | .197 | .166 | .956 | .000 | .925 | .000 | .000 | .000 | .000 |
| EE\_15 | -.001 | .001 | -.110 | .268 | .200 | .169 | .968 | .000 | .936 | .000 | .000 | .000 | .000 |
| EE\_14 | -.001 | .001 | -.108 | .263 | .197 | .166 | .953 | .000 | .921 | .000 | .000 | .000 | .000 |
| EE\_13 | -.001 | .001 | -.112 | .271 | .203 | .170 | .980 | .000 | 1.000 | .000 | .000 | .000 | .000 |
| EE\_12 | -.001 | .001 | -.107 | .261 | .195 | .165 | .946 | .000 | .000 | .936 | .000 | .000 | .000 |
| EE\_11 | -.001 | .001 | -.109 | .263 | .198 | .167 | .955 | .000 | .000 | .945 | .000 | .000 | .000 |
| EE\_10 | -.001 | .001 | -.109 | .264 | .198 | .168 | .958 | .000 | .000 | .949 | .000 | .000 | .000 |
| EE\_9 | -.001 | .001 | -.109 | .264 | .198 | .167 | .959 | .000 | .000 | .952 | .000 | .000 | .000 |
| EE\_8 | -.001 | .001 | -.105 | .254 | .191 | .161 | .924 | .000 | .000 | .916 | .000 | .000 | .000 |
| EE\_7 | -.001 | .001 | -.108 | .262 | .197 | .166 | .954 | .000 | .000 | 1.000 | .000 | .000 | .000 |
| EE\_6 | -.001 | .001 | -.109 | .263 | .197 | .167 | .979 | .000 | .000 | .000 | .979 | .000 | .000 |
| EE\_5 | -.001 | .001 | -.107 | .260 | .195 | .164 | .967 | .000 | .000 | .000 | .967 | .000 | .000 |
| EE\_4 | -.001 | .001 | -.107 | .260 | .195 | .164 | .963 | .000 | .000 | .000 | .963 | .000 | .000 |
| EE\_3 | -.001 | .001 | -.110 | .267 | .199 | .168 | .995 | .000 | .000 | .000 | .995 | .000 | .000 |
| EE\_2 | -.001 | .001 | -.107 | .259 | .193 | .165 | .962 | .000 | .000 | .000 | .962 | .000 | .000 |
| EE\_1 | -.001 | .001 | -.104 | .252 | .189 | .160 | 1.000 | .000 | .000 | .000 | 1.000 | .000 | .000 |
| ESE\_10 | -.008 | -.017 | -.092 | .293 | .204 | .879 | .000 | .000 | .000 | .000 | .000 | .925 | .000 |
| ESE\_9 | -.008 | -.016 | -.089 | .283 | .196 | .851 | .000 | .000 | .000 | .000 | .000 | .891 | .000 |
| ESE\_8 | -.009 | -.017 | -.095 | .302 | .208 | .905 | .000 | .000 | .000 | .000 | .000 | .950 | .000 |
| ESE\_7 | -.009 | -.017 | -.094 | .301 | .208 | .901 | .000 | .000 | .000 | .000 | .000 | 1.000 | .000 |
| ESE\_6 | -.009 | -.018 | -.099 | .312 | .214 | .968 | .000 | .000 | .000 | .000 | .000 | .000 | .968 |
| ESE\_5 | -.009 | -.017 | -.099 | .313 | .215 | .973 | .000 | .000 | .000 | .000 | .000 | .000 | .973 |
| ESE\_4 | -.009 | -.018 | -.100 | .318 | .218 | .987 | .000 | .000 | .000 | .000 | .000 | .000 | .987 |
| ESE\_3 | -.008 | -.016 | -.094 | .296 | .205 | .923 | .000 | .000 | .000 | .000 | .000 | .000 | .923 |
| ESE\_2 | -.009 | -.017 | -.099 | .311 | .213 | .969 | .000 | .000 | .000 | .000 | .000 | .000 | .969 |
| ESE\_1 | -.009 | -.017 | -.096 | .302 | .208 | 1.000 | .000 | .000 | .000 | .000 | .000 | .000 | 1.000 |
| EOE\_9 | -.007 | -.002 | -.079 | .331 | .943 | .000 | .000 | .000 | .000 | .000 | .000 | .000 | .000 |
| EOE\_8 | -.007 | -.002 | -.079 | .339 | .968 | .000 | .000 | .000 | .000 | .000 | .000 | .000 | .000 |
| EOE\_7 | -.007 | -.002 | -.080 | .341 | .974 | .000 | .000 | .000 | .000 | .000 | .000 | .000 | .000 |
| EOE\_6 | -.006 | -.002 | -.077 | .329 | .937 | .000 | .000 | .000 | .000 | .000 | .000 | .000 | .000 |
| EOE\_5 | -.007 | -.002 | -.080 | .343 | .975 | .000 | .000 | .000 | .000 | .000 | .000 | .000 | .000 |
| EOE\_4 | -.007 | -.002 | -.077 | .328 | .938 | .000 | .000 | .000 | .000 | .000 | .000 | .000 | .000 |
| EOE\_3 | -.007 | -.002 | -.078 | .333 | .949 | .000 | .000 | .000 | .000 | .000 | .000 | .000 | .000 |
| EOE\_2 | -.006 | -.002 | -.079 | .336 | .960 | .000 | .000 | .000 | .000 | .000 | .000 | .000 | .000 |
| EOE\_1 | -.006 | -.002 | -.078 | .332 | 1.000 | .000 | .000 | .000 | .000 | .000 | .000 | .000 | .000 |
| AISE\_6 | .000 | .000 | .000 | .948 | .000 | .000 | .000 | .000 | .000 | .000 | .000 | .000 | .000 |
| AISE\_5 | .000 | .000 | .000 | .905 | .000 | .000 | .000 | .000 | .000 | .000 | .000 | .000 | .000 |
| AISE\_4 | .000 | .000 | .000 | .940 | .000 | .000 | .000 | .000 | .000 | .000 | .000 | .000 | .000 |
| AISE\_3 | .000 | .000 | .000 | .909 | .000 | .000 | .000 | .000 | .000 | .000 | .000 | .000 | .000 |
| AISE\_2 | .000 | .000 | .000 | .932 | .000 | .000 | .000 | .000 | .000 | .000 | .000 | .000 | .000 |
| AISE\_1 | .000 | .000 | .000 | 1.000 | .000 | .000 | .000 | .000 | .000 | .000 | .000 | .000 | .000 |

## Total Effects - Upper Bounds (PC) (Group number 1 - Default model)

|  | Grade | Age | Gender | AISE | EOE | ESE | EE | Pa | Vc | Fs | Vp | Ss | Sm |
| --- | --- | --- | --- | --- | --- | --- | --- | --- | --- | --- | --- | --- | --- |
| EOE | .039 | .042 | .004 | .396 | .000 | .000 | .000 | .000 | .000 | .000 | .000 | .000 | .000 |
| ESE | .036 | .026 | -.013 | .369 | .309 | .000 | .000 | .000 | .000 | .000 | .000 | .000 | .000 |
| EE | .041 | .041 | -.031 | .315 | .283 | .308 | .000 | .000 | .000 | .000 | .000 | .000 | .000 |
| Pa | .041 | .040 | -.030 | .309 | .278 | .304 | 1.067 | .000 | .000 | .000 | .000 | .000 | .000 |
| Vc | .044 | .044 | -.032 | .333 | .300 | .328 | 1.156 | .000 | .000 | .000 | .000 | .000 | .000 |
| Fs | .042 | .042 | -.032 | .327 | .292 | .320 | 1.124 | .000 | .000 | .000 | .000 | .000 | .000 |
| Vp | .041 | .041 | -.031 | .315 | .283 | .308 | 1.000 | .000 | .000 | .000 | .000 | .000 | .000 |
| Ss | .036 | .026 | -.013 | .367 | .305 | 1.095 | .000 | .000 | .000 | .000 | .000 | .000 | .000 |
| Sm | .036 | .026 | -.013 | .369 | .309 | 1.000 | .000 | .000 | .000 | .000 | .000 | .000 | .000 |
| EE\_20 | .040 | .040 | -.029 | .305 | .276 | .299 | 1.051 | 1.053 | .000 | .000 | .000 | .000 | .000 |
| EE\_19 | .039 | .039 | -.029 | .301 | .271 | .295 | 1.033 | 1.036 | .000 | .000 | .000 | .000 | .000 |
| EE\_18 | .041 | .040 | -.030 | .309 | .278 | .304 | 1.067 | 1.000 | .000 | .000 | .000 | .000 | .000 |
| EE\_17 | .042 | .042 | -.031 | .323 | .291 | .318 | 1.121 | .000 | 1.028 | .000 | .000 | .000 | .000 |
| EE\_16 | .043 | .042 | -.032 | .326 | .293 | .322 | 1.128 | .000 | 1.033 | .000 | .000 | .000 | .000 |
| EE\_15 | .043 | .043 | -.032 | .330 | .297 | .324 | 1.139 | .000 | 1.044 | .000 | .000 | .000 | .000 |
| EE\_14 | .043 | .042 | -.031 | .326 | .291 | .318 | 1.120 | .000 | 1.028 | .000 | .000 | .000 | .000 |
| EE\_13 | .044 | .044 | -.032 | .333 | .300 | .328 | 1.156 | .000 | 1.000 | .000 | .000 | .000 | .000 |
| EE\_12 | .043 | .042 | -.032 | .324 | .291 | .317 | 1.118 | .000 | .000 | 1.056 | .000 | .000 | .000 |
| EE\_11 | .043 | .042 | -.032 | .327 | .293 | .322 | 1.127 | .000 | .000 | 1.063 | .000 | .000 | .000 |
| EE\_10 | .043 | .042 | -.032 | .328 | .294 | .323 | 1.134 | .000 | .000 | 1.068 | .000 | .000 | .000 |
| EE\_9 | .043 | .042 | -.032 | .328 | .294 | .322 | 1.133 | .000 | .000 | 1.065 | .000 | .000 | .000 |
| EE\_8 | .041 | .041 | -.030 | .317 | .284 | .311 | 1.096 | .000 | .000 | 1.035 | .000 | .000 | .000 |
| EE\_7 | .042 | .042 | -.032 | .327 | .292 | .320 | 1.124 | .000 | .000 | 1.000 | .000 | .000 | .000 |
| EE\_6 | .043 | .043 | -.032 | .326 | .293 | .322 | 1.102 | .000 | .000 | .000 | 1.102 | .000 | .000 |
| EE\_5 | .042 | .042 | -.031 | .323 | .289 | .317 | 1.090 | .000 | .000 | .000 | 1.090 | .000 | .000 |
| EE\_4 | .043 | .042 | -.031 | .322 | .287 | .316 | 1.088 | .000 | .000 | .000 | 1.088 | .000 | .000 |
| EE\_3 | .043 | .043 | -.032 | .329 | .295 | .324 | 1.110 | .000 | .000 | .000 | 1.110 | .000 | .000 |
| EE\_2 | .042 | .042 | -.031 | .322 | .289 | .316 | 1.084 | .000 | .000 | .000 | 1.084 | .000 | .000 |
| EE\_1 | .041 | .041 | -.031 | .315 | .283 | .308 | 1.000 | .000 | .000 | .000 | 1.000 | .000 | .000 |
| ESE\_10 | .035 | .026 | -.013 | .362 | .299 | 1.076 | .000 | .000 | .000 | .000 | .000 | 1.042 | .000 |
| ESE\_9 | .034 | .025 | -.013 | .348 | .290 | 1.040 | .000 | .000 | .000 | .000 | .000 | 1.004 | .000 |
| ESE\_8 | .036 | .026 | -.013 | .370 | .307 | 1.104 | .000 | .000 | .000 | .000 | .000 | 1.063 | .000 |
| ESE\_7 | .036 | .026 | -.013 | .367 | .305 | 1.095 | .000 | .000 | .000 | .000 | .000 | 1.000 | .000 |
| ESE\_6 | .037 | .027 | -.014 | .380 | .317 | 1.092 | .000 | .000 | .000 | .000 | .000 | .000 | 1.092 |
| ESE\_5 | .037 | .027 | -.014 | .382 | .319 | 1.097 | .000 | .000 | .000 | .000 | .000 | .000 | 1.097 |
| ESE\_4 | .038 | .028 | -.014 | .385 | .324 | 1.111 | .000 | .000 | .000 | .000 | .000 | .000 | 1.111 |
| ESE\_3 | .035 | .026 | -.013 | .363 | .303 | 1.046 | .000 | .000 | .000 | .000 | .000 | .000 | 1.046 |
| ESE\_2 | .037 | .027 | -.014 | .379 | .318 | 1.091 | .000 | .000 | .000 | .000 | .000 | .000 | 1.091 |
| ESE\_1 | .036 | .026 | -.013 | .369 | .309 | 1.000 | .000 | .000 | .000 | .000 | .000 | .000 | 1.000 |
| EOE\_9 | .039 | .042 | .004 | .398 | 1.061 | .000 | .000 | .000 | .000 | .000 | .000 | .000 | .000 |
| EOE\_8 | .040 | .044 | .004 | .407 | 1.085 | .000 | .000 | .000 | .000 | .000 | .000 | .000 | .000 |
| EOE\_7 | .040 | .044 | .004 | .409 | 1.092 | .000 | .000 | .000 | .000 | .000 | .000 | .000 | .000 |
| EOE\_6 | .038 | .042 | .004 | .393 | 1.051 | .000 | .000 | .000 | .000 | .000 | .000 | .000 | .000 |
| EOE\_5 | .040 | .044 | .004 | .409 | 1.093 | .000 | .000 | .000 | .000 | .000 | .000 | .000 | .000 |
| EOE\_4 | .038 | .042 | .004 | .395 | 1.051 | .000 | .000 | .000 | .000 | .000 | .000 | .000 | .000 |
| EOE\_3 | .039 | .043 | .004 | .398 | 1.059 | .000 | .000 | .000 | .000 | .000 | .000 | .000 | .000 |
| EOE\_2 | .039 | .043 | .004 | .403 | 1.070 | .000 | .000 | .000 | .000 | .000 | .000 | .000 | .000 |
| EOE\_1 | .039 | .042 | .004 | .396 | 1.000 | .000 | .000 | .000 | .000 | .000 | .000 | .000 | .000 |
| AISE\_6 | .000 | .000 | .000 | 1.052 | .000 | .000 | .000 | .000 | .000 | .000 | .000 | .000 | .000 |
| AISE\_5 | .000 | .000 | .000 | 1.009 | .000 | .000 | .000 | .000 | .000 | .000 | .000 | .000 | .000 |
| AISE\_4 | .000 | .000 | .000 | 1.046 | .000 | .000 | .000 | .000 | .000 | .000 | .000 | .000 | .000 |
| AISE\_3 | .000 | .000 | .000 | 1.012 | .000 | .000 | .000 | .000 | .000 | .000 | .000 | .000 | .000 |
| AISE\_2 | .000 | .000 | .000 | 1.036 | .000 | .000 | .000 | .000 | .000 | .000 | .000 | .000 | .000 |
| AISE\_1 | .000 | .000 | .000 | 1.000 | .000 | .000 | .000 | .000 | .000 | .000 | .000 | .000 | .000 |

## Total Effects - Two Tailed Significance (PC) (Group number 1 - Default model)

|  | Grade | Age | Gender | AISE | EOE | ESE | EE | Pa | Vc | Fs | Vp | Ss | Sm |
| --- | --- | --- | --- | --- | --- | --- | --- | --- | --- | --- | --- | --- | --- |
| EOE | .148 | .084 | .075 | .000 | ... | ... | ... | ... | ... | ... | ... | ... | ... |
| ESE | .243 | .675 | .010 | .000 | .000 | ... | ... | ... | ... | ... | ... | ... | ... |
| EE | .062 | .041 | .001 | .000 | .000 | .000 | ... | ... | ... | ... | ... | ... | ... |
| Pa | .062 | .041 | .001 | .000 | .000 | .000 | .000 | ... | ... | ... | ... | ... | ... |
| Vc | .062 | .041 | .001 | .000 | .000 | .000 | .000 | ... | ... | ... | ... | ... | ... |
| Fs | .062 | .041 | .001 | .000 | .000 | .000 | .000 | ... | ... | ... | ... | ... | ... |
| Vp | .062 | .041 | .001 | .000 | .000 | .000 | ... | ... | ... | ... | ... | ... | ... |
| Ss | .243 | .675 | .010 | .000 | .000 | .000 | ... | ... | ... | ... | ... | ... | ... |
| Sm | .243 | .675 | .010 | .000 | .000 | ... | ... | ... | ... | ... | ... | ... | ... |
| EE\_20 | .062 | .041 | .001 | .000 | .000 | .000 | .000 | .000 | ... | ... | ... | ... | ... |
| EE\_19 | .062 | .041 | .001 | .000 | .000 | .000 | .000 | .000 | ... | ... | ... | ... | ... |
| EE\_18 | .062 | .041 | .001 | .000 | .000 | .000 | .000 | ... | ... | ... | ... | ... | ... |
| EE\_17 | .062 | .041 | .001 | .000 | .000 | .000 | .000 | ... | .000 | ... | ... | ... | ... |
| EE\_16 | .062 | .041 | .001 | .000 | .000 | .000 | .000 | ... | .000 | ... | ... | ... | ... |
| EE\_15 | .062 | .041 | .001 | .000 | .000 | .000 | .000 | ... | .000 | ... | ... | ... | ... |
| EE\_14 | .062 | .041 | .001 | .000 | .000 | .000 | .000 | ... | .000 | ... | ... | ... | ... |
| EE\_13 | .062 | .041 | .001 | .000 | .000 | .000 | .000 | ... | ... | ... | ... | ... | ... |
| EE\_12 | .062 | .041 | .001 | .000 | .000 | .000 | .000 | ... | ... | .000 | ... | ... | ... |
| EE\_11 | .062 | .041 | .001 | .000 | .000 | .000 | .000 | ... | ... | .000 | ... | ... | ... |
| EE\_10 | .062 | .041 | .001 | .000 | .000 | .000 | .000 | ... | ... | .000 | ... | ... | ... |
| EE\_9 | .062 | .041 | .001 | .000 | .000 | .000 | .000 | ... | ... | .000 | ... | ... | ... |
| EE\_8 | .062 | .041 | .001 | .000 | .000 | .000 | .000 | ... | ... | .000 | ... | ... | ... |
| EE\_7 | .062 | .041 | .001 | .000 | .000 | .000 | .000 | ... | ... | ... | ... | ... | ... |
| EE\_6 | .062 | .041 | .001 | .000 | .000 | .000 | .000 | ... | ... | ... | .000 | ... | ... |
| EE\_5 | .062 | .041 | .001 | .000 | .000 | .000 | .000 | ... | ... | ... | .000 | ... | ... |
| EE\_4 | .062 | .041 | .001 | .000 | .000 | .000 | .000 | ... | ... | ... | .000 | ... | ... |
| EE\_3 | .062 | .041 | .001 | .000 | .000 | .000 | .000 | ... | ... | ... | .000 | ... | ... |
| EE\_2 | .062 | .041 | .001 | .000 | .000 | .000 | .000 | ... | ... | ... | .000 | ... | ... |
| EE\_1 | .062 | .041 | .001 | .000 | .000 | .000 | ... | ... | ... | ... | ... | ... | ... |
| ESE\_10 | .243 | .675 | .010 | .000 | .000 | .000 | ... | ... | ... | ... | ... | .000 | ... |
| ESE\_9 | .243 | .675 | .010 | .000 | .000 | .000 | ... | ... | ... | ... | ... | .000 | ... |
| ESE\_8 | .243 | .675 | .010 | .000 | .000 | .000 | ... | ... | ... | ... | ... | .000 | ... |
| ESE\_7 | .243 | .675 | .010 | .000 | .000 | .000 | ... | ... | ... | ... | ... | ... | ... |
| ESE\_6 | .243 | .675 | .010 | .000 | .000 | .000 | ... | ... | ... | ... | ... | ... | .000 |
| ESE\_5 | .243 | .675 | .010 | .000 | .000 | .000 | ... | ... | ... | ... | ... | ... | .000 |
| ESE\_4 | .243 | .675 | .010 | .000 | .000 | .000 | ... | ... | ... | ... | ... | ... | .000 |
| ESE\_3 | .243 | .675 | .010 | .000 | .000 | .000 | ... | ... | ... | ... | ... | ... | .000 |
| ESE\_2 | .243 | .675 | .010 | .000 | .000 | .000 | ... | ... | ... | ... | ... | ... | .000 |
| ESE\_1 | .243 | .675 | .010 | .000 | .000 | ... | ... | ... | ... | ... | ... | ... | ... |
| EOE\_9 | .148 | .084 | .075 | .000 | .000 | ... | ... | ... | ... | ... | ... | ... | ... |
| EOE\_8 | .148 | .084 | .075 | .000 | .000 | ... | ... | ... | ... | ... | ... | ... | ... |
| EOE\_7 | .148 | .084 | .075 | .000 | .000 | ... | ... | ... | ... | ... | ... | ... | ... |
| EOE\_6 | .148 | .084 | .075 | .000 | .000 | ... | ... | ... | ... | ... | ... | ... | ... |
| EOE\_5 | .148 | .084 | .075 | .000 | .000 | ... | ... | ... | ... | ... | ... | ... | ... |
| EOE\_4 | .148 | .084 | .075 | .000 | .000 | ... | ... | ... | ... | ... | ... | ... | ... |
| EOE\_3 | .148 | .084 | .075 | .000 | .000 | ... | ... | ... | ... | ... | ... | ... | ... |
| EOE\_2 | .148 | .084 | .075 | .000 | .000 | ... | ... | ... | ... | ... | ... | ... | ... |
| EOE\_1 | .148 | .084 | .075 | .000 | ... | ... | ... | ... | ... | ... | ... | ... | ... |
| AISE\_6 | ... | ... | ... | .000 | ... | ... | ... | ... | ... | ... | ... | ... | ... |
| AISE\_5 | ... | ... | ... | .000 | ... | ... | ... | ... | ... | ... | ... | ... | ... |
| AISE\_4 | ... | ... | ... | .000 | ... | ... | ... | ... | ... | ... | ... | ... | ... |
| AISE\_3 | ... | ... | ... | .000 | ... | ... | ... | ... | ... | ... | ... | ... | ... |
| AISE\_2 | ... | ... | ... | .000 | ... | ... | ... | ... | ... | ... | ... | ... | ... |
| AISE\_1 | ... | ... | ... | ... | ... | ... | ... | ... | ... | ... | ... | ... | ... |

## Standardized Total Effects (Group number 1 - Default model)

## Standardized Total Effects - Lower Bounds (PC) (Group number 1 - Default model)

|  | Grade | Age | Gender | AISE | EOE | ESE | EE | Pa | Vc | Fs | Vp | Ss | Sm |
| --- | --- | --- | --- | --- | --- | --- | --- | --- | --- | --- | --- | --- | --- |
| EOE | -.011 | -.003 | -.073 | .513 | .000 | .000 | .000 | .000 | .000 | .000 | .000 | .000 | .000 |
| ESE | -.017 | -.034 | -.104 | .554 | .248 | .000 | .000 | .000 | .000 | .000 | .000 | .000 | .000 |
| EE | -.002 | .002 | -.114 | .462 | .225 | .163 | .000 | .000 | .000 | .000 | .000 | .000 | .000 |
| Pa | -.001 | .001 | -.092 | .366 | .180 | .131 | .775 | .000 | .000 | .000 | .000 | .000 | .000 |
| Vc | -.001 | .001 | -.094 | .378 | .185 | .133 | .801 | .000 | .000 | .000 | .000 | .000 | .000 |
| Fs | -.001 | .001 | -.093 | .372 | .183 | .132 | .789 | .000 | .000 | .000 | .000 | .000 | .000 |
| Vp | -.001 | .001 | -.092 | .369 | .181 | .131 | .783 | .000 | .000 | .000 | .000 | .000 | .000 |
| Ss | -.013 | -.027 | -.081 | .428 | .193 | .748 | .000 | .000 | .000 | .000 | .000 | .000 | .000 |
| Sm | -.013 | -.028 | -.085 | .445 | .199 | .777 | .000 | .000 | .000 | .000 | .000 | .000 | .000 |
| EE\_20 | -.001 | .001 | -.067 | .263 | .130 | .094 | .552 | .696 | .000 | .000 | .000 | .000 | .000 |
| EE\_19 | -.001 | .001 | -.066 | .259 | .128 | .093 | .545 | .688 | .000 | .000 | .000 | .000 | .000 |
| EE\_18 | -.001 | .001 | -.068 | .268 | .132 | .096 | .564 | .711 | .000 | .000 | .000 | .000 | .000 |
| EE\_17 | -.001 | .001 | -.070 | .276 | .136 | .099 | .580 | .000 | .714 | .000 | .000 | .000 | .000 |
| EE\_16 | -.001 | .001 | -.069 | .276 | .135 | .098 | .578 | .000 | .713 | .000 | .000 | .000 | .000 |
| EE\_15 | -.001 | .001 | -.071 | .280 | .138 | .100 | .590 | .000 | .726 | .000 | .000 | .000 | .000 |
| EE\_14 | -.001 | .001 | -.069 | .275 | .135 | .098 | .578 | .000 | .711 | .000 | .000 | .000 | .000 |
| EE\_13 | -.001 | .001 | -.071 | .281 | .139 | .100 | .591 | .000 | .728 | .000 | .000 | .000 | .000 |
| EE\_12 | -.001 | .001 | -.068 | .271 | .134 | .097 | .570 | .000 | .000 | .712 | .000 | .000 | .000 |
| EE\_11 | -.001 | .001 | -.068 | .271 | .134 | .097 | .571 | .000 | .000 | .713 | .000 | .000 | .000 |
| EE\_10 | -.001 | .001 | -.069 | .272 | .134 | .097 | .573 | .000 | .000 | .715 | .000 | .000 | .000 |
| EE\_9 | -.001 | .001 | -.069 | .273 | .134 | .097 | .574 | .000 | .000 | .717 | .000 | .000 | .000 |
| EE\_8 | -.001 | .001 | -.067 | .263 | .130 | .095 | .554 | .000 | .000 | .691 | .000 | .000 | .000 |
| EE\_7 | -.001 | .001 | -.067 | .269 | .133 | .096 | .567 | .000 | .000 | .707 | .000 | .000 | .000 |
| EE\_6 | -.001 | .001 | -.068 | .271 | .133 | .097 | .570 | .000 | .000 | .000 | .718 | .000 | .000 |
| EE\_5 | -.001 | .001 | -.067 | .266 | .131 | .095 | .558 | .000 | .000 | .000 | .703 | .000 | .000 |
| EE\_4 | -.001 | .001 | -.068 | .268 | .133 | .096 | .565 | .000 | .000 | .000 | .710 | .000 | .000 |
| EE\_3 | -.001 | .001 | -.069 | .273 | .134 | .098 | .572 | .000 | .000 | .000 | .722 | .000 | .000 |
| EE\_2 | -.001 | .001 | -.067 | .264 | .130 | .095 | .555 | .000 | .000 | .000 | .698 | .000 | .000 |
| EE\_1 | -.001 | .001 | -.066 | .263 | .130 | .094 | .551 | .000 | .000 | .000 | .694 | .000 | .000 |
| ESE\_10 | -.010 | -.020 | -.060 | .310 | .142 | .542 | .000 | .000 | .000 | .000 | .000 | .708 | .000 |
| ESE\_9 | -.009 | -.019 | -.058 | .305 | .139 | .530 | .000 | .000 | .000 | .000 | .000 | .692 | .000 |
| ESE\_8 | -.010 | -.020 | -.061 | .318 | .144 | .553 | .000 | .000 | .000 | .000 | .000 | .724 | .000 |
| ESE\_7 | -.010 | -.020 | -.060 | .317 | .144 | .552 | .000 | .000 | .000 | .000 | .000 | .722 | .000 |
| ESE\_6 | -.010 | -.020 | -.062 | .318 | .143 | .552 | .000 | .000 | .000 | .000 | .000 | .000 | .697 |
| ESE\_5 | -.010 | -.020 | -.063 | .326 | .147 | .567 | .000 | .000 | .000 | .000 | .000 | .000 | .717 |
| ESE\_4 | -.010 | -.020 | -.063 | .327 | .147 | .568 | .000 | .000 | .000 | .000 | .000 | .000 | .716 |
| ESE\_3 | -.010 | -.020 | -.061 | .314 | .142 | .545 | .000 | .000 | .000 | .000 | .000 | .000 | .688 |
| ESE\_2 | -.010 | -.020 | -.063 | .323 | .146 | .562 | .000 | .000 | .000 | .000 | .000 | .000 | .710 |
| ESE\_1 | -.009 | -.020 | -.061 | .313 | .142 | .545 | .000 | .000 | .000 | .000 | .000 | .000 | .688 |
| EOE\_9 | -.008 | -.002 | -.053 | .364 | .697 | .000 | .000 | .000 | .000 | .000 | .000 | .000 | .000 |
| EOE\_8 | -.008 | -.003 | -.053 | .369 | .707 | .000 | .000 | .000 | .000 | .000 | .000 | .000 | .000 |
| EOE\_7 | -.008 | -.003 | -.055 | .379 | .726 | .000 | .000 | .000 | .000 | .000 | .000 | .000 | .000 |
| EOE\_6 | -.008 | -.002 | -.052 | .364 | .694 | .000 | .000 | .000 | .000 | .000 | .000 | .000 | .000 |
| EOE\_5 | -.008 | -.003 | -.054 | .375 | .717 | .000 | .000 | .000 | .000 | .000 | .000 | .000 | .000 |
| EOE\_4 | -.008 | -.003 | -.053 | .367 | .703 | .000 | .000 | .000 | .000 | .000 | .000 | .000 | .000 |
| EOE\_3 | -.008 | -.003 | -.053 | .366 | .699 | .000 | .000 | .000 | .000 | .000 | .000 | .000 | .000 |
| EOE\_2 | -.008 | -.003 | -.053 | .370 | .705 | .000 | .000 | .000 | .000 | .000 | .000 | .000 | .000 |
| EOE\_1 | -.008 | -.003 | -.053 | .365 | .698 | .000 | .000 | .000 | .000 | .000 | .000 | .000 | .000 |
| AISE\_6 | .000 | .000 | .000 | .731 | .000 | .000 | .000 | .000 | .000 | .000 | .000 | .000 | .000 |
| AISE\_5 | .000 | .000 | .000 | .739 | .000 | .000 | .000 | .000 | .000 | .000 | .000 | .000 | .000 |
| AISE\_4 | .000 | .000 | .000 | .726 | .000 | .000 | .000 | .000 | .000 | .000 | .000 | .000 | .000 |
| AISE\_3 | .000 | .000 | .000 | .714 | .000 | .000 | .000 | .000 | .000 | .000 | .000 | .000 | .000 |
| AISE\_2 | .000 | .000 | .000 | .736 | .000 | .000 | .000 | .000 | .000 | .000 | .000 | .000 | .000 |
| AISE\_1 | .000 | .000 | .000 | .698 | .000 | .000 | .000 | .000 | .000 | .000 | .000 | .000 | .000 |

## Standardized Total Effects - Upper Bounds (PC) (Group number 1 - Default model)

|  | Grade | Age | Gender | AISE | EOE | ESE | EE | Pa | Vc | Fs | Vp | Ss | Sm |
| --- | --- | --- | --- | --- | --- | --- | --- | --- | --- | --- | --- | --- | --- |
| EOE | .064 | .073 | .003 | .581 | .000 | .000 | .000 | .000 | .000 | .000 | .000 | .000 | .000 |
| ESE | .069 | .053 | -.015 | .631 | .355 | .000 | .000 | .000 | .000 | .000 | .000 | .000 | .000 |
| EE | .080 | .082 | -.034 | .537 | .325 | .300 | .000 | .000 | .000 | .000 | .000 | .000 | .000 |
| Pa | .064 | .066 | -.027 | .435 | .264 | .242 | .831 | .000 | .000 | .000 | .000 | .000 | .000 |
| Vc | .065 | .067 | -.028 | .444 | .269 | .247 | .846 | .000 | .000 | .000 | .000 | .000 | .000 |
| Fs | .064 | .067 | -.027 | .441 | .266 | .244 | .836 | .000 | .000 | .000 | .000 | .000 | .000 |
| Vp | .064 | .066 | -.027 | .436 | .263 | .243 | .830 | .000 | .000 | .000 | .000 | .000 | .000 |
| Ss | .054 | .042 | -.011 | .502 | .280 | .819 | .000 | .000 | .000 | .000 | .000 | .000 | .000 |
| Sm | .056 | .043 | -.012 | .516 | .291 | .845 | .000 | .000 | .000 | .000 | .000 | .000 | .000 |
| EE\_20 | .046 | .048 | -.019 | .317 | .192 | .175 | .610 | .748 | .000 | .000 | .000 | .000 | .000 |
| EE\_19 | .045 | .047 | -.019 | .314 | .190 | .174 | .604 | .740 | .000 | .000 | .000 | .000 | .000 |
| EE\_18 | .047 | .049 | -.020 | .324 | .195 | .179 | .621 | .762 | .000 | .000 | .000 | .000 | .000 |
| EE\_17 | .048 | .050 | -.020 | .331 | .199 | .183 | .634 | .000 | .760 | .000 | .000 | .000 | .000 |
| EE\_16 | .048 | .050 | -.020 | .329 | .199 | .183 | .631 | .000 | .757 | .000 | .000 | .000 | .000 |
| EE\_15 | .049 | .051 | -.021 | .335 | .202 | .185 | .643 | .000 | .769 | .000 | .000 | .000 | .000 |
| EE\_14 | .048 | .050 | -.020 | .330 | .198 | .181 | .630 | .000 | .755 | .000 | .000 | .000 | .000 |
| EE\_13 | .049 | .051 | -.021 | .336 | .202 | .186 | .644 | .000 | .771 | .000 | .000 | .000 | .000 |
| EE\_12 | .047 | .049 | -.020 | .327 | .196 | .180 | .624 | .000 | .000 | .756 | .000 | .000 | .000 |
| EE\_11 | .047 | .049 | -.020 | .326 | .196 | .181 | .625 | .000 | .000 | .758 | .000 | .000 | .000 |
| EE\_10 | .047 | .049 | -.020 | .328 | .196 | .181 | .625 | .000 | .000 | .757 | .000 | .000 | .000 |
| EE\_9 | .048 | .049 | -.020 | .329 | .197 | .182 | .628 | .000 | .000 | .761 | .000 | .000 | .000 |
| EE\_8 | .046 | .048 | -.019 | .318 | .191 | .175 | .608 | .000 | .000 | .737 | .000 | .000 | .000 |
| EE\_7 | .047 | .049 | -.020 | .323 | .194 | .179 | .618 | .000 | .000 | .750 | .000 | .000 | .000 |
| EE\_6 | .048 | .049 | -.020 | .325 | .196 | .181 | .625 | .000 | .000 | .000 | .761 | .000 | .000 |
| EE\_5 | .047 | .048 | -.020 | .319 | .192 | .176 | .612 | .000 | .000 | .000 | .747 | .000 | .000 |
| EE\_4 | .047 | .048 | -.020 | .323 | .193 | .178 | .616 | .000 | .000 | .000 | .753 | .000 | .000 |
| EE\_3 | .048 | .050 | -.020 | .327 | .196 | .181 | .627 | .000 | .000 | .000 | .764 | .000 | .000 |
| EE\_2 | .046 | .048 | -.020 | .318 | .191 | .175 | .609 | .000 | .000 | .000 | .744 | .000 | .000 |
| EE\_1 | .046 | .048 | -.020 | .317 | .191 | .175 | .608 | .000 | .000 | .000 | .742 | .000 | .000 |
| ESE\_10 | .040 | .031 | -.008 | .373 | .206 | .609 | .000 | .000 | .000 | .000 | .000 | .758 | .000 |
| ESE\_9 | .039 | .030 | -.008 | .362 | .201 | .593 | .000 | .000 | .000 | .000 | .000 | .740 | .000 |
| ESE\_8 | .041 | .031 | -.009 | .379 | .210 | .618 | .000 | .000 | .000 | .000 | .000 | .772 | .000 |
| ESE\_7 | .041 | .031 | -.009 | .376 | .209 | .615 | .000 | .000 | .000 | .000 | .000 | .766 | .000 |
| ESE\_6 | .041 | .031 | -.008 | .376 | .210 | .617 | .000 | .000 | .000 | .000 | .000 | .000 | .743 |
| ESE\_5 | .042 | .032 | -.009 | .385 | .216 | .632 | .000 | .000 | .000 | .000 | .000 | .000 | .760 |
| ESE\_4 | .042 | .032 | -.009 | .385 | .216 | .631 | .000 | .000 | .000 | .000 | .000 | .000 | .760 |
| ESE\_3 | .040 | .031 | -.008 | .372 | .208 | .609 | .000 | .000 | .000 | .000 | .000 | .000 | .736 |
| ESE\_2 | .041 | .032 | -.009 | .383 | .214 | .627 | .000 | .000 | .000 | .000 | .000 | .000 | .757 |
| ESE\_1 | .040 | .031 | -.008 | .371 | .208 | .609 | .000 | .000 | .000 | .000 | .000 | .000 | .735 |
| EOE\_9 | .046 | .052 | .002 | .422 | .740 | .000 | .000 | .000 | .000 | .000 | .000 | .000 | .000 |
| EOE\_8 | .046 | .053 | .002 | .427 | .749 | .000 | .000 | .000 | .000 | .000 | .000 | .000 | .000 |
| EOE\_7 | .047 | .054 | .003 | .437 | .765 | .000 | .000 | .000 | .000 | .000 | .000 | .000 | .000 |
| EOE\_6 | .045 | .052 | .002 | .420 | .738 | .000 | .000 | .000 | .000 | .000 | .000 | .000 | .000 |
| EOE\_5 | .047 | .054 | .002 | .433 | .758 | .000 | .000 | .000 | .000 | .000 | .000 | .000 | .000 |
| EOE\_4 | .046 | .053 | .002 | .425 | .744 | .000 | .000 | .000 | .000 | .000 | .000 | .000 | .000 |
| EOE\_3 | .046 | .053 | .002 | .423 | .742 | .000 | .000 | .000 | .000 | .000 | .000 | .000 | .000 |
| EOE\_2 | .046 | .053 | .002 | .427 | .749 | .000 | .000 | .000 | .000 | .000 | .000 | .000 | .000 |
| EOE\_1 | .046 | .052 | .002 | .422 | .741 | .000 | .000 | .000 | .000 | .000 | .000 | .000 | .000 |
| AISE\_6 | .000 | .000 | .000 | .773 | .000 | .000 | .000 | .000 | .000 | .000 | .000 | .000 | .000 |
| AISE\_5 | .000 | .000 | .000 | .780 | .000 | .000 | .000 | .000 | .000 | .000 | .000 | .000 | .000 |
| AISE\_4 | .000 | .000 | .000 | .768 | .000 | .000 | .000 | .000 | .000 | .000 | .000 | .000 | .000 |
| AISE\_3 | .000 | .000 | .000 | .757 | .000 | .000 | .000 | .000 | .000 | .000 | .000 | .000 | .000 |
| AISE\_2 | .000 | .000 | .000 | .776 | .000 | .000 | .000 | .000 | .000 | .000 | .000 | .000 | .000 |
| AISE\_1 | .000 | .000 | .000 | .743 | .000 | .000 | .000 | .000 | .000 | .000 | .000 | .000 | .000 |

## Standardized Total Effects - Two Tailed Significance (PC) (Group number 1 - Default model)

|  | Grade | Age | Gender | AISE | EOE | ESE | EE | Pa | Vc | Fs | Vp | Ss | Sm |
| --- | --- | --- | --- | --- | --- | --- | --- | --- | --- | --- | --- | --- | --- |
| EOE | .148 | .084 | .075 | .000 | ... | ... | ... | ... | ... | ... | ... | ... | ... |
| ESE | .243 | .675 | .010 | .000 | .000 | ... | ... | ... | ... | ... | ... | ... | ... |
| EE | .062 | .041 | .001 | .000 | .000 | .000 | ... | ... | ... | ... | ... | ... | ... |
| Pa | .062 | .041 | .001 | .000 | .000 | .000 | .000 | ... | ... | ... | ... | ... | ... |
| Vc | .062 | .041 | .001 | .000 | .000 | .000 | .000 | ... | ... | ... | ... | ... | ... |
| Fs | .062 | .041 | .001 | .000 | .000 | .000 | .000 | ... | ... | ... | ... | ... | ... |
| Vp | .062 | .041 | .001 | .000 | .000 | .000 | .000 | ... | ... | ... | ... | ... | ... |
| Ss | .243 | .675 | .010 | .000 | .000 | .000 | ... | ... | ... | ... | ... | ... | ... |
| Sm | .243 | .675 | .010 | .000 | .000 | .000 | ... | ... | ... | ... | ... | ... | ... |
| EE\_20 | .062 | .041 | .001 | .000 | .000 | .000 | .000 | .000 | ... | ... | ... | ... | ... |
| EE\_19 | .062 | .041 | .001 | .000 | .000 | .000 | .000 | .000 | ... | ... | ... | ... | ... |
| EE\_18 | .062 | .041 | .001 | .000 | .000 | .000 | .000 | .000 | ... | ... | ... | ... | ... |
| EE\_17 | .062 | .041 | .001 | .000 | .000 | .000 | .000 | ... | .000 | ... | ... | ... | ... |
| EE\_16 | .062 | .041 | .001 | .000 | .000 | .000 | .000 | ... | .000 | ... | ... | ... | ... |
| EE\_15 | .062 | .041 | .001 | .000 | .000 | .000 | .000 | ... | .000 | ... | ... | ... | ... |
| EE\_14 | .062 | .041 | .001 | .000 | .000 | .000 | .000 | ... | .000 | ... | ... | ... | ... |
| EE\_13 | .062 | .041 | .001 | .000 | .000 | .000 | .000 | ... | .000 | ... | ... | ... | ... |
| EE\_12 | .062 | .041 | .001 | .000 | .000 | .000 | .000 | ... | ... | .000 | ... | ... | ... |
| EE\_11 | .062 | .041 | .001 | .000 | .000 | .000 | .000 | ... | ... | .000 | ... | ... | ... |
| EE\_10 | .062 | .041 | .001 | .000 | .000 | .000 | .000 | ... | ... | .000 | ... | ... | ... |
| EE\_9 | .062 | .041 | .001 | .000 | .000 | .000 | .000 | ... | ... | .000 | ... | ... | ... |
| EE\_8 | .062 | .041 | .001 | .000 | .000 | .000 | .000 | ... | ... | .000 | ... | ... | ... |
| EE\_7 | .062 | .041 | .001 | .000 | .000 | .000 | .000 | ... | ... | .000 | ... | ... | ... |
| EE\_6 | .062 | .041 | .001 | .000 | .000 | .000 | .000 | ... | ... | ... | .000 | ... | ... |
| EE\_5 | .062 | .041 | .001 | .000 | .000 | .000 | .000 | ... | ... | ... | .000 | ... | ... |
| EE\_4 | .062 | .041 | .001 | .000 | .000 | .000 | .000 | ... | ... | ... | .000 | ... | ... |
| EE\_3 | .062 | .041 | .001 | .000 | .000 | .000 | .000 | ... | ... | ... | .000 | ... | ... |
| EE\_2 | .062 | .041 | .001 | .000 | .000 | .000 | .000 | ... | ... | ... | .000 | ... | ... |
| EE\_1 | .062 | .041 | .001 | .000 | .000 | .000 | .000 | ... | ... | ... | .000 | ... | ... |
| ESE\_10 | .243 | .675 | .010 | .000 | .000 | .000 | ... | ... | ... | ... | ... | .000 | ... |
| ESE\_9 | .243 | .675 | .010 | .000 | .000 | .000 | ... | ... | ... | ... | ... | .000 | ... |
| ESE\_8 | .243 | .675 | .010 | .000 | .000 | .000 | ... | ... | ... | ... | ... | .000 | ... |
| ESE\_7 | .243 | .675 | .010 | .000 | .000 | .000 | ... | ... | ... | ... | ... | .000 | ... |
| ESE\_6 | .243 | .675 | .010 | .000 | .000 | .000 | ... | ... | ... | ... | ... | ... | .000 |
| ESE\_5 | .243 | .675 | .010 | .000 | .000 | .000 | ... | ... | ... | ... | ... | ... | .000 |
| ESE\_4 | .243 | .675 | .010 | .000 | .000 | .000 | ... | ... | ... | ... | ... | ... | .000 |
| ESE\_3 | .243 | .675 | .010 | .000 | .000 | .000 | ... | ... | ... | ... | ... | ... | .000 |
| ESE\_2 | .243 | .675 | .010 | .000 | .000 | .000 | ... | ... | ... | ... | ... | ... | .000 |
| ESE\_1 | .243 | .675 | .010 | .000 | .000 | .000 | ... | ... | ... | ... | ... | ... | .000 |
| EOE\_9 | .148 | .084 | .075 | .000 | .000 | ... | ... | ... | ... | ... | ... | ... | ... |
| EOE\_8 | .148 | .084 | .075 | .000 | .000 | ... | ... | ... | ... | ... | ... | ... | ... |
| EOE\_7 | .148 | .084 | .075 | .000 | .000 | ... | ... | ... | ... | ... | ... | ... | ... |
| EOE\_6 | .148 | .084 | .075 | .000 | .000 | ... | ... | ... | ... | ... | ... | ... | ... |
| EOE\_5 | .148 | .084 | .075 | .000 | .000 | ... | ... | ... | ... | ... | ... | ... | ... |
| EOE\_4 | .148 | .084 | .075 | .000 | .000 | ... | ... | ... | ... | ... | ... | ... | ... |
| EOE\_3 | .148 | .084 | .075 | .000 | .000 | ... | ... | ... | ... | ... | ... | ... | ... |
| EOE\_2 | .148 | .084 | .075 | .000 | .000 | ... | ... | ... | ... | ... | ... | ... | ... |
| EOE\_1 | .148 | .084 | .075 | .000 | .000 | ... | ... | ... | ... | ... | ... | ... | ... |
| AISE\_6 | ... | ... | ... | .000 | ... | ... | ... | ... | ... | ... | ... | ... | ... |
| AISE\_5 | ... | ... | ... | .000 | ... | ... | ... | ... | ... | ... | ... | ... | ... |
| AISE\_4 | ... | ... | ... | .000 | ... | ... | ... | ... | ... | ... | ... | ... | ... |
| AISE\_3 | ... | ... | ... | .000 | ... | ... | ... | ... | ... | ... | ... | ... | ... |
| AISE\_2 | ... | ... | ... | .000 | ... | ... | ... | ... | ... | ... | ... | ... | ... |
| AISE\_1 | ... | ... | ... | .000 | ... | ... | ... | ... | ... | ... | ... | ... | ... |

## Direct Effects (Group number 1 - Default model)

## Direct Effects - Lower Bounds (PC) (Group number 1 - Default model)

|  | Grade | Age | Gender | AISE | EOE | ESE | EE | Pa | Vc | Fs | Vp | Ss | Sm |
| --- | --- | --- | --- | --- | --- | --- | --- | --- | --- | --- | --- | --- | --- |
| EOE | -.006 | -.002 | -.078 | .332 | .000 | .000 | .000 | .000 | .000 | .000 | .000 | .000 | .000 |
| ESE | -.013 | -.021 | -.085 | .208 | .208 | .000 | .000 | .000 | .000 | .000 | .000 | .000 | .000 |
| EE | -.006 | -.003 | -.084 | .106 | .129 | .160 | .000 | .000 | .000 | .000 | .000 | .000 | .000 |
| Pa | .000 | .000 | .000 | .000 | .000 | .000 | .896 | .000 | .000 | .000 | .000 | .000 | .000 |
| Vc | .000 | .000 | .000 | .000 | .000 | .000 | .980 | .000 | .000 | .000 | .000 | .000 | .000 |
| Fs | .000 | .000 | .000 | .000 | .000 | .000 | .954 | .000 | .000 | .000 | .000 | .000 | .000 |
| Vp | .000 | .000 | .000 | .000 | .000 | .000 | 1.000 | .000 | .000 | .000 | .000 | .000 | .000 |
| Ss | .000 | .000 | .000 | .000 | .000 | .901 | .000 | .000 | .000 | .000 | .000 | .000 | .000 |
| Sm | .000 | .000 | .000 | .000 | .000 | 1.000 | .000 | .000 | .000 | .000 | .000 | .000 | .000 |
| EE\_20 | .000 | .000 | .000 | .000 | .000 | .000 | .000 | .930 | .000 | .000 | .000 | .000 | .000 |
| EE\_19 | .000 | .000 | .000 | .000 | .000 | .000 | .000 | .916 | .000 | .000 | .000 | .000 | .000 |
| EE\_18 | .000 | .000 | .000 | .000 | .000 | .000 | .000 | 1.000 | .000 | .000 | .000 | .000 | .000 |
| EE\_17 | .000 | .000 | .000 | .000 | .000 | .000 | .000 | .000 | .913 | .000 | .000 | .000 | .000 |
| EE\_16 | .000 | .000 | .000 | .000 | .000 | .000 | .000 | .000 | .925 | .000 | .000 | .000 | .000 |
| EE\_15 | .000 | .000 | .000 | .000 | .000 | .000 | .000 | .000 | .936 | .000 | .000 | .000 | .000 |
| EE\_14 | .000 | .000 | .000 | .000 | .000 | .000 | .000 | .000 | .921 | .000 | .000 | .000 | .000 |
| EE\_13 | .000 | .000 | .000 | .000 | .000 | .000 | .000 | .000 | 1.000 | .000 | .000 | .000 | .000 |
| EE\_12 | .000 | .000 | .000 | .000 | .000 | .000 | .000 | .000 | .000 | .936 | .000 | .000 | .000 |
| EE\_11 | .000 | .000 | .000 | .000 | .000 | .000 | .000 | .000 | .000 | .945 | .000 | .000 | .000 |
| EE\_10 | .000 | .000 | .000 | .000 | .000 | .000 | .000 | .000 | .000 | .949 | .000 | .000 | .000 |
| EE\_9 | .000 | .000 | .000 | .000 | .000 | .000 | .000 | .000 | .000 | .952 | .000 | .000 | .000 |
| EE\_8 | .000 | .000 | .000 | .000 | .000 | .000 | .000 | .000 | .000 | .916 | .000 | .000 | .000 |
| EE\_7 | .000 | .000 | .000 | .000 | .000 | .000 | .000 | .000 | .000 | 1.000 | .000 | .000 | .000 |
| EE\_6 | .000 | .000 | .000 | .000 | .000 | .000 | .000 | .000 | .000 | .000 | .979 | .000 | .000 |
| EE\_5 | .000 | .000 | .000 | .000 | .000 | .000 | .000 | .000 | .000 | .000 | .967 | .000 | .000 |
| EE\_4 | .000 | .000 | .000 | .000 | .000 | .000 | .000 | .000 | .000 | .000 | .963 | .000 | .000 |
| EE\_3 | .000 | .000 | .000 | .000 | .000 | .000 | .000 | .000 | .000 | .000 | .995 | .000 | .000 |
| EE\_2 | .000 | .000 | .000 | .000 | .000 | .000 | .000 | .000 | .000 | .000 | .962 | .000 | .000 |
| EE\_1 | .000 | .000 | .000 | .000 | .000 | .000 | .000 | .000 | .000 | .000 | 1.000 | .000 | .000 |
| ESE\_10 | .000 | .000 | .000 | .000 | .000 | .000 | .000 | .000 | .000 | .000 | .000 | .925 | .000 |
| ESE\_9 | .000 | .000 | .000 | .000 | .000 | .000 | .000 | .000 | .000 | .000 | .000 | .891 | .000 |
| ESE\_8 | .000 | .000 | .000 | .000 | .000 | .000 | .000 | .000 | .000 | .000 | .000 | .950 | .000 |
| ESE\_7 | .000 | .000 | .000 | .000 | .000 | .000 | .000 | .000 | .000 | .000 | .000 | 1.000 | .000 |
| ESE\_6 | .000 | .000 | .000 | .000 | .000 | .000 | .000 | .000 | .000 | .000 | .000 | .000 | .968 |
| ESE\_5 | .000 | .000 | .000 | .000 | .000 | .000 | .000 | .000 | .000 | .000 | .000 | .000 | .973 |
| ESE\_4 | .000 | .000 | .000 | .000 | .000 | .000 | .000 | .000 | .000 | .000 | .000 | .000 | .987 |
| ESE\_3 | .000 | .000 | .000 | .000 | .000 | .000 | .000 | .000 | .000 | .000 | .000 | .000 | .923 |
| ESE\_2 | .000 | .000 | .000 | .000 | .000 | .000 | .000 | .000 | .000 | .000 | .000 | .000 | .969 |
| ESE\_1 | .000 | .000 | .000 | .000 | .000 | .000 | .000 | .000 | .000 | .000 | .000 | .000 | 1.000 |
| EOE\_9 | .000 | .000 | .000 | .000 | .943 | .000 | .000 | .000 | .000 | .000 | .000 | .000 | .000 |
| EOE\_8 | .000 | .000 | .000 | .000 | .968 | .000 | .000 | .000 | .000 | .000 | .000 | .000 | .000 |
| EOE\_7 | .000 | .000 | .000 | .000 | .974 | .000 | .000 | .000 | .000 | .000 | .000 | .000 | .000 |
| EOE\_6 | .000 | .000 | .000 | .000 | .937 | .000 | .000 | .000 | .000 | .000 | .000 | .000 | .000 |
| EOE\_5 | .000 | .000 | .000 | .000 | .975 | .000 | .000 | .000 | .000 | .000 | .000 | .000 | .000 |
| EOE\_4 | .000 | .000 | .000 | .000 | .938 | .000 | .000 | .000 | .000 | .000 | .000 | .000 | .000 |
| EOE\_3 | .000 | .000 | .000 | .000 | .949 | .000 | .000 | .000 | .000 | .000 | .000 | .000 | .000 |
| EOE\_2 | .000 | .000 | .000 | .000 | .960 | .000 | .000 | .000 | .000 | .000 | .000 | .000 | .000 |
| EOE\_1 | .000 | .000 | .000 | .000 | 1.000 | .000 | .000 | .000 | .000 | .000 | .000 | .000 | .000 |
| AISE\_6 | .000 | .000 | .000 | .948 | .000 | .000 | .000 | .000 | .000 | .000 | .000 | .000 | .000 |
| AISE\_5 | .000 | .000 | .000 | .905 | .000 | .000 | .000 | .000 | .000 | .000 | .000 | .000 | .000 |
| AISE\_4 | .000 | .000 | .000 | .940 | .000 | .000 | .000 | .000 | .000 | .000 | .000 | .000 | .000 |
| AISE\_3 | .000 | .000 | .000 | .909 | .000 | .000 | .000 | .000 | .000 | .000 | .000 | .000 | .000 |
| AISE\_2 | .000 | .000 | .000 | .932 | .000 | .000 | .000 | .000 | .000 | .000 | .000 | .000 | .000 |
| AISE\_1 | .000 | .000 | .000 | 1.000 | .000 | .000 | .000 | .000 | .000 | .000 | .000 | .000 | .000 |

## Direct Effects - Upper Bounds (PC) (Group number 1 - Default model)

|  | Grade | Age | Gender | AISE | EOE | ESE | EE | Pa | Vc | Fs | Vp | Ss | Sm |
| --- | --- | --- | --- | --- | --- | --- | --- | --- | --- | --- | --- | --- | --- |
| EOE | .039 | .042 | .004 | .396 | .000 | .000 | .000 | .000 | .000 | .000 | .000 | .000 | .000 |
| ESE | .031 | .020 | -.005 | .277 | .309 | .000 | .000 | .000 | .000 | .000 | .000 | .000 | .000 |
| EE | .034 | .035 | -.013 | .179 | .224 | .308 | .000 | .000 | .000 | .000 | .000 | .000 | .000 |
| Pa | .000 | .000 | .000 | .000 | .000 | .000 | 1.067 | .000 | .000 | .000 | .000 | .000 | .000 |
| Vc | .000 | .000 | .000 | .000 | .000 | .000 | 1.156 | .000 | .000 | .000 | .000 | .000 | .000 |
| Fs | .000 | .000 | .000 | .000 | .000 | .000 | 1.124 | .000 | .000 | .000 | .000 | .000 | .000 |
| Vp | .000 | .000 | .000 | .000 | .000 | .000 | 1.000 | .000 | .000 | .000 | .000 | .000 | .000 |
| Ss | .000 | .000 | .000 | .000 | .000 | 1.095 | .000 | .000 | .000 | .000 | .000 | .000 | .000 |
| Sm | .000 | .000 | .000 | .000 | .000 | 1.000 | .000 | .000 | .000 | .000 | .000 | .000 | .000 |
| EE\_20 | .000 | .000 | .000 | .000 | .000 | .000 | .000 | 1.053 | .000 | .000 | .000 | .000 | .000 |
| EE\_19 | .000 | .000 | .000 | .000 | .000 | .000 | .000 | 1.036 | .000 | .000 | .000 | .000 | .000 |
| EE\_18 | .000 | .000 | .000 | .000 | .000 | .000 | .000 | 1.000 | .000 | .000 | .000 | .000 | .000 |
| EE\_17 | .000 | .000 | .000 | .000 | .000 | .000 | .000 | .000 | 1.028 | .000 | .000 | .000 | .000 |
| EE\_16 | .000 | .000 | .000 | .000 | .000 | .000 | .000 | .000 | 1.033 | .000 | .000 | .000 | .000 |
| EE\_15 | .000 | .000 | .000 | .000 | .000 | .000 | .000 | .000 | 1.044 | .000 | .000 | .000 | .000 |
| EE\_14 | .000 | .000 | .000 | .000 | .000 | .000 | .000 | .000 | 1.028 | .000 | .000 | .000 | .000 |
| EE\_13 | .000 | .000 | .000 | .000 | .000 | .000 | .000 | .000 | 1.000 | .000 | .000 | .000 | .000 |
| EE\_12 | .000 | .000 | .000 | .000 | .000 | .000 | .000 | .000 | .000 | 1.056 | .000 | .000 | .000 |
| EE\_11 | .000 | .000 | .000 | .000 | .000 | .000 | .000 | .000 | .000 | 1.063 | .000 | .000 | .000 |
| EE\_10 | .000 | .000 | .000 | .000 | .000 | .000 | .000 | .000 | .000 | 1.068 | .000 | .000 | .000 |
| EE\_9 | .000 | .000 | .000 | .000 | .000 | .000 | .000 | .000 | .000 | 1.065 | .000 | .000 | .000 |
| EE\_8 | .000 | .000 | .000 | .000 | .000 | .000 | .000 | .000 | .000 | 1.035 | .000 | .000 | .000 |
| EE\_7 | .000 | .000 | .000 | .000 | .000 | .000 | .000 | .000 | .000 | 1.000 | .000 | .000 | .000 |
| EE\_6 | .000 | .000 | .000 | .000 | .000 | .000 | .000 | .000 | .000 | .000 | 1.102 | .000 | .000 |
| EE\_5 | .000 | .000 | .000 | .000 | .000 | .000 | .000 | .000 | .000 | .000 | 1.090 | .000 | .000 |
| EE\_4 | .000 | .000 | .000 | .000 | .000 | .000 | .000 | .000 | .000 | .000 | 1.088 | .000 | .000 |
| EE\_3 | .000 | .000 | .000 | .000 | .000 | .000 | .000 | .000 | .000 | .000 | 1.110 | .000 | .000 |
| EE\_2 | .000 | .000 | .000 | .000 | .000 | .000 | .000 | .000 | .000 | .000 | 1.084 | .000 | .000 |
| EE\_1 | .000 | .000 | .000 | .000 | .000 | .000 | .000 | .000 | .000 | .000 | 1.000 | .000 | .000 |
| ESE\_10 | .000 | .000 | .000 | .000 | .000 | .000 | .000 | .000 | .000 | .000 | .000 | 1.042 | .000 |
| ESE\_9 | .000 | .000 | .000 | .000 | .000 | .000 | .000 | .000 | .000 | .000 | .000 | 1.004 | .000 |
| ESE\_8 | .000 | .000 | .000 | .000 | .000 | .000 | .000 | .000 | .000 | .000 | .000 | 1.063 | .000 |
| ESE\_7 | .000 | .000 | .000 | .000 | .000 | .000 | .000 | .000 | .000 | .000 | .000 | 1.000 | .000 |
| ESE\_6 | .000 | .000 | .000 | .000 | .000 | .000 | .000 | .000 | .000 | .000 | .000 | .000 | 1.092 |
| ESE\_5 | .000 | .000 | .000 | .000 | .000 | .000 | .000 | .000 | .000 | .000 | .000 | .000 | 1.097 |
| ESE\_4 | .000 | .000 | .000 | .000 | .000 | .000 | .000 | .000 | .000 | .000 | .000 | .000 | 1.111 |
| ESE\_3 | .000 | .000 | .000 | .000 | .000 | .000 | .000 | .000 | .000 | .000 | .000 | .000 | 1.046 |
| ESE\_2 | .000 | .000 | .000 | .000 | .000 | .000 | .000 | .000 | .000 | .000 | .000 | .000 | 1.091 |
| ESE\_1 | .000 | .000 | .000 | .000 | .000 | .000 | .000 | .000 | .000 | .000 | .000 | .000 | 1.000 |
| EOE\_9 | .000 | .000 | .000 | .000 | 1.061 | .000 | .000 | .000 | .000 | .000 | .000 | .000 | .000 |
| EOE\_8 | .000 | .000 | .000 | .000 | 1.085 | .000 | .000 | .000 | .000 | .000 | .000 | .000 | .000 |
| EOE\_7 | .000 | .000 | .000 | .000 | 1.092 | .000 | .000 | .000 | .000 | .000 | .000 | .000 | .000 |
| EOE\_6 | .000 | .000 | .000 | .000 | 1.051 | .000 | .000 | .000 | .000 | .000 | .000 | .000 | .000 |
| EOE\_5 | .000 | .000 | .000 | .000 | 1.093 | .000 | .000 | .000 | .000 | .000 | .000 | .000 | .000 |
| EOE\_4 | .000 | .000 | .000 | .000 | 1.051 | .000 | .000 | .000 | .000 | .000 | .000 | .000 | .000 |
| EOE\_3 | .000 | .000 | .000 | .000 | 1.059 | .000 | .000 | .000 | .000 | .000 | .000 | .000 | .000 |
| EOE\_2 | .000 | .000 | .000 | .000 | 1.070 | .000 | .000 | .000 | .000 | .000 | .000 | .000 | .000 |
| EOE\_1 | .000 | .000 | .000 | .000 | 1.000 | .000 | .000 | .000 | .000 | .000 | .000 | .000 | .000 |
| AISE\_6 | .000 | .000 | .000 | 1.052 | .000 | .000 | .000 | .000 | .000 | .000 | .000 | .000 | .000 |
| AISE\_5 | .000 | .000 | .000 | 1.009 | .000 | .000 | .000 | .000 | .000 | .000 | .000 | .000 | .000 |
| AISE\_4 | .000 | .000 | .000 | 1.046 | .000 | .000 | .000 | .000 | .000 | .000 | .000 | .000 | .000 |
| AISE\_3 | .000 | .000 | .000 | 1.012 | .000 | .000 | .000 | .000 | .000 | .000 | .000 | .000 | .000 |
| AISE\_2 | .000 | .000 | .000 | 1.036 | .000 | .000 | .000 | .000 | .000 | .000 | .000 | .000 | .000 |
| AISE\_1 | .000 | .000 | .000 | 1.000 | .000 | .000 | .000 | .000 | .000 | .000 | .000 | .000 | .000 |

## Direct Effects - Two Tailed Significance (PC) (Group number 1 - Default model)

|  | Grade | Age | Gender | AISE | EOE | ESE | EE | Pa | Vc | Fs | Vp | Ss | Sm |
| --- | --- | --- | --- | --- | --- | --- | --- | --- | --- | --- | --- | --- | --- |
| EOE | .148 | .084 | .075 | .000 | ... | ... | ... | ... | ... | ... | ... | ... | ... |
| ESE | .396 | .971 | .027 | .000 | .000 | ... | ... | ... | ... | ... | ... | ... | ... |
| EE | .170 | .095 | .007 | .000 | .000 | .000 | ... | ... | ... | ... | ... | ... | ... |
| Pa | ... | ... | ... | ... | ... | ... | .000 | ... | ... | ... | ... | ... | ... |
| Vc | ... | ... | ... | ... | ... | ... | .000 | ... | ... | ... | ... | ... | ... |
| Fs | ... | ... | ... | ... | ... | ... | .000 | ... | ... | ... | ... | ... | ... |
| Vp | ... | ... | ... | ... | ... | ... | ... | ... | ... | ... | ... | ... | ... |
| Ss | ... | ... | ... | ... | ... | .000 | ... | ... | ... | ... | ... | ... | ... |
| Sm | ... | ... | ... | ... | ... | ... | ... | ... | ... | ... | ... | ... | ... |
| EE\_20 | ... | ... | ... | ... | ... | ... | ... | .000 | ... | ... | ... | ... | ... |
| EE\_19 | ... | ... | ... | ... | ... | ... | ... | .000 | ... | ... | ... | ... | ... |
| EE\_18 | ... | ... | ... | ... | ... | ... | ... | ... | ... | ... | ... | ... | ... |
| EE\_17 | ... | ... | ... | ... | ... | ... | ... | ... | .000 | ... | ... | ... | ... |
| EE\_16 | ... | ... | ... | ... | ... | ... | ... | ... | .000 | ... | ... | ... | ... |
| EE\_15 | ... | ... | ... | ... | ... | ... | ... | ... | .000 | ... | ... | ... | ... |
| EE\_14 | ... | ... | ... | ... | ... | ... | ... | ... | .000 | ... | ... | ... | ... |
| EE\_13 | ... | ... | ... | ... | ... | ... | ... | ... | ... | ... | ... | ... | ... |
| EE\_12 | ... | ... | ... | ... | ... | ... | ... | ... | ... | .000 | ... | ... | ... |
| EE\_11 | ... | ... | ... | ... | ... | ... | ... | ... | ... | .000 | ... | ... | ... |
| EE\_10 | ... | ... | ... | ... | ... | ... | ... | ... | ... | .000 | ... | ... | ... |
| EE\_9 | ... | ... | ... | ... | ... | ... | ... | ... | ... | .000 | ... | ... | ... |
| EE\_8 | ... | ... | ... | ... | ... | ... | ... | ... | ... | .000 | ... | ... | ... |
| EE\_7 | ... | ... | ... | ... | ... | ... | ... | ... | ... | ... | ... | ... | ... |
| EE\_6 | ... | ... | ... | ... | ... | ... | ... | ... | ... | ... | .000 | ... | ... |
| EE\_5 | ... | ... | ... | ... | ... | ... | ... | ... | ... | ... | .000 | ... | ... |
| EE\_4 | ... | ... | ... | ... | ... | ... | ... | ... | ... | ... | .000 | ... | ... |
| EE\_3 | ... | ... | ... | ... | ... | ... | ... | ... | ... | ... | .000 | ... | ... |
| EE\_2 | ... | ... | ... | ... | ... | ... | ... | ... | ... | ... | .000 | ... | ... |
| EE\_1 | ... | ... | ... | ... | ... | ... | ... | ... | ... | ... | ... | ... | ... |
| ESE\_10 | ... | ... | ... | ... | ... | ... | ... | ... | ... | ... | ... | .000 | ... |
| ESE\_9 | ... | ... | ... | ... | ... | ... | ... | ... | ... | ... | ... | .000 | ... |
| ESE\_8 | ... | ... | ... | ... | ... | ... | ... | ... | ... | ... | ... | .000 | ... |
| ESE\_7 | ... | ... | ... | ... | ... | ... | ... | ... | ... | ... | ... | ... | ... |
| ESE\_6 | ... | ... | ... | ... | ... | ... | ... | ... | ... | ... | ... | ... | .000 |
| ESE\_5 | ... | ... | ... | ... | ... | ... | ... | ... | ... | ... | ... | ... | .000 |
| ESE\_4 | ... | ... | ... | ... | ... | ... | ... | ... | ... | ... | ... | ... | .000 |
| ESE\_3 | ... | ... | ... | ... | ... | ... | ... | ... | ... | ... | ... | ... | .000 |
| ESE\_2 | ... | ... | ... | ... | ... | ... | ... | ... | ... | ... | ... | ... | .000 |
| ESE\_1 | ... | ... | ... | ... | ... | ... | ... | ... | ... | ... | ... | ... | ... |
| EOE\_9 | ... | ... | ... | ... | .000 | ... | ... | ... | ... | ... | ... | ... | ... |
| EOE\_8 | ... | ... | ... | ... | .000 | ... | ... | ... | ... | ... | ... | ... | ... |
| EOE\_7 | ... | ... | ... | ... | .000 | ... | ... | ... | ... | ... | ... | ... | ... |
| EOE\_6 | ... | ... | ... | ... | .000 | ... | ... | ... | ... | ... | ... | ... | ... |
| EOE\_5 | ... | ... | ... | ... | .000 | ... | ... | ... | ... | ... | ... | ... | ... |
| EOE\_4 | ... | ... | ... | ... | .000 | ... | ... | ... | ... | ... | ... | ... | ... |
| EOE\_3 | ... | ... | ... | ... | .000 | ... | ... | ... | ... | ... | ... | ... | ... |
| EOE\_2 | ... | ... | ... | ... | .000 | ... | ... | ... | ... | ... | ... | ... | ... |
| EOE\_1 | ... | ... | ... | ... | ... | ... | ... | ... | ... | ... | ... | ... | ... |
| AISE\_6 | ... | ... | ... | .000 | ... | ... | ... | ... | ... | ... | ... | ... | ... |
| AISE\_5 | ... | ... | ... | .000 | ... | ... | ... | ... | ... | ... | ... | ... | ... |
| AISE\_4 | ... | ... | ... | .000 | ... | ... | ... | ... | ... | ... | ... | ... | ... |
| AISE\_3 | ... | ... | ... | .000 | ... | ... | ... | ... | ... | ... | ... | ... | ... |
| AISE\_2 | ... | ... | ... | .000 | ... | ... | ... | ... | ... | ... | ... | ... | ... |
| AISE\_1 | ... | ... | ... | ... | ... | ... | ... | ... | ... | ... | ... | ... | ... |

## Standardized Direct Effects (Group number 1 - Default model)

## Standardized Direct Effects - Lower Bounds (PC) (Group number 1 - Default model)

|  | Grade | Age | Gender | AISE | EOE | ESE | EE | Pa | Vc | Fs | Vp | Ss | Sm |
| --- | --- | --- | --- | --- | --- | --- | --- | --- | --- | --- | --- | --- | --- |
| EOE | -.011 | -.003 | -.073 | .513 | .000 | .000 | .000 | .000 | .000 | .000 | .000 | .000 | .000 |
| ESE | -.024 | -.042 | -.093 | .374 | .248 | .000 | .000 | .000 | .000 | .000 | .000 | .000 | .000 |
| EE | -.012 | -.006 | -.092 | .188 | .153 | .163 | .000 | .000 | .000 | .000 | .000 | .000 | .000 |
| Pa | .000 | .000 | .000 | .000 | .000 | .000 | .775 | .000 | .000 | .000 | .000 | .000 | .000 |
| Vc | .000 | .000 | .000 | .000 | .000 | .000 | .801 | .000 | .000 | .000 | .000 | .000 | .000 |
| Fs | .000 | .000 | .000 | .000 | .000 | .000 | .789 | .000 | .000 | .000 | .000 | .000 | .000 |
| Vp | .000 | .000 | .000 | .000 | .000 | .000 | .783 | .000 | .000 | .000 | .000 | .000 | .000 |
| Ss | .000 | .000 | .000 | .000 | .000 | .748 | .000 | .000 | .000 | .000 | .000 | .000 | .000 |
| Sm | .000 | .000 | .000 | .000 | .000 | .777 | .000 | .000 | .000 | .000 | .000 | .000 | .000 |
| EE\_20 | .000 | .000 | .000 | .000 | .000 | .000 | .000 | .696 | .000 | .000 | .000 | .000 | .000 |
| EE\_19 | .000 | .000 | .000 | .000 | .000 | .000 | .000 | .688 | .000 | .000 | .000 | .000 | .000 |
| EE\_18 | .000 | .000 | .000 | .000 | .000 | .000 | .000 | .711 | .000 | .000 | .000 | .000 | .000 |
| EE\_17 | .000 | .000 | .000 | .000 | .000 | .000 | .000 | .000 | .714 | .000 | .000 | .000 | .000 |
| EE\_16 | .000 | .000 | .000 | .000 | .000 | .000 | .000 | .000 | .713 | .000 | .000 | .000 | .000 |
| EE\_15 | .000 | .000 | .000 | .000 | .000 | .000 | .000 | .000 | .726 | .000 | .000 | .000 | .000 |
| EE\_14 | .000 | .000 | .000 | .000 | .000 | .000 | .000 | .000 | .711 | .000 | .000 | .000 | .000 |
| EE\_13 | .000 | .000 | .000 | .000 | .000 | .000 | .000 | .000 | .728 | .000 | .000 | .000 | .000 |
| EE\_12 | .000 | .000 | .000 | .000 | .000 | .000 | .000 | .000 | .000 | .712 | .000 | .000 | .000 |
| EE\_11 | .000 | .000 | .000 | .000 | .000 | .000 | .000 | .000 | .000 | .713 | .000 | .000 | .000 |
| EE\_10 | .000 | .000 | .000 | .000 | .000 | .000 | .000 | .000 | .000 | .715 | .000 | .000 | .000 |
| EE\_9 | .000 | .000 | .000 | .000 | .000 | .000 | .000 | .000 | .000 | .717 | .000 | .000 | .000 |
| EE\_8 | .000 | .000 | .000 | .000 | .000 | .000 | .000 | .000 | .000 | .691 | .000 | .000 | .000 |
| EE\_7 | .000 | .000 | .000 | .000 | .000 | .000 | .000 | .000 | .000 | .707 | .000 | .000 | .000 |
| EE\_6 | .000 | .000 | .000 | .000 | .000 | .000 | .000 | .000 | .000 | .000 | .718 | .000 | .000 |
| EE\_5 | .000 | .000 | .000 | .000 | .000 | .000 | .000 | .000 | .000 | .000 | .703 | .000 | .000 |
| EE\_4 | .000 | .000 | .000 | .000 | .000 | .000 | .000 | .000 | .000 | .000 | .710 | .000 | .000 |
| EE\_3 | .000 | .000 | .000 | .000 | .000 | .000 | .000 | .000 | .000 | .000 | .722 | .000 | .000 |
| EE\_2 | .000 | .000 | .000 | .000 | .000 | .000 | .000 | .000 | .000 | .000 | .698 | .000 | .000 |
| EE\_1 | .000 | .000 | .000 | .000 | .000 | .000 | .000 | .000 | .000 | .000 | .694 | .000 | .000 |
| ESE\_10 | .000 | .000 | .000 | .000 | .000 | .000 | .000 | .000 | .000 | .000 | .000 | .708 | .000 |
| ESE\_9 | .000 | .000 | .000 | .000 | .000 | .000 | .000 | .000 | .000 | .000 | .000 | .692 | .000 |
| ESE\_8 | .000 | .000 | .000 | .000 | .000 | .000 | .000 | .000 | .000 | .000 | .000 | .724 | .000 |
| ESE\_7 | .000 | .000 | .000 | .000 | .000 | .000 | .000 | .000 | .000 | .000 | .000 | .722 | .000 |
| ESE\_6 | .000 | .000 | .000 | .000 | .000 | .000 | .000 | .000 | .000 | .000 | .000 | .000 | .697 |
| ESE\_5 | .000 | .000 | .000 | .000 | .000 | .000 | .000 | .000 | .000 | .000 | .000 | .000 | .717 |
| ESE\_4 | .000 | .000 | .000 | .000 | .000 | .000 | .000 | .000 | .000 | .000 | .000 | .000 | .716 |
| ESE\_3 | .000 | .000 | .000 | .000 | .000 | .000 | .000 | .000 | .000 | .000 | .000 | .000 | .688 |
| ESE\_2 | .000 | .000 | .000 | .000 | .000 | .000 | .000 | .000 | .000 | .000 | .000 | .000 | .710 |
| ESE\_1 | .000 | .000 | .000 | .000 | .000 | .000 | .000 | .000 | .000 | .000 | .000 | .000 | .688 |
| EOE\_9 | .000 | .000 | .000 | .000 | .697 | .000 | .000 | .000 | .000 | .000 | .000 | .000 | .000 |
| EOE\_8 | .000 | .000 | .000 | .000 | .707 | .000 | .000 | .000 | .000 | .000 | .000 | .000 | .000 |
| EOE\_7 | .000 | .000 | .000 | .000 | .726 | .000 | .000 | .000 | .000 | .000 | .000 | .000 | .000 |
| EOE\_6 | .000 | .000 | .000 | .000 | .694 | .000 | .000 | .000 | .000 | .000 | .000 | .000 | .000 |
| EOE\_5 | .000 | .000 | .000 | .000 | .717 | .000 | .000 | .000 | .000 | .000 | .000 | .000 | .000 |
| EOE\_4 | .000 | .000 | .000 | .000 | .703 | .000 | .000 | .000 | .000 | .000 | .000 | .000 | .000 |
| EOE\_3 | .000 | .000 | .000 | .000 | .699 | .000 | .000 | .000 | .000 | .000 | .000 | .000 | .000 |
| EOE\_2 | .000 | .000 | .000 | .000 | .705 | .000 | .000 | .000 | .000 | .000 | .000 | .000 | .000 |
| EOE\_1 | .000 | .000 | .000 | .000 | .698 | .000 | .000 | .000 | .000 | .000 | .000 | .000 | .000 |
| AISE\_6 | .000 | .000 | .000 | .731 | .000 | .000 | .000 | .000 | .000 | .000 | .000 | .000 | .000 |
| AISE\_5 | .000 | .000 | .000 | .739 | .000 | .000 | .000 | .000 | .000 | .000 | .000 | .000 | .000 |
| AISE\_4 | .000 | .000 | .000 | .726 | .000 | .000 | .000 | .000 | .000 | .000 | .000 | .000 | .000 |
| AISE\_3 | .000 | .000 | .000 | .714 | .000 | .000 | .000 | .000 | .000 | .000 | .000 | .000 | .000 |
| AISE\_2 | .000 | .000 | .000 | .736 | .000 | .000 | .000 | .000 | .000 | .000 | .000 | .000 | .000 |
| AISE\_1 | .000 | .000 | .000 | .698 | .000 | .000 | .000 | .000 | .000 | .000 | .000 | .000 | .000 |

## Standardized Direct Effects - Upper Bounds (PC) (Group number 1 - Default model)

|  | Grade | Age | Gender | AISE | EOE | ESE | EE | Pa | Vc | Fs | Vp | Ss | Sm |
| --- | --- | --- | --- | --- | --- | --- | --- | --- | --- | --- | --- | --- | --- |
| EOE | .064 | .073 | .003 | .581 | .000 | .000 | .000 | .000 | .000 | .000 | .000 | .000 | .000 |
| ESE | .060 | .041 | -.006 | .480 | .355 | .000 | .000 | .000 | .000 | .000 | .000 | .000 | .000 |
| EE | .066 | .071 | -.014 | .309 | .260 | .300 | .000 | .000 | .000 | .000 | .000 | .000 | .000 |
| Pa | .000 | .000 | .000 | .000 | .000 | .000 | .831 | .000 | .000 | .000 | .000 | .000 | .000 |
| Vc | .000 | .000 | .000 | .000 | .000 | .000 | .846 | .000 | .000 | .000 | .000 | .000 | .000 |
| Fs | .000 | .000 | .000 | .000 | .000 | .000 | .836 | .000 | .000 | .000 | .000 | .000 | .000 |
| Vp | .000 | .000 | .000 | .000 | .000 | .000 | .830 | .000 | .000 | .000 | .000 | .000 | .000 |
| Ss | .000 | .000 | .000 | .000 | .000 | .819 | .000 | .000 | .000 | .000 | .000 | .000 | .000 |
| Sm | .000 | .000 | .000 | .000 | .000 | .845 | .000 | .000 | .000 | .000 | .000 | .000 | .000 |
| EE\_20 | .000 | .000 | .000 | .000 | .000 | .000 | .000 | .748 | .000 | .000 | .000 | .000 | .000 |
| EE\_19 | .000 | .000 | .000 | .000 | .000 | .000 | .000 | .740 | .000 | .000 | .000 | .000 | .000 |
| EE\_18 | .000 | .000 | .000 | .000 | .000 | .000 | .000 | .762 | .000 | .000 | .000 | .000 | .000 |
| EE\_17 | .000 | .000 | .000 | .000 | .000 | .000 | .000 | .000 | .760 | .000 | .000 | .000 | .000 |
| EE\_16 | .000 | .000 | .000 | .000 | .000 | .000 | .000 | .000 | .757 | .000 | .000 | .000 | .000 |
| EE\_15 | .000 | .000 | .000 | .000 | .000 | .000 | .000 | .000 | .769 | .000 | .000 | .000 | .000 |
| EE\_14 | .000 | .000 | .000 | .000 | .000 | .000 | .000 | .000 | .755 | .000 | .000 | .000 | .000 |
| EE\_13 | .000 | .000 | .000 | .000 | .000 | .000 | .000 | .000 | .771 | .000 | .000 | .000 | .000 |
| EE\_12 | .000 | .000 | .000 | .000 | .000 | .000 | .000 | .000 | .000 | .756 | .000 | .000 | .000 |
| EE\_11 | .000 | .000 | .000 | .000 | .000 | .000 | .000 | .000 | .000 | .758 | .000 | .000 | .000 |
| EE\_10 | .000 | .000 | .000 | .000 | .000 | .000 | .000 | .000 | .000 | .757 | .000 | .000 | .000 |
| EE\_9 | .000 | .000 | .000 | .000 | .000 | .000 | .000 | .000 | .000 | .761 | .000 | .000 | .000 |
| EE\_8 | .000 | .000 | .000 | .000 | .000 | .000 | .000 | .000 | .000 | .737 | .000 | .000 | .000 |
| EE\_7 | .000 | .000 | .000 | .000 | .000 | .000 | .000 | .000 | .000 | .750 | .000 | .000 | .000 |
| EE\_6 | .000 | .000 | .000 | .000 | .000 | .000 | .000 | .000 | .000 | .000 | .761 | .000 | .000 |
| EE\_5 | .000 | .000 | .000 | .000 | .000 | .000 | .000 | .000 | .000 | .000 | .747 | .000 | .000 |
| EE\_4 | .000 | .000 | .000 | .000 | .000 | .000 | .000 | .000 | .000 | .000 | .753 | .000 | .000 |
| EE\_3 | .000 | .000 | .000 | .000 | .000 | .000 | .000 | .000 | .000 | .000 | .764 | .000 | .000 |
| EE\_2 | .000 | .000 | .000 | .000 | .000 | .000 | .000 | .000 | .000 | .000 | .744 | .000 | .000 |
| EE\_1 | .000 | .000 | .000 | .000 | .000 | .000 | .000 | .000 | .000 | .000 | .742 | .000 | .000 |
| ESE\_10 | .000 | .000 | .000 | .000 | .000 | .000 | .000 | .000 | .000 | .000 | .000 | .758 | .000 |
| ESE\_9 | .000 | .000 | .000 | .000 | .000 | .000 | .000 | .000 | .000 | .000 | .000 | .740 | .000 |
| ESE\_8 | .000 | .000 | .000 | .000 | .000 | .000 | .000 | .000 | .000 | .000 | .000 | .772 | .000 |
| ESE\_7 | .000 | .000 | .000 | .000 | .000 | .000 | .000 | .000 | .000 | .000 | .000 | .766 | .000 |
| ESE\_6 | .000 | .000 | .000 | .000 | .000 | .000 | .000 | .000 | .000 | .000 | .000 | .000 | .743 |
| ESE\_5 | .000 | .000 | .000 | .000 | .000 | .000 | .000 | .000 | .000 | .000 | .000 | .000 | .760 |
| ESE\_4 | .000 | .000 | .000 | .000 | .000 | .000 | .000 | .000 | .000 | .000 | .000 | .000 | .760 |
| ESE\_3 | .000 | .000 | .000 | .000 | .000 | .000 | .000 | .000 | .000 | .000 | .000 | .000 | .736 |
| ESE\_2 | .000 | .000 | .000 | .000 | .000 | .000 | .000 | .000 | .000 | .000 | .000 | .000 | .757 |
| ESE\_1 | .000 | .000 | .000 | .000 | .000 | .000 | .000 | .000 | .000 | .000 | .000 | .000 | .735 |
| EOE\_9 | .000 | .000 | .000 | .000 | .740 | .000 | .000 | .000 | .000 | .000 | .000 | .000 | .000 |
| EOE\_8 | .000 | .000 | .000 | .000 | .749 | .000 | .000 | .000 | .000 | .000 | .000 | .000 | .000 |
| EOE\_7 | .000 | .000 | .000 | .000 | .765 | .000 | .000 | .000 | .000 | .000 | .000 | .000 | .000 |
| EOE\_6 | .000 | .000 | .000 | .000 | .738 | .000 | .000 | .000 | .000 | .000 | .000 | .000 | .000 |
| EOE\_5 | .000 | .000 | .000 | .000 | .758 | .000 | .000 | .000 | .000 | .000 | .000 | .000 | .000 |
| EOE\_4 | .000 | .000 | .000 | .000 | .744 | .000 | .000 | .000 | .000 | .000 | .000 | .000 | .000 |
| EOE\_3 | .000 | .000 | .000 | .000 | .742 | .000 | .000 | .000 | .000 | .000 | .000 | .000 | .000 |
| EOE\_2 | .000 | .000 | .000 | .000 | .749 | .000 | .000 | .000 | .000 | .000 | .000 | .000 | .000 |
| EOE\_1 | .000 | .000 | .000 | .000 | .741 | .000 | .000 | .000 | .000 | .000 | .000 | .000 | .000 |
| AISE\_6 | .000 | .000 | .000 | .773 | .000 | .000 | .000 | .000 | .000 | .000 | .000 | .000 | .000 |
| AISE\_5 | .000 | .000 | .000 | .780 | .000 | .000 | .000 | .000 | .000 | .000 | .000 | .000 | .000 |
| AISE\_4 | .000 | .000 | .000 | .768 | .000 | .000 | .000 | .000 | .000 | .000 | .000 | .000 | .000 |
| AISE\_3 | .000 | .000 | .000 | .757 | .000 | .000 | .000 | .000 | .000 | .000 | .000 | .000 | .000 |
| AISE\_2 | .000 | .000 | .000 | .776 | .000 | .000 | .000 | .000 | .000 | .000 | .000 | .000 | .000 |
| AISE\_1 | .000 | .000 | .000 | .743 | .000 | .000 | .000 | .000 | .000 | .000 | .000 | .000 | .000 |

## Standardized Direct Effects - Two Tailed Significance (PC) (Group number 1 - Default model)

|  | Grade | Age | Gender | AISE | EOE | ESE | EE | Pa | Vc | Fs | Vp | Ss | Sm |
| --- | --- | --- | --- | --- | --- | --- | --- | --- | --- | --- | --- | --- | --- |
| EOE | .148 | .084 | .075 | .000 | ... | ... | ... | ... | ... | ... | ... | ... | ... |
| ESE | .396 | .971 | .027 | .000 | .000 | ... | ... | ... | ... | ... | ... | ... | ... |
| EE | .170 | .095 | .007 | .000 | .000 | .000 | ... | ... | ... | ... | ... | ... | ... |
| Pa | ... | ... | ... | ... | ... | ... | .000 | ... | ... | ... | ... | ... | ... |
| Vc | ... | ... | ... | ... | ... | ... | .000 | ... | ... | ... | ... | ... | ... |
| Fs | ... | ... | ... | ... | ... | ... | .000 | ... | ... | ... | ... | ... | ... |
| Vp | ... | ... | ... | ... | ... | ... | .000 | ... | ... | ... | ... | ... | ... |
| Ss | ... | ... | ... | ... | ... | .000 | ... | ... | ... | ... | ... | ... | ... |
| Sm | ... | ... | ... | ... | ... | .000 | ... | ... | ... | ... | ... | ... | ... |
| EE\_20 | ... | ... | ... | ... | ... | ... | ... | .000 | ... | ... | ... | ... | ... |
| EE\_19 | ... | ... | ... | ... | ... | ... | ... | .000 | ... | ... | ... | ... | ... |
| EE\_18 | ... | ... | ... | ... | ... | ... | ... | .000 | ... | ... | ... | ... | ... |
| EE\_17 | ... | ... | ... | ... | ... | ... | ... | ... | .000 | ... | ... | ... | ... |
| EE\_16 | ... | ... | ... | ... | ... | ... | ... | ... | .000 | ... | ... | ... | ... |
| EE\_15 | ... | ... | ... | ... | ... | ... | ... | ... | .000 | ... | ... | ... | ... |
| EE\_14 | ... | ... | ... | ... | ... | ... | ... | ... | .000 | ... | ... | ... | ... |
| EE\_13 | ... | ... | ... | ... | ... | ... | ... | ... | .000 | ... | ... | ... | ... |
| EE\_12 | ... | ... | ... | ... | ... | ... | ... | ... | ... | .000 | ... | ... | ... |
| EE\_11 | ... | ... | ... | ... | ... | ... | ... | ... | ... | .000 | ... | ... | ... |
| EE\_10 | ... | ... | ... | ... | ... | ... | ... | ... | ... | .000 | ... | ... | ... |
| EE\_9 | ... | ... | ... | ... | ... | ... | ... | ... | ... | .000 | ... | ... | ... |
| EE\_8 | ... | ... | ... | ... | ... | ... | ... | ... | ... | .000 | ... | ... | ... |
| EE\_7 | ... | ... | ... | ... | ... | ... | ... | ... | ... | .000 | ... | ... | ... |
| EE\_6 | ... | ... | ... | ... | ... | ... | ... | ... | ... | ... | .000 | ... | ... |
| EE\_5 | ... | ... | ... | ... | ... | ... | ... | ... | ... | ... | .000 | ... | ... |
| EE\_4 | ... | ... | ... | ... | ... | ... | ... | ... | ... | ... | .000 | ... | ... |
| EE\_3 | ... | ... | ... | ... | ... | ... | ... | ... | ... | ... | .000 | ... | ... |
| EE\_2 | ... | ... | ... | ... | ... | ... | ... | ... | ... | ... | .000 | ... | ... |
| EE\_1 | ... | ... | ... | ... | ... | ... | ... | ... | ... | ... | .000 | ... | ... |
| ESE\_10 | ... | ... | ... | ... | ... | ... | ... | ... | ... | ... | ... | .000 | ... |
| ESE\_9 | ... | ... | ... | ... | ... | ... | ... | ... | ... | ... | ... | .000 | ... |
| ESE\_8 | ... | ... | ... | ... | ... | ... | ... | ... | ... | ... | ... | .000 | ... |
| ESE\_7 | ... | ... | ... | ... | ... | ... | ... | ... | ... | ... | ... | .000 | ... |
| ESE\_6 | ... | ... | ... | ... | ... | ... | ... | ... | ... | ... | ... | ... | .000 |
| ESE\_5 | ... | ... | ... | ... | ... | ... | ... | ... | ... | ... | ... | ... | .000 |
| ESE\_4 | ... | ... | ... | ... | ... | ... | ... | ... | ... | ... | ... | ... | .000 |
| ESE\_3 | ... | ... | ... | ... | ... | ... | ... | ... | ... | ... | ... | ... | .000 |
| ESE\_2 | ... | ... | ... | ... | ... | ... | ... | ... | ... | ... | ... | ... | .000 |
| ESE\_1 | ... | ... | ... | ... | ... | ... | ... | ... | ... | ... | ... | ... | .000 |
| EOE\_9 | ... | ... | ... | ... | .000 | ... | ... | ... | ... | ... | ... | ... | ... |
| EOE\_8 | ... | ... | ... | ... | .000 | ... | ... | ... | ... | ... | ... | ... | ... |
| EOE\_7 | ... | ... | ... | ... | .000 | ... | ... | ... | ... | ... | ... | ... | ... |
| EOE\_6 | ... | ... | ... | ... | .000 | ... | ... | ... | ... | ... | ... | ... | ... |
| EOE\_5 | ... | ... | ... | ... | .000 | ... | ... | ... | ... | ... | ... | ... | ... |
| EOE\_4 | ... | ... | ... | ... | .000 | ... | ... | ... | ... | ... | ... | ... | ... |
| EOE\_3 | ... | ... | ... | ... | .000 | ... | ... | ... | ... | ... | ... | ... | ... |
| EOE\_2 | ... | ... | ... | ... | .000 | ... | ... | ... | ... | ... | ... | ... | ... |
| EOE\_1 | ... | ... | ... | ... | .000 | ... | ... | ... | ... | ... | ... | ... | ... |
| AISE\_6 | ... | ... | ... | .000 | ... | ... | ... | ... | ... | ... | ... | ... | ... |
| AISE\_5 | ... | ... | ... | .000 | ... | ... | ... | ... | ... | ... | ... | ... | ... |
| AISE\_4 | ... | ... | ... | .000 | ... | ... | ... | ... | ... | ... | ... | ... | ... |
| AISE\_3 | ... | ... | ... | .000 | ... | ... | ... | ... | ... | ... | ... | ... | ... |
| AISE\_2 | ... | ... | ... | .000 | ... | ... | ... | ... | ... | ... | ... | ... | ... |
| AISE\_1 | ... | ... | ... | .000 | ... | ... | ... | ... | ... | ... | ... | ... | ... |

## Indirect Effects (Group number 1 - Default model)

## Indirect Effects - Lower Bounds (PC) (Group number 1 - Default model)

|  | Grade | Age | Gender | AISE | EOE | ESE | EE | Pa | Vc | Fs | Vp | Ss | Sm |
| --- | --- | --- | --- | --- | --- | --- | --- | --- | --- | --- | --- | --- | --- |
| EOE | .000 | .000 | .000 | .000 | .000 | .000 | .000 | .000 | .000 | .000 | .000 | .000 | .000 |
| ESE | -.002 | -.001 | -.021 | .075 | .000 | .000 | .000 | .000 | .000 | .000 | .000 | .000 | .000 |
| EE | -.001 | -.003 | -.034 | .116 | .040 | .000 | .000 | .000 | .000 | .000 | .000 | .000 | .000 |
| Pa | -.001 | .001 | -.103 | .248 | .185 | .157 | .000 | .000 | .000 | .000 | .000 | .000 | .000 |
| Vc | -.001 | .001 | -.112 | .271 | .203 | .170 | .000 | .000 | .000 | .000 | .000 | .000 | .000 |
| Fs | -.001 | .001 | -.108 | .262 | .197 | .166 | .000 | .000 | .000 | .000 | .000 | .000 | .000 |
| Vp | -.001 | .001 | -.104 | .252 | .189 | .160 | .000 | .000 | .000 | .000 | .000 | .000 | .000 |
| Ss | -.009 | -.017 | -.094 | .301 | .208 | .000 | .000 | .000 | .000 | .000 | .000 | .000 | .000 |
| Sm | -.009 | -.017 | -.096 | .302 | .208 | .000 | .000 | .000 | .000 | .000 | .000 | .000 | .000 |
| EE\_20 | -.001 | .001 | -.102 | .246 | .184 | .155 | .891 | .000 | .000 | .000 | .000 | .000 | .000 |
| EE\_19 | -.001 | .001 | -.100 | .242 | .180 | .153 | .878 | .000 | .000 | .000 | .000 | .000 | .000 |
| EE\_18 | -.001 | .001 | -.103 | .248 | .185 | .157 | .896 | .000 | .000 | .000 | .000 | .000 | .000 |
| EE\_17 | -.001 | .001 | -.108 | .262 | .196 | .166 | .947 | .000 | .000 | .000 | .000 | .000 | .000 |
| EE\_16 | -.001 | .001 | -.108 | .265 | .197 | .166 | .956 | .000 | .000 | .000 | .000 | .000 | .000 |
| EE\_15 | -.001 | .001 | -.110 | .268 | .200 | .169 | .968 | .000 | .000 | .000 | .000 | .000 | .000 |
| EE\_14 | -.001 | .001 | -.108 | .263 | .197 | .166 | .953 | .000 | .000 | .000 | .000 | .000 | .000 |
| EE\_13 | -.001 | .001 | -.112 | .271 | .203 | .170 | .980 | .000 | .000 | .000 | .000 | .000 | .000 |
| EE\_12 | -.001 | .001 | -.107 | .261 | .195 | .165 | .946 | .000 | .000 | .000 | .000 | .000 | .000 |
| EE\_11 | -.001 | .001 | -.109 | .263 | .198 | .167 | .955 | .000 | .000 | .000 | .000 | .000 | .000 |
| EE\_10 | -.001 | .001 | -.109 | .264 | .198 | .168 | .958 | .000 | .000 | .000 | .000 | .000 | .000 |
| EE\_9 | -.001 | .001 | -.109 | .264 | .198 | .167 | .959 | .000 | .000 | .000 | .000 | .000 | .000 |
| EE\_8 | -.001 | .001 | -.105 | .254 | .191 | .161 | .924 | .000 | .000 | .000 | .000 | .000 | .000 |
| EE\_7 | -.001 | .001 | -.108 | .262 | .197 | .166 | .954 | .000 | .000 | .000 | .000 | .000 | .000 |
| EE\_6 | -.001 | .001 | -.109 | .263 | .197 | .167 | .979 | .000 | .000 | .000 | .000 | .000 | .000 |
| EE\_5 | -.001 | .001 | -.107 | .260 | .195 | .164 | .967 | .000 | .000 | .000 | .000 | .000 | .000 |
| EE\_4 | -.001 | .001 | -.107 | .260 | .195 | .164 | .963 | .000 | .000 | .000 | .000 | .000 | .000 |
| EE\_3 | -.001 | .001 | -.110 | .267 | .199 | .168 | .995 | .000 | .000 | .000 | .000 | .000 | .000 |
| EE\_2 | -.001 | .001 | -.107 | .259 | .193 | .165 | .962 | .000 | .000 | .000 | .000 | .000 | .000 |
| EE\_1 | -.001 | .001 | -.104 | .252 | .189 | .160 | 1.000 | .000 | .000 | .000 | .000 | .000 | .000 |
| ESE\_10 | -.008 | -.017 | -.092 | .293 | .204 | .879 | .000 | .000 | .000 | .000 | .000 | .000 | .000 |
| ESE\_9 | -.008 | -.016 | -.089 | .283 | .196 | .851 | .000 | .000 | .000 | .000 | .000 | .000 | .000 |
| ESE\_8 | -.009 | -.017 | -.095 | .302 | .208 | .905 | .000 | .000 | .000 | .000 | .000 | .000 | .000 |
| ESE\_7 | -.009 | -.017 | -.094 | .301 | .208 | .901 | .000 | .000 | .000 | .000 | .000 | .000 | .000 |
| ESE\_6 | -.009 | -.018 | -.099 | .312 | .214 | .968 | .000 | .000 | .000 | .000 | .000 | .000 | .000 |
| ESE\_5 | -.009 | -.017 | -.099 | .313 | .215 | .973 | .000 | .000 | .000 | .000 | .000 | .000 | .000 |
| ESE\_4 | -.009 | -.018 | -.100 | .318 | .218 | .987 | .000 | .000 | .000 | .000 | .000 | .000 | .000 |
| ESE\_3 | -.008 | -.016 | -.094 | .296 | .205 | .923 | .000 | .000 | .000 | .000 | .000 | .000 | .000 |
| ESE\_2 | -.009 | -.017 | -.099 | .311 | .213 | .969 | .000 | .000 | .000 | .000 | .000 | .000 | .000 |
| ESE\_1 | -.009 | -.017 | -.096 | .302 | .208 | 1.000 | .000 | .000 | .000 | .000 | .000 | .000 | .000 |
| EOE\_9 | -.007 | -.002 | -.079 | .331 | .000 | .000 | .000 | .000 | .000 | .000 | .000 | .000 | .000 |
| EOE\_8 | -.007 | -.002 | -.079 | .339 | .000 | .000 | .000 | .000 | .000 | .000 | .000 | .000 | .000 |
| EOE\_7 | -.007 | -.002 | -.080 | .341 | .000 | .000 | .000 | .000 | .000 | .000 | .000 | .000 | .000 |
| EOE\_6 | -.006 | -.002 | -.077 | .329 | .000 | .000 | .000 | .000 | .000 | .000 | .000 | .000 | .000 |
| EOE\_5 | -.007 | -.002 | -.080 | .343 | .000 | .000 | .000 | .000 | .000 | .000 | .000 | .000 | .000 |
| EOE\_4 | -.007 | -.002 | -.077 | .328 | .000 | .000 | .000 | .000 | .000 | .000 | .000 | .000 | .000 |
| EOE\_3 | -.007 | -.002 | -.078 | .333 | .000 | .000 | .000 | .000 | .000 | .000 | .000 | .000 | .000 |
| EOE\_2 | -.006 | -.002 | -.079 | .336 | .000 | .000 | .000 | .000 | .000 | .000 | .000 | .000 | .000 |
| EOE\_1 | -.006 | -.002 | -.078 | .332 | .000 | .000 | .000 | .000 | .000 | .000 | .000 | .000 | .000 |
| AISE\_6 | .000 | .000 | .000 | .000 | .000 | .000 | .000 | .000 | .000 | .000 | .000 | .000 | .000 |
| AISE\_5 | .000 | .000 | .000 | .000 | .000 | .000 | .000 | .000 | .000 | .000 | .000 | .000 | .000 |
| AISE\_4 | .000 | .000 | .000 | .000 | .000 | .000 | .000 | .000 | .000 | .000 | .000 | .000 | .000 |
| AISE\_3 | .000 | .000 | .000 | .000 | .000 | .000 | .000 | .000 | .000 | .000 | .000 | .000 | .000 |
| AISE\_2 | .000 | .000 | .000 | .000 | .000 | .000 | .000 | .000 | .000 | .000 | .000 | .000 | .000 |
| AISE\_1 | .000 | .000 | .000 | .000 | .000 | .000 | .000 | .000 | .000 | .000 | .000 | .000 | .000 |

## Indirect Effects - Upper Bounds (PC) (Group number 1 - Default model)

|  | Grade | Age | Gender | AISE | EOE | ESE | EE | Pa | Vc | Fs | Vp | Ss | Sm |
| --- | --- | --- | --- | --- | --- | --- | --- | --- | --- | --- | --- | --- | --- |
| EOE | .000 | .000 | .000 | .000 | .000 | .000 | .000 | .000 | .000 | .000 | .000 | .000 | .000 |
| ESE | .010 | .011 | .001 | .113 | .000 | .000 | .000 | .000 | .000 | .000 | .000 | .000 | .000 |
| EE | .014 | .012 | -.006 | .168 | .082 | .000 | .000 | .000 | .000 | .000 | .000 | .000 | .000 |
| Pa | .041 | .040 | -.030 | .309 | .278 | .304 | .000 | .000 | .000 | .000 | .000 | .000 | .000 |
| Vc | .044 | .044 | -.032 | .333 | .300 | .328 | .000 | .000 | .000 | .000 | .000 | .000 | .000 |
| Fs | .042 | .042 | -.032 | .327 | .292 | .320 | .000 | .000 | .000 | .000 | .000 | .000 | .000 |
| Vp | .041 | .041 | -.031 | .315 | .283 | .308 | .000 | .000 | .000 | .000 | .000 | .000 | .000 |
| Ss | .036 | .026 | -.013 | .367 | .305 | .000 | .000 | .000 | .000 | .000 | .000 | .000 | .000 |
| Sm | .036 | .026 | -.013 | .369 | .309 | .000 | .000 | .000 | .000 | .000 | .000 | .000 | .000 |
| EE\_20 | .040 | .040 | -.029 | .305 | .276 | .299 | 1.051 | .000 | .000 | .000 | .000 | .000 | .000 |
| EE\_19 | .039 | .039 | -.029 | .301 | .271 | .295 | 1.033 | .000 | .000 | .000 | .000 | .000 | .000 |
| EE\_18 | .041 | .040 | -.030 | .309 | .278 | .304 | 1.067 | .000 | .000 | .000 | .000 | .000 | .000 |
| EE\_17 | .042 | .042 | -.031 | .323 | .291 | .318 | 1.121 | .000 | .000 | .000 | .000 | .000 | .000 |
| EE\_16 | .043 | .042 | -.032 | .326 | .293 | .322 | 1.128 | .000 | .000 | .000 | .000 | .000 | .000 |
| EE\_15 | .043 | .043 | -.032 | .330 | .297 | .324 | 1.139 | .000 | .000 | .000 | .000 | .000 | .000 |
| EE\_14 | .043 | .042 | -.031 | .326 | .291 | .318 | 1.120 | .000 | .000 | .000 | .000 | .000 | .000 |
| EE\_13 | .044 | .044 | -.032 | .333 | .300 | .328 | 1.156 | .000 | .000 | .000 | .000 | .000 | .000 |
| EE\_12 | .043 | .042 | -.032 | .324 | .291 | .317 | 1.118 | .000 | .000 | .000 | .000 | .000 | .000 |
| EE\_11 | .043 | .042 | -.032 | .327 | .293 | .322 | 1.127 | .000 | .000 | .000 | .000 | .000 | .000 |
| EE\_10 | .043 | .042 | -.032 | .328 | .294 | .323 | 1.134 | .000 | .000 | .000 | .000 | .000 | .000 |
| EE\_9 | .043 | .042 | -.032 | .328 | .294 | .322 | 1.133 | .000 | .000 | .000 | .000 | .000 | .000 |
| EE\_8 | .041 | .041 | -.030 | .317 | .284 | .311 | 1.096 | .000 | .000 | .000 | .000 | .000 | .000 |
| EE\_7 | .042 | .042 | -.032 | .327 | .292 | .320 | 1.124 | .000 | .000 | .000 | .000 | .000 | .000 |
| EE\_6 | .043 | .043 | -.032 | .326 | .293 | .322 | 1.102 | .000 | .000 | .000 | .000 | .000 | .000 |
| EE\_5 | .042 | .042 | -.031 | .323 | .289 | .317 | 1.090 | .000 | .000 | .000 | .000 | .000 | .000 |
| EE\_4 | .043 | .042 | -.031 | .322 | .287 | .316 | 1.088 | .000 | .000 | .000 | .000 | .000 | .000 |
| EE\_3 | .043 | .043 | -.032 | .329 | .295 | .324 | 1.110 | .000 | .000 | .000 | .000 | .000 | .000 |
| EE\_2 | .042 | .042 | -.031 | .322 | .289 | .316 | 1.084 | .000 | .000 | .000 | .000 | .000 | .000 |
| EE\_1 | .041 | .041 | -.031 | .315 | .283 | .308 | 1.000 | .000 | .000 | .000 | .000 | .000 | .000 |
| ESE\_10 | .035 | .026 | -.013 | .362 | .299 | 1.076 | .000 | .000 | .000 | .000 | .000 | .000 | .000 |
| ESE\_9 | .034 | .025 | -.013 | .348 | .290 | 1.040 | .000 | .000 | .000 | .000 | .000 | .000 | .000 |
| ESE\_8 | .036 | .026 | -.013 | .370 | .307 | 1.104 | .000 | .000 | .000 | .000 | .000 | .000 | .000 |
| ESE\_7 | .036 | .026 | -.013 | .367 | .305 | 1.095 | .000 | .000 | .000 | .000 | .000 | .000 | .000 |
| ESE\_6 | .037 | .027 | -.014 | .380 | .317 | 1.092 | .000 | .000 | .000 | .000 | .000 | .000 | .000 |
| ESE\_5 | .037 | .027 | -.014 | .382 | .319 | 1.097 | .000 | .000 | .000 | .000 | .000 | .000 | .000 |
| ESE\_4 | .038 | .028 | -.014 | .385 | .324 | 1.111 | .000 | .000 | .000 | .000 | .000 | .000 | .000 |
| ESE\_3 | .035 | .026 | -.013 | .363 | .303 | 1.046 | .000 | .000 | .000 | .000 | .000 | .000 | .000 |
| ESE\_2 | .037 | .027 | -.014 | .379 | .318 | 1.091 | .000 | .000 | .000 | .000 | .000 | .000 | .000 |
| ESE\_1 | .036 | .026 | -.013 | .369 | .309 | 1.000 | .000 | .000 | .000 | .000 | .000 | .000 | .000 |
| EOE\_9 | .039 | .042 | .004 | .398 | .000 | .000 | .000 | .000 | .000 | .000 | .000 | .000 | .000 |
| EOE\_8 | .040 | .044 | .004 | .407 | .000 | .000 | .000 | .000 | .000 | .000 | .000 | .000 | .000 |
| EOE\_7 | .040 | .044 | .004 | .409 | .000 | .000 | .000 | .000 | .000 | .000 | .000 | .000 | .000 |
| EOE\_6 | .038 | .042 | .004 | .393 | .000 | .000 | .000 | .000 | .000 | .000 | .000 | .000 | .000 |
| EOE\_5 | .040 | .044 | .004 | .409 | .000 | .000 | .000 | .000 | .000 | .000 | .000 | .000 | .000 |
| EOE\_4 | .038 | .042 | .004 | .395 | .000 | .000 | .000 | .000 | .000 | .000 | .000 | .000 | .000 |
| EOE\_3 | .039 | .043 | .004 | .398 | .000 | .000 | .000 | .000 | .000 | .000 | .000 | .000 | .000 |
| EOE\_2 | .039 | .043 | .004 | .403 | .000 | .000 | .000 | .000 | .000 | .000 | .000 | .000 | .000 |
| EOE\_1 | .039 | .042 | .004 | .396 | .000 | .000 | .000 | .000 | .000 | .000 | .000 | .000 | .000 |
| AISE\_6 | .000 | .000 | .000 | .000 | .000 | .000 | .000 | .000 | .000 | .000 | .000 | .000 | .000 |
| AISE\_5 | .000 | .000 | .000 | .000 | .000 | .000 | .000 | .000 | .000 | .000 | .000 | .000 | .000 |
| AISE\_4 | .000 | .000 | .000 | .000 | .000 | .000 | .000 | .000 | .000 | .000 | .000 | .000 | .000 |
| AISE\_3 | .000 | .000 | .000 | .000 | .000 | .000 | .000 | .000 | .000 | .000 | .000 | .000 | .000 |
| AISE\_2 | .000 | .000 | .000 | .000 | .000 | .000 | .000 | .000 | .000 | .000 | .000 | .000 | .000 |
| AISE\_1 | .000 | .000 | .000 | .000 | .000 | .000 | .000 | .000 | .000 | .000 | .000 | .000 | .000 |

## Indirect Effects - Two Tailed Significance (PC) (Group number 1 - Default model)

|  | Grade | Age | Gender | AISE | EOE | ESE | EE | Pa | Vc | Fs | Vp | Ss | Sm |
| --- | --- | --- | --- | --- | --- | --- | --- | --- | --- | --- | --- | --- | --- |
| EOE | ... | ... | ... | ... | ... | ... | ... | ... | ... | ... | ... | ... | ... |
| ESE | .148 | .084 | .075 | .000 | ... | ... | ... | ... | ... | ... | ... | ... | ... |
| EE | .098 | .206 | .004 | .000 | .000 | ... | ... | ... | ... | ... | ... | ... | ... |
| Pa | .062 | .041 | .001 | .000 | .000 | .000 | ... | ... | ... | ... | ... | ... | ... |
| Vc | .062 | .041 | .001 | .000 | .000 | .000 | ... | ... | ... | ... | ... | ... | ... |
| Fs | .062 | .041 | .001 | .000 | .000 | .000 | ... | ... | ... | ... | ... | ... | ... |
| Vp | .062 | .041 | .001 | .000 | .000 | .000 | ... | ... | ... | ... | ... | ... | ... |
| Ss | .243 | .675 | .010 | .000 | .000 | ... | ... | ... | ... | ... | ... | ... | ... |
| Sm | .243 | .675 | .010 | .000 | .000 | ... | ... | ... | ... | ... | ... | ... | ... |
| EE\_20 | .062 | .041 | .001 | .000 | .000 | .000 | .000 | ... | ... | ... | ... | ... | ... |
| EE\_19 | .062 | .041 | .001 | .000 | .000 | .000 | .000 | ... | ... | ... | ... | ... | ... |
| EE\_18 | .062 | .041 | .001 | .000 | .000 | .000 | .000 | ... | ... | ... | ... | ... | ... |
| EE\_17 | .062 | .041 | .001 | .000 | .000 | .000 | .000 | ... | ... | ... | ... | ... | ... |
| EE\_16 | .062 | .041 | .001 | .000 | .000 | .000 | .000 | ... | ... | ... | ... | ... | ... |
| EE\_15 | .062 | .041 | .001 | .000 | .000 | .000 | .000 | ... | ... | ... | ... | ... | ... |
| EE\_14 | .062 | .041 | .001 | .000 | .000 | .000 | .000 | ... | ... | ... | ... | ... | ... |
| EE\_13 | .062 | .041 | .001 | .000 | .000 | .000 | .000 | ... | ... | ... | ... | ... | ... |
| EE\_12 | .062 | .041 | .001 | .000 | .000 | .000 | .000 | ... | ... | ... | ... | ... | ... |
| EE\_11 | .062 | .041 | .001 | .000 | .000 | .000 | .000 | ... | ... | ... | ... | ... | ... |
| EE\_10 | .062 | .041 | .001 | .000 | .000 | .000 | .000 | ... | ... | ... | ... | ... | ... |
| EE\_9 | .062 | .041 | .001 | .000 | .000 | .000 | .000 | ... | ... | ... | ... | ... | ... |
| EE\_8 | .062 | .041 | .001 | .000 | .000 | .000 | .000 | ... | ... | ... | ... | ... | ... |
| EE\_7 | .062 | .041 | .001 | .000 | .000 | .000 | .000 | ... | ... | ... | ... | ... | ... |
| EE\_6 | .062 | .041 | .001 | .000 | .000 | .000 | .000 | ... | ... | ... | ... | ... | ... |
| EE\_5 | .062 | .041 | .001 | .000 | .000 | .000 | .000 | ... | ... | ... | ... | ... | ... |
| EE\_4 | .062 | .041 | .001 | .000 | .000 | .000 | .000 | ... | ... | ... | ... | ... | ... |
| EE\_3 | .062 | .041 | .001 | .000 | .000 | .000 | .000 | ... | ... | ... | ... | ... | ... |
| EE\_2 | .062 | .041 | .001 | .000 | .000 | .000 | .000 | ... | ... | ... | ... | ... | ... |
| EE\_1 | .062 | .041 | .001 | .000 | .000 | .000 | ... | ... | ... | ... | ... | ... | ... |
| ESE\_10 | .243 | .675 | .010 | .000 | .000 | .000 | ... | ... | ... | ... | ... | ... | ... |
| ESE\_9 | .243 | .675 | .010 | .000 | .000 | .000 | ... | ... | ... | ... | ... | ... | ... |
| ESE\_8 | .243 | .675 | .010 | .000 | .000 | .000 | ... | ... | ... | ... | ... | ... | ... |
| ESE\_7 | .243 | .675 | .010 | .000 | .000 | .000 | ... | ... | ... | ... | ... | ... | ... |
| ESE\_6 | .243 | .675 | .010 | .000 | .000 | .000 | ... | ... | ... | ... | ... | ... | ... |
| ESE\_5 | .243 | .675 | .010 | .000 | .000 | .000 | ... | ... | ... | ... | ... | ... | ... |
| ESE\_4 | .243 | .675 | .010 | .000 | .000 | .000 | ... | ... | ... | ... | ... | ... | ... |
| ESE\_3 | .243 | .675 | .010 | .000 | .000 | .000 | ... | ... | ... | ... | ... | ... | ... |
| ESE\_2 | .243 | .675 | .010 | .000 | .000 | .000 | ... | ... | ... | ... | ... | ... | ... |
| ESE\_1 | .243 | .675 | .010 | .000 | .000 | ... | ... | ... | ... | ... | ... | ... | ... |
| EOE\_9 | .148 | .084 | .075 | .000 | ... | ... | ... | ... | ... | ... | ... | ... | ... |
| EOE\_8 | .148 | .084 | .075 | .000 | ... | ... | ... | ... | ... | ... | ... | ... | ... |
| EOE\_7 | .148 | .084 | .075 | .000 | ... | ... | ... | ... | ... | ... | ... | ... | ... |
| EOE\_6 | .148 | .084 | .075 | .000 | ... | ... | ... | ... | ... | ... | ... | ... | ... |
| EOE\_5 | .148 | .084 | .075 | .000 | ... | ... | ... | ... | ... | ... | ... | ... | ... |
| EOE\_4 | .148 | .084 | .075 | .000 | ... | ... | ... | ... | ... | ... | ... | ... | ... |
| EOE\_3 | .148 | .084 | .075 | .000 | ... | ... | ... | ... | ... | ... | ... | ... | ... |
| EOE\_2 | .148 | .084 | .075 | .000 | ... | ... | ... | ... | ... | ... | ... | ... | ... |
| EOE\_1 | .148 | .084 | .075 | .000 | ... | ... | ... | ... | ... | ... | ... | ... | ... |
| AISE\_6 | ... | ... | ... | ... | ... | ... | ... | ... | ... | ... | ... | ... | ... |
| AISE\_5 | ... | ... | ... | ... | ... | ... | ... | ... | ... | ... | ... | ... | ... |
| AISE\_4 | ... | ... | ... | ... | ... | ... | ... | ... | ... | ... | ... | ... | ... |
| AISE\_3 | ... | ... | ... | ... | ... | ... | ... | ... | ... | ... | ... | ... | ... |
| AISE\_2 | ... | ... | ... | ... | ... | ... | ... | ... | ... | ... | ... | ... | ... |
| AISE\_1 | ... | ... | ... | ... | ... | ... | ... | ... | ... | ... | ... | ... | ... |

## Standardized Indirect Effects (Group number 1 - Default model)

## Standardized Indirect Effects - Lower Bounds (PC) (Group number 1 - Default model)

|  | Grade | Age | Gender | AISE | EOE | ESE | EE | Pa | Vc | Fs | Vp | Ss | Sm |
| --- | --- | --- | --- | --- | --- | --- | --- | --- | --- | --- | --- | --- | --- |
| EOE | .000 | .000 | .000 | .000 | .000 | .000 | .000 | .000 | .000 | .000 | .000 | .000 | .000 |
| ESE | -.003 | -.001 | -.023 | .135 | .000 | .000 | .000 | .000 | .000 | .000 | .000 | .000 | .000 |
| EE | -.002 | -.005 | -.037 | .208 | .048 | .000 | .000 | .000 | .000 | .000 | .000 | .000 | .000 |
| Pa | -.001 | .001 | -.092 | .366 | .180 | .131 | .000 | .000 | .000 | .000 | .000 | .000 | .000 |
| Vc | -.001 | .001 | -.094 | .378 | .185 | .133 | .000 | .000 | .000 | .000 | .000 | .000 | .000 |
| Fs | -.001 | .001 | -.093 | .372 | .183 | .132 | .000 | .000 | .000 | .000 | .000 | .000 | .000 |
| Vp | -.001 | .001 | -.092 | .369 | .181 | .131 | .000 | .000 | .000 | .000 | .000 | .000 | .000 |
| Ss | -.013 | -.027 | -.081 | .428 | .193 | .000 | .000 | .000 | .000 | .000 | .000 | .000 | .000 |
| Sm | -.013 | -.028 | -.085 | .445 | .199 | .000 | .000 | .000 | .000 | .000 | .000 | .000 | .000 |
| EE\_20 | -.001 | .001 | -.067 | .263 | .130 | .094 | .552 | .000 | .000 | .000 | .000 | .000 | .000 |
| EE\_19 | -.001 | .001 | -.066 | .259 | .128 | .093 | .545 | .000 | .000 | .000 | .000 | .000 | .000 |
| EE\_18 | -.001 | .001 | -.068 | .268 | .132 | .096 | .564 | .000 | .000 | .000 | .000 | .000 | .000 |
| EE\_17 | -.001 | .001 | -.070 | .276 | .136 | .099 | .580 | .000 | .000 | .000 | .000 | .000 | .000 |
| EE\_16 | -.001 | .001 | -.069 | .276 | .135 | .098 | .578 | .000 | .000 | .000 | .000 | .000 | .000 |
| EE\_15 | -.001 | .001 | -.071 | .280 | .138 | .100 | .590 | .000 | .000 | .000 | .000 | .000 | .000 |
| EE\_14 | -.001 | .001 | -.069 | .275 | .135 | .098 | .578 | .000 | .000 | .000 | .000 | .000 | .000 |
| EE\_13 | -.001 | .001 | -.071 | .281 | .139 | .100 | .591 | .000 | .000 | .000 | .000 | .000 | .000 |
| EE\_12 | -.001 | .001 | -.068 | .271 | .134 | .097 | .570 | .000 | .000 | .000 | .000 | .000 | .000 |
| EE\_11 | -.001 | .001 | -.068 | .271 | .134 | .097 | .571 | .000 | .000 | .000 | .000 | .000 | .000 |
| EE\_10 | -.001 | .001 | -.069 | .272 | .134 | .097 | .573 | .000 | .000 | .000 | .000 | .000 | .000 |
| EE\_9 | -.001 | .001 | -.069 | .273 | .134 | .097 | .574 | .000 | .000 | .000 | .000 | .000 | .000 |
| EE\_8 | -.001 | .001 | -.067 | .263 | .130 | .095 | .554 | .000 | .000 | .000 | .000 | .000 | .000 |
| EE\_7 | -.001 | .001 | -.067 | .269 | .133 | .096 | .567 | .000 | .000 | .000 | .000 | .000 | .000 |
| EE\_6 | -.001 | .001 | -.068 | .271 | .133 | .097 | .570 | .000 | .000 | .000 | .000 | .000 | .000 |
| EE\_5 | -.001 | .001 | -.067 | .266 | .131 | .095 | .558 | .000 | .000 | .000 | .000 | .000 | .000 |
| EE\_4 | -.001 | .001 | -.068 | .268 | .133 | .096 | .565 | .000 | .000 | .000 | .000 | .000 | .000 |
| EE\_3 | -.001 | .001 | -.069 | .273 | .134 | .098 | .572 | .000 | .000 | .000 | .000 | .000 | .000 |
| EE\_2 | -.001 | .001 | -.067 | .264 | .130 | .095 | .555 | .000 | .000 | .000 | .000 | .000 | .000 |
| EE\_1 | -.001 | .001 | -.066 | .263 | .130 | .094 | .551 | .000 | .000 | .000 | .000 | .000 | .000 |
| ESE\_10 | -.010 | -.020 | -.060 | .310 | .142 | .542 | .000 | .000 | .000 | .000 | .000 | .000 | .000 |
| ESE\_9 | -.009 | -.019 | -.058 | .305 | .139 | .530 | .000 | .000 | .000 | .000 | .000 | .000 | .000 |
| ESE\_8 | -.010 | -.020 | -.061 | .318 | .144 | .553 | .000 | .000 | .000 | .000 | .000 | .000 | .000 |
| ESE\_7 | -.010 | -.020 | -.060 | .317 | .144 | .552 | .000 | .000 | .000 | .000 | .000 | .000 | .000 |
| ESE\_6 | -.010 | -.020 | -.062 | .318 | .143 | .552 | .000 | .000 | .000 | .000 | .000 | .000 | .000 |
| ESE\_5 | -.010 | -.020 | -.063 | .326 | .147 | .567 | .000 | .000 | .000 | .000 | .000 | .000 | .000 |
| ESE\_4 | -.010 | -.020 | -.063 | .327 | .147 | .568 | .000 | .000 | .000 | .000 | .000 | .000 | .000 |
| ESE\_3 | -.010 | -.020 | -.061 | .314 | .142 | .545 | .000 | .000 | .000 | .000 | .000 | .000 | .000 |
| ESE\_2 | -.010 | -.020 | -.063 | .323 | .146 | .562 | .000 | .000 | .000 | .000 | .000 | .000 | .000 |
| ESE\_1 | -.009 | -.020 | -.061 | .313 | .142 | .545 | .000 | .000 | .000 | .000 | .000 | .000 | .000 |
| EOE\_9 | -.008 | -.002 | -.053 | .364 | .000 | .000 | .000 | .000 | .000 | .000 | .000 | .000 | .000 |
| EOE\_8 | -.008 | -.003 | -.053 | .369 | .000 | .000 | .000 | .000 | .000 | .000 | .000 | .000 | .000 |
| EOE\_7 | -.008 | -.003 | -.055 | .379 | .000 | .000 | .000 | .000 | .000 | .000 | .000 | .000 | .000 |
| EOE\_6 | -.008 | -.002 | -.052 | .364 | .000 | .000 | .000 | .000 | .000 | .000 | .000 | .000 | .000 |
| EOE\_5 | -.008 | -.003 | -.054 | .375 | .000 | .000 | .000 | .000 | .000 | .000 | .000 | .000 | .000 |
| EOE\_4 | -.008 | -.003 | -.053 | .367 | .000 | .000 | .000 | .000 | .000 | .000 | .000 | .000 | .000 |
| EOE\_3 | -.008 | -.003 | -.053 | .366 | .000 | .000 | .000 | .000 | .000 | .000 | .000 | .000 | .000 |
| EOE\_2 | -.008 | -.003 | -.053 | .370 | .000 | .000 | .000 | .000 | .000 | .000 | .000 | .000 | .000 |
| EOE\_1 | -.008 | -.003 | -.053 | .365 | .000 | .000 | .000 | .000 | .000 | .000 | .000 | .000 | .000 |
| AISE\_6 | .000 | .000 | .000 | .000 | .000 | .000 | .000 | .000 | .000 | .000 | .000 | .000 | .000 |
| AISE\_5 | .000 | .000 | .000 | .000 | .000 | .000 | .000 | .000 | .000 | .000 | .000 | .000 | .000 |
| AISE\_4 | .000 | .000 | .000 | .000 | .000 | .000 | .000 | .000 | .000 | .000 | .000 | .000 | .000 |
| AISE\_3 | .000 | .000 | .000 | .000 | .000 | .000 | .000 | .000 | .000 | .000 | .000 | .000 | .000 |
| AISE\_2 | .000 | .000 | .000 | .000 | .000 | .000 | .000 | .000 | .000 | .000 | .000 | .000 | .000 |
| AISE\_1 | .000 | .000 | .000 | .000 | .000 | .000 | .000 | .000 | .000 | .000 | .000 | .000 | .000 |

## Standardized Indirect Effects - Upper Bounds (PC) (Group number 1 - Default model)

|  | Grade | Age | Gender | AISE | EOE | ESE | EE | Pa | Vc | Fs | Vp | Ss | Sm |
| --- | --- | --- | --- | --- | --- | --- | --- | --- | --- | --- | --- | --- | --- |
| EOE | .000 | .000 | .000 | .000 | .000 | .000 | .000 | .000 | .000 | .000 | .000 | .000 | .000 |
| ESE | .019 | .022 | .001 | .196 | .000 | .000 | .000 | .000 | .000 | .000 | .000 | .000 | .000 |
| EE | .026 | .024 | -.006 | .292 | .095 | .000 | .000 | .000 | .000 | .000 | .000 | .000 | .000 |
| Pa | .064 | .066 | -.027 | .435 | .264 | .242 | .000 | .000 | .000 | .000 | .000 | .000 | .000 |
| Vc | .065 | .067 | -.028 | .444 | .269 | .247 | .000 | .000 | .000 | .000 | .000 | .000 | .000 |
| Fs | .064 | .067 | -.027 | .441 | .266 | .244 | .000 | .000 | .000 | .000 | .000 | .000 | .000 |
| Vp | .064 | .066 | -.027 | .436 | .263 | .243 | .000 | .000 | .000 | .000 | .000 | .000 | .000 |
| Ss | .054 | .042 | -.011 | .502 | .280 | .000 | .000 | .000 | .000 | .000 | .000 | .000 | .000 |
| Sm | .056 | .043 | -.012 | .516 | .291 | .000 | .000 | .000 | .000 | .000 | .000 | .000 | .000 |
| EE\_20 | .046 | .048 | -.019 | .317 | .192 | .175 | .610 | .000 | .000 | .000 | .000 | .000 | .000 |
| EE\_19 | .045 | .047 | -.019 | .314 | .190 | .174 | .604 | .000 | .000 | .000 | .000 | .000 | .000 |
| EE\_18 | .047 | .049 | -.020 | .324 | .195 | .179 | .621 | .000 | .000 | .000 | .000 | .000 | .000 |
| EE\_17 | .048 | .050 | -.020 | .331 | .199 | .183 | .634 | .000 | .000 | .000 | .000 | .000 | .000 |
| EE\_16 | .048 | .050 | -.020 | .329 | .199 | .183 | .631 | .000 | .000 | .000 | .000 | .000 | .000 |
| EE\_15 | .049 | .051 | -.021 | .335 | .202 | .185 | .643 | .000 | .000 | .000 | .000 | .000 | .000 |
| EE\_14 | .048 | .050 | -.020 | .330 | .198 | .181 | .630 | .000 | .000 | .000 | .000 | .000 | .000 |
| EE\_13 | .049 | .051 | -.021 | .336 | .202 | .186 | .644 | .000 | .000 | .000 | .000 | .000 | .000 |
| EE\_12 | .047 | .049 | -.020 | .327 | .196 | .180 | .624 | .000 | .000 | .000 | .000 | .000 | .000 |
| EE\_11 | .047 | .049 | -.020 | .326 | .196 | .181 | .625 | .000 | .000 | .000 | .000 | .000 | .000 |
| EE\_10 | .047 | .049 | -.020 | .328 | .196 | .181 | .625 | .000 | .000 | .000 | .000 | .000 | .000 |
| EE\_9 | .048 | .049 | -.020 | .329 | .197 | .182 | .628 | .000 | .000 | .000 | .000 | .000 | .000 |
| EE\_8 | .046 | .048 | -.019 | .318 | .191 | .175 | .608 | .000 | .000 | .000 | .000 | .000 | .000 |
| EE\_7 | .047 | .049 | -.020 | .323 | .194 | .179 | .618 | .000 | .000 | .000 | .000 | .000 | .000 |
| EE\_6 | .048 | .049 | -.020 | .325 | .196 | .181 | .625 | .000 | .000 | .000 | .000 | .000 | .000 |
| EE\_5 | .047 | .048 | -.020 | .319 | .192 | .176 | .612 | .000 | .000 | .000 | .000 | .000 | .000 |
| EE\_4 | .047 | .048 | -.020 | .323 | .193 | .178 | .616 | .000 | .000 | .000 | .000 | .000 | .000 |
| EE\_3 | .048 | .050 | -.020 | .327 | .196 | .181 | .627 | .000 | .000 | .000 | .000 | .000 | .000 |
| EE\_2 | .046 | .048 | -.020 | .318 | .191 | .175 | .609 | .000 | .000 | .000 | .000 | .000 | .000 |
| EE\_1 | .046 | .048 | -.020 | .317 | .191 | .175 | .608 | .000 | .000 | .000 | .000 | .000 | .000 |
| ESE\_10 | .040 | .031 | -.008 | .373 | .206 | .609 | .000 | .000 | .000 | .000 | .000 | .000 | .000 |
| ESE\_9 | .039 | .030 | -.008 | .362 | .201 | .593 | .000 | .000 | .000 | .000 | .000 | .000 | .000 |
| ESE\_8 | .041 | .031 | -.009 | .379 | .210 | .618 | .000 | .000 | .000 | .000 | .000 | .000 | .000 |
| ESE\_7 | .041 | .031 | -.009 | .376 | .209 | .615 | .000 | .000 | .000 | .000 | .000 | .000 | .000 |
| ESE\_6 | .041 | .031 | -.008 | .376 | .210 | .617 | .000 | .000 | .000 | .000 | .000 | .000 | .000 |
| ESE\_5 | .042 | .032 | -.009 | .385 | .216 | .632 | .000 | .000 | .000 | .000 | .000 | .000 | .000 |
| ESE\_4 | .042 | .032 | -.009 | .385 | .216 | .631 | .000 | .000 | .000 | .000 | .000 | .000 | .000 |
| ESE\_3 | .040 | .031 | -.008 | .372 | .208 | .609 | .000 | .000 | .000 | .000 | .000 | .000 | .000 |
| ESE\_2 | .041 | .032 | -.009 | .383 | .214 | .627 | .000 | .000 | .000 | .000 | .000 | .000 | .000 |
| ESE\_1 | .040 | .031 | -.008 | .371 | .208 | .609 | .000 | .000 | .000 | .000 | .000 | .000 | .000 |
| EOE\_9 | .046 | .052 | .002 | .422 | .000 | .000 | .000 | .000 | .000 | .000 | .000 | .000 | .000 |
| EOE\_8 | .046 | .053 | .002 | .427 | .000 | .000 | .000 | .000 | .000 | .000 | .000 | .000 | .000 |
| EOE\_7 | .047 | .054 | .003 | .437 | .000 | .000 | .000 | .000 | .000 | .000 | .000 | .000 | .000 |
| EOE\_6 | .045 | .052 | .002 | .420 | .000 | .000 | .000 | .000 | .000 | .000 | .000 | .000 | .000 |
| EOE\_5 | .047 | .054 | .002 | .433 | .000 | .000 | .000 | .000 | .000 | .000 | .000 | .000 | .000 |
| EOE\_4 | .046 | .053 | .002 | .425 | .000 | .000 | .000 | .000 | .000 | .000 | .000 | .000 | .000 |
| EOE\_3 | .046 | .053 | .002 | .423 | .000 | .000 | .000 | .000 | .000 | .000 | .000 | .000 | .000 |
| EOE\_2 | .046 | .053 | .002 | .427 | .000 | .000 | .000 | .000 | .000 | .000 | .000 | .000 | .000 |
| EOE\_1 | .046 | .052 | .002 | .422 | .000 | .000 | .000 | .000 | .000 | .000 | .000 | .000 | .000 |
| AISE\_6 | .000 | .000 | .000 | .000 | .000 | .000 | .000 | .000 | .000 | .000 | .000 | .000 | .000 |
| AISE\_5 | .000 | .000 | .000 | .000 | .000 | .000 | .000 | .000 | .000 | .000 | .000 | .000 | .000 |
| AISE\_4 | .000 | .000 | .000 | .000 | .000 | .000 | .000 | .000 | .000 | .000 | .000 | .000 | .000 |
| AISE\_3 | .000 | .000 | .000 | .000 | .000 | .000 | .000 | .000 | .000 | .000 | .000 | .000 | .000 |
| AISE\_2 | .000 | .000 | .000 | .000 | .000 | .000 | .000 | .000 | .000 | .000 | .000 | .000 | .000 |
| AISE\_1 | .000 | .000 | .000 | .000 | .000 | .000 | .000 | .000 | .000 | .000 | .000 | .000 | .000 |

## Standardized Indirect Effects - Two Tailed Significance (PC) (Group number 1 - Default model)

|  | Grade | Age | Gender | AISE | EOE | ESE | EE | Pa | Vc | Fs | Vp | Ss | Sm |
| --- | --- | --- | --- | --- | --- | --- | --- | --- | --- | --- | --- | --- | --- |
| EOE | ... | ... | ... | ... | ... | ... | ... | ... | ... | ... | ... | ... | ... |
| ESE | .148 | .084 | .075 | .000 | ... | ... | ... | ... | ... | ... | ... | ... | ... |
| EE | .098 | .206 | .004 | .000 | .000 | ... | ... | ... | ... | ... | ... | ... | ... |
| Pa | .062 | .041 | .001 | .000 | .000 | .000 | ... | ... | ... | ... | ... | ... | ... |
| Vc | .062 | .041 | .001 | .000 | .000 | .000 | ... | ... | ... | ... | ... | ... | ... |
| Fs | .062 | .041 | .001 | .000 | .000 | .000 | ... | ... | ... | ... | ... | ... | ... |
| Vp | .062 | .041 | .001 | .000 | .000 | .000 | ... | ... | ... | ... | ... | ... | ... |
| Ss | .243 | .675 | .010 | .000 | .000 | ... | ... | ... | ... | ... | ... | ... | ... |
| Sm | .243 | .675 | .010 | .000 | .000 | ... | ... | ... | ... | ... | ... | ... | ... |
| EE\_20 | .062 | .041 | .001 | .000 | .000 | .000 | .000 | ... | ... | ... | ... | ... | ... |
| EE\_19 | .062 | .041 | .001 | .000 | .000 | .000 | .000 | ... | ... | ... | ... | ... | ... |
| EE\_18 | .062 | .041 | .001 | .000 | .000 | .000 | .000 | ... | ... | ... | ... | ... | ... |
| EE\_17 | .062 | .041 | .001 | .000 | .000 | .000 | .000 | ... | ... | ... | ... | ... | ... |
| EE\_16 | .062 | .041 | .001 | .000 | .000 | .000 | .000 | ... | ... | ... | ... | ... | ... |
| EE\_15 | .062 | .041 | .001 | .000 | .000 | .000 | .000 | ... | ... | ... | ... | ... | ... |
| EE\_14 | .062 | .041 | .001 | .000 | .000 | .000 | .000 | ... | ... | ... | ... | ... | ... |
| EE\_13 | .062 | .041 | .001 | .000 | .000 | .000 | .000 | ... | ... | ... | ... | ... | ... |
| EE\_12 | .062 | .041 | .001 | .000 | .000 | .000 | .000 | ... | ... | ... | ... | ... | ... |
| EE\_11 | .062 | .041 | .001 | .000 | .000 | .000 | .000 | ... | ... | ... | ... | ... | ... |
| EE\_10 | .062 | .041 | .001 | .000 | .000 | .000 | .000 | ... | ... | ... | ... | ... | ... |
| EE\_9 | .062 | .041 | .001 | .000 | .000 | .000 | .000 | ... | ... | ... | ... | ... | ... |
| EE\_8 | .062 | .041 | .001 | .000 | .000 | .000 | .000 | ... | ... | ... | ... | ... | ... |
| EE\_7 | .062 | .041 | .001 | .000 | .000 | .000 | .000 | ... | ... | ... | ... | ... | ... |
| EE\_6 | .062 | .041 | .001 | .000 | .000 | .000 | .000 | ... | ... | ... | ... | ... | ... |
| EE\_5 | .062 | .041 | .001 | .000 | .000 | .000 | .000 | ... | ... | ... | ... | ... | ... |
| EE\_4 | .062 | .041 | .001 | .000 | .000 | .000 | .000 | ... | ... | ... | ... | ... | ... |
| EE\_3 | .062 | .041 | .001 | .000 | .000 | .000 | .000 | ... | ... | ... | ... | ... | ... |
| EE\_2 | .062 | .041 | .001 | .000 | .000 | .000 | .000 | ... | ... | ... | ... | ... | ... |
| EE\_1 | .062 | .041 | .001 | .000 | .000 | .000 | .000 | ... | ... | ... | ... | ... | ... |
| ESE\_10 | .243 | .675 | .010 | .000 | .000 | .000 | ... | ... | ... | ... | ... | ... | ... |
| ESE\_9 | .243 | .675 | .010 | .000 | .000 | .000 | ... | ... | ... | ... | ... | ... | ... |
| ESE\_8 | .243 | .675 | .010 | .000 | .000 | .000 | ... | ... | ... | ... | ... | ... | ... |
| ESE\_7 | .243 | .675 | .010 | .000 | .000 | .000 | ... | ... | ... | ... | ... | ... | ... |
| ESE\_6 | .243 | .675 | .010 | .000 | .000 | .000 | ... | ... | ... | ... | ... | ... | ... |
| ESE\_5 | .243 | .675 | .010 | .000 | .000 | .000 | ... | ... | ... | ... | ... | ... | ... |
| ESE\_4 | .243 | .675 | .010 | .000 | .000 | .000 | ... | ... | ... | ... | ... | ... | ... |
| ESE\_3 | .243 | .675 | .010 | .000 | .000 | .000 | ... | ... | ... | ... | ... | ... | ... |
| ESE\_2 | .243 | .675 | .010 | .000 | .000 | .000 | ... | ... | ... | ... | ... | ... | ... |
| ESE\_1 | .243 | .675 | .010 | .000 | .000 | .000 | ... | ... | ... | ... | ... | ... | ... |
| EOE\_9 | .148 | .084 | .075 | .000 | ... | ... | ... | ... | ... | ... | ... | ... | ... |
| EOE\_8 | .148 | .084 | .075 | .000 | ... | ... | ... | ... | ... | ... | ... | ... | ... |
| EOE\_7 | .148 | .084 | .075 | .000 | ... | ... | ... | ... | ... | ... | ... | ... | ... |
| EOE\_6 | .148 | .084 | .075 | .000 | ... | ... | ... | ... | ... | ... | ... | ... | ... |
| EOE\_5 | .148 | .084 | .075 | .000 | ... | ... | ... | ... | ... | ... | ... | ... | ... |
| EOE\_4 | .148 | .084 | .075 | .000 | ... | ... | ... | ... | ... | ... | ... | ... | ... |
| EOE\_3 | .148 | .084 | .075 | .000 | ... | ... | ... | ... | ... | ... | ... | ... | ... |
| EOE\_2 | .148 | .084 | .075 | .000 | ... | ... | ... | ... | ... | ... | ... | ... | ... |
| EOE\_1 | .148 | .084 | .075 | .000 | ... | ... | ... | ... | ... | ... | ... | ... | ... |
| AISE\_6 | ... | ... | ... | ... | ... | ... | ... | ... | ... | ... | ... | ... | ... |
| AISE\_5 | ... | ... | ... | ... | ... | ... | ... | ... | ... | ... | ... | ... | ... |
| AISE\_4 | ... | ... | ... | ... | ... | ... | ... | ... | ... | ... | ... | ... | ... |
| AISE\_3 | ... | ... | ... | ... | ... | ... | ... | ... | ... | ... | ... | ... | ... |
| AISE\_2 | ... | ... | ... | ... | ... | ... | ... | ... | ... | ... | ... | ... | ... |
| AISE\_1 | ... | ... | ... | ... | ... | ... | ... | ... | ... | ... | ... | ... | ... |

## Bias-corrected percentile method (Group number 1 - Default model)

## 95% confidence intervals (bias-corrected percentile method)

## Scalar Estimates (Group number 1 - Default model)

## Regression Weights: (Group number 1 - Default model)

| Parameter | | | Estimate | Lower | Upper | P |
| --- | --- | --- | --- | --- | --- | --- |
| EOE | <--- | AISE | .363 | .331 | .395 | .001 |
| EOE | <--- | Gender | -.037 | -.078 | .003 | .071 |
| EOE | <--- | Age | .020 | -.002 | .043 | .082 |
| EOE | <--- | Grade | .016 | -.006 | .039 | .139 |
| ESE | <--- | AISE | .242 | .208 | .277 | .000 |
| ESE | <--- | EOE | .257 | .207 | .308 | .000 |
| ESE | <--- | Gender | -.045 | -.085 | -.006 | .026 |
| ESE | <--- | Age | .000 | -.021 | .020 | .983 |
| ESE | <--- | Grade | .009 | -.013 | .031 | .393 |
| EE | <--- | ESE | .232 | .160 | .308 | .000 |
| EE | <--- | EOE | .176 | .131 | .226 | .000 |
| EE | <--- | AISE | .142 | .107 | .179 | .000 |
| EE | <--- | Gender | -.049 | -.084 | -.012 | .007 |
| EE | <--- | Age | .016 | -.003 | .035 | .102 |
| EE | <--- | Grade | .014 | -.007 | .034 | .172 |
| Sm | <--- | ESE | 1.000 | 1.000 | 1.000 | ... |
| Ss | <--- | ESE | .993 | .902 | 1.096 | .000 |
| Vp | <--- | EE | 1.000 | 1.000 | 1.000 | ... |
| Fs | <--- | EE | 1.034 | .953 | 1.121 | .000 |
| Vc | <--- | EE | 1.062 | .979 | 1.154 | .000 |
| Pa | <--- | EE | .978 | .895 | 1.063 | .001 |
| AISE\_1 | <--- | AISE | 1.000 | 1.000 | 1.000 | ... |
| AISE\_2 | <--- | AISE | .983 | .932 | 1.036 | .000 |
| AISE\_3 | <--- | AISE | .959 | .909 | 1.012 | .000 |
| AISE\_4 | <--- | AISE | .991 | .939 | 1.045 | .000 |
| AISE\_5 | <--- | AISE | .955 | .905 | 1.009 | .000 |
| AISE\_6 | <--- | AISE | .999 | .948 | 1.052 | .000 |
| EOE\_1 | <--- | EOE | 1.000 | 1.000 | 1.000 | ... |
| EOE\_2 | <--- | EOE | 1.014 | .961 | 1.071 | .000 |
| EOE\_3 | <--- | EOE | 1.002 | .947 | 1.058 | .000 |
| EOE\_4 | <--- | EOE | .993 | .938 | 1.051 | .000 |
| EOE\_5 | <--- | EOE | 1.032 | .974 | 1.092 | .000 |
| EOE\_6 | <--- | EOE | .992 | .938 | 1.052 | .000 |
| EOE\_7 | <--- | EOE | 1.030 | .973 | 1.091 | .000 |
| EOE\_8 | <--- | EOE | 1.024 | .969 | 1.086 | .000 |
| EOE\_9 | <--- | EOE | 1.001 | .943 | 1.061 | .000 |
| ESE\_1 | <--- | Sm | 1.000 | 1.000 | 1.000 | ... |
| ESE\_2 | <--- | Sm | 1.028 | .969 | 1.091 | .000 |
| ESE\_3 | <--- | Sm | .982 | .924 | 1.048 | .000 |
| ESE\_4 | <--- | Sm | 1.047 | .986 | 1.110 | .000 |
| ESE\_5 | <--- | Sm | 1.033 | .973 | 1.097 | .000 |
| ESE\_6 | <--- | Sm | 1.028 | .970 | 1.094 | .000 |
| ESE\_7 | <--- | Ss | 1.000 | 1.000 | 1.000 | ... |
| ESE\_8 | <--- | Ss | 1.005 | .952 | 1.066 | .000 |
| ESE\_9 | <--- | Ss | .947 | .893 | 1.006 | .000 |
| ESE\_10 | <--- | Ss | .981 | .926 | 1.043 | .000 |
| EE\_1 | <--- | Vp | 1.000 | 1.000 | 1.000 | ... |
| EE\_2 | <--- | Vp | 1.020 | .961 | 1.083 | .000 |
| EE\_3 | <--- | Vp | 1.049 | .995 | 1.111 | .000 |
| EE\_4 | <--- | Vp | 1.022 | .962 | 1.086 | .000 |
| EE\_5 | <--- | Vp | 1.027 | .966 | 1.090 | .000 |
| EE\_6 | <--- | Vp | 1.037 | .977 | 1.101 | .000 |
| EE\_7 | <--- | Fs | 1.000 | 1.000 | 1.000 | ... |
| EE\_8 | <--- | Fs | .972 | .917 | 1.035 | .000 |
| EE\_9 | <--- | Fs | 1.007 | .952 | 1.066 | .000 |
| EE\_10 | <--- | Fs | 1.008 | .949 | 1.068 | .000 |
| EE\_11 | <--- | Fs | 1.003 | .945 | 1.063 | .000 |
| EE\_12 | <--- | Fs | .994 | .938 | 1.058 | .000 |
| EE\_13 | <--- | Vc | 1.000 | 1.000 | 1.000 | ... |
| EE\_14 | <--- | Vc | .973 | .922 | 1.029 | .000 |
| EE\_15 | <--- | Vc | .989 | .937 | 1.044 | .000 |
| EE\_16 | <--- | Vc | .977 | .926 | 1.034 | .000 |
| EE\_17 | <--- | Vc | .970 | .914 | 1.029 | .000 |
| EE\_18 | <--- | Pa | 1.000 | 1.000 | 1.000 | ... |
| EE\_19 | <--- | Pa | .973 | .916 | 1.037 | .000 |
| EE\_20 | <--- | Pa | .990 | .931 | 1.054 | .000 |

## Standardized Regression Weights: (Group number 1 - Default model)

| Parameter | | | Estimate | Lower | Upper | P |
| --- | --- | --- | --- | --- | --- | --- |
| EOE | <--- | AISE | .547 | .512 | .580 | .000 |
| EOE | <--- | Gender | -.035 | -.074 | .003 | .071 |
| EOE | <--- | Age | .034 | -.004 | .073 | .085 |
| EOE | <--- | Grade | .027 | -.010 | .064 | .138 |
| ESE | <--- | AISE | .428 | .374 | .480 | .000 |
| ESE | <--- | EOE | .301 | .247 | .355 | .000 |
| ESE | <--- | Gender | -.049 | -.094 | -.006 | .025 |
| ESE | <--- | Age | -.001 | -.042 | .041 | .983 |
| ESE | <--- | Grade | .018 | -.024 | .060 | .400 |
| EE | <--- | ESE | .231 | .163 | .301 | .000 |
| EE | <--- | EOE | .206 | .155 | .262 | .000 |
| EE | <--- | AISE | .249 | .188 | .309 | .000 |
| EE | <--- | Gender | -.054 | -.092 | -.013 | .007 |
| EE | <--- | Age | .032 | -.007 | .070 | .107 |
| EE | <--- | Grade | .027 | -.013 | .066 | .174 |
| Sm | <--- | ESE | .811 | .777 | .844 | .000 |
| Ss | <--- | ESE | .784 | .748 | .819 | .000 |
| Vp | <--- | EE | .807 | .782 | .830 | .000 |
| Fs | <--- | EE | .813 | .789 | .835 | .000 |
| Vc | <--- | EE | .824 | .801 | .847 | .000 |
| Pa | <--- | EE | .803 | .774 | .831 | .000 |
| AISE\_1 | <--- | AISE | .721 | .698 | .743 | .000 |
| AISE\_2 | <--- | AISE | .757 | .736 | .776 | .000 |
| AISE\_3 | <--- | AISE | .736 | .713 | .755 | .001 |
| AISE\_4 | <--- | AISE | .748 | .726 | .768 | .000 |
| AISE\_5 | <--- | AISE | .760 | .739 | .780 | .001 |
| AISE\_6 | <--- | AISE | .752 | .731 | .773 | .000 |
| EOE\_1 | <--- | EOE | .720 | .697 | .740 | .000 |
| EOE\_2 | <--- | EOE | .727 | .704 | .749 | .000 |
| EOE\_3 | <--- | EOE | .721 | .698 | .742 | .000 |
| EOE\_4 | <--- | EOE | .724 | .702 | .744 | .000 |
| EOE\_5 | <--- | EOE | .738 | .717 | .758 | .000 |
| EOE\_6 | <--- | EOE | .716 | .693 | .737 | .000 |
| EOE\_7 | <--- | EOE | .745 | .725 | .765 | .000 |
| EOE\_8 | <--- | EOE | .728 | .707 | .748 | .000 |
| EOE\_9 | <--- | EOE | .718 | .697 | .740 | .000 |
| ESE\_1 | <--- | Sm | .711 | .687 | .734 | .001 |
| ESE\_2 | <--- | Sm | .733 | .708 | .756 | .001 |
| ESE\_3 | <--- | Sm | .713 | .688 | .736 | .000 |
| ESE\_4 | <--- | Sm | .739 | .716 | .760 | .001 |
| ESE\_5 | <--- | Sm | .739 | .717 | .760 | .000 |
| ESE\_6 | <--- | Sm | .721 | .697 | .743 | .000 |
| ESE\_7 | <--- | Ss | .744 | .722 | .766 | .000 |
| ESE\_8 | <--- | Ss | .748 | .724 | .772 | .000 |
| ESE\_9 | <--- | Ss | .716 | .692 | .740 | .000 |
| ESE\_10 | <--- | Ss | .735 | .708 | .758 | .000 |
| EE\_1 | <--- | Vp | .719 | .694 | .743 | .000 |
| EE\_2 | <--- | Vp | .722 | .698 | .744 | .000 |
| EE\_3 | <--- | Vp | .744 | .722 | .764 | .000 |
| EE\_4 | <--- | Vp | .732 | .709 | .752 | .000 |
| EE\_5 | <--- | Vp | .726 | .703 | .748 | .000 |
| EE\_6 | <--- | Vp | .740 | .718 | .761 | .000 |
| EE\_7 | <--- | Fs | .729 | .706 | .749 | .001 |
| EE\_8 | <--- | Fs | .715 | .691 | .737 | .000 |
| EE\_9 | <--- | Fs | .739 | .717 | .761 | .000 |
| EE\_10 | <--- | Fs | .737 | .714 | .757 | .000 |
| EE\_11 | <--- | Fs | .736 | .712 | .757 | .000 |
| EE\_12 | <--- | Fs | .735 | .712 | .756 | .000 |
| EE\_13 | <--- | Vc | .750 | .727 | .771 | .001 |
| EE\_14 | <--- | Vc | .734 | .711 | .755 | .001 |
| EE\_15 | <--- | Vc | .749 | .726 | .769 | .000 |
| EE\_16 | <--- | Vc | .735 | .713 | .756 | .000 |
| EE\_17 | <--- | Vc | .738 | .714 | .760 | .000 |
| EE\_18 | <--- | Pa | .738 | .711 | .762 | .000 |
| EE\_19 | <--- | Pa | .715 | .688 | .740 | .000 |
| EE\_20 | <--- | Pa | .723 | .696 | .748 | .000 |

## Covariances: (Group number 1 - Default model)

| Parameter | | | Estimate | Lower | Upper | P |
| --- | --- | --- | --- | --- | --- | --- |
| Gender | <--> | Age | .006 | -.013 | .023 | .557 |
| Gender | <--> | Grade | -.006 | -.023 | .012 | .553 |
| Age | <--> | Grade | .017 | -.016 | .048 | .317 |
| AISE | <--> | Gender | -.017 | -.035 | .000 | .051 |
| AISE | <--> | Age | .070 | .039 | .101 | .000 |
| AISE | <--> | Grade | .015 | -.015 | .045 | .316 |

## Correlations: (Group number 1 - Default model)

| Parameter | | | Estimate | Lower | Upper | P |
| --- | --- | --- | --- | --- | --- | --- |
| Gender | <--> | Age | .012 | -.028 | .052 | .553 |
| Gender | <--> | Grade | -.013 | -.054 | .029 | .553 |
| Age | <--> | Grade | .021 | -.020 | .062 | .316 |
| AISE | <--> | Gender | -.045 | -.090 | .000 | .050 |
| AISE | <--> | Age | .098 | .055 | .140 | .000 |
| AISE | <--> | Grade | .023 | -.021 | .065 | .318 |

## Variances: (Group number 1 - Default model)

| Parameter | | | Estimate | Lower | Upper | P |
| --- | --- | --- | --- | --- | --- | --- |
| AISE |  |  | .623 | .566 | .678 | .000 |
| Gender |  |  | .242 | .238 | .245 | .000 |
| Age |  |  | .816 | .778 | .854 | .000 |
| Grade |  |  | .743 | .725 | .762 | .000 |
| e52 |  |  | .190 | .172 | .211 | .000 |
| e53 |  |  | .115 | .099 | .134 | .000 |
| e54 |  |  | .131 | .114 | .150 | .000 |
| e46 |  |  | .103 | .085 | .124 | .000 |
| e47 |  |  | .123 | .103 | .143 | .000 |
| e48 |  |  | .108 | .094 | .122 | .000 |
| e49 |  |  | .110 | .096 | .125 | .000 |
| e50 |  |  | .107 | .093 | .123 | .000 |
| e51 |  |  | .106 | .090 | .123 | .000 |
| e1 |  |  | .576 | .538 | .615 | .000 |
| e2 |  |  | .449 | .420 | .480 | .000 |
| e3 |  |  | .485 | .455 | .519 | .000 |
| e4 |  |  | .483 | .451 | .518 | .000 |
| e5 |  |  | .415 | .388 | .445 | .000 |
| e6 |  |  | .477 | .445 | .511 | .000 |
| e7 |  |  | .255 | .240 | .272 | .000 |
| e8 |  |  | .251 | .236 | .268 | .000 |
| e9 |  |  | .255 | .239 | .271 | .000 |
| e10 |  |  | .246 | .231 | .262 | .000 |
| e11 |  |  | .245 | .230 | .261 | .000 |
| e12 |  |  | .256 | .241 | .272 | .000 |
| e13 |  |  | .233 | .219 | .247 | .000 |
| e14 |  |  | .256 | .241 | .272 | .000 |
| e15 |  |  | .258 | .242 | .275 | .000 |
| e16 |  |  | .296 | .276 | .316 | .000 |
| e17 |  |  | .275 | .257 | .296 | .000 |
| e18 |  |  | .283 | .265 | .302 | .000 |
| e19 |  |  | .275 | .258 | .294 | .000 |
| e20 |  |  | .269 | .252 | .287 | .000 |
| e21 |  |  | .296 | .278 | .317 | .000 |
| e22 |  |  | .257 | .239 | .276 | .000 |
| e23 |  |  | .255 | .236 | .276 | .000 |
| e24 |  |  | .272 | .254 | .292 | .000 |
| e25 |  |  | .262 | .243 | .282 | .000 |
| e26 |  |  | .288 | .269 | .307 | .000 |
| e27 |  |  | .296 | .277 | .316 | .000 |
| e28 |  |  | .275 | .257 | .294 | .000 |
| e29 |  |  | .280 | .262 | .298 | .000 |
| e30 |  |  | .293 | .272 | .314 | .000 |
| e31 |  |  | .274 | .256 | .293 | .000 |
| e32 |  |  | .287 | .270 | .307 | .000 |
| e33 |  |  | .294 | .276 | .313 | .000 |
| e34 |  |  | .273 | .256 | .292 | .000 |
| e35 |  |  | .278 | .261 | .297 | .000 |
| e36 |  |  | .277 | .259 | .297 | .000 |
| e37 |  |  | .273 | .255 | .292 | .000 |
| e38 |  |  | .260 | .243 | .279 | .000 |
| e39 |  |  | .271 | .253 | .290 | .000 |
| e40 |  |  | .256 | .239 | .275 | .000 |
| e41 |  |  | .271 | .253 | .290 | .000 |
| e42 |  |  | .262 | .245 | .281 | .000 |
| e43 |  |  | .250 | .231 | .270 | .000 |
| e44 |  |  | .270 | .250 | .292 | .000 |
| e45 |  |  | .266 | .247 | .288 | .000 |

## User-defined estimands: (Group number 1 - Default model)

| Parameter | | | Estimate | Lower | Upper | P |
| --- | --- | --- | --- | --- | --- | --- |
| MedIndirect1 |  |  | .099 | .068 | .134 | .000 |
| MedDirect1 |  |  | .249 | .188 | .309 | .000 |
| MedTotal1 |  |  | .348 | .297 | .399 | .000 |
| MedIndirect2 |  |  | .113 | .084 | .143 | .000 |
| MedDirect2 |  |  | .249 | .188 | .309 | .000 |
| MedTotal2 |  |  | .362 | .300 | .420 | .000 |
| MedIndirect3 |  |  | .250 | .209 | .292 | .000 |
| MedDirect3 |  |  | .249 | .188 | .309 | .000 |
| MedTotal3 |  |  | .499 | .462 | .537 | .000 |

## Matrices (Group number 1 - Default model)

## Total Effects (Group number 1 - Default model)

## Total Effects - Lower Bounds (BC) (Group number 1 - Default model)

|  | Grade | Age | Gender | AISE | EOE | ESE | EE | Pa | Vc | Fs | Vp | Ss | Sm |
| --- | --- | --- | --- | --- | --- | --- | --- | --- | --- | --- | --- | --- | --- |
| EOE | -.006 | -.002 | -.078 | .331 | .000 | .000 | .000 | .000 | .000 | .000 | .000 | .000 | .000 |
| ESE | -.009 | -.016 | -.097 | .302 | .207 | .000 | .000 | .000 | .000 | .000 | .000 | .000 | .000 |
| EE | -.001 | .001 | -.104 | .253 | .191 | .160 | .000 | .000 | .000 | .000 | .000 | .000 | .000 |
| Pa | -.001 | .001 | -.102 | .248 | .186 | .158 | .895 | .000 | .000 | .000 | .000 | .000 | .000 |
| Vc | -.001 | .001 | -.112 | .271 | .204 | .171 | .979 | .000 | .000 | .000 | .000 | .000 | .000 |
| Fs | -.001 | .001 | -.108 | .263 | .199 | .167 | .953 | .000 | .000 | .000 | .000 | .000 | .000 |
| Vp | -.001 | .001 | -.104 | .253 | .191 | .160 | 1.000 | .000 | .000 | .000 | .000 | .000 | .000 |
| Ss | -.009 | -.016 | -.094 | .302 | .207 | .902 | .000 | .000 | .000 | .000 | .000 | .000 | .000 |
| Sm | -.009 | -.016 | -.097 | .302 | .207 | 1.000 | .000 | .000 | .000 | .000 | .000 | .000 | .000 |
| EE\_20 | -.001 | .001 | -.101 | .246 | .185 | .156 | .891 | .931 | .000 | .000 | .000 | .000 | .000 |
| EE\_19 | -.001 | .001 | -.100 | .242 | .181 | .153 | .877 | .916 | .000 | .000 | .000 | .000 | .000 |
| EE\_18 | -.001 | .001 | -.102 | .248 | .186 | .158 | .895 | 1.000 | .000 | .000 | .000 | .000 | .000 |
| EE\_17 | -.001 | .001 | -.108 | .263 | .198 | .166 | .950 | .000 | .914 | .000 | .000 | .000 | .000 |
| EE\_16 | -.001 | .001 | -.108 | .265 | .199 | .167 | .956 | .000 | .926 | .000 | .000 | .000 | .000 |
| EE\_15 | -.001 | .001 | -.109 | .268 | .201 | .169 | .969 | .000 | .937 | .000 | .000 | .000 | .000 |
| EE\_14 | -.001 | .001 | -.107 | .264 | .199 | .168 | .952 | .000 | .922 | .000 | .000 | .000 | .000 |
| EE\_13 | -.001 | .001 | -.112 | .271 | .204 | .171 | .979 | .000 | 1.000 | .000 | .000 | .000 | .000 |
| EE\_12 | -.001 | .001 | -.107 | .262 | .197 | .166 | .946 | .000 | .000 | .938 | .000 | .000 | .000 |
| EE\_11 | -.001 | .001 | -.108 | .263 | .199 | .169 | .953 | .000 | .000 | .945 | .000 | .000 | .000 |
| EE\_10 | -.001 | .001 | -.109 | .264 | .200 | .169 | .958 | .000 | .000 | .949 | .000 | .000 | .000 |
| EE\_9 | -.001 | .001 | -.109 | .265 | .199 | .168 | .959 | .000 | .000 | .952 | .000 | .000 | .000 |
| EE\_8 | -.001 | .001 | -.105 | .255 | .192 | .163 | .923 | .000 | .000 | .917 | .000 | .000 | .000 |
| EE\_7 | -.001 | .001 | -.108 | .263 | .199 | .167 | .953 | .000 | .000 | 1.000 | .000 | .000 | .000 |
| EE\_6 | -.001 | .001 | -.109 | .263 | .199 | .167 | .977 | .000 | .000 | .000 | .977 | .000 | .000 |
| EE\_5 | -.001 | .001 | -.107 | .261 | .197 | .165 | .966 | .000 | .000 | .000 | .966 | .000 | .000 |
| EE\_4 | -.001 | .001 | -.107 | .260 | .196 | .165 | .962 | .000 | .000 | .000 | .962 | .000 | .000 |
| EE\_3 | -.001 | .001 | -.109 | .267 | .201 | .169 | .995 | .000 | .000 | .000 | .995 | .000 | .000 |
| EE\_2 | -.001 | .001 | -.106 | .259 | .195 | .165 | .961 | .000 | .000 | .000 | .961 | .000 | .000 |
| EE\_1 | -.001 | .001 | -.104 | .253 | .191 | .160 | 1.000 | .000 | .000 | .000 | 1.000 | .000 | .000 |
| ESE\_10 | -.008 | -.016 | -.092 | .293 | .205 | .879 | .000 | .000 | .000 | .000 | .000 | .926 | .000 |
| ESE\_9 | -.008 | -.016 | -.089 | .284 | .196 | .853 | .000 | .000 | .000 | .000 | .000 | .893 | .000 |
| ESE\_8 | -.009 | -.017 | -.095 | .302 | .208 | .907 | .000 | .000 | .000 | .000 | .000 | .952 | .000 |
| ESE\_7 | -.009 | -.016 | -.094 | .302 | .207 | .902 | .000 | .000 | .000 | .000 | .000 | 1.000 | .000 |
| ESE\_6 | -.009 | -.017 | -.099 | .312 | .214 | .970 | .000 | .000 | .000 | .000 | .000 | .000 | .970 |
| ESE\_5 | -.009 | -.017 | -.100 | .312 | .214 | .973 | .000 | .000 | .000 | .000 | .000 | .000 | .973 |
| ESE\_4 | -.009 | -.017 | -.100 | .318 | .216 | .986 | .000 | .000 | .000 | .000 | .000 | .000 | .986 |
| ESE\_3 | -.009 | -.016 | -.094 | .297 | .204 | .924 | .000 | .000 | .000 | .000 | .000 | .000 | .924 |
| ESE\_2 | -.009 | -.017 | -.099 | .311 | .213 | .969 | .000 | .000 | .000 | .000 | .000 | .000 | .969 |
| ESE\_1 | -.009 | -.016 | -.097 | .302 | .207 | 1.000 | .000 | .000 | .000 | .000 | .000 | .000 | 1.000 |
| EOE\_9 | -.006 | -.002 | -.079 | .331 | .943 | .000 | .000 | .000 | .000 | .000 | .000 | .000 | .000 |
| EOE\_8 | -.006 | -.002 | -.080 | .339 | .969 | .000 | .000 | .000 | .000 | .000 | .000 | .000 | .000 |
| EOE\_7 | -.006 | -.002 | -.081 | .341 | .973 | .000 | .000 | .000 | .000 | .000 | .000 | .000 | .000 |
| EOE\_6 | -.006 | -.002 | -.078 | .329 | .938 | .000 | .000 | .000 | .000 | .000 | .000 | .000 | .000 |
| EOE\_5 | -.006 | -.002 | -.081 | .342 | .974 | .000 | .000 | .000 | .000 | .000 | .000 | .000 | .000 |
| EOE\_4 | -.006 | -.002 | -.078 | .328 | .938 | .000 | .000 | .000 | .000 | .000 | .000 | .000 | .000 |
| EOE\_3 | -.006 | -.002 | -.079 | .332 | .947 | .000 | .000 | .000 | .000 | .000 | .000 | .000 | .000 |
| EOE\_2 | -.006 | -.002 | -.080 | .336 | .961 | .000 | .000 | .000 | .000 | .000 | .000 | .000 | .000 |
| EOE\_1 | -.006 | -.002 | -.078 | .331 | 1.000 | .000 | .000 | .000 | .000 | .000 | .000 | .000 | .000 |
| AISE\_6 | .000 | .000 | .000 | .948 | .000 | .000 | .000 | .000 | .000 | .000 | .000 | .000 | .000 |
| AISE\_5 | .000 | .000 | .000 | .905 | .000 | .000 | .000 | .000 | .000 | .000 | .000 | .000 | .000 |
| AISE\_4 | .000 | .000 | .000 | .939 | .000 | .000 | .000 | .000 | .000 | .000 | .000 | .000 | .000 |
| AISE\_3 | .000 | .000 | .000 | .909 | .000 | .000 | .000 | .000 | .000 | .000 | .000 | .000 | .000 |
| AISE\_2 | .000 | .000 | .000 | .932 | .000 | .000 | .000 | .000 | .000 | .000 | .000 | .000 | .000 |
| AISE\_1 | .000 | .000 | .000 | 1.000 | .000 | .000 | .000 | .000 | .000 | .000 | .000 | .000 | .000 |

## Total Effects - Upper Bounds (BC) (Group number 1 - Default model)

|  | Grade | Age | Gender | AISE | EOE | ESE | EE | Pa | Vc | Fs | Vp | Ss | Sm |
| --- | --- | --- | --- | --- | --- | --- | --- | --- | --- | --- | --- | --- | --- |
| EOE | .039 | .043 | .003 | .395 | .000 | .000 | .000 | .000 | .000 | .000 | .000 | .000 | .000 |
| ESE | .036 | .027 | -.013 | .368 | .308 | .000 | .000 | .000 | .000 | .000 | .000 | .000 | .000 |
| EE | .042 | .041 | -.030 | .316 | .283 | .308 | .000 | .000 | .000 | .000 | .000 | .000 | .000 |
| Pa | .041 | .040 | -.029 | .308 | .279 | .305 | 1.063 | .000 | .000 | .000 | .000 | .000 | .000 |
| Vc | .044 | .043 | -.032 | .334 | .301 | .329 | 1.154 | .000 | .000 | .000 | .000 | .000 | .000 |
| Fs | .043 | .042 | -.032 | .328 | .295 | .321 | 1.121 | .000 | .000 | .000 | .000 | .000 | .000 |
| Vp | .042 | .041 | -.030 | .316 | .283 | .308 | 1.000 | .000 | .000 | .000 | .000 | .000 | .000 |
| Ss | .036 | .027 | -.014 | .367 | .305 | 1.096 | .000 | .000 | .000 | .000 | .000 | .000 | .000 |
| Sm | .036 | .027 | -.013 | .368 | .308 | 1.000 | .000 | .000 | .000 | .000 | .000 | .000 | .000 |
| EE\_20 | .040 | .040 | -.029 | .305 | .276 | .301 | 1.051 | 1.054 | .000 | .000 | .000 | .000 | .000 |
| EE\_19 | .039 | .039 | -.029 | .300 | .272 | .296 | 1.031 | 1.037 | .000 | .000 | .000 | .000 | .000 |
| EE\_18 | .041 | .040 | -.029 | .308 | .279 | .305 | 1.063 | 1.000 | .000 | .000 | .000 | .000 | .000 |
| EE\_17 | .043 | .042 | -.031 | .324 | .294 | .320 | 1.123 | .000 | 1.029 | .000 | .000 | .000 | .000 |
| EE\_16 | .043 | .042 | -.031 | .327 | .296 | .323 | 1.127 | .000 | 1.034 | .000 | .000 | .000 | .000 |
| EE\_15 | .044 | .043 | -.032 | .331 | .298 | .325 | 1.140 | .000 | 1.044 | .000 | .000 | .000 | .000 |
| EE\_14 | .043 | .042 | -.031 | .327 | .293 | .319 | 1.118 | .000 | 1.029 | .000 | .000 | .000 | .000 |
| EE\_13 | .044 | .043 | -.032 | .334 | .301 | .329 | 1.154 | .000 | 1.000 | .000 | .000 | .000 | .000 |
| EE\_12 | .043 | .042 | -.031 | .325 | .293 | .319 | 1.118 | .000 | .000 | 1.058 | .000 | .000 | .000 |
| EE\_11 | .043 | .042 | -.031 | .328 | .294 | .324 | 1.123 | .000 | .000 | 1.063 | .000 | .000 | .000 |
| EE\_10 | .044 | .042 | -.032 | .328 | .295 | .324 | 1.134 | .000 | .000 | 1.068 | .000 | .000 | .000 |
| EE\_9 | .043 | .042 | -.032 | .328 | .297 | .326 | 1.133 | .000 | .000 | 1.066 | .000 | .000 | .000 |
| EE\_8 | .041 | .041 | -.030 | .318 | .286 | .312 | 1.096 | .000 | .000 | 1.035 | .000 | .000 | .000 |
| EE\_7 | .043 | .042 | -.032 | .328 | .295 | .321 | 1.121 | .000 | .000 | 1.000 | .000 | .000 | .000 |
| EE\_6 | .043 | .043 | -.032 | .326 | .294 | .322 | 1.101 | .000 | .000 | .000 | 1.101 | .000 | .000 |
| EE\_5 | .043 | .042 | -.031 | .323 | .291 | .318 | 1.090 | .000 | .000 | .000 | 1.090 | .000 | .000 |
| EE\_4 | .043 | .042 | -.031 | .322 | .289 | .317 | 1.086 | .000 | .000 | .000 | 1.086 | .000 | .000 |
| EE\_3 | .044 | .043 | -.032 | .330 | .297 | .325 | 1.111 | .000 | .000 | .000 | 1.111 | .000 | .000 |
| EE\_2 | .043 | .042 | -.031 | .322 | .291 | .317 | 1.083 | .000 | .000 | .000 | 1.083 | .000 | .000 |
| EE\_1 | .042 | .041 | -.030 | .316 | .283 | .308 | 1.000 | .000 | .000 | .000 | 1.000 | .000 | .000 |
| ESE\_10 | .035 | .026 | -.013 | .362 | .300 | 1.079 | .000 | .000 | .000 | .000 | .000 | 1.043 | .000 |
| ESE\_9 | .034 | .025 | -.013 | .348 | .290 | 1.042 | .000 | .000 | .000 | .000 | .000 | 1.006 | .000 |
| ESE\_8 | .036 | .027 | -.014 | .371 | .306 | 1.107 | .000 | .000 | .000 | .000 | .000 | 1.066 | .000 |
| ESE\_7 | .036 | .027 | -.014 | .367 | .305 | 1.096 | .000 | .000 | .000 | .000 | .000 | 1.000 | .000 |
| ESE\_6 | .037 | .028 | -.014 | .380 | .316 | 1.094 | .000 | .000 | .000 | .000 | .000 | .000 | 1.094 |
| ESE\_5 | .037 | .028 | -.014 | .381 | .318 | 1.097 | .000 | .000 | .000 | .000 | .000 | .000 | 1.097 |
| ESE\_4 | .038 | .028 | -.014 | .385 | .323 | 1.110 | .000 | .000 | .000 | .000 | .000 | .000 | 1.110 |
| ESE\_3 | .035 | .026 | -.013 | .363 | .302 | 1.048 | .000 | .000 | .000 | .000 | .000 | .000 | 1.048 |
| ESE\_2 | .037 | .028 | -.014 | .379 | .316 | 1.091 | .000 | .000 | .000 | .000 | .000 | .000 | 1.091 |
| ESE\_1 | .036 | .027 | -.013 | .368 | .308 | 1.000 | .000 | .000 | .000 | .000 | .000 | .000 | 1.000 |
| EOE\_9 | .039 | .042 | .003 | .398 | 1.061 | .000 | .000 | .000 | .000 | .000 | .000 | .000 | .000 |
| EOE\_8 | .040 | .044 | .003 | .407 | 1.086 | .000 | .000 | .000 | .000 | .000 | .000 | .000 | .000 |
| EOE\_7 | .041 | .044 | .003 | .408 | 1.091 | .000 | .000 | .000 | .000 | .000 | .000 | .000 | .000 |
| EOE\_6 | .039 | .042 | .003 | .393 | 1.052 | .000 | .000 | .000 | .000 | .000 | .000 | .000 | .000 |
| EOE\_5 | .040 | .044 | .003 | .408 | 1.092 | .000 | .000 | .000 | .000 | .000 | .000 | .000 | .000 |
| EOE\_4 | .039 | .042 | .003 | .395 | 1.051 | .000 | .000 | .000 | .000 | .000 | .000 | .000 | .000 |
| EOE\_3 | .039 | .043 | .003 | .397 | 1.058 | .000 | .000 | .000 | .000 | .000 | .000 | .000 | .000 |
| EOE\_2 | .040 | .043 | .003 | .402 | 1.071 | .000 | .000 | .000 | .000 | .000 | .000 | .000 | .000 |
| EOE\_1 | .039 | .043 | .003 | .395 | 1.000 | .000 | .000 | .000 | .000 | .000 | .000 | .000 | .000 |
| AISE\_6 | .000 | .000 | .000 | 1.052 | .000 | .000 | .000 | .000 | .000 | .000 | .000 | .000 | .000 |
| AISE\_5 | .000 | .000 | .000 | 1.009 | .000 | .000 | .000 | .000 | .000 | .000 | .000 | .000 | .000 |
| AISE\_4 | .000 | .000 | .000 | 1.045 | .000 | .000 | .000 | .000 | .000 | .000 | .000 | .000 | .000 |
| AISE\_3 | .000 | .000 | .000 | 1.012 | .000 | .000 | .000 | .000 | .000 | .000 | .000 | .000 | .000 |
| AISE\_2 | .000 | .000 | .000 | 1.036 | .000 | .000 | .000 | .000 | .000 | .000 | .000 | .000 | .000 |
| AISE\_1 | .000 | .000 | .000 | 1.000 | .000 | .000 | .000 | .000 | .000 | .000 | .000 | .000 | .000 |

## Total Effects - Two Tailed Significance (BC) (Group number 1 - Default model)

|  | Grade | Age | Gender | AISE | EOE | ESE | EE | Pa | Vc | Fs | Vp | Ss | Sm |
| --- | --- | --- | --- | --- | --- | --- | --- | --- | --- | --- | --- | --- | --- |
| EOE | .139 | .082 | .071 | .001 | ... | ... | ... | ... | ... | ... | ... | ... | ... |
| ESE | .245 | .645 | .009 | .000 | .000 | ... | ... | ... | ... | ... | ... | ... | ... |
| EE | .059 | .040 | .001 | .000 | .000 | .000 | ... | ... | ... | ... | ... | ... | ... |
| Pa | .056 | .042 | .001 | .000 | .000 | .000 | .001 | ... | ... | ... | ... | ... | ... |
| Vc | .057 | .042 | .001 | .000 | .000 | .000 | .000 | ... | ... | ... | ... | ... | ... |
| Fs | .058 | .042 | .001 | .000 | .000 | .000 | .000 | ... | ... | ... | ... | ... | ... |
| Vp | .059 | .040 | .001 | .000 | .000 | .000 | ... | ... | ... | ... | ... | ... | ... |
| Ss | .249 | .652 | .009 | .000 | .000 | .000 | ... | ... | ... | ... | ... | ... | ... |
| Sm | .245 | .645 | .009 | .000 | .000 | ... | ... | ... | ... | ... | ... | ... | ... |
| EE\_20 | .057 | .042 | .001 | .000 | .000 | .000 | .000 | .000 | ... | ... | ... | ... | ... |
| EE\_19 | .058 | .043 | .001 | .000 | .000 | .000 | .000 | .000 | ... | ... | ... | ... | ... |
| EE\_18 | .056 | .042 | .001 | .000 | .000 | .000 | .001 | ... | ... | ... | ... | ... | ... |
| EE\_17 | .058 | .041 | .001 | .000 | .000 | .000 | .000 | ... | .000 | ... | ... | ... | ... |
| EE\_16 | .058 | .042 | .001 | .000 | .000 | .000 | .000 | ... | .000 | ... | ... | ... | ... |
| EE\_15 | .057 | .041 | .001 | .000 | .000 | .000 | .000 | ... | .000 | ... | ... | ... | ... |
| EE\_14 | .059 | .042 | .001 | .000 | .000 | .000 | .000 | ... | .000 | ... | ... | ... | ... |
| EE\_13 | .057 | .042 | .001 | .000 | .000 | .000 | .000 | ... | ... | ... | ... | ... | ... |
| EE\_12 | .058 | .041 | .001 | .000 | .000 | .000 | .000 | ... | ... | .000 | ... | ... | ... |
| EE\_11 | .056 | .041 | .001 | .000 | .000 | .000 | .001 | ... | ... | .000 | ... | ... | ... |
| EE\_10 | .056 | .043 | .001 | .000 | .000 | .000 | .000 | ... | ... | .000 | ... | ... | ... |
| EE\_9 | .058 | .042 | .001 | .000 | .000 | .000 | .000 | ... | ... | .000 | ... | ... | ... |
| EE\_8 | .057 | .042 | .001 | .000 | .000 | .000 | .000 | ... | ... | .000 | ... | ... | ... |
| EE\_7 | .058 | .042 | .001 | .000 | .000 | .000 | .000 | ... | ... | ... | ... | ... | ... |
| EE\_6 | .058 | .041 | .001 | .000 | .000 | .000 | .000 | ... | ... | ... | .000 | ... | ... |
| EE\_5 | .058 | .039 | .001 | .000 | .000 | .000 | .000 | ... | ... | ... | .000 | ... | ... |
| EE\_4 | .058 | .041 | .001 | .000 | .000 | .000 | .000 | ... | ... | ... | .000 | ... | ... |
| EE\_3 | .059 | .042 | .001 | .000 | .000 | .000 | .000 | ... | ... | ... | .000 | ... | ... |
| EE\_2 | .058 | .042 | .001 | .000 | .000 | .000 | .000 | ... | ... | ... | .000 | ... | ... |
| EE\_1 | .059 | .040 | .001 | .000 | .000 | .000 | ... | ... | ... | ... | ... | ... | ... |
| ESE\_10 | .246 | .646 | .009 | .000 | .000 | .000 | ... | ... | ... | ... | ... | .000 | ... |
| ESE\_9 | .245 | .649 | .009 | .000 | .000 | .000 | ... | ... | ... | ... | ... | .000 | ... |
| ESE\_8 | .245 | .648 | .009 | .000 | .000 | .000 | ... | ... | ... | ... | ... | .000 | ... |
| ESE\_7 | .249 | .652 | .009 | .000 | .000 | .000 | ... | ... | ... | ... | ... | ... | ... |
| ESE\_6 | .249 | .644 | .009 | .000 | .000 | .000 | ... | ... | ... | ... | ... | ... | .000 |
| ESE\_5 | .246 | .643 | .009 | .000 | .000 | .000 | ... | ... | ... | ... | ... | ... | .000 |
| ESE\_4 | .249 | .644 | .010 | .000 | .000 | .000 | ... | ... | ... | ... | ... | ... | .000 |
| ESE\_3 | .247 | .650 | .009 | .000 | .000 | .000 | ... | ... | ... | ... | ... | ... | .000 |
| ESE\_2 | .251 | .649 | .009 | .000 | .000 | .000 | ... | ... | ... | ... | ... | ... | .000 |
| ESE\_1 | .245 | .645 | .009 | .000 | .000 | ... | ... | ... | ... | ... | ... | ... | ... |
| EOE\_9 | .138 | .085 | .069 | .000 | .000 | ... | ... | ... | ... | ... | ... | ... | ... |
| EOE\_8 | .138 | .084 | .070 | .000 | .000 | ... | ... | ... | ... | ... | ... | ... | ... |
| EOE\_7 | .139 | .086 | .071 | .000 | .000 | ... | ... | ... | ... | ... | ... | ... | ... |
| EOE\_6 | .141 | .082 | .072 | .000 | .000 | ... | ... | ... | ... | ... | ... | ... | ... |
| EOE\_5 | .136 | .083 | .071 | .000 | .000 | ... | ... | ... | ... | ... | ... | ... | ... |
| EOE\_4 | .139 | .084 | .071 | .000 | .000 | ... | ... | ... | ... | ... | ... | ... | ... |
| EOE\_3 | .136 | .083 | .070 | .000 | .000 | ... | ... | ... | ... | ... | ... | ... | ... |
| EOE\_2 | .137 | .085 | .071 | .000 | .000 | ... | ... | ... | ... | ... | ... | ... | ... |
| EOE\_1 | .139 | .082 | .071 | .001 | ... | ... | ... | ... | ... | ... | ... | ... | ... |
| AISE\_6 | ... | ... | ... | .000 | ... | ... | ... | ... | ... | ... | ... | ... | ... |
| AISE\_5 | ... | ... | ... | .000 | ... | ... | ... | ... | ... | ... | ... | ... | ... |
| AISE\_4 | ... | ... | ... | .000 | ... | ... | ... | ... | ... | ... | ... | ... | ... |
| AISE\_3 | ... | ... | ... | .000 | ... | ... | ... | ... | ... | ... | ... | ... | ... |
| AISE\_2 | ... | ... | ... | .000 | ... | ... | ... | ... | ... | ... | ... | ... | ... |
| AISE\_1 | ... | ... | ... | ... | ... | ... | ... | ... | ... | ... | ... | ... | ... |

## Standardized Total Effects (Group number 1 - Default model)

## Standardized Total Effects - Lower Bounds (BC) (Group number 1 - Default model)

|  | Grade | Age | Gender | AISE | EOE | ESE | EE | Pa | Vc | Fs | Vp | Ss | Sm |
| --- | --- | --- | --- | --- | --- | --- | --- | --- | --- | --- | --- | --- | --- |
| EOE | -.010 | -.004 | -.074 | .512 | .000 | .000 | .000 | .000 | .000 | .000 | .000 | .000 | .000 |
| ESE | -.017 | -.033 | -.105 | .554 | .247 | .000 | .000 | .000 | .000 | .000 | .000 | .000 | .000 |
| EE | -.001 | .002 | -.113 | .462 | .226 | .163 | .000 | .000 | .000 | .000 | .000 | .000 | .000 |
| Pa | -.001 | .001 | -.091 | .366 | .181 | .131 | .774 | .000 | .000 | .000 | .000 | .000 | .000 |
| Vc | -.001 | .001 | -.094 | .378 | .186 | .133 | .801 | .000 | .000 | .000 | .000 | .000 | .000 |
| Fs | -.001 | .002 | -.092 | .373 | .184 | .133 | .789 | .000 | .000 | .000 | .000 | .000 | .000 |
| Vp | -.001 | .001 | -.091 | .370 | .182 | .131 | .782 | .000 | .000 | .000 | .000 | .000 | .000 |
| Ss | -.013 | -.026 | -.081 | .428 | .194 | .748 | .000 | .000 | .000 | .000 | .000 | .000 | .000 |
| Sm | -.014 | -.027 | -.085 | .444 | .199 | .777 | .000 | .000 | .000 | .000 | .000 | .000 | .000 |
| EE\_20 | -.001 | .001 | -.066 | .263 | .130 | .095 | .552 | .696 | .000 | .000 | .000 | .000 | .000 |
| EE\_19 | -.001 | .001 | -.066 | .259 | .129 | .093 | .544 | .688 | .000 | .000 | .000 | .000 | .000 |
| EE\_18 | -.001 | .001 | -.068 | .268 | .133 | .097 | .563 | .711 | .000 | .000 | .000 | .000 | .000 |
| EE\_17 | -.001 | .001 | -.069 | .277 | .137 | .099 | .581 | .000 | .714 | .000 | .000 | .000 | .000 |
| EE\_16 | -.001 | .001 | -.069 | .277 | .136 | .098 | .578 | .000 | .713 | .000 | .000 | .000 | .000 |
| EE\_15 | -.001 | .001 | -.070 | .281 | .139 | .100 | .590 | .000 | .726 | .000 | .000 | .000 | .000 |
| EE\_14 | -.001 | .001 | -.068 | .275 | .136 | .098 | .578 | .000 | .711 | .000 | .000 | .000 | .000 |
| EE\_13 | -.001 | .001 | -.071 | .281 | .139 | .101 | .591 | .000 | .727 | .000 | .000 | .000 | .000 |
| EE\_12 | -.001 | .001 | -.068 | .271 | .135 | .098 | .569 | .000 | .000 | .712 | .000 | .000 | .000 |
| EE\_11 | -.001 | .001 | -.068 | .271 | .135 | .098 | .571 | .000 | .000 | .712 | .000 | .000 | .000 |
| EE\_10 | -.001 | .001 | -.069 | .272 | .135 | .098 | .572 | .000 | .000 | .714 | .000 | .000 | .000 |
| EE\_9 | -.001 | .001 | -.069 | .273 | .135 | .098 | .575 | .000 | .000 | .717 | .000 | .000 | .000 |
| EE\_8 | -.001 | .001 | -.066 | .263 | .130 | .095 | .554 | .000 | .000 | .691 | .000 | .000 | .000 |
| EE\_7 | -.001 | .001 | -.067 | .269 | .133 | .097 | .567 | .000 | .000 | .706 | .000 | .000 | .000 |
| EE\_6 | -.001 | .001 | -.068 | .271 | .134 | .097 | .570 | .000 | .000 | .000 | .718 | .000 | .000 |
| EE\_5 | -.001 | .001 | -.066 | .266 | .132 | .095 | .558 | .000 | .000 | .000 | .703 | .000 | .000 |
| EE\_4 | -.001 | .001 | -.067 | .268 | .133 | .096 | .565 | .000 | .000 | .000 | .709 | .000 | .000 |
| EE\_3 | -.001 | .001 | -.068 | .273 | .135 | .098 | .574 | .000 | .000 | .000 | .722 | .000 | .000 |
| EE\_2 | -.001 | .001 | -.066 | .265 | .131 | .095 | .554 | .000 | .000 | .000 | .698 | .000 | .000 |
| EE\_1 | -.001 | .001 | -.066 | .263 | .130 | .095 | .551 | .000 | .000 | .000 | .694 | .000 | .000 |
| ESE\_10 | -.010 | -.019 | -.060 | .310 | .142 | .542 | .000 | .000 | .000 | .000 | .000 | .708 | .000 |
| ESE\_9 | -.009 | -.019 | -.058 | .305 | .139 | .531 | .000 | .000 | .000 | .000 | .000 | .692 | .000 |
| ESE\_8 | -.010 | -.020 | -.061 | .318 | .144 | .554 | .000 | .000 | .000 | .000 | .000 | .724 | .000 |
| ESE\_7 | -.010 | -.020 | -.061 | .317 | .145 | .552 | .000 | .000 | .000 | .000 | .000 | .722 | .000 |
| ESE\_6 | -.010 | -.019 | -.062 | .318 | .143 | .551 | .000 | .000 | .000 | .000 | .000 | .000 | .697 |
| ESE\_5 | -.010 | -.020 | -.064 | .325 | .146 | .566 | .000 | .000 | .000 | .000 | .000 | .000 | .717 |
| ESE\_4 | -.010 | -.020 | -.063 | .326 | .147 | .568 | .000 | .000 | .000 | .000 | .000 | .000 | .716 |
| ESE\_3 | -.010 | -.019 | -.061 | .314 | .142 | .545 | .000 | .000 | .000 | .000 | .000 | .000 | .688 |
| ESE\_2 | -.010 | -.020 | -.063 | .322 | .146 | .562 | .000 | .000 | .000 | .000 | .000 | .000 | .708 |
| ESE\_1 | -.010 | -.019 | -.061 | .312 | .141 | .544 | .000 | .000 | .000 | .000 | .000 | .000 | .687 |
| EOE\_9 | -.007 | -.003 | -.053 | .363 | .697 | .000 | .000 | .000 | .000 | .000 | .000 | .000 | .000 |
| EOE\_8 | -.007 | -.003 | -.054 | .369 | .707 | .000 | .000 | .000 | .000 | .000 | .000 | .000 | .000 |
| EOE\_7 | -.007 | -.003 | -.055 | .379 | .725 | .000 | .000 | .000 | .000 | .000 | .000 | .000 | .000 |
| EOE\_6 | -.007 | -.002 | -.053 | .364 | .693 | .000 | .000 | .000 | .000 | .000 | .000 | .000 | .000 |
| EOE\_5 | -.007 | -.003 | -.054 | .375 | .717 | .000 | .000 | .000 | .000 | .000 | .000 | .000 | .000 |
| EOE\_4 | -.007 | -.003 | -.053 | .366 | .702 | .000 | .000 | .000 | .000 | .000 | .000 | .000 | .000 |
| EOE\_3 | -.007 | -.002 | -.053 | .365 | .698 | .000 | .000 | .000 | .000 | .000 | .000 | .000 | .000 |
| EOE\_2 | -.007 | -.003 | -.053 | .369 | .704 | .000 | .000 | .000 | .000 | .000 | .000 | .000 | .000 |
| EOE\_1 | -.007 | -.003 | -.053 | .364 | .697 | .000 | .000 | .000 | .000 | .000 | .000 | .000 | .000 |
| AISE\_6 | .000 | .000 | .000 | .731 | .000 | .000 | .000 | .000 | .000 | .000 | .000 | .000 | .000 |
| AISE\_5 | .000 | .000 | .000 | .739 | .000 | .000 | .000 | .000 | .000 | .000 | .000 | .000 | .000 |
| AISE\_4 | .000 | .000 | .000 | .726 | .000 | .000 | .000 | .000 | .000 | .000 | .000 | .000 | .000 |
| AISE\_3 | .000 | .000 | .000 | .713 | .000 | .000 | .000 | .000 | .000 | .000 | .000 | .000 | .000 |
| AISE\_2 | .000 | .000 | .000 | .736 | .000 | .000 | .000 | .000 | .000 | .000 | .000 | .000 | .000 |
| AISE\_1 | .000 | .000 | .000 | .698 | .000 | .000 | .000 | .000 | .000 | .000 | .000 | .000 | .000 |

## Standardized Total Effects - Upper Bounds (BC) (Group number 1 - Default model)

|  | Grade | Age | Gender | AISE | EOE | ESE | EE | Pa | Vc | Fs | Vp | Ss | Sm |
| --- | --- | --- | --- | --- | --- | --- | --- | --- | --- | --- | --- | --- | --- |
| EOE | .064 | .073 | .003 | .580 | .000 | .000 | .000 | .000 | .000 | .000 | .000 | .000 | .000 |
| ESE | .069 | .054 | -.015 | .631 | .355 | .000 | .000 | .000 | .000 | .000 | .000 | .000 | .000 |
| EE | .080 | .082 | -.033 | .537 | .326 | .301 | .000 | .000 | .000 | .000 | .000 | .000 | .000 |
| Pa | .064 | .066 | -.026 | .435 | .264 | .243 | .831 | .000 | .000 | .000 | .000 | .000 | .000 |
| Vc | .066 | .067 | -.027 | .445 | .270 | .247 | .847 | .000 | .000 | .000 | .000 | .000 | .000 |
| Fs | .065 | .067 | -.027 | .441 | .267 | .246 | .835 | .000 | .000 | .000 | .000 | .000 | .000 |
| Vp | .064 | .066 | -.027 | .436 | .264 | .243 | .830 | .000 | .000 | .000 | .000 | .000 | .000 |
| Ss | .054 | .043 | -.012 | .501 | .280 | .819 | .000 | .000 | .000 | .000 | .000 | .000 | .000 |
| Sm | .056 | .044 | -.012 | .516 | .290 | .844 | .000 | .000 | .000 | .000 | .000 | .000 | .000 |
| EE\_20 | .046 | .048 | -.019 | .318 | .192 | .176 | .610 | .748 | .000 | .000 | .000 | .000 | .000 |
| EE\_19 | .046 | .047 | -.019 | .314 | .190 | .174 | .603 | .740 | .000 | .000 | .000 | .000 | .000 |
| EE\_18 | .048 | .049 | -.019 | .324 | .196 | .180 | .621 | .762 | .000 | .000 | .000 | .000 | .000 |
| EE\_17 | .049 | .050 | -.020 | .332 | .200 | .183 | .634 | .000 | .760 | .000 | .000 | .000 | .000 |
| EE\_16 | .048 | .050 | -.020 | .330 | .199 | .183 | .631 | .000 | .756 | .000 | .000 | .000 | .000 |
| EE\_15 | .049 | .051 | -.020 | .336 | .202 | .185 | .644 | .000 | .769 | .000 | .000 | .000 | .000 |
| EE\_14 | .048 | .049 | -.020 | .330 | .199 | .182 | .630 | .000 | .755 | .000 | .000 | .000 | .000 |
| EE\_13 | .049 | .051 | -.021 | .336 | .203 | .186 | .643 | .000 | .771 | .000 | .000 | .000 | .000 |
| EE\_12 | .048 | .049 | -.020 | .327 | .197 | .181 | .623 | .000 | .000 | .756 | .000 | .000 | .000 |
| EE\_11 | .048 | .049 | -.020 | .326 | .196 | .182 | .624 | .000 | .000 | .757 | .000 | .000 | .000 |
| EE\_10 | .048 | .049 | -.020 | .328 | .197 | .182 | .625 | .000 | .000 | .757 | .000 | .000 | .000 |
| EE\_9 | .048 | .050 | -.020 | .329 | .198 | .183 | .628 | .000 | .000 | .761 | .000 | .000 | .000 |
| EE\_8 | .046 | .048 | -.019 | .318 | .191 | .176 | .608 | .000 | .000 | .737 | .000 | .000 | .000 |
| EE\_7 | .047 | .049 | -.020 | .324 | .195 | .179 | .618 | .000 | .000 | .749 | .000 | .000 | .000 |
| EE\_6 | .048 | .049 | -.020 | .325 | .196 | .181 | .624 | .000 | .000 | .000 | .761 | .000 | .000 |
| EE\_5 | .047 | .048 | -.019 | .320 | .192 | .176 | .612 | .000 | .000 | .000 | .748 | .000 | .000 |
| EE\_4 | .047 | .048 | -.020 | .323 | .194 | .178 | .616 | .000 | .000 | .000 | .752 | .000 | .000 |
| EE\_3 | .048 | .050 | -.020 | .328 | .197 | .181 | .628 | .000 | .000 | .000 | .764 | .000 | .000 |
| EE\_2 | .047 | .048 | -.019 | .319 | .192 | .176 | .609 | .000 | .000 | .000 | .744 | .000 | .000 |
| EE\_1 | .046 | .048 | -.019 | .317 | .191 | .175 | .608 | .000 | .000 | .000 | .743 | .000 | .000 |
| ESE\_10 | .040 | .031 | -.009 | .373 | .206 | .610 | .000 | .000 | .000 | .000 | .000 | .758 | .000 |
| ESE\_9 | .039 | .031 | -.009 | .362 | .202 | .594 | .000 | .000 | .000 | .000 | .000 | .740 | .000 |
| ESE\_8 | .041 | .032 | -.009 | .379 | .210 | .618 | .000 | .000 | .000 | .000 | .000 | .772 | .000 |
| ESE\_7 | .040 | .032 | -.009 | .376 | .209 | .615 | .000 | .000 | .000 | .000 | .000 | .766 | .000 |
| ESE\_6 | .041 | .032 | -.009 | .376 | .210 | .616 | .000 | .000 | .000 | .000 | .000 | .000 | .743 |
| ESE\_5 | .042 | .033 | -.009 | .385 | .215 | .631 | .000 | .000 | .000 | .000 | .000 | .000 | .760 |
| ESE\_4 | .042 | .033 | -.009 | .384 | .215 | .631 | .000 | .000 | .000 | .000 | .000 | .000 | .760 |
| ESE\_3 | .040 | .032 | -.009 | .372 | .208 | .609 | .000 | .000 | .000 | .000 | .000 | .000 | .736 |
| ESE\_2 | .041 | .033 | -.009 | .382 | .214 | .626 | .000 | .000 | .000 | .000 | .000 | .000 | .756 |
| ESE\_1 | .040 | .031 | -.008 | .371 | .208 | .608 | .000 | .000 | .000 | .000 | .000 | .000 | .734 |
| EOE\_9 | .046 | .052 | .002 | .422 | .740 | .000 | .000 | .000 | .000 | .000 | .000 | .000 | .000 |
| EOE\_8 | .047 | .053 | .002 | .426 | .748 | .000 | .000 | .000 | .000 | .000 | .000 | .000 | .000 |
| EOE\_7 | .048 | .054 | .002 | .436 | .765 | .000 | .000 | .000 | .000 | .000 | .000 | .000 | .000 |
| EOE\_6 | .046 | .052 | .002 | .420 | .737 | .000 | .000 | .000 | .000 | .000 | .000 | .000 | .000 |
| EOE\_5 | .047 | .054 | .002 | .432 | .758 | .000 | .000 | .000 | .000 | .000 | .000 | .000 | .000 |
| EOE\_4 | .047 | .053 | .002 | .424 | .744 | .000 | .000 | .000 | .000 | .000 | .000 | .000 | .000 |
| EOE\_3 | .046 | .053 | .002 | .422 | .742 | .000 | .000 | .000 | .000 | .000 | .000 | .000 | .000 |
| EOE\_2 | .047 | .053 | .002 | .426 | .749 | .000 | .000 | .000 | .000 | .000 | .000 | .000 | .000 |
| EOE\_1 | .046 | .052 | .002 | .421 | .740 | .000 | .000 | .000 | .000 | .000 | .000 | .000 | .000 |
| AISE\_6 | .000 | .000 | .000 | .773 | .000 | .000 | .000 | .000 | .000 | .000 | .000 | .000 | .000 |
| AISE\_5 | .000 | .000 | .000 | .780 | .000 | .000 | .000 | .000 | .000 | .000 | .000 | .000 | .000 |
| AISE\_4 | .000 | .000 | .000 | .768 | .000 | .000 | .000 | .000 | .000 | .000 | .000 | .000 | .000 |
| AISE\_3 | .000 | .000 | .000 | .755 | .000 | .000 | .000 | .000 | .000 | .000 | .000 | .000 | .000 |
| AISE\_2 | .000 | .000 | .000 | .776 | .000 | .000 | .000 | .000 | .000 | .000 | .000 | .000 | .000 |
| AISE\_1 | .000 | .000 | .000 | .743 | .000 | .000 | .000 | .000 | .000 | .000 | .000 | .000 | .000 |

## Standardized Total Effects - Two Tailed Significance (BC) (Group number 1 - Default model)

|  | Grade | Age | Gender | AISE | EOE | ESE | EE | Pa | Vc | Fs | Vp | Ss | Sm |
| --- | --- | --- | --- | --- | --- | --- | --- | --- | --- | --- | --- | --- | --- |
| EOE | .138 | .085 | .071 | .000 | ... | ... | ... | ... | ... | ... | ... | ... | ... |
| ESE | .251 | .645 | .009 | .000 | .000 | ... | ... | ... | ... | ... | ... | ... | ... |
| EE | .058 | .041 | .001 | .000 | .000 | .000 | ... | ... | ... | ... | ... | ... | ... |
| Pa | .058 | .043 | .001 | .000 | .000 | .000 | .000 | ... | ... | ... | ... | ... | ... |
| Vc | .057 | .042 | .001 | .000 | .000 | .000 | .000 | ... | ... | ... | ... | ... | ... |
| Fs | .058 | .040 | .001 | .000 | .000 | .000 | .000 | ... | ... | ... | ... | ... | ... |
| Vp | .059 | .041 | .001 | .000 | .000 | .000 | .000 | ... | ... | ... | ... | ... | ... |
| Ss | .249 | .646 | .009 | .000 | .000 | .000 | ... | ... | ... | ... | ... | ... | ... |
| Sm | .247 | .641 | .010 | .000 | .000 | .000 | ... | ... | ... | ... | ... | ... | ... |
| EE\_20 | .058 | .042 | .001 | .000 | .000 | .000 | .000 | .000 | ... | ... | ... | ... | ... |
| EE\_19 | .059 | .042 | .001 | .000 | .000 | .000 | .000 | .000 | ... | ... | ... | ... | ... |
| EE\_18 | .056 | .041 | .001 | .000 | .000 | .000 | .000 | .000 | ... | ... | ... | ... | ... |
| EE\_17 | .058 | .042 | .001 | .000 | .000 | .000 | .000 | ... | .000 | ... | ... | ... | ... |
| EE\_16 | .057 | .042 | .001 | .000 | .000 | .000 | .000 | ... | .000 | ... | ... | ... | ... |
| EE\_15 | .058 | .041 | .001 | .000 | .000 | .000 | .000 | ... | .000 | ... | ... | ... | ... |
| EE\_14 | .059 | .042 | .001 | .000 | .000 | .000 | .000 | ... | .001 | ... | ... | ... | ... |
| EE\_13 | .057 | .042 | .001 | .000 | .000 | .000 | .000 | ... | .001 | ... | ... | ... | ... |
| EE\_12 | .057 | .040 | .001 | .000 | .000 | .000 | .000 | ... | ... | .000 | ... | ... | ... |
| EE\_11 | .058 | .040 | .001 | .000 | .000 | .000 | .000 | ... | ... | .000 | ... | ... | ... |
| EE\_10 | .057 | .041 | .001 | .000 | .000 | .000 | .000 | ... | ... | .000 | ... | ... | ... |
| EE\_9 | .057 | .040 | .001 | .000 | .000 | .000 | .000 | ... | ... | .000 | ... | ... | ... |
| EE\_8 | .057 | .040 | .001 | .000 | .000 | .000 | .000 | ... | ... | .000 | ... | ... | ... |
| EE\_7 | .057 | .041 | .001 | .000 | .000 | .000 | .000 | ... | ... | .001 | ... | ... | ... |
| EE\_6 | .058 | .041 | .001 | .000 | .000 | .000 | .000 | ... | ... | ... | .000 | ... | ... |
| EE\_5 | .058 | .040 | .001 | .000 | .000 | .000 | .000 | ... | ... | ... | .000 | ... | ... |
| EE\_4 | .059 | .041 | .001 | .000 | .000 | .000 | .000 | ... | ... | ... | .000 | ... | ... |
| EE\_3 | .059 | .041 | .001 | .000 | .000 | .000 | .000 | ... | ... | ... | .000 | ... | ... |
| EE\_2 | .058 | .041 | .001 | .000 | .000 | .000 | .000 | ... | ... | ... | .000 | ... | ... |
| EE\_1 | .059 | .041 | .001 | .000 | .000 | .000 | .000 | ... | ... | ... | .000 | ... | ... |
| ESE\_10 | .246 | .642 | .009 | .000 | .000 | .000 | ... | ... | ... | ... | ... | .000 | ... |
| ESE\_9 | .249 | .647 | .009 | .000 | .000 | .000 | ... | ... | ... | ... | ... | .000 | ... |
| ESE\_8 | .246 | .645 | .009 | .000 | .000 | .000 | ... | ... | ... | ... | ... | .000 | ... |
| ESE\_7 | .251 | .650 | .009 | .000 | .000 | .000 | ... | ... | ... | ... | ... | .000 | ... |
| ESE\_6 | .248 | .643 | .009 | .000 | .000 | .000 | ... | ... | ... | ... | ... | ... | .000 |
| ESE\_5 | .245 | .643 | .009 | .000 | .000 | .000 | ... | ... | ... | ... | ... | ... | .000 |
| ESE\_4 | .249 | .647 | .010 | .000 | .000 | .000 | ... | ... | ... | ... | ... | ... | .001 |
| ESE\_3 | .248 | .645 | .009 | .000 | .000 | .000 | ... | ... | ... | ... | ... | ... | .000 |
| ESE\_2 | .249 | .646 | .009 | .000 | .000 | .000 | ... | ... | ... | ... | ... | ... | .001 |
| ESE\_1 | .246 | .643 | .009 | .000 | .000 | .001 | ... | ... | ... | ... | ... | ... | .001 |
| EOE\_9 | .136 | .085 | .070 | .000 | .000 | ... | ... | ... | ... | ... | ... | ... | ... |
| EOE\_8 | .138 | .085 | .070 | .001 | .000 | ... | ... | ... | ... | ... | ... | ... | ... |
| EOE\_7 | .139 | .085 | .070 | .000 | .000 | ... | ... | ... | ... | ... | ... | ... | ... |
| EOE\_6 | .139 | .084 | .071 | .000 | .000 | ... | ... | ... | ... | ... | ... | ... | ... |
| EOE\_5 | .138 | .086 | .070 | .000 | .000 | ... | ... | ... | ... | ... | ... | ... | ... |
| EOE\_4 | .137 | .085 | .070 | .000 | .000 | ... | ... | ... | ... | ... | ... | ... | ... |
| EOE\_3 | .135 | .084 | .070 | .000 | .000 | ... | ... | ... | ... | ... | ... | ... | ... |
| EOE\_2 | .138 | .086 | .072 | .000 | .000 | ... | ... | ... | ... | ... | ... | ... | ... |
| EOE\_1 | .137 | .084 | .071 | .001 | .000 | ... | ... | ... | ... | ... | ... | ... | ... |
| AISE\_6 | ... | ... | ... | .000 | ... | ... | ... | ... | ... | ... | ... | ... | ... |
| AISE\_5 | ... | ... | ... | .001 | ... | ... | ... | ... | ... | ... | ... | ... | ... |
| AISE\_4 | ... | ... | ... | .000 | ... | ... | ... | ... | ... | ... | ... | ... | ... |
| AISE\_3 | ... | ... | ... | .001 | ... | ... | ... | ... | ... | ... | ... | ... | ... |
| AISE\_2 | ... | ... | ... | .000 | ... | ... | ... | ... | ... | ... | ... | ... | ... |
| AISE\_1 | ... | ... | ... | .000 | ... | ... | ... | ... | ... | ... | ... | ... | ... |

## Direct Effects (Group number 1 - Default model)

## Direct Effects - Lower Bounds (BC) (Group number 1 - Default model)

|  | Grade | Age | Gender | AISE | EOE | ESE | EE | Pa | Vc | Fs | Vp | Ss | Sm |
| --- | --- | --- | --- | --- | --- | --- | --- | --- | --- | --- | --- | --- | --- |
| EOE | -.006 | -.002 | -.078 | .331 | .000 | .000 | .000 | .000 | .000 | .000 | .000 | .000 | .000 |
| ESE | -.013 | -.021 | -.085 | .208 | .207 | .000 | .000 | .000 | .000 | .000 | .000 | .000 | .000 |
| EE | -.007 | -.003 | -.084 | .107 | .131 | .160 | .000 | .000 | .000 | .000 | .000 | .000 | .000 |
| Pa | .000 | .000 | .000 | .000 | .000 | .000 | .895 | .000 | .000 | .000 | .000 | .000 | .000 |
| Vc | .000 | .000 | .000 | .000 | .000 | .000 | .979 | .000 | .000 | .000 | .000 | .000 | .000 |
| Fs | .000 | .000 | .000 | .000 | .000 | .000 | .953 | .000 | .000 | .000 | .000 | .000 | .000 |
| Vp | .000 | .000 | .000 | .000 | .000 | .000 | 1.000 | .000 | .000 | .000 | .000 | .000 | .000 |
| Ss | .000 | .000 | .000 | .000 | .000 | .902 | .000 | .000 | .000 | .000 | .000 | .000 | .000 |
| Sm | .000 | .000 | .000 | .000 | .000 | 1.000 | .000 | .000 | .000 | .000 | .000 | .000 | .000 |
| EE\_20 | .000 | .000 | .000 | .000 | .000 | .000 | .000 | .931 | .000 | .000 | .000 | .000 | .000 |
| EE\_19 | .000 | .000 | .000 | .000 | .000 | .000 | .000 | .916 | .000 | .000 | .000 | .000 | .000 |
| EE\_18 | .000 | .000 | .000 | .000 | .000 | .000 | .000 | 1.000 | .000 | .000 | .000 | .000 | .000 |
| EE\_17 | .000 | .000 | .000 | .000 | .000 | .000 | .000 | .000 | .914 | .000 | .000 | .000 | .000 |
| EE\_16 | .000 | .000 | .000 | .000 | .000 | .000 | .000 | .000 | .926 | .000 | .000 | .000 | .000 |
| EE\_15 | .000 | .000 | .000 | .000 | .000 | .000 | .000 | .000 | .937 | .000 | .000 | .000 | .000 |
| EE\_14 | .000 | .000 | .000 | .000 | .000 | .000 | .000 | .000 | .922 | .000 | .000 | .000 | .000 |
| EE\_13 | .000 | .000 | .000 | .000 | .000 | .000 | .000 | .000 | 1.000 | .000 | .000 | .000 | .000 |
| EE\_12 | .000 | .000 | .000 | .000 | .000 | .000 | .000 | .000 | .000 | .938 | .000 | .000 | .000 |
| EE\_11 | .000 | .000 | .000 | .000 | .000 | .000 | .000 | .000 | .000 | .945 | .000 | .000 | .000 |
| EE\_10 | .000 | .000 | .000 | .000 | .000 | .000 | .000 | .000 | .000 | .949 | .000 | .000 | .000 |
| EE\_9 | .000 | .000 | .000 | .000 | .000 | .000 | .000 | .000 | .000 | .952 | .000 | .000 | .000 |
| EE\_8 | .000 | .000 | .000 | .000 | .000 | .000 | .000 | .000 | .000 | .917 | .000 | .000 | .000 |
| EE\_7 | .000 | .000 | .000 | .000 | .000 | .000 | .000 | .000 | .000 | 1.000 | .000 | .000 | .000 |
| EE\_6 | .000 | .000 | .000 | .000 | .000 | .000 | .000 | .000 | .000 | .000 | .977 | .000 | .000 |
| EE\_5 | .000 | .000 | .000 | .000 | .000 | .000 | .000 | .000 | .000 | .000 | .966 | .000 | .000 |
| EE\_4 | .000 | .000 | .000 | .000 | .000 | .000 | .000 | .000 | .000 | .000 | .962 | .000 | .000 |
| EE\_3 | .000 | .000 | .000 | .000 | .000 | .000 | .000 | .000 | .000 | .000 | .995 | .000 | .000 |
| EE\_2 | .000 | .000 | .000 | .000 | .000 | .000 | .000 | .000 | .000 | .000 | .961 | .000 | .000 |
| EE\_1 | .000 | .000 | .000 | .000 | .000 | .000 | .000 | .000 | .000 | .000 | 1.000 | .000 | .000 |
| ESE\_10 | .000 | .000 | .000 | .000 | .000 | .000 | .000 | .000 | .000 | .000 | .000 | .926 | .000 |
| ESE\_9 | .000 | .000 | .000 | .000 | .000 | .000 | .000 | .000 | .000 | .000 | .000 | .893 | .000 |
| ESE\_8 | .000 | .000 | .000 | .000 | .000 | .000 | .000 | .000 | .000 | .000 | .000 | .952 | .000 |
| ESE\_7 | .000 | .000 | .000 | .000 | .000 | .000 | .000 | .000 | .000 | .000 | .000 | 1.000 | .000 |
| ESE\_6 | .000 | .000 | .000 | .000 | .000 | .000 | .000 | .000 | .000 | .000 | .000 | .000 | .970 |
| ESE\_5 | .000 | .000 | .000 | .000 | .000 | .000 | .000 | .000 | .000 | .000 | .000 | .000 | .973 |
| ESE\_4 | .000 | .000 | .000 | .000 | .000 | .000 | .000 | .000 | .000 | .000 | .000 | .000 | .986 |
| ESE\_3 | .000 | .000 | .000 | .000 | .000 | .000 | .000 | .000 | .000 | .000 | .000 | .000 | .924 |
| ESE\_2 | .000 | .000 | .000 | .000 | .000 | .000 | .000 | .000 | .000 | .000 | .000 | .000 | .969 |
| ESE\_1 | .000 | .000 | .000 | .000 | .000 | .000 | .000 | .000 | .000 | .000 | .000 | .000 | 1.000 |
| EOE\_9 | .000 | .000 | .000 | .000 | .943 | .000 | .000 | .000 | .000 | .000 | .000 | .000 | .000 |
| EOE\_8 | .000 | .000 | .000 | .000 | .969 | .000 | .000 | .000 | .000 | .000 | .000 | .000 | .000 |
| EOE\_7 | .000 | .000 | .000 | .000 | .973 | .000 | .000 | .000 | .000 | .000 | .000 | .000 | .000 |
| EOE\_6 | .000 | .000 | .000 | .000 | .938 | .000 | .000 | .000 | .000 | .000 | .000 | .000 | .000 |
| EOE\_5 | .000 | .000 | .000 | .000 | .974 | .000 | .000 | .000 | .000 | .000 | .000 | .000 | .000 |
| EOE\_4 | .000 | .000 | .000 | .000 | .938 | .000 | .000 | .000 | .000 | .000 | .000 | .000 | .000 |
| EOE\_3 | .000 | .000 | .000 | .000 | .947 | .000 | .000 | .000 | .000 | .000 | .000 | .000 | .000 |
| EOE\_2 | .000 | .000 | .000 | .000 | .961 | .000 | .000 | .000 | .000 | .000 | .000 | .000 | .000 |
| EOE\_1 | .000 | .000 | .000 | .000 | 1.000 | .000 | .000 | .000 | .000 | .000 | .000 | .000 | .000 |
| AISE\_6 | .000 | .000 | .000 | .948 | .000 | .000 | .000 | .000 | .000 | .000 | .000 | .000 | .000 |
| AISE\_5 | .000 | .000 | .000 | .905 | .000 | .000 | .000 | .000 | .000 | .000 | .000 | .000 | .000 |
| AISE\_4 | .000 | .000 | .000 | .939 | .000 | .000 | .000 | .000 | .000 | .000 | .000 | .000 | .000 |
| AISE\_3 | .000 | .000 | .000 | .909 | .000 | .000 | .000 | .000 | .000 | .000 | .000 | .000 | .000 |
| AISE\_2 | .000 | .000 | .000 | .932 | .000 | .000 | .000 | .000 | .000 | .000 | .000 | .000 | .000 |
| AISE\_1 | .000 | .000 | .000 | 1.000 | .000 | .000 | .000 | .000 | .000 | .000 | .000 | .000 | .000 |

## Direct Effects - Upper Bounds (BC) (Group number 1 - Default model)

|  | Grade | Age | Gender | AISE | EOE | ESE | EE | Pa | Vc | Fs | Vp | Ss | Sm |
| --- | --- | --- | --- | --- | --- | --- | --- | --- | --- | --- | --- | --- | --- |
| EOE | .039 | .043 | .003 | .395 | .000 | .000 | .000 | .000 | .000 | .000 | .000 | .000 | .000 |
| ESE | .031 | .020 | -.006 | .277 | .308 | .000 | .000 | .000 | .000 | .000 | .000 | .000 | .000 |
| EE | .034 | .035 | -.012 | .179 | .226 | .308 | .000 | .000 | .000 | .000 | .000 | .000 | .000 |
| Pa | .000 | .000 | .000 | .000 | .000 | .000 | 1.063 | .000 | .000 | .000 | .000 | .000 | .000 |
| Vc | .000 | .000 | .000 | .000 | .000 | .000 | 1.154 | .000 | .000 | .000 | .000 | .000 | .000 |
| Fs | .000 | .000 | .000 | .000 | .000 | .000 | 1.121 | .000 | .000 | .000 | .000 | .000 | .000 |
| Vp | .000 | .000 | .000 | .000 | .000 | .000 | 1.000 | .000 | .000 | .000 | .000 | .000 | .000 |
| Ss | .000 | .000 | .000 | .000 | .000 | 1.096 | .000 | .000 | .000 | .000 | .000 | .000 | .000 |
| Sm | .000 | .000 | .000 | .000 | .000 | 1.000 | .000 | .000 | .000 | .000 | .000 | .000 | .000 |
| EE\_20 | .000 | .000 | .000 | .000 | .000 | .000 | .000 | 1.054 | .000 | .000 | .000 | .000 | .000 |
| EE\_19 | .000 | .000 | .000 | .000 | .000 | .000 | .000 | 1.037 | .000 | .000 | .000 | .000 | .000 |
| EE\_18 | .000 | .000 | .000 | .000 | .000 | .000 | .000 | 1.000 | .000 | .000 | .000 | .000 | .000 |
| EE\_17 | .000 | .000 | .000 | .000 | .000 | .000 | .000 | .000 | 1.029 | .000 | .000 | .000 | .000 |
| EE\_16 | .000 | .000 | .000 | .000 | .000 | .000 | .000 | .000 | 1.034 | .000 | .000 | .000 | .000 |
| EE\_15 | .000 | .000 | .000 | .000 | .000 | .000 | .000 | .000 | 1.044 | .000 | .000 | .000 | .000 |
| EE\_14 | .000 | .000 | .000 | .000 | .000 | .000 | .000 | .000 | 1.029 | .000 | .000 | .000 | .000 |
| EE\_13 | .000 | .000 | .000 | .000 | .000 | .000 | .000 | .000 | 1.000 | .000 | .000 | .000 | .000 |
| EE\_12 | .000 | .000 | .000 | .000 | .000 | .000 | .000 | .000 | .000 | 1.058 | .000 | .000 | .000 |
| EE\_11 | .000 | .000 | .000 | .000 | .000 | .000 | .000 | .000 | .000 | 1.063 | .000 | .000 | .000 |
| EE\_10 | .000 | .000 | .000 | .000 | .000 | .000 | .000 | .000 | .000 | 1.068 | .000 | .000 | .000 |
| EE\_9 | .000 | .000 | .000 | .000 | .000 | .000 | .000 | .000 | .000 | 1.066 | .000 | .000 | .000 |
| EE\_8 | .000 | .000 | .000 | .000 | .000 | .000 | .000 | .000 | .000 | 1.035 | .000 | .000 | .000 |
| EE\_7 | .000 | .000 | .000 | .000 | .000 | .000 | .000 | .000 | .000 | 1.000 | .000 | .000 | .000 |
| EE\_6 | .000 | .000 | .000 | .000 | .000 | .000 | .000 | .000 | .000 | .000 | 1.101 | .000 | .000 |
| EE\_5 | .000 | .000 | .000 | .000 | .000 | .000 | .000 | .000 | .000 | .000 | 1.090 | .000 | .000 |
| EE\_4 | .000 | .000 | .000 | .000 | .000 | .000 | .000 | .000 | .000 | .000 | 1.086 | .000 | .000 |
| EE\_3 | .000 | .000 | .000 | .000 | .000 | .000 | .000 | .000 | .000 | .000 | 1.111 | .000 | .000 |
| EE\_2 | .000 | .000 | .000 | .000 | .000 | .000 | .000 | .000 | .000 | .000 | 1.083 | .000 | .000 |
| EE\_1 | .000 | .000 | .000 | .000 | .000 | .000 | .000 | .000 | .000 | .000 | 1.000 | .000 | .000 |
| ESE\_10 | .000 | .000 | .000 | .000 | .000 | .000 | .000 | .000 | .000 | .000 | .000 | 1.043 | .000 |
| ESE\_9 | .000 | .000 | .000 | .000 | .000 | .000 | .000 | .000 | .000 | .000 | .000 | 1.006 | .000 |
| ESE\_8 | .000 | .000 | .000 | .000 | .000 | .000 | .000 | .000 | .000 | .000 | .000 | 1.066 | .000 |
| ESE\_7 | .000 | .000 | .000 | .000 | .000 | .000 | .000 | .000 | .000 | .000 | .000 | 1.000 | .000 |
| ESE\_6 | .000 | .000 | .000 | .000 | .000 | .000 | .000 | .000 | .000 | .000 | .000 | .000 | 1.094 |
| ESE\_5 | .000 | .000 | .000 | .000 | .000 | .000 | .000 | .000 | .000 | .000 | .000 | .000 | 1.097 |
| ESE\_4 | .000 | .000 | .000 | .000 | .000 | .000 | .000 | .000 | .000 | .000 | .000 | .000 | 1.110 |
| ESE\_3 | .000 | .000 | .000 | .000 | .000 | .000 | .000 | .000 | .000 | .000 | .000 | .000 | 1.048 |
| ESE\_2 | .000 | .000 | .000 | .000 | .000 | .000 | .000 | .000 | .000 | .000 | .000 | .000 | 1.091 |
| ESE\_1 | .000 | .000 | .000 | .000 | .000 | .000 | .000 | .000 | .000 | .000 | .000 | .000 | 1.000 |
| EOE\_9 | .000 | .000 | .000 | .000 | 1.061 | .000 | .000 | .000 | .000 | .000 | .000 | .000 | .000 |
| EOE\_8 | .000 | .000 | .000 | .000 | 1.086 | .000 | .000 | .000 | .000 | .000 | .000 | .000 | .000 |
| EOE\_7 | .000 | .000 | .000 | .000 | 1.091 | .000 | .000 | .000 | .000 | .000 | .000 | .000 | .000 |
| EOE\_6 | .000 | .000 | .000 | .000 | 1.052 | .000 | .000 | .000 | .000 | .000 | .000 | .000 | .000 |
| EOE\_5 | .000 | .000 | .000 | .000 | 1.092 | .000 | .000 | .000 | .000 | .000 | .000 | .000 | .000 |
| EOE\_4 | .000 | .000 | .000 | .000 | 1.051 | .000 | .000 | .000 | .000 | .000 | .000 | .000 | .000 |
| EOE\_3 | .000 | .000 | .000 | .000 | 1.058 | .000 | .000 | .000 | .000 | .000 | .000 | .000 | .000 |
| EOE\_2 | .000 | .000 | .000 | .000 | 1.071 | .000 | .000 | .000 | .000 | .000 | .000 | .000 | .000 |
| EOE\_1 | .000 | .000 | .000 | .000 | 1.000 | .000 | .000 | .000 | .000 | .000 | .000 | .000 | .000 |
| AISE\_6 | .000 | .000 | .000 | 1.052 | .000 | .000 | .000 | .000 | .000 | .000 | .000 | .000 | .000 |
| AISE\_5 | .000 | .000 | .000 | 1.009 | .000 | .000 | .000 | .000 | .000 | .000 | .000 | .000 | .000 |
| AISE\_4 | .000 | .000 | .000 | 1.045 | .000 | .000 | .000 | .000 | .000 | .000 | .000 | .000 | .000 |
| AISE\_3 | .000 | .000 | .000 | 1.012 | .000 | .000 | .000 | .000 | .000 | .000 | .000 | .000 | .000 |
| AISE\_2 | .000 | .000 | .000 | 1.036 | .000 | .000 | .000 | .000 | .000 | .000 | .000 | .000 | .000 |
| AISE\_1 | .000 | .000 | .000 | 1.000 | .000 | .000 | .000 | .000 | .000 | .000 | .000 | .000 | .000 |

## Direct Effects - Two Tailed Significance (BC) (Group number 1 - Default model)

|  | Grade | Age | Gender | AISE | EOE | ESE | EE | Pa | Vc | Fs | Vp | Ss | Sm |
| --- | --- | --- | --- | --- | --- | --- | --- | --- | --- | --- | --- | --- | --- |
| EOE | .139 | .082 | .071 | .001 | ... | ... | ... | ... | ... | ... | ... | ... | ... |
| ESE | .393 | .983 | .026 | .000 | .000 | ... | ... | ... | ... | ... | ... | ... | ... |
| EE | .172 | .102 | .007 | .000 | .000 | .000 | ... | ... | ... | ... | ... | ... | ... |
| Pa | ... | ... | ... | ... | ... | ... | .001 | ... | ... | ... | ... | ... | ... |
| Vc | ... | ... | ... | ... | ... | ... | .000 | ... | ... | ... | ... | ... | ... |
| Fs | ... | ... | ... | ... | ... | ... | .000 | ... | ... | ... | ... | ... | ... |
| Vp | ... | ... | ... | ... | ... | ... | ... | ... | ... | ... | ... | ... | ... |
| Ss | ... | ... | ... | ... | ... | .000 | ... | ... | ... | ... | ... | ... | ... |
| Sm | ... | ... | ... | ... | ... | ... | ... | ... | ... | ... | ... | ... | ... |
| EE\_20 | ... | ... | ... | ... | ... | ... | ... | .000 | ... | ... | ... | ... | ... |
| EE\_19 | ... | ... | ... | ... | ... | ... | ... | .000 | ... | ... | ... | ... | ... |
| EE\_18 | ... | ... | ... | ... | ... | ... | ... | ... | ... | ... | ... | ... | ... |
| EE\_17 | ... | ... | ... | ... | ... | ... | ... | ... | .000 | ... | ... | ... | ... |
| EE\_16 | ... | ... | ... | ... | ... | ... | ... | ... | .000 | ... | ... | ... | ... |
| EE\_15 | ... | ... | ... | ... | ... | ... | ... | ... | .000 | ... | ... | ... | ... |
| EE\_14 | ... | ... | ... | ... | ... | ... | ... | ... | .000 | ... | ... | ... | ... |
| EE\_13 | ... | ... | ... | ... | ... | ... | ... | ... | ... | ... | ... | ... | ... |
| EE\_12 | ... | ... | ... | ... | ... | ... | ... | ... | ... | .000 | ... | ... | ... |
| EE\_11 | ... | ... | ... | ... | ... | ... | ... | ... | ... | .000 | ... | ... | ... |
| EE\_10 | ... | ... | ... | ... | ... | ... | ... | ... | ... | .000 | ... | ... | ... |
| EE\_9 | ... | ... | ... | ... | ... | ... | ... | ... | ... | .000 | ... | ... | ... |
| EE\_8 | ... | ... | ... | ... | ... | ... | ... | ... | ... | .000 | ... | ... | ... |
| EE\_7 | ... | ... | ... | ... | ... | ... | ... | ... | ... | ... | ... | ... | ... |
| EE\_6 | ... | ... | ... | ... | ... | ... | ... | ... | ... | ... | .000 | ... | ... |
| EE\_5 | ... | ... | ... | ... | ... | ... | ... | ... | ... | ... | .000 | ... | ... |
| EE\_4 | ... | ... | ... | ... | ... | ... | ... | ... | ... | ... | .000 | ... | ... |
| EE\_3 | ... | ... | ... | ... | ... | ... | ... | ... | ... | ... | .000 | ... | ... |
| EE\_2 | ... | ... | ... | ... | ... | ... | ... | ... | ... | ... | .000 | ... | ... |
| EE\_1 | ... | ... | ... | ... | ... | ... | ... | ... | ... | ... | ... | ... | ... |
| ESE\_10 | ... | ... | ... | ... | ... | ... | ... | ... | ... | ... | ... | .000 | ... |
| ESE\_9 | ... | ... | ... | ... | ... | ... | ... | ... | ... | ... | ... | .000 | ... |
| ESE\_8 | ... | ... | ... | ... | ... | ... | ... | ... | ... | ... | ... | .000 | ... |
| ESE\_7 | ... | ... | ... | ... | ... | ... | ... | ... | ... | ... | ... | ... | ... |
| ESE\_6 | ... | ... | ... | ... | ... | ... | ... | ... | ... | ... | ... | ... | .000 |
| ESE\_5 | ... | ... | ... | ... | ... | ... | ... | ... | ... | ... | ... | ... | .000 |
| ESE\_4 | ... | ... | ... | ... | ... | ... | ... | ... | ... | ... | ... | ... | .000 |
| ESE\_3 | ... | ... | ... | ... | ... | ... | ... | ... | ... | ... | ... | ... | .000 |
| ESE\_2 | ... | ... | ... | ... | ... | ... | ... | ... | ... | ... | ... | ... | .000 |
| ESE\_1 | ... | ... | ... | ... | ... | ... | ... | ... | ... | ... | ... | ... | ... |
| EOE\_9 | ... | ... | ... | ... | .000 | ... | ... | ... | ... | ... | ... | ... | ... |
| EOE\_8 | ... | ... | ... | ... | .000 | ... | ... | ... | ... | ... | ... | ... | ... |
| EOE\_7 | ... | ... | ... | ... | .000 | ... | ... | ... | ... | ... | ... | ... | ... |
| EOE\_6 | ... | ... | ... | ... | .000 | ... | ... | ... | ... | ... | ... | ... | ... |
| EOE\_5 | ... | ... | ... | ... | .000 | ... | ... | ... | ... | ... | ... | ... | ... |
| EOE\_4 | ... | ... | ... | ... | .000 | ... | ... | ... | ... | ... | ... | ... | ... |
| EOE\_3 | ... | ... | ... | ... | .000 | ... | ... | ... | ... | ... | ... | ... | ... |
| EOE\_2 | ... | ... | ... | ... | .000 | ... | ... | ... | ... | ... | ... | ... | ... |
| EOE\_1 | ... | ... | ... | ... | ... | ... | ... | ... | ... | ... | ... | ... | ... |
| AISE\_6 | ... | ... | ... | .000 | ... | ... | ... | ... | ... | ... | ... | ... | ... |
| AISE\_5 | ... | ... | ... | .000 | ... | ... | ... | ... | ... | ... | ... | ... | ... |
| AISE\_4 | ... | ... | ... | .000 | ... | ... | ... | ... | ... | ... | ... | ... | ... |
| AISE\_3 | ... | ... | ... | .000 | ... | ... | ... | ... | ... | ... | ... | ... | ... |
| AISE\_2 | ... | ... | ... | .000 | ... | ... | ... | ... | ... | ... | ... | ... | ... |
| AISE\_1 | ... | ... | ... | ... | ... | ... | ... | ... | ... | ... | ... | ... | ... |

## Standardized Direct Effects (Group number 1 - Default model)

## Standardized Direct Effects - Lower Bounds (BC) (Group number 1 - Default model)

|  | Grade | Age | Gender | AISE | EOE | ESE | EE | Pa | Vc | Fs | Vp | Ss | Sm |
| --- | --- | --- | --- | --- | --- | --- | --- | --- | --- | --- | --- | --- | --- |
| EOE | -.010 | -.004 | -.074 | .512 | .000 | .000 | .000 | .000 | .000 | .000 | .000 | .000 | .000 |
| ESE | -.024 | -.042 | -.094 | .374 | .247 | .000 | .000 | .000 | .000 | .000 | .000 | .000 | .000 |
| EE | -.013 | -.007 | -.092 | .188 | .155 | .163 | .000 | .000 | .000 | .000 | .000 | .000 | .000 |
| Pa | .000 | .000 | .000 | .000 | .000 | .000 | .774 | .000 | .000 | .000 | .000 | .000 | .000 |
| Vc | .000 | .000 | .000 | .000 | .000 | .000 | .801 | .000 | .000 | .000 | .000 | .000 | .000 |
| Fs | .000 | .000 | .000 | .000 | .000 | .000 | .789 | .000 | .000 | .000 | .000 | .000 | .000 |
| Vp | .000 | .000 | .000 | .000 | .000 | .000 | .782 | .000 | .000 | .000 | .000 | .000 | .000 |
| Ss | .000 | .000 | .000 | .000 | .000 | .748 | .000 | .000 | .000 | .000 | .000 | .000 | .000 |
| Sm | .000 | .000 | .000 | .000 | .000 | .777 | .000 | .000 | .000 | .000 | .000 | .000 | .000 |
| EE\_20 | .000 | .000 | .000 | .000 | .000 | .000 | .000 | .696 | .000 | .000 | .000 | .000 | .000 |
| EE\_19 | .000 | .000 | .000 | .000 | .000 | .000 | .000 | .688 | .000 | .000 | .000 | .000 | .000 |
| EE\_18 | .000 | .000 | .000 | .000 | .000 | .000 | .000 | .711 | .000 | .000 | .000 | .000 | .000 |
| EE\_17 | .000 | .000 | .000 | .000 | .000 | .000 | .000 | .000 | .714 | .000 | .000 | .000 | .000 |
| EE\_16 | .000 | .000 | .000 | .000 | .000 | .000 | .000 | .000 | .713 | .000 | .000 | .000 | .000 |
| EE\_15 | .000 | .000 | .000 | .000 | .000 | .000 | .000 | .000 | .726 | .000 | .000 | .000 | .000 |
| EE\_14 | .000 | .000 | .000 | .000 | .000 | .000 | .000 | .000 | .711 | .000 | .000 | .000 | .000 |
| EE\_13 | .000 | .000 | .000 | .000 | .000 | .000 | .000 | .000 | .727 | .000 | .000 | .000 | .000 |
| EE\_12 | .000 | .000 | .000 | .000 | .000 | .000 | .000 | .000 | .000 | .712 | .000 | .000 | .000 |
| EE\_11 | .000 | .000 | .000 | .000 | .000 | .000 | .000 | .000 | .000 | .712 | .000 | .000 | .000 |
| EE\_10 | .000 | .000 | .000 | .000 | .000 | .000 | .000 | .000 | .000 | .714 | .000 | .000 | .000 |
| EE\_9 | .000 | .000 | .000 | .000 | .000 | .000 | .000 | .000 | .000 | .717 | .000 | .000 | .000 |
| EE\_8 | .000 | .000 | .000 | .000 | .000 | .000 | .000 | .000 | .000 | .691 | .000 | .000 | .000 |
| EE\_7 | .000 | .000 | .000 | .000 | .000 | .000 | .000 | .000 | .000 | .706 | .000 | .000 | .000 |
| EE\_6 | .000 | .000 | .000 | .000 | .000 | .000 | .000 | .000 | .000 | .000 | .718 | .000 | .000 |
| EE\_5 | .000 | .000 | .000 | .000 | .000 | .000 | .000 | .000 | .000 | .000 | .703 | .000 | .000 |
| EE\_4 | .000 | .000 | .000 | .000 | .000 | .000 | .000 | .000 | .000 | .000 | .709 | .000 | .000 |
| EE\_3 | .000 | .000 | .000 | .000 | .000 | .000 | .000 | .000 | .000 | .000 | .722 | .000 | .000 |
| EE\_2 | .000 | .000 | .000 | .000 | .000 | .000 | .000 | .000 | .000 | .000 | .698 | .000 | .000 |
| EE\_1 | .000 | .000 | .000 | .000 | .000 | .000 | .000 | .000 | .000 | .000 | .694 | .000 | .000 |
| ESE\_10 | .000 | .000 | .000 | .000 | .000 | .000 | .000 | .000 | .000 | .000 | .000 | .708 | .000 |
| ESE\_9 | .000 | .000 | .000 | .000 | .000 | .000 | .000 | .000 | .000 | .000 | .000 | .692 | .000 |
| ESE\_8 | .000 | .000 | .000 | .000 | .000 | .000 | .000 | .000 | .000 | .000 | .000 | .724 | .000 |
| ESE\_7 | .000 | .000 | .000 | .000 | .000 | .000 | .000 | .000 | .000 | .000 | .000 | .722 | .000 |
| ESE\_6 | .000 | .000 | .000 | .000 | .000 | .000 | .000 | .000 | .000 | .000 | .000 | .000 | .697 |
| ESE\_5 | .000 | .000 | .000 | .000 | .000 | .000 | .000 | .000 | .000 | .000 | .000 | .000 | .717 |
| ESE\_4 | .000 | .000 | .000 | .000 | .000 | .000 | .000 | .000 | .000 | .000 | .000 | .000 | .716 |
| ESE\_3 | .000 | .000 | .000 | .000 | .000 | .000 | .000 | .000 | .000 | .000 | .000 | .000 | .688 |
| ESE\_2 | .000 | .000 | .000 | .000 | .000 | .000 | .000 | .000 | .000 | .000 | .000 | .000 | .708 |
| ESE\_1 | .000 | .000 | .000 | .000 | .000 | .000 | .000 | .000 | .000 | .000 | .000 | .000 | .687 |
| EOE\_9 | .000 | .000 | .000 | .000 | .697 | .000 | .000 | .000 | .000 | .000 | .000 | .000 | .000 |
| EOE\_8 | .000 | .000 | .000 | .000 | .707 | .000 | .000 | .000 | .000 | .000 | .000 | .000 | .000 |
| EOE\_7 | .000 | .000 | .000 | .000 | .725 | .000 | .000 | .000 | .000 | .000 | .000 | .000 | .000 |
| EOE\_6 | .000 | .000 | .000 | .000 | .693 | .000 | .000 | .000 | .000 | .000 | .000 | .000 | .000 |
| EOE\_5 | .000 | .000 | .000 | .000 | .717 | .000 | .000 | .000 | .000 | .000 | .000 | .000 | .000 |
| EOE\_4 | .000 | .000 | .000 | .000 | .702 | .000 | .000 | .000 | .000 | .000 | .000 | .000 | .000 |
| EOE\_3 | .000 | .000 | .000 | .000 | .698 | .000 | .000 | .000 | .000 | .000 | .000 | .000 | .000 |
| EOE\_2 | .000 | .000 | .000 | .000 | .704 | .000 | .000 | .000 | .000 | .000 | .000 | .000 | .000 |
| EOE\_1 | .000 | .000 | .000 | .000 | .697 | .000 | .000 | .000 | .000 | .000 | .000 | .000 | .000 |
| AISE\_6 | .000 | .000 | .000 | .731 | .000 | .000 | .000 | .000 | .000 | .000 | .000 | .000 | .000 |
| AISE\_5 | .000 | .000 | .000 | .739 | .000 | .000 | .000 | .000 | .000 | .000 | .000 | .000 | .000 |
| AISE\_4 | .000 | .000 | .000 | .726 | .000 | .000 | .000 | .000 | .000 | .000 | .000 | .000 | .000 |
| AISE\_3 | .000 | .000 | .000 | .713 | .000 | .000 | .000 | .000 | .000 | .000 | .000 | .000 | .000 |
| AISE\_2 | .000 | .000 | .000 | .736 | .000 | .000 | .000 | .000 | .000 | .000 | .000 | .000 | .000 |
| AISE\_1 | .000 | .000 | .000 | .698 | .000 | .000 | .000 | .000 | .000 | .000 | .000 | .000 | .000 |

## Standardized Direct Effects - Upper Bounds (BC) (Group number 1 - Default model)

|  | Grade | Age | Gender | AISE | EOE | ESE | EE | Pa | Vc | Fs | Vp | Ss | Sm |
| --- | --- | --- | --- | --- | --- | --- | --- | --- | --- | --- | --- | --- | --- |
| EOE | .064 | .073 | .003 | .580 | .000 | .000 | .000 | .000 | .000 | .000 | .000 | .000 | .000 |
| ESE | .060 | .041 | -.006 | .480 | .355 | .000 | .000 | .000 | .000 | .000 | .000 | .000 | .000 |
| EE | .066 | .070 | -.013 | .309 | .262 | .301 | .000 | .000 | .000 | .000 | .000 | .000 | .000 |
| Pa | .000 | .000 | .000 | .000 | .000 | .000 | .831 | .000 | .000 | .000 | .000 | .000 | .000 |
| Vc | .000 | .000 | .000 | .000 | .000 | .000 | .847 | .000 | .000 | .000 | .000 | .000 | .000 |
| Fs | .000 | .000 | .000 | .000 | .000 | .000 | .835 | .000 | .000 | .000 | .000 | .000 | .000 |
| Vp | .000 | .000 | .000 | .000 | .000 | .000 | .830 | .000 | .000 | .000 | .000 | .000 | .000 |
| Ss | .000 | .000 | .000 | .000 | .000 | .819 | .000 | .000 | .000 | .000 | .000 | .000 | .000 |
| Sm | .000 | .000 | .000 | .000 | .000 | .844 | .000 | .000 | .000 | .000 | .000 | .000 | .000 |
| EE\_20 | .000 | .000 | .000 | .000 | .000 | .000 | .000 | .748 | .000 | .000 | .000 | .000 | .000 |
| EE\_19 | .000 | .000 | .000 | .000 | .000 | .000 | .000 | .740 | .000 | .000 | .000 | .000 | .000 |
| EE\_18 | .000 | .000 | .000 | .000 | .000 | .000 | .000 | .762 | .000 | .000 | .000 | .000 | .000 |
| EE\_17 | .000 | .000 | .000 | .000 | .000 | .000 | .000 | .000 | .760 | .000 | .000 | .000 | .000 |
| EE\_16 | .000 | .000 | .000 | .000 | .000 | .000 | .000 | .000 | .756 | .000 | .000 | .000 | .000 |
| EE\_15 | .000 | .000 | .000 | .000 | .000 | .000 | .000 | .000 | .769 | .000 | .000 | .000 | .000 |
| EE\_14 | .000 | .000 | .000 | .000 | .000 | .000 | .000 | .000 | .755 | .000 | .000 | .000 | .000 |
| EE\_13 | .000 | .000 | .000 | .000 | .000 | .000 | .000 | .000 | .771 | .000 | .000 | .000 | .000 |
| EE\_12 | .000 | .000 | .000 | .000 | .000 | .000 | .000 | .000 | .000 | .756 | .000 | .000 | .000 |
| EE\_11 | .000 | .000 | .000 | .000 | .000 | .000 | .000 | .000 | .000 | .757 | .000 | .000 | .000 |
| EE\_10 | .000 | .000 | .000 | .000 | .000 | .000 | .000 | .000 | .000 | .757 | .000 | .000 | .000 |
| EE\_9 | .000 | .000 | .000 | .000 | .000 | .000 | .000 | .000 | .000 | .761 | .000 | .000 | .000 |
| EE\_8 | .000 | .000 | .000 | .000 | .000 | .000 | .000 | .000 | .000 | .737 | .000 | .000 | .000 |
| EE\_7 | .000 | .000 | .000 | .000 | .000 | .000 | .000 | .000 | .000 | .749 | .000 | .000 | .000 |
| EE\_6 | .000 | .000 | .000 | .000 | .000 | .000 | .000 | .000 | .000 | .000 | .761 | .000 | .000 |
| EE\_5 | .000 | .000 | .000 | .000 | .000 | .000 | .000 | .000 | .000 | .000 | .748 | .000 | .000 |
| EE\_4 | .000 | .000 | .000 | .000 | .000 | .000 | .000 | .000 | .000 | .000 | .752 | .000 | .000 |
| EE\_3 | .000 | .000 | .000 | .000 | .000 | .000 | .000 | .000 | .000 | .000 | .764 | .000 | .000 |
| EE\_2 | .000 | .000 | .000 | .000 | .000 | .000 | .000 | .000 | .000 | .000 | .744 | .000 | .000 |
| EE\_1 | .000 | .000 | .000 | .000 | .000 | .000 | .000 | .000 | .000 | .000 | .743 | .000 | .000 |
| ESE\_10 | .000 | .000 | .000 | .000 | .000 | .000 | .000 | .000 | .000 | .000 | .000 | .758 | .000 |
| ESE\_9 | .000 | .000 | .000 | .000 | .000 | .000 | .000 | .000 | .000 | .000 | .000 | .740 | .000 |
| ESE\_8 | .000 | .000 | .000 | .000 | .000 | .000 | .000 | .000 | .000 | .000 | .000 | .772 | .000 |
| ESE\_7 | .000 | .000 | .000 | .000 | .000 | .000 | .000 | .000 | .000 | .000 | .000 | .766 | .000 |
| ESE\_6 | .000 | .000 | .000 | .000 | .000 | .000 | .000 | .000 | .000 | .000 | .000 | .000 | .743 |
| ESE\_5 | .000 | .000 | .000 | .000 | .000 | .000 | .000 | .000 | .000 | .000 | .000 | .000 | .760 |
| ESE\_4 | .000 | .000 | .000 | .000 | .000 | .000 | .000 | .000 | .000 | .000 | .000 | .000 | .760 |
| ESE\_3 | .000 | .000 | .000 | .000 | .000 | .000 | .000 | .000 | .000 | .000 | .000 | .000 | .736 |
| ESE\_2 | .000 | .000 | .000 | .000 | .000 | .000 | .000 | .000 | .000 | .000 | .000 | .000 | .756 |
| ESE\_1 | .000 | .000 | .000 | .000 | .000 | .000 | .000 | .000 | .000 | .000 | .000 | .000 | .734 |
| EOE\_9 | .000 | .000 | .000 | .000 | .740 | .000 | .000 | .000 | .000 | .000 | .000 | .000 | .000 |
| EOE\_8 | .000 | .000 | .000 | .000 | .748 | .000 | .000 | .000 | .000 | .000 | .000 | .000 | .000 |
| EOE\_7 | .000 | .000 | .000 | .000 | .765 | .000 | .000 | .000 | .000 | .000 | .000 | .000 | .000 |
| EOE\_6 | .000 | .000 | .000 | .000 | .737 | .000 | .000 | .000 | .000 | .000 | .000 | .000 | .000 |
| EOE\_5 | .000 | .000 | .000 | .000 | .758 | .000 | .000 | .000 | .000 | .000 | .000 | .000 | .000 |
| EOE\_4 | .000 | .000 | .000 | .000 | .744 | .000 | .000 | .000 | .000 | .000 | .000 | .000 | .000 |
| EOE\_3 | .000 | .000 | .000 | .000 | .742 | .000 | .000 | .000 | .000 | .000 | .000 | .000 | .000 |
| EOE\_2 | .000 | .000 | .000 | .000 | .749 | .000 | .000 | .000 | .000 | .000 | .000 | .000 | .000 |
| EOE\_1 | .000 | .000 | .000 | .000 | .740 | .000 | .000 | .000 | .000 | .000 | .000 | .000 | .000 |
| AISE\_6 | .000 | .000 | .000 | .773 | .000 | .000 | .000 | .000 | .000 | .000 | .000 | .000 | .000 |
| AISE\_5 | .000 | .000 | .000 | .780 | .000 | .000 | .000 | .000 | .000 | .000 | .000 | .000 | .000 |
| AISE\_4 | .000 | .000 | .000 | .768 | .000 | .000 | .000 | .000 | .000 | .000 | .000 | .000 | .000 |
| AISE\_3 | .000 | .000 | .000 | .755 | .000 | .000 | .000 | .000 | .000 | .000 | .000 | .000 | .000 |
| AISE\_2 | .000 | .000 | .000 | .776 | .000 | .000 | .000 | .000 | .000 | .000 | .000 | .000 | .000 |
| AISE\_1 | .000 | .000 | .000 | .743 | .000 | .000 | .000 | .000 | .000 | .000 | .000 | .000 | .000 |

## Standardized Direct Effects - Two Tailed Significance (BC) (Group number 1 - Default model)

|  | Grade | Age | Gender | AISE | EOE | ESE | EE | Pa | Vc | Fs | Vp | Ss | Sm |
| --- | --- | --- | --- | --- | --- | --- | --- | --- | --- | --- | --- | --- | --- |
| EOE | .138 | .085 | .071 | .000 | ... | ... | ... | ... | ... | ... | ... | ... | ... |
| ESE | .400 | .983 | .025 | .000 | .000 | ... | ... | ... | ... | ... | ... | ... | ... |
| EE | .174 | .107 | .007 | .000 | .000 | .000 | ... | ... | ... | ... | ... | ... | ... |
| Pa | ... | ... | ... | ... | ... | ... | .000 | ... | ... | ... | ... | ... | ... |
| Vc | ... | ... | ... | ... | ... | ... | .000 | ... | ... | ... | ... | ... | ... |
| Fs | ... | ... | ... | ... | ... | ... | .000 | ... | ... | ... | ... | ... | ... |
| Vp | ... | ... | ... | ... | ... | ... | .000 | ... | ... | ... | ... | ... | ... |
| Ss | ... | ... | ... | ... | ... | .000 | ... | ... | ... | ... | ... | ... | ... |
| Sm | ... | ... | ... | ... | ... | .000 | ... | ... | ... | ... | ... | ... | ... |
| EE\_20 | ... | ... | ... | ... | ... | ... | ... | .000 | ... | ... | ... | ... | ... |
| EE\_19 | ... | ... | ... | ... | ... | ... | ... | .000 | ... | ... | ... | ... | ... |
| EE\_18 | ... | ... | ... | ... | ... | ... | ... | .000 | ... | ... | ... | ... | ... |
| EE\_17 | ... | ... | ... | ... | ... | ... | ... | ... | .000 | ... | ... | ... | ... |
| EE\_16 | ... | ... | ... | ... | ... | ... | ... | ... | .000 | ... | ... | ... | ... |
| EE\_15 | ... | ... | ... | ... | ... | ... | ... | ... | .000 | ... | ... | ... | ... |
| EE\_14 | ... | ... | ... | ... | ... | ... | ... | ... | .001 | ... | ... | ... | ... |
| EE\_13 | ... | ... | ... | ... | ... | ... | ... | ... | .001 | ... | ... | ... | ... |
| EE\_12 | ... | ... | ... | ... | ... | ... | ... | ... | ... | .000 | ... | ... | ... |
| EE\_11 | ... | ... | ... | ... | ... | ... | ... | ... | ... | .000 | ... | ... | ... |
| EE\_10 | ... | ... | ... | ... | ... | ... | ... | ... | ... | .000 | ... | ... | ... |
| EE\_9 | ... | ... | ... | ... | ... | ... | ... | ... | ... | .000 | ... | ... | ... |
| EE\_8 | ... | ... | ... | ... | ... | ... | ... | ... | ... | .000 | ... | ... | ... |
| EE\_7 | ... | ... | ... | ... | ... | ... | ... | ... | ... | .001 | ... | ... | ... |
| EE\_6 | ... | ... | ... | ... | ... | ... | ... | ... | ... | ... | .000 | ... | ... |
| EE\_5 | ... | ... | ... | ... | ... | ... | ... | ... | ... | ... | .000 | ... | ... |
| EE\_4 | ... | ... | ... | ... | ... | ... | ... | ... | ... | ... | .000 | ... | ... |
| EE\_3 | ... | ... | ... | ... | ... | ... | ... | ... | ... | ... | .000 | ... | ... |
| EE\_2 | ... | ... | ... | ... | ... | ... | ... | ... | ... | ... | .000 | ... | ... |
| EE\_1 | ... | ... | ... | ... | ... | ... | ... | ... | ... | ... | .000 | ... | ... |
| ESE\_10 | ... | ... | ... | ... | ... | ... | ... | ... | ... | ... | ... | .000 | ... |
| ESE\_9 | ... | ... | ... | ... | ... | ... | ... | ... | ... | ... | ... | .000 | ... |
| ESE\_8 | ... | ... | ... | ... | ... | ... | ... | ... | ... | ... | ... | .000 | ... |
| ESE\_7 | ... | ... | ... | ... | ... | ... | ... | ... | ... | ... | ... | .000 | ... |
| ESE\_6 | ... | ... | ... | ... | ... | ... | ... | ... | ... | ... | ... | ... | .000 |
| ESE\_5 | ... | ... | ... | ... | ... | ... | ... | ... | ... | ... | ... | ... | .000 |
| ESE\_4 | ... | ... | ... | ... | ... | ... | ... | ... | ... | ... | ... | ... | .001 |
| ESE\_3 | ... | ... | ... | ... | ... | ... | ... | ... | ... | ... | ... | ... | .000 |
| ESE\_2 | ... | ... | ... | ... | ... | ... | ... | ... | ... | ... | ... | ... | .001 |
| ESE\_1 | ... | ... | ... | ... | ... | ... | ... | ... | ... | ... | ... | ... | .001 |
| EOE\_9 | ... | ... | ... | ... | .000 | ... | ... | ... | ... | ... | ... | ... | ... |
| EOE\_8 | ... | ... | ... | ... | .000 | ... | ... | ... | ... | ... | ... | ... | ... |
| EOE\_7 | ... | ... | ... | ... | .000 | ... | ... | ... | ... | ... | ... | ... | ... |
| EOE\_6 | ... | ... | ... | ... | .000 | ... | ... | ... | ... | ... | ... | ... | ... |
| EOE\_5 | ... | ... | ... | ... | .000 | ... | ... | ... | ... | ... | ... | ... | ... |
| EOE\_4 | ... | ... | ... | ... | .000 | ... | ... | ... | ... | ... | ... | ... | ... |
| EOE\_3 | ... | ... | ... | ... | .000 | ... | ... | ... | ... | ... | ... | ... | ... |
| EOE\_2 | ... | ... | ... | ... | .000 | ... | ... | ... | ... | ... | ... | ... | ... |
| EOE\_1 | ... | ... | ... | ... | .000 | ... | ... | ... | ... | ... | ... | ... | ... |
| AISE\_6 | ... | ... | ... | .000 | ... | ... | ... | ... | ... | ... | ... | ... | ... |
| AISE\_5 | ... | ... | ... | .001 | ... | ... | ... | ... | ... | ... | ... | ... | ... |
| AISE\_4 | ... | ... | ... | .000 | ... | ... | ... | ... | ... | ... | ... | ... | ... |
| AISE\_3 | ... | ... | ... | .001 | ... | ... | ... | ... | ... | ... | ... | ... | ... |
| AISE\_2 | ... | ... | ... | .000 | ... | ... | ... | ... | ... | ... | ... | ... | ... |
| AISE\_1 | ... | ... | ... | .000 | ... | ... | ... | ... | ... | ... | ... | ... | ... |

## Indirect Effects (Group number 1 - Default model)

## Indirect Effects - Lower Bounds (BC) (Group number 1 - Default model)

|  | Grade | Age | Gender | AISE | EOE | ESE | EE | Pa | Vc | Fs | Vp | Ss | Sm |
| --- | --- | --- | --- | --- | --- | --- | --- | --- | --- | --- | --- | --- | --- |
| EOE | .000 | .000 | .000 | .000 | .000 | .000 | .000 | .000 | .000 | .000 | .000 | .000 | .000 |
| ESE | -.001 | .000 | -.021 | .074 | .000 | .000 | .000 | .000 | .000 | .000 | .000 | .000 | .000 |
| EE | -.001 | -.003 | -.035 | .116 | .041 | .000 | .000 | .000 | .000 | .000 | .000 | .000 | .000 |
| Pa | -.001 | .001 | -.102 | .248 | .186 | .158 | .000 | .000 | .000 | .000 | .000 | .000 | .000 |
| Vc | -.001 | .001 | -.112 | .271 | .204 | .171 | .000 | .000 | .000 | .000 | .000 | .000 | .000 |
| Fs | -.001 | .001 | -.108 | .263 | .199 | .167 | .000 | .000 | .000 | .000 | .000 | .000 | .000 |
| Vp | -.001 | .001 | -.104 | .253 | .191 | .160 | .000 | .000 | .000 | .000 | .000 | .000 | .000 |
| Ss | -.009 | -.016 | -.094 | .302 | .207 | .000 | .000 | .000 | .000 | .000 | .000 | .000 | .000 |
| Sm | -.009 | -.016 | -.097 | .302 | .207 | .000 | .000 | .000 | .000 | .000 | .000 | .000 | .000 |
| EE\_20 | -.001 | .001 | -.101 | .246 | .185 | .156 | .891 | .000 | .000 | .000 | .000 | .000 | .000 |
| EE\_19 | -.001 | .001 | -.100 | .242 | .181 | .153 | .877 | .000 | .000 | .000 | .000 | .000 | .000 |
| EE\_18 | -.001 | .001 | -.102 | .248 | .186 | .158 | .895 | .000 | .000 | .000 | .000 | .000 | .000 |
| EE\_17 | -.001 | .001 | -.108 | .263 | .198 | .166 | .950 | .000 | .000 | .000 | .000 | .000 | .000 |
| EE\_16 | -.001 | .001 | -.108 | .265 | .199 | .167 | .956 | .000 | .000 | .000 | .000 | .000 | .000 |
| EE\_15 | -.001 | .001 | -.109 | .268 | .201 | .169 | .969 | .000 | .000 | .000 | .000 | .000 | .000 |
| EE\_14 | -.001 | .001 | -.107 | .264 | .199 | .168 | .952 | .000 | .000 | .000 | .000 | .000 | .000 |
| EE\_13 | -.001 | .001 | -.112 | .271 | .204 | .171 | .979 | .000 | .000 | .000 | .000 | .000 | .000 |
| EE\_12 | -.001 | .001 | -.107 | .262 | .197 | .166 | .946 | .000 | .000 | .000 | .000 | .000 | .000 |
| EE\_11 | -.001 | .001 | -.108 | .263 | .199 | .169 | .953 | .000 | .000 | .000 | .000 | .000 | .000 |
| EE\_10 | -.001 | .001 | -.109 | .264 | .200 | .169 | .958 | .000 | .000 | .000 | .000 | .000 | .000 |
| EE\_9 | -.001 | .001 | -.109 | .265 | .199 | .168 | .959 | .000 | .000 | .000 | .000 | .000 | .000 |
| EE\_8 | -.001 | .001 | -.105 | .255 | .192 | .163 | .923 | .000 | .000 | .000 | .000 | .000 | .000 |
| EE\_7 | -.001 | .001 | -.108 | .263 | .199 | .167 | .953 | .000 | .000 | .000 | .000 | .000 | .000 |
| EE\_6 | -.001 | .001 | -.109 | .263 | .199 | .167 | .977 | .000 | .000 | .000 | .000 | .000 | .000 |
| EE\_5 | -.001 | .001 | -.107 | .261 | .197 | .165 | .966 | .000 | .000 | .000 | .000 | .000 | .000 |
| EE\_4 | -.001 | .001 | -.107 | .260 | .196 | .165 | .962 | .000 | .000 | .000 | .000 | .000 | .000 |
| EE\_3 | -.001 | .001 | -.109 | .267 | .201 | .169 | .995 | .000 | .000 | .000 | .000 | .000 | .000 |
| EE\_2 | -.001 | .001 | -.106 | .259 | .195 | .165 | .961 | .000 | .000 | .000 | .000 | .000 | .000 |
| EE\_1 | -.001 | .001 | -.104 | .253 | .191 | .160 | 1.000 | .000 | .000 | .000 | .000 | .000 | .000 |
| ESE\_10 | -.008 | -.016 | -.092 | .293 | .205 | .879 | .000 | .000 | .000 | .000 | .000 | .000 | .000 |
| ESE\_9 | -.008 | -.016 | -.089 | .284 | .196 | .853 | .000 | .000 | .000 | .000 | .000 | .000 | .000 |
| ESE\_8 | -.009 | -.017 | -.095 | .302 | .208 | .907 | .000 | .000 | .000 | .000 | .000 | .000 | .000 |
| ESE\_7 | -.009 | -.016 | -.094 | .302 | .207 | .902 | .000 | .000 | .000 | .000 | .000 | .000 | .000 |
| ESE\_6 | -.009 | -.017 | -.099 | .312 | .214 | .970 | .000 | .000 | .000 | .000 | .000 | .000 | .000 |
| ESE\_5 | -.009 | -.017 | -.100 | .312 | .214 | .973 | .000 | .000 | .000 | .000 | .000 | .000 | .000 |
| ESE\_4 | -.009 | -.017 | -.100 | .318 | .216 | .986 | .000 | .000 | .000 | .000 | .000 | .000 | .000 |
| ESE\_3 | -.009 | -.016 | -.094 | .297 | .204 | .924 | .000 | .000 | .000 | .000 | .000 | .000 | .000 |
| ESE\_2 | -.009 | -.017 | -.099 | .311 | .213 | .969 | .000 | .000 | .000 | .000 | .000 | .000 | .000 |
| ESE\_1 | -.009 | -.016 | -.097 | .302 | .207 | 1.000 | .000 | .000 | .000 | .000 | .000 | .000 | .000 |
| EOE\_9 | -.006 | -.002 | -.079 | .331 | .000 | .000 | .000 | .000 | .000 | .000 | .000 | .000 | .000 |
| EOE\_8 | -.006 | -.002 | -.080 | .339 | .000 | .000 | .000 | .000 | .000 | .000 | .000 | .000 | .000 |
| EOE\_7 | -.006 | -.002 | -.081 | .341 | .000 | .000 | .000 | .000 | .000 | .000 | .000 | .000 | .000 |
| EOE\_6 | -.006 | -.002 | -.078 | .329 | .000 | .000 | .000 | .000 | .000 | .000 | .000 | .000 | .000 |
| EOE\_5 | -.006 | -.002 | -.081 | .342 | .000 | .000 | .000 | .000 | .000 | .000 | .000 | .000 | .000 |
| EOE\_4 | -.006 | -.002 | -.078 | .328 | .000 | .000 | .000 | .000 | .000 | .000 | .000 | .000 | .000 |
| EOE\_3 | -.006 | -.002 | -.079 | .332 | .000 | .000 | .000 | .000 | .000 | .000 | .000 | .000 | .000 |
| EOE\_2 | -.006 | -.002 | -.080 | .336 | .000 | .000 | .000 | .000 | .000 | .000 | .000 | .000 | .000 |
| EOE\_1 | -.006 | -.002 | -.078 | .331 | .000 | .000 | .000 | .000 | .000 | .000 | .000 | .000 | .000 |
| AISE\_6 | .000 | .000 | .000 | .000 | .000 | .000 | .000 | .000 | .000 | .000 | .000 | .000 | .000 |
| AISE\_5 | .000 | .000 | .000 | .000 | .000 | .000 | .000 | .000 | .000 | .000 | .000 | .000 | .000 |
| AISE\_4 | .000 | .000 | .000 | .000 | .000 | .000 | .000 | .000 | .000 | .000 | .000 | .000 | .000 |
| AISE\_3 | .000 | .000 | .000 | .000 | .000 | .000 | .000 | .000 | .000 | .000 | .000 | .000 | .000 |
| AISE\_2 | .000 | .000 | .000 | .000 | .000 | .000 | .000 | .000 | .000 | .000 | .000 | .000 | .000 |
| AISE\_1 | .000 | .000 | .000 | .000 | .000 | .000 | .000 | .000 | .000 | .000 | .000 | .000 | .000 |

## Indirect Effects - Upper Bounds (BC) (Group number 1 - Default model)

|  | Grade | Age | Gender | AISE | EOE | ESE | EE | Pa | Vc | Fs | Vp | Ss | Sm |
| --- | --- | --- | --- | --- | --- | --- | --- | --- | --- | --- | --- | --- | --- |
| EOE | .000 | .000 | .000 | .000 | .000 | .000 | .000 | .000 | .000 | .000 | .000 | .000 | .000 |
| ESE | .010 | .011 | .000 | .113 | .000 | .000 | .000 | .000 | .000 | .000 | .000 | .000 | .000 |
| EE | .014 | .012 | -.006 | .169 | .083 | .000 | .000 | .000 | .000 | .000 | .000 | .000 | .000 |
| Pa | .041 | .040 | -.029 | .308 | .279 | .305 | .000 | .000 | .000 | .000 | .000 | .000 | .000 |
| Vc | .044 | .043 | -.032 | .334 | .301 | .329 | .000 | .000 | .000 | .000 | .000 | .000 | .000 |
| Fs | .043 | .042 | -.032 | .328 | .295 | .321 | .000 | .000 | .000 | .000 | .000 | .000 | .000 |
| Vp | .042 | .041 | -.030 | .316 | .283 | .308 | .000 | .000 | .000 | .000 | .000 | .000 | .000 |
| Ss | .036 | .027 | -.014 | .367 | .305 | .000 | .000 | .000 | .000 | .000 | .000 | .000 | .000 |
| Sm | .036 | .027 | -.013 | .368 | .308 | .000 | .000 | .000 | .000 | .000 | .000 | .000 | .000 |
| EE\_20 | .040 | .040 | -.029 | .305 | .276 | .301 | 1.051 | .000 | .000 | .000 | .000 | .000 | .000 |
| EE\_19 | .039 | .039 | -.029 | .300 | .272 | .296 | 1.031 | .000 | .000 | .000 | .000 | .000 | .000 |
| EE\_18 | .041 | .040 | -.029 | .308 | .279 | .305 | 1.063 | .000 | .000 | .000 | .000 | .000 | .000 |
| EE\_17 | .043 | .042 | -.031 | .324 | .294 | .320 | 1.123 | .000 | .000 | .000 | .000 | .000 | .000 |
| EE\_16 | .043 | .042 | -.031 | .327 | .296 | .323 | 1.127 | .000 | .000 | .000 | .000 | .000 | .000 |
| EE\_15 | .044 | .043 | -.032 | .331 | .298 | .325 | 1.140 | .000 | .000 | .000 | .000 | .000 | .000 |
| EE\_14 | .043 | .042 | -.031 | .327 | .293 | .319 | 1.118 | .000 | .000 | .000 | .000 | .000 | .000 |
| EE\_13 | .044 | .043 | -.032 | .334 | .301 | .329 | 1.154 | .000 | .000 | .000 | .000 | .000 | .000 |
| EE\_12 | .043 | .042 | -.031 | .325 | .293 | .319 | 1.118 | .000 | .000 | .000 | .000 | .000 | .000 |
| EE\_11 | .043 | .042 | -.031 | .328 | .294 | .324 | 1.123 | .000 | .000 | .000 | .000 | .000 | .000 |
| EE\_10 | .044 | .042 | -.032 | .328 | .295 | .324 | 1.134 | .000 | .000 | .000 | .000 | .000 | .000 |
| EE\_9 | .043 | .042 | -.032 | .328 | .297 | .326 | 1.133 | .000 | .000 | .000 | .000 | .000 | .000 |
| EE\_8 | .041 | .041 | -.030 | .318 | .286 | .312 | 1.096 | .000 | .000 | .000 | .000 | .000 | .000 |
| EE\_7 | .043 | .042 | -.032 | .328 | .295 | .321 | 1.121 | .000 | .000 | .000 | .000 | .000 | .000 |
| EE\_6 | .043 | .043 | -.032 | .326 | .294 | .322 | 1.101 | .000 | .000 | .000 | .000 | .000 | .000 |
| EE\_5 | .043 | .042 | -.031 | .323 | .291 | .318 | 1.090 | .000 | .000 | .000 | .000 | .000 | .000 |
| EE\_4 | .043 | .042 | -.031 | .322 | .289 | .317 | 1.086 | .000 | .000 | .000 | .000 | .000 | .000 |
| EE\_3 | .044 | .043 | -.032 | .330 | .297 | .325 | 1.111 | .000 | .000 | .000 | .000 | .000 | .000 |
| EE\_2 | .043 | .042 | -.031 | .322 | .291 | .317 | 1.083 | .000 | .000 | .000 | .000 | .000 | .000 |
| EE\_1 | .042 | .041 | -.030 | .316 | .283 | .308 | 1.000 | .000 | .000 | .000 | .000 | .000 | .000 |
| ESE\_10 | .035 | .026 | -.013 | .362 | .300 | 1.079 | .000 | .000 | .000 | .000 | .000 | .000 | .000 |
| ESE\_9 | .034 | .025 | -.013 | .348 | .290 | 1.042 | .000 | .000 | .000 | .000 | .000 | .000 | .000 |
| ESE\_8 | .036 | .027 | -.014 | .371 | .306 | 1.107 | .000 | .000 | .000 | .000 | .000 | .000 | .000 |
| ESE\_7 | .036 | .027 | -.014 | .367 | .305 | 1.096 | .000 | .000 | .000 | .000 | .000 | .000 | .000 |
| ESE\_6 | .037 | .028 | -.014 | .380 | .316 | 1.094 | .000 | .000 | .000 | .000 | .000 | .000 | .000 |
| ESE\_5 | .037 | .028 | -.014 | .381 | .318 | 1.097 | .000 | .000 | .000 | .000 | .000 | .000 | .000 |
| ESE\_4 | .038 | .028 | -.014 | .385 | .323 | 1.110 | .000 | .000 | .000 | .000 | .000 | .000 | .000 |
| ESE\_3 | .035 | .026 | -.013 | .363 | .302 | 1.048 | .000 | .000 | .000 | .000 | .000 | .000 | .000 |
| ESE\_2 | .037 | .028 | -.014 | .379 | .316 | 1.091 | .000 | .000 | .000 | .000 | .000 | .000 | .000 |
| ESE\_1 | .036 | .027 | -.013 | .368 | .308 | 1.000 | .000 | .000 | .000 | .000 | .000 | .000 | .000 |
| EOE\_9 | .039 | .042 | .003 | .398 | .000 | .000 | .000 | .000 | .000 | .000 | .000 | .000 | .000 |
| EOE\_8 | .040 | .044 | .003 | .407 | .000 | .000 | .000 | .000 | .000 | .000 | .000 | .000 | .000 |
| EOE\_7 | .041 | .044 | .003 | .408 | .000 | .000 | .000 | .000 | .000 | .000 | .000 | .000 | .000 |
| EOE\_6 | .039 | .042 | .003 | .393 | .000 | .000 | .000 | .000 | .000 | .000 | .000 | .000 | .000 |
| EOE\_5 | .040 | .044 | .003 | .408 | .000 | .000 | .000 | .000 | .000 | .000 | .000 | .000 | .000 |
| EOE\_4 | .039 | .042 | .003 | .395 | .000 | .000 | .000 | .000 | .000 | .000 | .000 | .000 | .000 |
| EOE\_3 | .039 | .043 | .003 | .397 | .000 | .000 | .000 | .000 | .000 | .000 | .000 | .000 | .000 |
| EOE\_2 | .040 | .043 | .003 | .402 | .000 | .000 | .000 | .000 | .000 | .000 | .000 | .000 | .000 |
| EOE\_1 | .039 | .043 | .003 | .395 | .000 | .000 | .000 | .000 | .000 | .000 | .000 | .000 | .000 |
| AISE\_6 | .000 | .000 | .000 | .000 | .000 | .000 | .000 | .000 | .000 | .000 | .000 | .000 | .000 |
| AISE\_5 | .000 | .000 | .000 | .000 | .000 | .000 | .000 | .000 | .000 | .000 | .000 | .000 | .000 |
| AISE\_4 | .000 | .000 | .000 | .000 | .000 | .000 | .000 | .000 | .000 | .000 | .000 | .000 | .000 |
| AISE\_3 | .000 | .000 | .000 | .000 | .000 | .000 | .000 | .000 | .000 | .000 | .000 | .000 | .000 |
| AISE\_2 | .000 | .000 | .000 | .000 | .000 | .000 | .000 | .000 | .000 | .000 | .000 | .000 | .000 |
| AISE\_1 | .000 | .000 | .000 | .000 | .000 | .000 | .000 | .000 | .000 | .000 | .000 | .000 | .000 |

## Indirect Effects - Two Tailed Significance (BC) (Group number 1 - Default model)

|  | Grade | Age | Gender | AISE | EOE | ESE | EE | Pa | Vc | Fs | Vp | Ss | Sm |
| --- | --- | --- | --- | --- | --- | --- | --- | --- | --- | --- | --- | --- | --- |
| EOE | ... | ... | ... | ... | ... | ... | ... | ... | ... | ... | ... | ... | ... |
| ESE | .129 | .077 | .064 | .000 | ... | ... | ... | ... | ... | ... | ... | ... | ... |
| EE | .096 | .200 | .003 | .000 | .000 | ... | ... | ... | ... | ... | ... | ... | ... |
| Pa | .056 | .042 | .001 | .000 | .000 | .000 | ... | ... | ... | ... | ... | ... | ... |
| Vc | .057 | .042 | .001 | .000 | .000 | .000 | ... | ... | ... | ... | ... | ... | ... |
| Fs | .058 | .042 | .001 | .000 | .000 | .000 | ... | ... | ... | ... | ... | ... | ... |
| Vp | .059 | .040 | .001 | .000 | .000 | .000 | ... | ... | ... | ... | ... | ... | ... |
| Ss | .249 | .652 | .009 | .000 | .000 | ... | ... | ... | ... | ... | ... | ... | ... |
| Sm | .245 | .645 | .009 | .000 | .000 | ... | ... | ... | ... | ... | ... | ... | ... |
| EE\_20 | .057 | .042 | .001 | .000 | .000 | .000 | .000 | ... | ... | ... | ... | ... | ... |
| EE\_19 | .058 | .043 | .001 | .000 | .000 | .000 | .000 | ... | ... | ... | ... | ... | ... |
| EE\_18 | .056 | .042 | .001 | .000 | .000 | .000 | .001 | ... | ... | ... | ... | ... | ... |
| EE\_17 | .058 | .041 | .001 | .000 | .000 | .000 | .000 | ... | ... | ... | ... | ... | ... |
| EE\_16 | .058 | .042 | .001 | .000 | .000 | .000 | .000 | ... | ... | ... | ... | ... | ... |
| EE\_15 | .057 | .041 | .001 | .000 | .000 | .000 | .000 | ... | ... | ... | ... | ... | ... |
| EE\_14 | .059 | .042 | .001 | .000 | .000 | .000 | .000 | ... | ... | ... | ... | ... | ... |
| EE\_13 | .057 | .042 | .001 | .000 | .000 | .000 | .000 | ... | ... | ... | ... | ... | ... |
| EE\_12 | .058 | .041 | .001 | .000 | .000 | .000 | .000 | ... | ... | ... | ... | ... | ... |
| EE\_11 | .056 | .041 | .001 | .000 | .000 | .000 | .001 | ... | ... | ... | ... | ... | ... |
| EE\_10 | .056 | .043 | .001 | .000 | .000 | .000 | .000 | ... | ... | ... | ... | ... | ... |
| EE\_9 | .058 | .042 | .001 | .000 | .000 | .000 | .000 | ... | ... | ... | ... | ... | ... |
| EE\_8 | .057 | .042 | .001 | .000 | .000 | .000 | .000 | ... | ... | ... | ... | ... | ... |
| EE\_7 | .058 | .042 | .001 | .000 | .000 | .000 | .000 | ... | ... | ... | ... | ... | ... |
| EE\_6 | .058 | .041 | .001 | .000 | .000 | .000 | .000 | ... | ... | ... | ... | ... | ... |
| EE\_5 | .058 | .039 | .001 | .000 | .000 | .000 | .000 | ... | ... | ... | ... | ... | ... |
| EE\_4 | .058 | .041 | .001 | .000 | .000 | .000 | .000 | ... | ... | ... | ... | ... | ... |
| EE\_3 | .059 | .042 | .001 | .000 | .000 | .000 | .000 | ... | ... | ... | ... | ... | ... |
| EE\_2 | .058 | .042 | .001 | .000 | .000 | .000 | .000 | ... | ... | ... | ... | ... | ... |
| EE\_1 | .059 | .040 | .001 | .000 | .000 | .000 | ... | ... | ... | ... | ... | ... | ... |
| ESE\_10 | .246 | .646 | .009 | .000 | .000 | .000 | ... | ... | ... | ... | ... | ... | ... |
| ESE\_9 | .245 | .649 | .009 | .000 | .000 | .000 | ... | ... | ... | ... | ... | ... | ... |
| ESE\_8 | .245 | .648 | .009 | .000 | .000 | .000 | ... | ... | ... | ... | ... | ... | ... |
| ESE\_7 | .249 | .652 | .009 | .000 | .000 | .000 | ... | ... | ... | ... | ... | ... | ... |
| ESE\_6 | .249 | .644 | .009 | .000 | .000 | .000 | ... | ... | ... | ... | ... | ... | ... |
| ESE\_5 | .246 | .643 | .009 | .000 | .000 | .000 | ... | ... | ... | ... | ... | ... | ... |
| ESE\_4 | .249 | .644 | .010 | .000 | .000 | .000 | ... | ... | ... | ... | ... | ... | ... |
| ESE\_3 | .247 | .650 | .009 | .000 | .000 | .000 | ... | ... | ... | ... | ... | ... | ... |
| ESE\_2 | .251 | .649 | .009 | .000 | .000 | .000 | ... | ... | ... | ... | ... | ... | ... |
| ESE\_1 | .245 | .645 | .009 | .000 | .000 | ... | ... | ... | ... | ... | ... | ... | ... |
| EOE\_9 | .138 | .085 | .069 | .000 | ... | ... | ... | ... | ... | ... | ... | ... | ... |
| EOE\_8 | .138 | .084 | .070 | .000 | ... | ... | ... | ... | ... | ... | ... | ... | ... |
| EOE\_7 | .139 | .086 | .071 | .000 | ... | ... | ... | ... | ... | ... | ... | ... | ... |
| EOE\_6 | .141 | .082 | .072 | .000 | ... | ... | ... | ... | ... | ... | ... | ... | ... |
| EOE\_5 | .136 | .083 | .071 | .000 | ... | ... | ... | ... | ... | ... | ... | ... | ... |
| EOE\_4 | .139 | .084 | .071 | .000 | ... | ... | ... | ... | ... | ... | ... | ... | ... |
| EOE\_3 | .136 | .083 | .070 | .000 | ... | ... | ... | ... | ... | ... | ... | ... | ... |
| EOE\_2 | .137 | .085 | .071 | .000 | ... | ... | ... | ... | ... | ... | ... | ... | ... |
| EOE\_1 | .139 | .082 | .071 | .001 | ... | ... | ... | ... | ... | ... | ... | ... | ... |
| AISE\_6 | ... | ... | ... | ... | ... | ... | ... | ... | ... | ... | ... | ... | ... |
| AISE\_5 | ... | ... | ... | ... | ... | ... | ... | ... | ... | ... | ... | ... | ... |
| AISE\_4 | ... | ... | ... | ... | ... | ... | ... | ... | ... | ... | ... | ... | ... |
| AISE\_3 | ... | ... | ... | ... | ... | ... | ... | ... | ... | ... | ... | ... | ... |
| AISE\_2 | ... | ... | ... | ... | ... | ... | ... | ... | ... | ... | ... | ... | ... |
| AISE\_1 | ... | ... | ... | ... | ... | ... | ... | ... | ... | ... | ... | ... | ... |

## Standardized Indirect Effects (Group number 1 - Default model)

## Standardized Indirect Effects - Lower Bounds (BC) (Group number 1 - Default model)

|  | Grade | Age | Gender | AISE | EOE | ESE | EE | Pa | Vc | Fs | Vp | Ss | Sm |
| --- | --- | --- | --- | --- | --- | --- | --- | --- | --- | --- | --- | --- | --- |
| EOE | .000 | .000 | .000 | .000 | .000 | .000 | .000 | .000 | .000 | .000 | .000 | .000 | .000 |
| ESE | -.003 | -.001 | -.023 | .135 | .000 | .000 | .000 | .000 | .000 | .000 | .000 | .000 | .000 |
| EE | -.002 | -.005 | -.038 | .209 | .048 | .000 | .000 | .000 | .000 | .000 | .000 | .000 | .000 |
| Pa | -.001 | .001 | -.091 | .366 | .181 | .131 | .000 | .000 | .000 | .000 | .000 | .000 | .000 |
| Vc | -.001 | .001 | -.094 | .378 | .186 | .133 | .000 | .000 | .000 | .000 | .000 | .000 | .000 |
| Fs | -.001 | .002 | -.092 | .373 | .184 | .133 | .000 | .000 | .000 | .000 | .000 | .000 | .000 |
| Vp | -.001 | .001 | -.091 | .370 | .182 | .131 | .000 | .000 | .000 | .000 | .000 | .000 | .000 |
| Ss | -.013 | -.026 | -.081 | .428 | .194 | .000 | .000 | .000 | .000 | .000 | .000 | .000 | .000 |
| Sm | -.014 | -.027 | -.085 | .444 | .199 | .000 | .000 | .000 | .000 | .000 | .000 | .000 | .000 |
| EE\_20 | -.001 | .001 | -.066 | .263 | .130 | .095 | .552 | .000 | .000 | .000 | .000 | .000 | .000 |
| EE\_19 | -.001 | .001 | -.066 | .259 | .129 | .093 | .544 | .000 | .000 | .000 | .000 | .000 | .000 |
| EE\_18 | -.001 | .001 | -.068 | .268 | .133 | .097 | .563 | .000 | .000 | .000 | .000 | .000 | .000 |
| EE\_17 | -.001 | .001 | -.069 | .277 | .137 | .099 | .581 | .000 | .000 | .000 | .000 | .000 | .000 |
| EE\_16 | -.001 | .001 | -.069 | .277 | .136 | .098 | .578 | .000 | .000 | .000 | .000 | .000 | .000 |
| EE\_15 | -.001 | .001 | -.070 | .281 | .139 | .100 | .590 | .000 | .000 | .000 | .000 | .000 | .000 |
| EE\_14 | -.001 | .001 | -.068 | .275 | .136 | .098 | .578 | .000 | .000 | .000 | .000 | .000 | .000 |
| EE\_13 | -.001 | .001 | -.071 | .281 | .139 | .101 | .591 | .000 | .000 | .000 | .000 | .000 | .000 |
| EE\_12 | -.001 | .001 | -.068 | .271 | .135 | .098 | .569 | .000 | .000 | .000 | .000 | .000 | .000 |
| EE\_11 | -.001 | .001 | -.068 | .271 | .135 | .098 | .571 | .000 | .000 | .000 | .000 | .000 | .000 |
| EE\_10 | -.001 | .001 | -.069 | .272 | .135 | .098 | .572 | .000 | .000 | .000 | .000 | .000 | .000 |
| EE\_9 | -.001 | .001 | -.069 | .273 | .135 | .098 | .575 | .000 | .000 | .000 | .000 | .000 | .000 |
| EE\_8 | -.001 | .001 | -.066 | .263 | .130 | .095 | .554 | .000 | .000 | .000 | .000 | .000 | .000 |
| EE\_7 | -.001 | .001 | -.067 | .269 | .133 | .097 | .567 | .000 | .000 | .000 | .000 | .000 | .000 |
| EE\_6 | -.001 | .001 | -.068 | .271 | .134 | .097 | .570 | .000 | .000 | .000 | .000 | .000 | .000 |
| EE\_5 | -.001 | .001 | -.066 | .266 | .132 | .095 | .558 | .000 | .000 | .000 | .000 | .000 | .000 |
| EE\_4 | -.001 | .001 | -.067 | .268 | .133 | .096 | .565 | .000 | .000 | .000 | .000 | .000 | .000 |
| EE\_3 | -.001 | .001 | -.068 | .273 | .135 | .098 | .574 | .000 | .000 | .000 | .000 | .000 | .000 |
| EE\_2 | -.001 | .001 | -.066 | .265 | .131 | .095 | .554 | .000 | .000 | .000 | .000 | .000 | .000 |
| EE\_1 | -.001 | .001 | -.066 | .263 | .130 | .095 | .551 | .000 | .000 | .000 | .000 | .000 | .000 |
| ESE\_10 | -.010 | -.019 | -.060 | .310 | .142 | .542 | .000 | .000 | .000 | .000 | .000 | .000 | .000 |
| ESE\_9 | -.009 | -.019 | -.058 | .305 | .139 | .531 | .000 | .000 | .000 | .000 | .000 | .000 | .000 |
| ESE\_8 | -.010 | -.020 | -.061 | .318 | .144 | .554 | .000 | .000 | .000 | .000 | .000 | .000 | .000 |
| ESE\_7 | -.010 | -.020 | -.061 | .317 | .145 | .552 | .000 | .000 | .000 | .000 | .000 | .000 | .000 |
| ESE\_6 | -.010 | -.019 | -.062 | .318 | .143 | .551 | .000 | .000 | .000 | .000 | .000 | .000 | .000 |
| ESE\_5 | -.010 | -.020 | -.064 | .325 | .146 | .566 | .000 | .000 | .000 | .000 | .000 | .000 | .000 |
| ESE\_4 | -.010 | -.020 | -.063 | .326 | .147 | .568 | .000 | .000 | .000 | .000 | .000 | .000 | .000 |
| ESE\_3 | -.010 | -.019 | -.061 | .314 | .142 | .545 | .000 | .000 | .000 | .000 | .000 | .000 | .000 |
| ESE\_2 | -.010 | -.020 | -.063 | .322 | .146 | .562 | .000 | .000 | .000 | .000 | .000 | .000 | .000 |
| ESE\_1 | -.010 | -.019 | -.061 | .312 | .141 | .544 | .000 | .000 | .000 | .000 | .000 | .000 | .000 |
| EOE\_9 | -.007 | -.003 | -.053 | .363 | .000 | .000 | .000 | .000 | .000 | .000 | .000 | .000 | .000 |
| EOE\_8 | -.007 | -.003 | -.054 | .369 | .000 | .000 | .000 | .000 | .000 | .000 | .000 | .000 | .000 |
| EOE\_7 | -.007 | -.003 | -.055 | .379 | .000 | .000 | .000 | .000 | .000 | .000 | .000 | .000 | .000 |
| EOE\_6 | -.007 | -.002 | -.053 | .364 | .000 | .000 | .000 | .000 | .000 | .000 | .000 | .000 | .000 |
| EOE\_5 | -.007 | -.003 | -.054 | .375 | .000 | .000 | .000 | .000 | .000 | .000 | .000 | .000 | .000 |
| EOE\_4 | -.007 | -.003 | -.053 | .366 | .000 | .000 | .000 | .000 | .000 | .000 | .000 | .000 | .000 |
| EOE\_3 | -.007 | -.002 | -.053 | .365 | .000 | .000 | .000 | .000 | .000 | .000 | .000 | .000 | .000 |
| EOE\_2 | -.007 | -.003 | -.053 | .369 | .000 | .000 | .000 | .000 | .000 | .000 | .000 | .000 | .000 |
| EOE\_1 | -.007 | -.003 | -.053 | .364 | .000 | .000 | .000 | .000 | .000 | .000 | .000 | .000 | .000 |
| AISE\_6 | .000 | .000 | .000 | .000 | .000 | .000 | .000 | .000 | .000 | .000 | .000 | .000 | .000 |
| AISE\_5 | .000 | .000 | .000 | .000 | .000 | .000 | .000 | .000 | .000 | .000 | .000 | .000 | .000 |
| AISE\_4 | .000 | .000 | .000 | .000 | .000 | .000 | .000 | .000 | .000 | .000 | .000 | .000 | .000 |
| AISE\_3 | .000 | .000 | .000 | .000 | .000 | .000 | .000 | .000 | .000 | .000 | .000 | .000 | .000 |
| AISE\_2 | .000 | .000 | .000 | .000 | .000 | .000 | .000 | .000 | .000 | .000 | .000 | .000 | .000 |
| AISE\_1 | .000 | .000 | .000 | .000 | .000 | .000 | .000 | .000 | .000 | .000 | .000 | .000 | .000 |

## Standardized Indirect Effects - Upper Bounds (BC) (Group number 1 - Default model)

|  | Grade | Age | Gender | AISE | EOE | ESE | EE | Pa | Vc | Fs | Vp | Ss | Sm |
| --- | --- | --- | --- | --- | --- | --- | --- | --- | --- | --- | --- | --- | --- |
| EOE | .000 | .000 | .000 | .000 | .000 | .000 | .000 | .000 | .000 | .000 | .000 | .000 | .000 |
| ESE | .020 | .023 | .001 | .196 | .000 | .000 | .000 | .000 | .000 | .000 | .000 | .000 | .000 |
| EE | .026 | .024 | -.007 | .292 | .096 | .000 | .000 | .000 | .000 | .000 | .000 | .000 | .000 |
| Pa | .064 | .066 | -.026 | .435 | .264 | .243 | .000 | .000 | .000 | .000 | .000 | .000 | .000 |
| Vc | .066 | .067 | -.027 | .445 | .270 | .247 | .000 | .000 | .000 | .000 | .000 | .000 | .000 |
| Fs | .065 | .067 | -.027 | .441 | .267 | .246 | .000 | .000 | .000 | .000 | .000 | .000 | .000 |
| Vp | .064 | .066 | -.027 | .436 | .264 | .243 | .000 | .000 | .000 | .000 | .000 | .000 | .000 |
| Ss | .054 | .043 | -.012 | .501 | .280 | .000 | .000 | .000 | .000 | .000 | .000 | .000 | .000 |
| Sm | .056 | .044 | -.012 | .516 | .290 | .000 | .000 | .000 | .000 | .000 | .000 | .000 | .000 |
| EE\_20 | .046 | .048 | -.019 | .318 | .192 | .176 | .610 | .000 | .000 | .000 | .000 | .000 | .000 |
| EE\_19 | .046 | .047 | -.019 | .314 | .190 | .174 | .603 | .000 | .000 | .000 | .000 | .000 | .000 |
| EE\_18 | .048 | .049 | -.019 | .324 | .196 | .180 | .621 | .000 | .000 | .000 | .000 | .000 | .000 |
| EE\_17 | .049 | .050 | -.020 | .332 | .200 | .183 | .634 | .000 | .000 | .000 | .000 | .000 | .000 |
| EE\_16 | .048 | .050 | -.020 | .330 | .199 | .183 | .631 | .000 | .000 | .000 | .000 | .000 | .000 |
| EE\_15 | .049 | .051 | -.020 | .336 | .202 | .185 | .644 | .000 | .000 | .000 | .000 | .000 | .000 |
| EE\_14 | .048 | .049 | -.020 | .330 | .199 | .182 | .630 | .000 | .000 | .000 | .000 | .000 | .000 |
| EE\_13 | .049 | .051 | -.021 | .336 | .203 | .186 | .643 | .000 | .000 | .000 | .000 | .000 | .000 |
| EE\_12 | .048 | .049 | -.020 | .327 | .197 | .181 | .623 | .000 | .000 | .000 | .000 | .000 | .000 |
| EE\_11 | .048 | .049 | -.020 | .326 | .196 | .182 | .624 | .000 | .000 | .000 | .000 | .000 | .000 |
| EE\_10 | .048 | .049 | -.020 | .328 | .197 | .182 | .625 | .000 | .000 | .000 | .000 | .000 | .000 |
| EE\_9 | .048 | .050 | -.020 | .329 | .198 | .183 | .628 | .000 | .000 | .000 | .000 | .000 | .000 |
| EE\_8 | .046 | .048 | -.019 | .318 | .191 | .176 | .608 | .000 | .000 | .000 | .000 | .000 | .000 |
| EE\_7 | .047 | .049 | -.020 | .324 | .195 | .179 | .618 | .000 | .000 | .000 | .000 | .000 | .000 |
| EE\_6 | .048 | .049 | -.020 | .325 | .196 | .181 | .624 | .000 | .000 | .000 | .000 | .000 | .000 |
| EE\_5 | .047 | .048 | -.019 | .320 | .192 | .176 | .612 | .000 | .000 | .000 | .000 | .000 | .000 |
| EE\_4 | .047 | .048 | -.020 | .323 | .194 | .178 | .616 | .000 | .000 | .000 | .000 | .000 | .000 |
| EE\_3 | .048 | .050 | -.020 | .328 | .197 | .181 | .628 | .000 | .000 | .000 | .000 | .000 | .000 |
| EE\_2 | .047 | .048 | -.019 | .319 | .192 | .176 | .609 | .000 | .000 | .000 | .000 | .000 | .000 |
| EE\_1 | .046 | .048 | -.019 | .317 | .191 | .175 | .608 | .000 | .000 | .000 | .000 | .000 | .000 |
| ESE\_10 | .040 | .031 | -.009 | .373 | .206 | .610 | .000 | .000 | .000 | .000 | .000 | .000 | .000 |
| ESE\_9 | .039 | .031 | -.009 | .362 | .202 | .594 | .000 | .000 | .000 | .000 | .000 | .000 | .000 |
| ESE\_8 | .041 | .032 | -.009 | .379 | .210 | .618 | .000 | .000 | .000 | .000 | .000 | .000 | .000 |
| ESE\_7 | .040 | .032 | -.009 | .376 | .209 | .615 | .000 | .000 | .000 | .000 | .000 | .000 | .000 |
| ESE\_6 | .041 | .032 | -.009 | .376 | .210 | .616 | .000 | .000 | .000 | .000 | .000 | .000 | .000 |
| ESE\_5 | .042 | .033 | -.009 | .385 | .215 | .631 | .000 | .000 | .000 | .000 | .000 | .000 | .000 |
| ESE\_4 | .042 | .033 | -.009 | .384 | .215 | .631 | .000 | .000 | .000 | .000 | .000 | .000 | .000 |
| ESE\_3 | .040 | .032 | -.009 | .372 | .208 | .609 | .000 | .000 | .000 | .000 | .000 | .000 | .000 |
| ESE\_2 | .041 | .033 | -.009 | .382 | .214 | .626 | .000 | .000 | .000 | .000 | .000 | .000 | .000 |
| ESE\_1 | .040 | .031 | -.008 | .371 | .208 | .608 | .000 | .000 | .000 | .000 | .000 | .000 | .000 |
| EOE\_9 | .046 | .052 | .002 | .422 | .000 | .000 | .000 | .000 | .000 | .000 | .000 | .000 | .000 |
| EOE\_8 | .047 | .053 | .002 | .426 | .000 | .000 | .000 | .000 | .000 | .000 | .000 | .000 | .000 |
| EOE\_7 | .048 | .054 | .002 | .436 | .000 | .000 | .000 | .000 | .000 | .000 | .000 | .000 | .000 |
| EOE\_6 | .046 | .052 | .002 | .420 | .000 | .000 | .000 | .000 | .000 | .000 | .000 | .000 | .000 |
| EOE\_5 | .047 | .054 | .002 | .432 | .000 | .000 | .000 | .000 | .000 | .000 | .000 | .000 | .000 |
| EOE\_4 | .047 | .053 | .002 | .424 | .000 | .000 | .000 | .000 | .000 | .000 | .000 | .000 | .000 |
| EOE\_3 | .046 | .053 | .002 | .422 | .000 | .000 | .000 | .000 | .000 | .000 | .000 | .000 | .000 |
| EOE\_2 | .047 | .053 | .002 | .426 | .000 | .000 | .000 | .000 | .000 | .000 | .000 | .000 | .000 |
| EOE\_1 | .046 | .052 | .002 | .421 | .000 | .000 | .000 | .000 | .000 | .000 | .000 | .000 | .000 |
| AISE\_6 | .000 | .000 | .000 | .000 | .000 | .000 | .000 | .000 | .000 | .000 | .000 | .000 | .000 |
| AISE\_5 | .000 | .000 | .000 | .000 | .000 | .000 | .000 | .000 | .000 | .000 | .000 | .000 | .000 |
| AISE\_4 | .000 | .000 | .000 | .000 | .000 | .000 | .000 | .000 | .000 | .000 | .000 | .000 | .000 |
| AISE\_3 | .000 | .000 | .000 | .000 | .000 | .000 | .000 | .000 | .000 | .000 | .000 | .000 | .000 |
| AISE\_2 | .000 | .000 | .000 | .000 | .000 | .000 | .000 | .000 | .000 | .000 | .000 | .000 | .000 |
| AISE\_1 | .000 | .000 | .000 | .000 | .000 | .000 | .000 | .000 | .000 | .000 | .000 | .000 | .000 |

## Standardized Indirect Effects - Two Tailed Significance (BC) (Group number 1 - Default model)

|  | Grade | Age | Gender | AISE | EOE | ESE | EE | Pa | Vc | Fs | Vp | Ss | Sm |
| --- | --- | --- | --- | --- | --- | --- | --- | --- | --- | --- | --- | --- | --- |
| EOE | ... | ... | ... | ... | ... | ... | ... | ... | ... | ... | ... | ... | ... |
| ESE | .130 | .077 | .064 | .000 | ... | ... | ... | ... | ... | ... | ... | ... | ... |
| EE | .097 | .203 | .003 | .000 | .000 | ... | ... | ... | ... | ... | ... | ... | ... |
| Pa | .058 | .043 | .001 | .000 | .000 | .000 | ... | ... | ... | ... | ... | ... | ... |
| Vc | .057 | .042 | .001 | .000 | .000 | .000 | ... | ... | ... | ... | ... | ... | ... |
| Fs | .058 | .040 | .001 | .000 | .000 | .000 | ... | ... | ... | ... | ... | ... | ... |
| Vp | .059 | .041 | .001 | .000 | .000 | .000 | ... | ... | ... | ... | ... | ... | ... |
| Ss | .249 | .646 | .009 | .000 | .000 | ... | ... | ... | ... | ... | ... | ... | ... |
| Sm | .247 | .641 | .010 | .000 | .000 | ... | ... | ... | ... | ... | ... | ... | ... |
| EE\_20 | .058 | .042 | .001 | .000 | .000 | .000 | .000 | ... | ... | ... | ... | ... | ... |
| EE\_19 | .059 | .042 | .001 | .000 | .000 | .000 | .000 | ... | ... | ... | ... | ... | ... |
| EE\_18 | .056 | .041 | .001 | .000 | .000 | .000 | .000 | ... | ... | ... | ... | ... | ... |
| EE\_17 | .058 | .042 | .001 | .000 | .000 | .000 | .000 | ... | ... | ... | ... | ... | ... |
| EE\_16 | .057 | .042 | .001 | .000 | .000 | .000 | .000 | ... | ... | ... | ... | ... | ... |
| EE\_15 | .058 | .041 | .001 | .000 | .000 | .000 | .000 | ... | ... | ... | ... | ... | ... |
| EE\_14 | .059 | .042 | .001 | .000 | .000 | .000 | .000 | ... | ... | ... | ... | ... | ... |
| EE\_13 | .057 | .042 | .001 | .000 | .000 | .000 | .000 | ... | ... | ... | ... | ... | ... |
| EE\_12 | .057 | .040 | .001 | .000 | .000 | .000 | .000 | ... | ... | ... | ... | ... | ... |
| EE\_11 | .058 | .040 | .001 | .000 | .000 | .000 | .000 | ... | ... | ... | ... | ... | ... |
| EE\_10 | .057 | .041 | .001 | .000 | .000 | .000 | .000 | ... | ... | ... | ... | ... | ... |
| EE\_9 | .057 | .040 | .001 | .000 | .000 | .000 | .000 | ... | ... | ... | ... | ... | ... |
| EE\_8 | .057 | .040 | .001 | .000 | .000 | .000 | .000 | ... | ... | ... | ... | ... | ... |
| EE\_7 | .057 | .041 | .001 | .000 | .000 | .000 | .000 | ... | ... | ... | ... | ... | ... |
| EE\_6 | .058 | .041 | .001 | .000 | .000 | .000 | .000 | ... | ... | ... | ... | ... | ... |
| EE\_5 | .058 | .040 | .001 | .000 | .000 | .000 | .000 | ... | ... | ... | ... | ... | ... |
| EE\_4 | .059 | .041 | .001 | .000 | .000 | .000 | .000 | ... | ... | ... | ... | ... | ... |
| EE\_3 | .059 | .041 | .001 | .000 | .000 | .000 | .000 | ... | ... | ... | ... | ... | ... |
| EE\_2 | .058 | .041 | .001 | .000 | .000 | .000 | .000 | ... | ... | ... | ... | ... | ... |
| EE\_1 | .059 | .041 | .001 | .000 | .000 | .000 | .000 | ... | ... | ... | ... | ... | ... |
| ESE\_10 | .246 | .642 | .009 | .000 | .000 | .000 | ... | ... | ... | ... | ... | ... | ... |
| ESE\_9 | .249 | .647 | .009 | .000 | .000 | .000 | ... | ... | ... | ... | ... | ... | ... |
| ESE\_8 | .246 | .645 | .009 | .000 | .000 | .000 | ... | ... | ... | ... | ... | ... | ... |
| ESE\_7 | .251 | .650 | .009 | .000 | .000 | .000 | ... | ... | ... | ... | ... | ... | ... |
| ESE\_6 | .248 | .643 | .009 | .000 | .000 | .000 | ... | ... | ... | ... | ... | ... | ... |
| ESE\_5 | .245 | .643 | .009 | .000 | .000 | .000 | ... | ... | ... | ... | ... | ... | ... |
| ESE\_4 | .249 | .647 | .010 | .000 | .000 | .000 | ... | ... | ... | ... | ... | ... | ... |
| ESE\_3 | .248 | .645 | .009 | .000 | .000 | .000 | ... | ... | ... | ... | ... | ... | ... |
| ESE\_2 | .249 | .646 | .009 | .000 | .000 | .000 | ... | ... | ... | ... | ... | ... | ... |
| ESE\_1 | .246 | .643 | .009 | .000 | .000 | .001 | ... | ... | ... | ... | ... | ... | ... |
| EOE\_9 | .136 | .085 | .070 | .000 | ... | ... | ... | ... | ... | ... | ... | ... | ... |
| EOE\_8 | .138 | .085 | .070 | .001 | ... | ... | ... | ... | ... | ... | ... | ... | ... |
| EOE\_7 | .139 | .085 | .070 | .000 | ... | ... | ... | ... | ... | ... | ... | ... | ... |
| EOE\_6 | .139 | .084 | .071 | .000 | ... | ... | ... | ... | ... | ... | ... | ... | ... |
| EOE\_5 | .138 | .086 | .070 | .000 | ... | ... | ... | ... | ... | ... | ... | ... | ... |
| EOE\_4 | .137 | .085 | .070 | .000 | ... | ... | ... | ... | ... | ... | ... | ... | ... |
| EOE\_3 | .135 | .084 | .070 | .000 | ... | ... | ... | ... | ... | ... | ... | ... | ... |
| EOE\_2 | .138 | .086 | .072 | .000 | ... | ... | ... | ... | ... | ... | ... | ... | ... |
| EOE\_1 | .137 | .084 | .071 | .001 | ... | ... | ... | ... | ... | ... | ... | ... | ... |
| AISE\_6 | ... | ... | ... | ... | ... | ... | ... | ... | ... | ... | ... | ... | ... |
| AISE\_5 | ... | ... | ... | ... | ... | ... | ... | ... | ... | ... | ... | ... | ... |
| AISE\_4 | ... | ... | ... | ... | ... | ... | ... | ... | ... | ... | ... | ... | ... |
| AISE\_3 | ... | ... | ... | ... | ... | ... | ... | ... | ... | ... | ... | ... | ... |
| AISE\_2 | ... | ... | ... | ... | ... | ... | ... | ... | ... | ... | ... | ... | ... |
| AISE\_1 | ... | ... | ... | ... | ... | ... | ... | ... | ... | ... | ... | ... | ... |

## Minimization History (Default model)

| Iteration |  | Negative eigenvalues | Condition # | Smallest eigenvalue | Diameter | F | NTries | Ratio |
| --- | --- | --- | --- | --- | --- | --- | --- | --- |
| 0 | e | 18 |  | -1.061 | 9999.000 | 48893.585 | 0 | 9999.000 |
| 1 | e | 10 |  | -.086 | 5.266 | 21352.863 | 20 | .253 |
| 2 | e | 2 |  | -.138 | 3.554 | 9440.197 | 5 | .533 |
| 3 | e | 0 | 325.774 |  | 1.410 | 2990.240 | 5 | 1.004 |
| 4 | e | 0 | 348.958 |  | .758 | 2844.372 | 3 | .000 |
| 5 | e | 0 | 327.510 |  | .869 | 1513.394 | 1 | 1.104 |
| 6 | e | 0 | 106.735 |  | .541 | 1259.329 | 1 | 1.151 |
| 7 | e | 0 | 87.179 |  | .259 | 1205.785 | 1 | 1.216 |
| 8 | e | 0 | 87.746 |  | .113 | 1194.387 | 1 | 1.148 |
| 9 | e | 0 | 87.422 |  | .031 | 1193.614 | 1 | 1.051 |
| 10 | e | 0 | 83.486 |  | .002 | 1193.609 | 1 | 1.004 |
| 11 | e | 0 | 83.490 |  | .000 | 1193.609 | 1 | 1.001 |

## Bootstrap (Default model)

## Summary of Bootstrap Iterations (Default model)

## (Default model)

| Iterations | Method 0 | Method 1 | Method 2 |
| --- | --- | --- | --- |
| 1 | 0 | 0 | 0 |
| 2 | 0 | 0 | 0 |
| 3 | 0 | 0 | 0 |
| 4 | 0 | 191 | 0 |
| 5 | 0 | 2830 | 0 |
| 6 | 0 | 1703 | 0 |
| 7 | 0 | 259 | 0 |
| 8 | 0 | 16 | 0 |
| 9 | 0 | 1 | 0 |
| 10 | 0 | 0 | 0 |
| 11 | 0 | 0 | 0 |
| 12 | 0 | 0 | 0 |
| 13 | 0 | 0 | 0 |
| 14 | 0 | 0 | 0 |
| 15 | 0 | 0 | 0 |
| 16 | 0 | 0 | 0 |
| 17 | 0 | 0 | 0 |
| 18 | 0 | 0 | 0 |
| 19 | 0 | 0 | 0 |
| Total | 0 | 5000 | 0 |

0 bootstrap samples were unused because of a singular covariance matrix.

0 bootstrap samples were unused because a solution was not found.

5000 usable bootstrap samples were obtained.

## Bootstrap Distributions (Default model)

## ML discrepancy (implied vs sample) (Default model)

|  |  |  |
| --- | --- | --- |
|  |  | \|-------------------- |
|  | 1951.496 | \|\* |
|  | 2000.326 | \|\* |
|  | 2049.156 | \|\*\* |
|  | 2097.986 | \|\*\*\*\*\* |
|  | 2146.816 | \|\*\*\*\*\*\*\*\*\*\* |
|  | 2195.646 | \|\*\*\*\*\*\*\*\*\*\*\*\*\*\*\*\*\* |
|  | 2244.476 | \|\*\*\*\*\*\*\*\*\*\*\*\*\*\*\*\*\*\*\*\* |
| N = 5000 | 2293.306 | \|\*\*\*\*\*\*\*\*\*\*\*\*\*\*\*\*\*\* |
| Mean = 2257.388 | 2342.136 | \|\*\*\*\*\*\*\*\*\*\*\*\*\* |
| S. e. = 1.268 | 2390.966 | \|\*\*\*\*\*\*\* |
|  | 2439.796 | \|\*\*\* |
|  | 2488.626 | \|\* |
|  | 2537.455 | \|\* |
|  | 2586.285 | \|\* |
|  | 2635.115 | \|\* |
|  |  | \|-------------------- |

## ML discrepancy (implied vs pop) (Default model)

|  |  |  |
| --- | --- | --- |
|  |  | \|-------------------- |
|  | 1302.132 | \|\* |
|  | 1311.733 | \|\*\* |
|  | 1321.334 | \|\*\*\*\* |
|  | 1330.935 | \|\*\*\*\*\*\*\*\*\*\*\* |
|  | 1340.536 | \|\*\*\*\*\*\*\*\*\*\*\*\*\*\*\*\*\* |
|  | 1350.138 | \|\*\*\*\*\*\*\*\*\*\*\*\*\*\*\*\*\*\*\*\* |
|  | 1359.739 | \|\*\*\*\*\*\*\*\*\*\*\*\*\*\*\*\*\*\*\*\* |
| N = 5000 | 1369.340 | \|\*\*\*\*\*\*\*\*\*\*\*\*\*\* |
| Mean = 1354.664 | 1378.941 | \|\*\*\*\*\*\*\*\*\* |
| S. e. = .260 | 1388.543 | \|\*\*\*\* |
|  | 1398.144 | \|\*\* |
|  | 1407.745 | \|\* |
|  | 1417.346 | \|\* |
|  | 1426.947 | \|\* |
|  | 1436.549 | \|\* |
|  |  | \|-------------------- |

## K-L overoptimism (unstabilized) (Default model)

|  |  |  |
| --- | --- | --- |
|  |  | \|-------------------- |
|  | -1143.910 | \|\* |
|  | -916.589 | \|\* |
|  | -689.269 | \|\*\* |
|  | -461.948 | \|\*\*\*\* |
|  | -234.628 | \|\*\*\*\*\*\*\*\*\*\* |
|  | -7.307 | \|\*\*\*\*\*\*\*\*\*\*\*\*\*\*\*\* |
|  | 220.013 | \|\*\*\*\*\*\*\*\*\*\*\*\*\*\*\*\*\*\*\*\* |
| N = 5000 | 447.334 | \|\*\*\*\*\*\*\*\*\*\*\*\*\*\*\*\*\*\*\* |
| Mean = 317.229 | 674.654 | \|\*\*\*\*\*\*\*\*\*\*\*\*\*\* |
| S. e. = 6.124 | 901.975 | \|\*\*\*\*\*\*\*\*\* |
|  | 1129.295 | \|\*\*\*\* |
|  | 1356.616 | \|\*\* |
|  | 1583.936 | \|\* |
|  | 1811.257 | \|\* |
|  | 2038.577 | \|\* |
|  |  | \|-------------------- |

## K-L overoptimism (stabilized) (Default model)

|  |  |  |
| --- | --- | --- |
|  |  | \|-------------------- |
|  | 70.686 | \|\* |
|  | 114.991 | \|\* |
|  | 159.295 | \|\*\*\* |
|  | 203.599 | \|\*\*\*\*\*\*\* |
|  | 247.904 | \|\*\*\*\*\*\*\*\*\*\*\*\*\*\* |
|  | 292.208 | \|\*\*\*\*\*\*\*\*\*\*\*\*\*\*\*\*\*\*\* |
|  | 336.512 | \|\*\*\*\*\*\*\*\*\*\*\*\*\*\*\*\*\*\*\*\* |
| N = 5000 | 380.817 | \|\*\*\*\*\*\*\*\*\*\*\*\*\*\*\*\* |
| Mean = 321.363 | 425.121 | \|\*\*\*\*\*\*\*\* |
| S. e. = 1.076 | 469.425 | \|\*\*\*\* |
|  | 513.730 | \|\* |
|  | 558.034 | \|\* |
|  | 602.338 | \|\* |
|  | 646.643 | \| |
|  | 690.947 | \|\* |
|  |  | \|-------------------- |

## ML discrepancy (implied vs pop) (Default model)

|  |  |  |
| --- | --- | --- |
|  |  | \|-------------------- |
|  | 1302.132 | \|\* |
|  | 1311.733 | \|\*\* |
|  | 1321.334 | \|\*\*\*\* |
|  | 1330.935 | \|\*\*\*\*\*\*\*\*\*\*\* |
|  | 1340.536 | \|\*\*\*\*\*\*\*\*\*\*\*\*\*\*\*\*\* |
|  | 1350.138 | \|\*\*\*\*\*\*\*\*\*\*\*\*\*\*\*\*\*\*\*\* |
|  | 1359.739 | \|\*\*\*\*\*\*\*\*\*\*\*\*\*\*\*\*\*\*\*\* |
| N = 5000 | 1369.340 | \|\*\*\*\*\*\*\*\*\*\*\*\*\*\* |
| Mean = 1354.664 | 1378.941 | \|\*\*\*\*\*\*\*\*\* |
| S. e. = .260 | 1388.543 | \|\*\*\*\* |
|  | 1398.144 | \|\*\* |
|  | 1407.745 | \|\* |
|  | 1417.346 | \|\* |
|  | 1426.947 | \|\* |
|  | 1436.549 | \|\* |
|  |  | \|-------------------- |

## Model Fit Summary

## CMIN

| Model | NPAR | CMIN | DF | P | CMIN/DF |
| --- | --- | --- | --- | --- | --- |
| Default model | 120 | 1193.609 | 1056 | .002 | 1.130 |
| Saturated model | 1176 | .000 | 0 |  |  |
| Independence model | 48 | 50180.015 | 1128 | .000 | 44.486 |

## RMR, GFI

| Model | RMR | GFI | AGFI | PGFI |
| --- | --- | --- | --- | --- |
| Default model | .010 | .979 | .976 | .879 |
| Saturated model | .000 | 1.000 |  |  |
| Independence model | .179 | .214 | .180 | .205 |

## Baseline Comparisons

| Model | NFI Delta1 | RFI rho1 | IFI Delta2 | TLI rho2 | CFI |
| --- | --- | --- | --- | --- | --- |
| Default model | .976 | .975 | .997 | .997 | .997 |
| Saturated model | 1.000 |  | 1.000 |  | 1.000 |
| Independence model | .000 | .000 | .000 | .000 | .000 |

## Parsimony-Adjusted Measures

| Model | PRATIO | PNFI | PCFI |
| --- | --- | --- | --- |
| Default model | .936 | .914 | .934 |
| Saturated model | .000 | .000 | .000 |
| Independence model | 1.000 | .000 | .000 |

## NCP

| Model | NCP | LO 90 | HI 90 |
| --- | --- | --- | --- |
| Default model | 137.609 | 56.871 | 226.653 |
| Saturated model | .000 | .000 | .000 |
| Independence model | 49052.015 | 48322.410 | 49787.944 |

## FMIN

| Model | FMIN | F0 | LO 90 | HI 90 |
| --- | --- | --- | --- | --- |
| Default model | .524 | .060 | .025 | .099 |
| Saturated model | .000 | .000 | .000 | .000 |
| Independence model | 22.028 | 21.533 | 21.213 | 21.856 |

## RMSEA

| Model | RMSEA | LO 90 | HI 90 | PCLOSE |
| --- | --- | --- | --- | --- |
| Default model | .008 | .005 | .010 | 1.000 |
| Independence model | .138 | .137 | .139 | .000 |

## AIC

| Model | AIC | BCC | BIC | CAIC |
| --- | --- | --- | --- | --- |
| Default model | 1433.609 | 1438.885 | 2121.388 | 2241.388 |
| Saturated model | 2352.000 | 2403.704 | 9092.235 | 10268.235 |
| Independence model | 50276.015 | 50278.125 | 50551.126 | 50599.126 |

## ECVI

| Model | ECVI | LO 90 | HI 90 | MECVI |
| --- | --- | --- | --- | --- |
| Default model | .629 | .594 | .668 | .632 |
| Saturated model | 1.032 | 1.032 | 1.032 | 1.055 |
| Independence model | 22.070 | 21.750 | 22.393 | 22.071 |

## HOELTER

| Model | HOELTER .05 | HOELTER .01 |
| --- | --- | --- |
| Default model | 2162 | 2226 |
| Independence model | 55 | 57 |

## Execution time summary

|  |  |
| --- | --- |
| Minimization: | .030 |
| Miscellaneous: | 1.275 |
| Bootstrap: | 29.240 |
| Total: | 30.545 |
