# Supplementary material for: AI-contextualized self-efficacy and exercise engagement among vocational college students in China: a cross-sectional serial mediation analysis of exercise self-efficacy and exercise outcome expectations
Source: Front Psychol. 2026 Oct 6;17:1933716. doi: 10.3389/fpsyg.2026.1933716 (PMC13680253; doi:10.3389/fpsyg.2026.1933716)
Supplement: Supplementary file 2 [file Data_Sheet_1.zip › Revision_Package_1933716/CFA_Model_1/CFA_Model_1.AmosOutput]

验证性因子分析模型.amw


# D:\系统默认\桌面\danzi\2025年9月份单子\361.数据分析-220元\验证性因子分析模型.amw

## Analysis Summary

## Date and Time

Date: 2026年9月13日

Time: 12:42:20

## Title

验证性因子分析模型: 2026年9月13日 12:42

## Groups

## Group number 1 (Group number 1)

## Notes for Group (Group number 1)

The model is recursive.

Sample size = 2279

## Variable Summary (Group number 1)

## Your model contains the following variables (Group number 1)

Observed, endogenous variables

AISE\_1

AISE\_2

AISE\_3

AISE\_4

AISE\_5

AISE\_6

EOE\_1

EOE\_2

EOE\_3

EOE\_4

EOE\_5

EOE\_6

EOE\_7

EOE\_8

EOE\_9

ESE\_1

ESE\_2

ESE\_3

ESE\_4

ESE\_5

ESE\_6

ESE\_7

ESE\_8

ESE\_9

ESE\_10

EE\_1

EE\_2

EE\_3

EE\_4

EE\_5

EE\_6

EE\_7

EE\_8

EE\_9

EE\_10

EE\_11

EE\_12

EE\_13

EE\_14

EE\_15

EE\_16

EE\_17

EE\_18

EE\_19

EE\_20

Unobserved, endogenous variables

Sm

Ss

Vp

Fs

Vc

Pa

Unobserved, exogenous variables

AISE

e1

e2

e3

e4

e5

e6

EOE

e7

e8

e9

e10

e11

e12

e13

e14

e15

e16

e17

e18

e19

e20

e21

e22

e23

e24

e25

ESE

e26

e27

e28

e29

e30

e31

e32

e33

e34

e35

e36

e37

e38

e39

e40

e41

e42

e43

e44

e45

EE

e46

e47

e48

e49

e50

e51

## Variable counts (Group number 1)

|  |  |
| --- | --- |
| Number of variables in your model: | 106 |
| Number of observed variables: | 45 |
| Number of unobserved variables: | 61 |
| Number of exogenous variables: | 55 |
| Number of endogenous variables: | 51 |

## Parameter Summary (Group number 1)

|  | Weights | Covariances | Variances | Means | Intercepts | Total |
| --- | --- | --- | --- | --- | --- | --- |
| Fixed | 61 | 0 | 0 | 0 | 0 | 61 |
| Labeled | 0 | 0 | 0 | 0 | 0 | 0 |
| Unlabeled | 41 | 6 | 55 | 0 | 0 | 102 |
| Total | 102 | 6 | 55 | 0 | 0 | 163 |

## Models

## Default model (Default model)

## Notes for Model (Default model)

## Computation of degrees of freedom (Default model)

|  |  |
| --- | --- |
| Number of distinct sample moments: | 1035 |
| Number of distinct parameters to be estimated: | 102 |
| Degrees of freedom (1035 - 102): | 933 |

## Result (Default model)

Minimum was achieved

Chi-square = 1035.773

Degrees of freedom = 933

Probability level = .010

## Group number 1 (Group number 1 - Default model)

## Estimates (Group number 1 - Default model)

## Scalar Estimates (Group number 1 - Default model)

## Maximum Likelihood Estimates

## Regression Weights: (Group number 1 - Default model)

|  |  |  | Estimate | S.E. | C.R. | P | Label |
| --- | --- | --- | --- | --- | --- | --- | --- |
| Sm | <--- | ESE | 1.000 |  |  |  |  |
| Ss | <--- | ESE | .997 | .051 | 19.723 | \*\*\* |  |
| Vp | <--- | EE | 1.000 |  |  |  |  |
| Fs | <--- | EE | 1.034 | .042 | 24.501 | \*\*\* |  |
| Vc | <--- | EE | 1.062 | .043 | 24.962 | \*\*\* |  |
| Pa | <--- | EE | .976 | .041 | 23.774 | \*\*\* |  |
| AISE\_1 | <--- | AISE | 1.000 |  |  |  |  |
| AISE\_2 | <--- | AISE | .983 | .029 | 34.111 | \*\*\* |  |
| AISE\_3 | <--- | AISE | .959 | .029 | 33.196 | \*\*\* |  |
| AISE\_4 | <--- | AISE | .992 | .029 | 33.709 | \*\*\* |  |
| AISE\_5 | <--- | AISE | .955 | .028 | 34.266 | \*\*\* |  |
| AISE\_6 | <--- | AISE | .999 | .029 | 33.907 | \*\*\* |  |
| EOE\_1 | <--- | EOE | 1.000 |  |  |  |  |
| EOE\_2 | <--- | EOE | 1.014 | .030 | 33.441 | \*\*\* |  |
| EOE\_3 | <--- | EOE | 1.002 | .030 | 33.149 | \*\*\* |  |
| EOE\_4 | <--- | EOE | .993 | .030 | 33.272 | \*\*\* |  |
| EOE\_5 | <--- | EOE | 1.032 | .030 | 33.931 | \*\*\* |  |
| EOE\_6 | <--- | EOE | .992 | .030 | 32.935 | \*\*\* |  |
| EOE\_7 | <--- | EOE | 1.030 | .030 | 34.275 | \*\*\* |  |
| EOE\_8 | <--- | EOE | 1.024 | .031 | 33.465 | \*\*\* |  |
| EOE\_9 | <--- | EOE | 1.002 | .030 | 33.022 | \*\*\* |  |
| ESE\_1 | <--- | Sm | 1.000 |  |  |  |  |
| ESE\_2 | <--- | Sm | 1.028 | .032 | 32.277 | \*\*\* |  |
| ESE\_3 | <--- | Sm | .982 | .031 | 31.437 | \*\*\* |  |
| ESE\_4 | <--- | Sm | 1.047 | .032 | 32.532 | \*\*\* |  |
| ESE\_5 | <--- | Sm | 1.033 | .032 | 32.523 | \*\*\* |  |
| ESE\_6 | <--- | Sm | 1.028 | .032 | 31.754 | \*\*\* |  |
| ESE\_7 | <--- | Ss | 1.000 |  |  |  |  |
| ESE\_8 | <--- | Ss | 1.006 | .031 | 32.643 | \*\*\* |  |
| ESE\_9 | <--- | Ss | .947 | .030 | 31.414 | \*\*\* |  |
| ESE\_10 | <--- | Ss | .982 | .031 | 32.150 | \*\*\* |  |
| EE\_1 | <--- | Vp | 1.000 |  |  |  |  |
| EE\_2 | <--- | Vp | 1.020 | .031 | 32.392 | \*\*\* |  |
| EE\_3 | <--- | Vp | 1.049 | .031 | 33.354 | \*\*\* |  |
| EE\_4 | <--- | Vp | 1.022 | .031 | 32.838 | \*\*\* |  |
| EE\_5 | <--- | Vp | 1.027 | .032 | 32.570 | \*\*\* |  |
| EE\_6 | <--- | Vp | 1.037 | .031 | 33.202 | \*\*\* |  |
| EE\_7 | <--- | Fs | 1.000 |  |  |  |  |
| EE\_8 | <--- | Fs | .972 | .030 | 32.528 | \*\*\* |  |
| EE\_9 | <--- | Fs | 1.007 | .030 | 33.658 | \*\*\* |  |
| EE\_10 | <--- | Fs | 1.008 | .030 | 33.541 | \*\*\* |  |
| EE\_11 | <--- | Fs | 1.003 | .030 | 33.496 | \*\*\* |  |
| EE\_12 | <--- | Fs | .993 | .030 | 33.449 | \*\*\* |  |
| EE\_13 | <--- | Vc | 1.000 |  |  |  |  |
| EE\_14 | <--- | Vc | .973 | .029 | 34.017 | \*\*\* |  |
| EE\_15 | <--- | Vc | .989 | .028 | 34.721 | \*\*\* |  |
| EE\_16 | <--- | Vc | .977 | .029 | 34.075 | \*\*\* |  |
| EE\_17 | <--- | Vc | .970 | .028 | 34.210 | \*\*\* |  |
| EE\_18 | <--- | Pa | 1.000 |  |  |  |  |
| EE\_19 | <--- | Pa | .972 | .033 | 29.575 | \*\*\* |  |
| EE\_20 | <--- | Pa | .990 | .033 | 29.857 | \*\*\* |  |

## Standardized Regression Weights: (Group number 1 - Default model)

|  |  |  | Estimate |
| --- | --- | --- | --- |
| Sm | <--- | ESE | .810 |
| Ss | <--- | ESE | .786 |
| Vp | <--- | EE | .807 |
| Fs | <--- | EE | .814 |
| Vc | <--- | EE | .824 |
| Pa | <--- | EE | .802 |
| AISE\_1 | <--- | AISE | .721 |
| AISE\_2 | <--- | AISE | .757 |
| AISE\_3 | <--- | AISE | .736 |
| AISE\_4 | <--- | AISE | .748 |
| AISE\_5 | <--- | AISE | .760 |
| AISE\_6 | <--- | AISE | .752 |
| EOE\_1 | <--- | EOE | .720 |
| EOE\_2 | <--- | EOE | .727 |
| EOE\_3 | <--- | EOE | .721 |
| EOE\_4 | <--- | EOE | .724 |
| EOE\_5 | <--- | EOE | .738 |
| EOE\_6 | <--- | EOE | .716 |
| EOE\_7 | <--- | EOE | .745 |
| EOE\_8 | <--- | EOE | .728 |
| EOE\_9 | <--- | EOE | .718 |
| ESE\_1 | <--- | Sm | .711 |
| ESE\_2 | <--- | Sm | .733 |
| ESE\_3 | <--- | Sm | .713 |
| ESE\_4 | <--- | Sm | .739 |
| ESE\_5 | <--- | Sm | .739 |
| ESE\_6 | <--- | Sm | .721 |
| ESE\_7 | <--- | Ss | .744 |
| ESE\_8 | <--- | Ss | .747 |
| ESE\_9 | <--- | Ss | .716 |
| ESE\_10 | <--- | Ss | .735 |
| EE\_1 | <--- | Vp | .719 |
| EE\_2 | <--- | Vp | .722 |
| EE\_3 | <--- | Vp | .744 |
| EE\_4 | <--- | Vp | .732 |
| EE\_5 | <--- | Vp | .726 |
| EE\_6 | <--- | Vp | .740 |
| EE\_7 | <--- | Fs | .729 |
| EE\_8 | <--- | Fs | .715 |
| EE\_9 | <--- | Fs | .739 |
| EE\_10 | <--- | Fs | .737 |
| EE\_11 | <--- | Fs | .736 |
| EE\_12 | <--- | Fs | .735 |
| EE\_13 | <--- | Vc | .750 |
| EE\_14 | <--- | Vc | .734 |
| EE\_15 | <--- | Vc | .749 |
| EE\_16 | <--- | Vc | .735 |
| EE\_17 | <--- | Vc | .738 |
| EE\_18 | <--- | Pa | .738 |
| EE\_19 | <--- | Pa | .714 |
| EE\_20 | <--- | Pa | .723 |

## Covariances: (Group number 1 - Default model)

|  |  |  | Estimate | S.E. | C.R. | P | Label |
| --- | --- | --- | --- | --- | --- | --- | --- |
| AISE | <--> | EOE | .228 | .013 | 18.129 | \*\*\* |  |
| AISE | <--> | ESE | .210 | .013 | 16.663 | \*\*\* |  |
| AISE | <--> | EE | .180 | .011 | 16.026 | \*\*\* |  |
| EOE | <--> | ESE | .126 | .008 | 15.883 | \*\*\* |  |
| EOE | <--> | EE | .112 | .007 | 15.553 | \*\*\* |  |
| ESE | <--> | EE | .100 | .007 | 14.222 | \*\*\* |  |

## Correlations: (Group number 1 - Default model)

|  |  |  | Estimate |
| --- | --- | --- | --- |
| AISE | <--> | EOE | .553 |
| AISE | <--> | ESE | .597 |
| AISE | <--> | EE | .507 |
| EOE | <--> | ESE | .541 |
| EOE | <--> | EE | .476 |
| ESE | <--> | EE | .500 |

## Variances: (Group number 1 - Default model)

|  |  |  | Estimate | S.E. | C.R. | P | Label |
| --- | --- | --- | --- | --- | --- | --- | --- |
| AISE |  |  | .623 | .033 | 19.068 | \*\*\* |  |
| EOE |  |  | .274 | .014 | 19.267 | \*\*\* |  |
| ESE |  |  | .199 | .014 | 13.762 | \*\*\* |  |
| EE |  |  | .201 | .013 | 15.409 | \*\*\* |  |
| e46 |  |  | .104 | .010 | 10.859 | \*\*\* |  |
| e47 |  |  | .122 | .010 | 11.836 | \*\*\* |  |
| e48 |  |  | .107 | .007 | 14.487 | \*\*\* |  |
| e49 |  |  | .110 | .008 | 14.440 | \*\*\* |  |
| e50 |  |  | .107 | .008 | 14.067 | \*\*\* |  |
| e51 |  |  | .106 | .008 | 12.715 | \*\*\* |  |
| e1 |  |  | .576 | .020 | 29.267 | \*\*\* |  |
| e2 |  |  | .449 | .016 | 28.179 | \*\*\* |  |
| e3 |  |  | .485 | .017 | 28.841 | \*\*\* |  |
| e4 |  |  | .483 | .017 | 28.484 | \*\*\* |  |
| e5 |  |  | .415 | .015 | 28.055 | \*\*\* |  |
| e6 |  |  | .477 | .017 | 28.337 | \*\*\* |  |
| e7 |  |  | .255 | .008 | 30.318 | \*\*\* |  |
| e8 |  |  | .251 | .008 | 30.163 | \*\*\* |  |
| e9 |  |  | .255 | .008 | 30.292 | \*\*\* |  |
| e10 |  |  | .246 | .008 | 30.238 | \*\*\* |  |
| e11 |  |  | .245 | .008 | 29.931 | \*\*\* |  |
| e12 |  |  | .256 | .008 | 30.383 | \*\*\* |  |
| e13 |  |  | .233 | .008 | 29.756 | \*\*\* |  |
| e14 |  |  | .256 | .008 | 30.152 | \*\*\* |  |
| e15 |  |  | .258 | .009 | 30.346 | \*\*\* |  |
| e16 |  |  | .296 | .010 | 29.040 | \*\*\* |  |
| e17 |  |  | .275 | .010 | 28.398 | \*\*\* |  |
| e18 |  |  | .283 | .010 | 28.993 | \*\*\* |  |
| e19 |  |  | .275 | .010 | 28.197 | \*\*\* |  |
| e20 |  |  | .269 | .010 | 28.204 | \*\*\* |  |
| e21 |  |  | .296 | .010 | 28.780 | \*\*\* |  |
| e22 |  |  | .257 | .010 | 25.812 | \*\*\* |  |
| e23 |  |  | .255 | .010 | 25.657 | \*\*\* |  |
| e24 |  |  | .272 | .010 | 27.030 | \*\*\* |  |
| e25 |  |  | .262 | .010 | 26.258 | \*\*\* |  |
| e26 |  |  | .288 | .010 | 29.164 | \*\*\* |  |
| e27 |  |  | .296 | .010 | 29.103 | \*\*\* |  |
| e28 |  |  | .275 | .010 | 28.455 | \*\*\* |  |
| e29 |  |  | .280 | .010 | 28.817 | \*\*\* |  |
| e30 |  |  | .293 | .010 | 28.992 | \*\*\* |  |
| e31 |  |  | .274 | .010 | 28.566 | \*\*\* |  |
| e32 |  |  | .287 | .010 | 28.953 | \*\*\* |  |
| e33 |  |  | .294 | .010 | 29.335 | \*\*\* |  |
| e34 |  |  | .273 | .010 | 28.643 | \*\*\* |  |
| e35 |  |  | .278 | .010 | 28.721 | \*\*\* |  |
| e36 |  |  | .277 | .010 | 28.751 | \*\*\* |  |
| e37 |  |  | .274 | .010 | 28.781 | \*\*\* |  |
| e38 |  |  | .260 | .009 | 27.703 | \*\*\* |  |
| e39 |  |  | .271 | .010 | 28.266 | \*\*\* |  |
| e40 |  |  | .256 | .009 | 27.757 | \*\*\* |  |
| e41 |  |  | .271 | .010 | 28.226 | \*\*\* |  |
| e42 |  |  | .263 | .009 | 28.132 | \*\*\* |  |
| e43 |  |  | .249 | .010 | 24.246 | \*\*\* |  |
| e44 |  |  | .271 | .011 | 25.500 | \*\*\* |  |
| e45 |  |  | .266 | .011 | 25.049 | \*\*\* |  |

## Modification Indices (Group number 1 - Default model)

## Covariances: (Group number 1 - Default model)

|  |  |  | M.I. | Par Change |
| --- | --- | --- | --- | --- |
| e44 | <--> | EOE | 5.313 | .013 |
| e41 | <--> | AISE | 8.289 | -.023 |
| e41 | <--> | e42 | 4.017 | .013 |
| e40 | <--> | e46 | 5.984 | -.013 |
| e40 | <--> | e44 | 5.929 | .016 |
| e39 | <--> | AISE | 6.356 | .020 |
| e39 | <--> | e51 | 5.088 | -.013 |
| e38 | <--> | e46 | 4.543 | .012 |
| e38 | <--> | e42 | 4.790 | -.014 |
| e36 | <--> | e46 | 4.008 | -.011 |
| e33 | <--> | e41 | 4.693 | .015 |
| e31 | <--> | e36 | 6.768 | .017 |
| e30 | <--> | e51 | 6.273 | .015 |
| e30 | <--> | e46 | 4.457 | -.012 |
| e29 | <--> | AISE | 6.279 | .020 |
| e29 | <--> | e50 | 4.888 | -.012 |
| e29 | <--> | e48 | 4.148 | .010 |
| e28 | <--> | e47 | 8.165 | .017 |
| e27 | <--> | EOE | 6.428 | .014 |
| e26 | <--> | e43 | 5.979 | -.017 |
| e25 | <--> | AISE | 4.088 | .016 |
| e25 | <--> | e35 | 4.091 | -.014 |
| e24 | <--> | e45 | 6.594 | .018 |
| e24 | <--> | e30 | 4.019 | .014 |
| e23 | <--> | EOE | 5.059 | -.012 |
| e23 | <--> | e41 | 5.743 | -.016 |
| e23 | <--> | e37 | 6.428 | .017 |
| e23 | <--> | e28 | 4.966 | .015 |
| e22 | <--> | AISE | 6.045 | -.019 |
| e21 | <--> | e37 | 6.532 | .017 |
| e20 | <--> | e39 | 5.298 | -.015 |
| e20 | <--> | e24 | 4.804 | -.015 |
| e20 | <--> | e23 | 6.916 | .017 |
| e19 | <--> | e33 | 4.132 | .014 |
| e18 | <--> | e45 | 4.278 | .014 |
| e16 | <--> | e45 | 4.557 | -.015 |
| e16 | <--> | e27 | 4.723 | -.015 |
| e16 | <--> | e26 | 6.154 | .017 |
| e16 | <--> | e23 | 5.227 | -.016 |
| e15 | <--> | e21 | 5.606 | .015 |
| e15 | <--> | e18 | 11.288 | -.021 |
| e14 | <--> | e18 | 7.502 | .017 |
| e13 | <--> | e32 | 4.129 | -.012 |
| e13 | <--> | e29 | 7.807 | .017 |
| e12 | <--> | e27 | 4.532 | -.014 |
| e12 | <--> | e21 | 5.109 | .015 |
| e11 | <--> | e47 | 5.568 | -.013 |
| e11 | <--> | e46 | 4.641 | .011 |
| e11 | <--> | e23 | 4.194 | -.013 |
| e11 | <--> | e15 | 4.816 | .013 |
| e11 | <--> | e12 | 6.010 | .014 |
| e10 | <--> | e42 | 5.469 | -.014 |
| e10 | <--> | e33 | 4.764 | -.014 |
| e10 | <--> | e15 | 10.428 | -.019 |
| e10 | <--> | e12 | 6.095 | -.014 |
| e9 | <--> | e21 | 8.373 | -.019 |
| e9 | <--> | e20 | 8.773 | .018 |
| e9 | <--> | e11 | 9.200 | -.017 |
| e9 | <--> | e10 | 6.095 | .014 |
| e8 | <--> | e32 | 5.307 | .015 |
| e8 | <--> | e28 | 6.840 | -.016 |
| e8 | <--> | e21 | 6.285 | -.016 |
| e8 | <--> | e12 | 4.853 | -.013 |
| e8 | <--> | e10 | 8.288 | .016 |
| e8 | <--> | e9 | 5.714 | .014 |
| e7 | <--> | e11 | 5.606 | -.014 |
| e7 | <--> | e8 | 5.591 | .014 |
| e6 | <--> | e37 | 4.641 | -.019 |
| e6 | <--> | e25 | 5.118 | .020 |
| e6 | <--> | e22 | 7.528 | -.024 |
| e6 | <--> | e13 | 4.921 | -.018 |
| e5 | <--> | e13 | 4.199 | .015 |
| e5 | <--> | e6 | 6.830 | .028 |
| e4 | <--> | e50 | 7.511 | -.020 |
| e4 | <--> | e41 | 9.676 | -.027 |
| e3 | <--> | e30 | 4.151 | .018 |
| e3 | <--> | e27 | 9.201 | -.027 |
| e2 | <--> | e37 | 4.404 | .018 |
| e1 | <--> | e24 | 5.369 | -.022 |
| e1 | <--> | e5 | 9.229 | -.035 |

## Variances: (Group number 1 - Default model)

|  |  |  | M.I. | Par Change |
| --- | --- | --- | --- | --- |

## Regression Weights: (Group number 1 - Default model)

|  |  |  | M.I. | Par Change |
| --- | --- | --- | --- | --- |
| EE\_19 | <--- | EOE | 4.806 | .053 |
| EE\_19 | <--- | EOE\_9 | 4.309 | .034 |
| EE\_19 | <--- | EOE\_6 | 6.781 | .044 |
| EE\_19 | <--- | EOE\_5 | 6.172 | .041 |
| EE\_16 | <--- | AISE | 4.897 | -.035 |
| EE\_16 | <--- | AISE\_6 | 4.742 | -.024 |
| EE\_16 | <--- | AISE\_4 | 12.119 | -.039 |
| EE\_16 | <--- | AISE\_1 | 4.080 | -.022 |
| EE\_15 | <--- | EE\_19 | 4.620 | .033 |
| EE\_15 | <--- | ESE\_6 | 5.690 | -.035 |
| EE\_15 | <--- | ESE\_3 | 5.280 | -.035 |
| EE\_14 | <--- | AISE\_6 | 4.032 | .023 |
| EE\_13 | <--- | ESE\_5 | 5.453 | .035 |
| EE\_13 | <--- | ESE\_4 | 4.937 | .033 |
| EE\_13 | <--- | ESE\_3 | 5.282 | .035 |
| EE\_12 | <--- | ESE\_8 | 4.452 | .033 |
| EE\_12 | <--- | ESE\_6 | 4.722 | .033 |
| EE\_12 | <--- | EOE\_5 | 4.710 | .035 |
| EE\_12 | <--- | EOE\_3 | 4.286 | .033 |
| EE\_12 | <--- | AISE\_2 | 4.881 | .025 |
| EE\_11 | <--- | Sm | 4.777 | -.050 |
| EE\_11 | <--- | ESE\_6 | 4.291 | -.031 |
| EE\_11 | <--- | ESE\_2 | 5.769 | -.037 |
| EE\_11 | <--- | EOE\_9 | 6.314 | -.041 |
| EE\_11 | <--- | EOE\_5 | 5.153 | -.037 |
| EE\_6 | <--- | EE\_11 | 6.303 | .038 |
| EE\_6 | <--- | EE\_8 | 4.207 | .031 |
| EE\_4 | <--- | Ss | 4.483 | -.048 |
| EE\_4 | <--- | EE\_17 | 4.282 | -.032 |
| EE\_4 | <--- | EE\_13 | 4.182 | -.031 |
| EE\_4 | <--- | ESE\_9 | 4.768 | -.035 |
| EE\_3 | <--- | Ss | 4.495 | .048 |
| EE\_3 | <--- | ESE\_8 | 7.749 | .043 |
| EE\_3 | <--- | ESE\_7 | 5.495 | .037 |
| EE\_2 | <--- | EOE\_9 | 4.806 | .036 |
| EE\_2 | <--- | EOE\_7 | 4.425 | .035 |
| EE\_2 | <--- | EOE\_5 | 6.371 | .042 |
| EE\_2 | <--- | EOE\_2 | 5.647 | .040 |
| EE\_2 | <--- | AISE\_3 | 5.185 | -.027 |
| EE\_1 | <--- | ESE\_1 | 6.885 | .041 |
| ESE\_10 | <--- | AISE | 4.672 | .034 |
| ESE\_10 | <--- | Vc | 4.066 | .044 |
| ESE\_10 | <--- | EE\_16 | 6.509 | .039 |
| ESE\_10 | <--- | EE\_14 | 5.755 | .037 |
| ESE\_10 | <--- | ESE\_6 | 4.831 | .033 |
| ESE\_10 | <--- | AISE\_6 | 8.509 | .033 |
| ESE\_10 | <--- | AISE\_2 | 4.043 | .023 |
| ESE\_10 | <--- | AISE\_1 | 7.538 | .030 |
| ESE\_8 | <--- | EE\_12 | 7.278 | .041 |
| ESE\_8 | <--- | EE\_3 | 5.836 | .036 |
| ESE\_8 | <--- | EOE\_5 | 4.656 | -.035 |
| ESE\_7 | <--- | AISE | 5.882 | -.038 |
| ESE\_7 | <--- | AISE\_6 | 11.550 | -.038 |
| ESE\_7 | <--- | AISE\_3 | 4.759 | -.025 |
| ESE\_7 | <--- | AISE\_2 | 5.481 | -.027 |
| ESE\_5 | <--- | ESE\_8 | 4.424 | .032 |
| ESE\_3 | <--- | EOE\_9 | 4.501 | -.035 |
| ESE\_1 | <--- | EE\_20 | 4.361 | -.034 |
| ESE\_1 | <--- | EE\_2 | 5.431 | -.036 |
| EOE\_9 | <--- | ESE\_3 | 6.144 | -.037 |
| EOE\_9 | <--- | EOE\_4 | 4.545 | -.033 |
| EOE\_8 | <--- | ESE\_3 | 4.590 | .031 |
| EOE\_7 | <--- | EE\_4 | 6.152 | .034 |
| EOE\_6 | <--- | EE\_2 | 4.082 | -.029 |
| EOE\_5 | <--- | EOE\_3 | 4.054 | -.030 |
| EOE\_4 | <--- | EE\_5 | 4.136 | .028 |
| EOE\_4 | <--- | EE\_4 | 5.731 | .034 |
| EOE\_4 | <--- | EOE\_9 | 4.634 | -.032 |
| EOE\_4 | <--- | AISE\_1 | 4.977 | .022 |
| EOE\_2 | <--- | EE\_3 | 5.569 | -.033 |
| AISE\_6 | <--- | EOE\_7 | 4.698 | -.047 |
| AISE\_5 | <--- | AISE\_1 | 4.007 | -.027 |
| AISE\_4 | <--- | EE\_16 | 7.496 | -.056 |
| AISE\_3 | <--- | EE\_2 | 5.057 | -.045 |

## Minimization History (Default model)

| Iteration |  | Negative eigenvalues | Condition # | Smallest eigenvalue | Diameter | F | NTries | Ratio |
| --- | --- | --- | --- | --- | --- | --- | --- | --- |
| 0 | e | 20 |  | -1.098 | 9999.000 | 48874.120 | 0 | 9999.000 |
| 1 | e | 14 |  | -.130 | 4.669 | 21688.648 | 20 | .306 |
| 2 | e\* | 5 |  | -.132 | 2.467 | 7343.431 | 4 | .862 |
| 3 | e | 1 |  | -.020 | 1.267 | 3233.829 | 5 | .893 |
| 4 | e | 0 | 402.099 |  | .873 | 1788.873 | 5 | .848 |
| 5 | e | 0 | 109.929 |  | 1.091 | 1453.383 | 3 | .000 |
| 6 | e | 0 | 148.374 |  | .573 | 1071.796 | 1 | 1.124 |
| 7 | e | 0 | 163.992 |  | .256 | 1037.043 | 1 | 1.090 |
| 8 | e | 0 | 180.627 |  | .053 | 1035.779 | 1 | 1.041 |
| 9 | e | 0 | 179.544 |  | .004 | 1035.773 | 1 | 1.004 |
| 10 | e | 0 | 179.280 |  | .000 | 1035.773 | 1 | 1.001 |

## Model Fit Summary

## CMIN

| Model | NPAR | CMIN | DF | P | CMIN/DF |
| --- | --- | --- | --- | --- | --- |
| Default model | 102 | 1035.773 | 933 | .010 | 1.110 |
| Saturated model | 1035 | .000 | 0 |  |  |
| Independence model | 45 | 49963.739 | 990 | .000 | 50.468 |

## RMR, GFI

| Model | RMR | GFI | AGFI | PGFI |
| --- | --- | --- | --- | --- |
| Default model | .010 | .980 | .978 | .884 |
| Saturated model | .000 | 1.000 |  |  |
| Independence model | .190 | .204 | .168 | .195 |

## Baseline Comparisons

| Model | NFI Delta1 | RFI rho1 | IFI Delta2 | TLI rho2 | CFI |
| --- | --- | --- | --- | --- | --- |
| Default model | .979 | .978 | .998 | .998 | .998 |
| Saturated model | 1.000 |  | 1.000 |  | 1.000 |
| Independence model | .000 | .000 | .000 | .000 | .000 |

## Parsimony-Adjusted Measures

| Model | PRATIO | PNFI | PCFI |
| --- | --- | --- | --- |
| Default model | .942 | .923 | .940 |
| Saturated model | .000 | .000 | .000 |
| Independence model | 1.000 | .000 | .000 |

## NCP

| Model | NCP | LO 90 | HI 90 |
| --- | --- | --- | --- |
| Default model | 102.773 | 28.433 | 185.451 |
| Saturated model | .000 | .000 | .000 |
| Independence model | 48973.739 | 48245.220 | 49708.577 |

## FMIN

| Model | FMIN | F0 | LO 90 | HI 90 |
| --- | --- | --- | --- | --- |
| Default model | .455 | .045 | .012 | .081 |
| Saturated model | .000 | .000 | .000 | .000 |
| Independence model | 21.933 | 21.499 | 21.179 | 21.821 |

## RMSEA

| Model | RMSEA | LO 90 | HI 90 | PCLOSE |
| --- | --- | --- | --- | --- |
| Default model | .007 | .004 | .009 | 1.000 |
| Independence model | .147 | .146 | .148 | .000 |

## AIC

| Model | AIC | BCC | BIC | CAIC |
| --- | --- | --- | --- | --- |
| Default model | 1239.773 | 1243.977 | 1824.385 | 1926.385 |
| Saturated model | 2070.000 | 2112.661 | 8002.094 | 9037.094 |
| Independence model | 50053.739 | 50055.594 | 50311.656 | 50356.656 |

## ECVI

| Model | ECVI | LO 90 | HI 90 | MECVI |
| --- | --- | --- | --- | --- |
| Default model | .544 | .512 | .581 | .546 |
| Saturated model | .909 | .909 | .909 | .927 |
| Independence model | 21.973 | 21.653 | 22.295 | 21.973 |

## HOELTER

| Model | HOELTER .05 | HOELTER .01 |
| --- | --- | --- |
| Default model | 2211 | 2280 |
| Independence model | 49 | 50 |

## Execution time summary

|  |  |
| --- | --- |
| Minimization: | .074 |
| Miscellaneous: | 1.001 |
| Bootstrap: | .000 |
| Total: | 1.075 |
