# Supplementary material for: AI-contextualized self-efficacy and exercise engagement among vocational college students in China: a cross-sectional serial mediation analysis of exercise self-efficacy and exercise outcome expectations
Source: Front Psychol. 2026 Oct 6;17:1933716. doi: 10.3389/fpsyg.2026.1933716 (PMC13680253; doi:10.3389/fpsyg.2026.1933716)
Supplement: Supplementary file 2 [file Data_Sheet_1.zip › Revision_Package_1933716/CFA_Model_2/CFA_Model_2.AmosOutput]

验证性因子分析模型2.amw


# D:\系统默认\桌面\danzi\2025年9月份单子\361.数据分析-220元\验证性因子分析模型2.amw

## Analysis Summary

## Date and Time

Date: 2026年9月13日

Time: 18:03:31

## Title

验证性因子分析模型2: 2026年9月13日 18:03

## Groups

## Group number 1 (Group number 1)

## Notes for Group (Group number 1)

The model is recursive.

Sample size = 2279

## Variable Summary (Group number 1)

## Your model contains the following variables (Group number 1)

Observed, endogenous variables

EOE\_1

EOE\_2

EOE\_3

EOE\_4

EOE\_5

EOE\_6

EOE\_7

EOE\_8

EOE\_9

EE\_13

EE\_14

EE\_15

EE\_16

EE\_17

Unobserved, exogenous variables

EOE

e1

e2

e3

e4

e5

e6

e7

e8

e9

ValueCognition

e10

e11

e12

e13

e14

## Variable counts (Group number 1)

|  |  |
| --- | --- |
| Number of variables in your model: | 30 |
| Number of observed variables: | 14 |
| Number of unobserved variables: | 16 |
| Number of exogenous variables: | 16 |
| Number of endogenous variables: | 14 |

## Parameter Summary (Group number 1)

|  | Weights | Covariances | Variances | Means | Intercepts | Total |
| --- | --- | --- | --- | --- | --- | --- |
| Fixed | 16 | 0 | 0 | 0 | 0 | 16 |
| Labeled | 0 | 0 | 0 | 0 | 0 | 0 |
| Unlabeled | 12 | 1 | 16 | 0 | 0 | 29 |
| Total | 28 | 1 | 16 | 0 | 0 | 45 |

## Models

## Default model (Default model)

## Notes for Model (Default model)

## Computation of degrees of freedom (Default model)

|  |  |
| --- | --- |
| Number of distinct sample moments: | 105 |
| Number of distinct parameters to be estimated: | 29 |
| Degrees of freedom (105 - 29): | 76 |

## Result (Default model)

Minimum was achieved

Chi-square = 119.346

Degrees of freedom = 76

Probability level = .001

## Group number 1 (Group number 1 - Default model)

## Estimates (Group number 1 - Default model)

## Scalar Estimates (Group number 1 - Default model)

## Maximum Likelihood Estimates

## Regression Weights: (Group number 1 - Default model)

|  |  |  | Estimate | S.E. | C.R. | P | Label |
| --- | --- | --- | --- | --- | --- | --- | --- |
| EOE\_1 | <--- | EOE | 1.000 |  |  |  |  |
| EOE\_2 | <--- | EOE | 1.014 | .030 | 33.412 | \*\*\* |  |
| EOE\_3 | <--- | EOE | 1.002 | .030 | 33.100 | \*\*\* |  |
| EOE\_4 | <--- | EOE | .989 | .030 | 33.073 | \*\*\* |  |
| EOE\_5 | <--- | EOE | 1.031 | .030 | 33.843 | \*\*\* |  |
| EOE\_6 | <--- | EOE | .994 | .030 | 32.938 | \*\*\* |  |
| EOE\_7 | <--- | EOE | 1.030 | .030 | 34.217 | \*\*\* |  |
| EOE\_8 | <--- | EOE | 1.023 | .031 | 33.365 | \*\*\* |  |
| EOE\_9 | <--- | EOE | 1.001 | .030 | 32.947 | \*\*\* |  |
| EE\_13 | <--- | ValueCognition | 1.000 |  |  |  |  |
| EE\_14 | <--- | ValueCognition | .979 | .030 | 33.168 | \*\*\* |  |
| EE\_15 | <--- | ValueCognition | .991 | .029 | 33.676 | \*\*\* |  |
| EE\_16 | <--- | ValueCognition | .983 | .030 | 33.239 | \*\*\* |  |
| EE\_17 | <--- | ValueCognition | .977 | .029 | 33.391 | \*\*\* |  |

## Standardized Regression Weights: (Group number 1 - Default model)

|  |  |  | Estimate |
| --- | --- | --- | --- |
| EOE\_1 | <--- | EOE | .720 |
| EOE\_2 | <--- | EOE | .728 |
| EOE\_3 | <--- | EOE | .721 |
| EOE\_4 | <--- | EOE | .721 |
| EOE\_5 | <--- | EOE | .738 |
| EOE\_6 | <--- | EOE | .718 |
| EOE\_7 | <--- | EOE | .746 |
| EOE\_8 | <--- | EOE | .727 |
| EOE\_9 | <--- | EOE | .718 |
| EE\_13 | <--- | ValueCognition | .747 |
| EE\_14 | <--- | ValueCognition | .735 |
| EE\_15 | <--- | ValueCognition | .747 |
| EE\_16 | <--- | ValueCognition | .737 |
| EE\_17 | <--- | ValueCognition | .740 |

## Covariances: (Group number 1 - Default model)

|  |  |  | Estimate | S.E. | C.R. | P | Label |
| --- | --- | --- | --- | --- | --- | --- | --- |
| EOE | <--> | ValueCognition | .117 | .008 | 14.261 | \*\*\* |  |

## Correlations: (Group number 1 - Default model)

|  |  |  | Estimate |
| --- | --- | --- | --- |
| EOE | <--> | ValueCognition | .389 |

## Variances: (Group number 1 - Default model)

|  |  |  | Estimate | S.E. | C.R. | P | Label |
| --- | --- | --- | --- | --- | --- | --- | --- |
| EOE |  |  | .275 | .014 | 19.250 | \*\*\* |  |
| ValueCognition |  |  | .331 | .017 | 19.638 | \*\*\* |  |
| e1 |  |  | .255 | .008 | 30.169 | \*\*\* |  |
| e2 |  |  | .250 | .008 | 29.995 | \*\*\* |  |
| e3 |  |  | .254 | .008 | 30.140 | \*\*\* |  |
| e4 |  |  | .248 | .008 | 30.152 | \*\*\* |  |
| e5 |  |  | .245 | .008 | 29.781 | \*\*\* |  |
| e6 |  |  | .255 | .008 | 30.213 | \*\*\* |  |
| e7 |  |  | .233 | .008 | 29.583 | \*\*\* |  |
| e8 |  |  | .256 | .009 | 30.017 | \*\*\* |  |
| e9 |  |  | .258 | .009 | 30.209 | \*\*\* |  |
| e10 |  |  | .262 | .010 | 26.776 | \*\*\* |  |
| e11 |  |  | .270 | .010 | 27.254 | \*\*\* |  |
| e12 |  |  | .258 | .010 | 26.781 | \*\*\* |  |
| e13 |  |  | .270 | .010 | 27.191 | \*\*\* |  |
| e14 |  |  | .261 | .010 | 27.053 | \*\*\* |  |

## Modification Indices (Group number 1 - Default model)

## Covariances: (Group number 1 - Default model)

|  |  |  | M.I. | Par Change |
| --- | --- | --- | --- | --- |
| e10 | <--> | e14 | 4.623 | -.014 |
| e10 | <--> | e11 | 4.141 | .013 |
| e5 | <--> | e9 | 4.982 | .013 |
| e5 | <--> | e6 | 5.562 | .014 |
| e4 | <--> | e14 | 4.006 | -.012 |
| e4 | <--> | e9 | 9.014 | -.018 |
| e4 | <--> | e6 | 5.675 | -.014 |
| e3 | <--> | e5 | 9.369 | -.018 |
| e3 | <--> | e4 | 6.997 | .015 |
| e2 | <--> | e6 | 5.862 | -.014 |
| e2 | <--> | e4 | 9.153 | .017 |
| e2 | <--> | e3 | 5.248 | .013 |
| e1 | <--> | e5 | 5.684 | -.014 |
| e1 | <--> | e2 | 5.166 | .013 |

## Variances: (Group number 1 - Default model)

|  |  |  | M.I. | Par Change |
| --- | --- | --- | --- | --- |

## Regression Weights: (Group number 1 - Default model)

|  |  |  | M.I. | Par Change |
| --- | --- | --- | --- | --- |
| EOE\_5 | <--- | EOE\_3 | 4.109 | -.031 |
| EOE\_2 | <--- | EOE\_4 | 4.020 | .031 |

## Minimization History (Default model)

| Iteration |  | Negative eigenvalues | Condition # | Smallest eigenvalue | Diameter | F | NTries | Ratio |
| --- | --- | --- | --- | --- | --- | --- | --- | --- |
| 0 | e | 4 |  | -1.064 | 9999.000 | 14073.838 | 0 | 9999.000 |
| 1 | e | 2 |  | -.094 | 3.691 | 4952.889 | 20 | .178 |
| 2 | e | 1 |  | -.067 | 1.717 | 1676.243 | 5 | .607 |
| 3 | e | 0 | 63.497 |  | .760 | 460.207 | 5 | .969 |
| 4 | e | 0 | 40.644 |  | .429 | 231.281 | 2 | .000 |
| 5 | e | 0 | 49.487 |  | .171 | 129.071 | 1 | 1.154 |
| 6 | e | 0 | 52.800 |  | .060 | 119.537 | 1 | 1.086 |
| 7 | e | 0 | 52.776 |  | .008 | 119.346 | 1 | 1.016 |
| 8 | e | 0 | 52.776 |  | .000 | 119.346 | 1 | 1.000 |

## Model Fit Summary

## CMIN

| Model | NPAR | CMIN | DF | P | CMIN/DF |
| --- | --- | --- | --- | --- | --- |
| Default model | 29 | 119.346 | 76 | .001 | 1.570 |
| Saturated model | 105 | .000 | 0 |  |  |
| Independence model | 14 | 14934.693 | 91 | .000 | 164.118 |

## RMR, GFI

| Model | RMR | GFI | AGFI | PGFI |
| --- | --- | --- | --- | --- |
| Default model | .006 | .992 | .989 | .718 |
| Saturated model | .000 | 1.000 |  |  |
| Independence model | .206 | .318 | .213 | .275 |

## Baseline Comparisons

| Model | NFI Delta1 | RFI rho1 | IFI Delta2 | TLI rho2 | CFI |
| --- | --- | --- | --- | --- | --- |
| Default model | .992 | .990 | .997 | .997 | .997 |
| Saturated model | 1.000 |  | 1.000 |  | 1.000 |
| Independence model | .000 | .000 | .000 | .000 | .000 |

## Parsimony-Adjusted Measures

| Model | PRATIO | PNFI | PCFI |
| --- | --- | --- | --- |
| Default model | .835 | .828 | .833 |
| Saturated model | .000 | .000 | .000 |
| Independence model | 1.000 | .000 | .000 |

## NCP

| Model | NCP | LO 90 | HI 90 |
| --- | --- | --- | --- |
| Default model | 43.346 | 17.575 | 77.050 |
| Saturated model | .000 | .000 | .000 |
| Independence model | 14843.693 | 14445.430 | 15248.244 |

## FMIN

| Model | FMIN | F0 | LO 90 | HI 90 |
| --- | --- | --- | --- | --- |
| Default model | .052 | .019 | .008 | .034 |
| Saturated model | .000 | .000 | .000 | .000 |
| Independence model | 6.556 | 6.516 | 6.341 | 6.694 |

## RMSEA

| Model | RMSEA | LO 90 | HI 90 | PCLOSE |
| --- | --- | --- | --- | --- |
| Default model | .016 | .010 | .021 | 1.000 |
| Independence model | .268 | .264 | .271 | .000 |

## AIC

| Model | AIC | BCC | BIC | CAIC |
| --- | --- | --- | --- | --- |
| Default model | 177.346 | 177.731 | 343.559 | 372.559 |
| Saturated model | 210.000 | 211.392 | 811.807 | 916.807 |
| Independence model | 14962.693 | 14962.879 | 15042.934 | 15056.934 |

## ECVI

| Model | ECVI | LO 90 | HI 90 | MECVI |
| --- | --- | --- | --- | --- |
| Default model | .078 | .067 | .093 | .078 |
| Saturated model | .092 | .092 | .092 | .093 |
| Independence model | 6.568 | 6.394 | 6.746 | 6.568 |

## HOELTER

| Model | HOELTER .05 | HOELTER .01 |
| --- | --- | --- |
| Default model | 1859 | 2054 |
| Independence model | 18 | 20 |

## Execution time summary

|  |  |
| --- | --- |
| Minimization: | .024 |
| Miscellaneous: | .187 |
| Bootstrap: | .000 |
| Total: | .211 |
