# Supplementary material for: AI-contextualized self-efficacy and exercise engagement among vocational college students in China: a cross-sectional serial mediation analysis of exercise self-efficacy and exercise outcome expectations
Source: Front Psychol. 2026 Oct 6;17:1933716. doi: 10.3389/fpsyg.2026.1933716 (PMC13680253; doi:10.3389/fpsyg.2026.1933716)
Supplement: Supplementary file 2 [file Data_Sheet_1.zip › Revision_Package_1933716/Single_Factor_Model_1/Single_Factor_Model_1.AmosOutput]

单因子模型.amw


# D:\系统默认\桌面\danzi\2025年9月份单子\361.数据分析-220元\单因子模型.amw

## Analysis Summary

## Date and Time

Date: 2026年9月13日

Time: 12:45:00

## Title

单因子模型: 2026年9月13日 12:45

## Groups

## Group number 1 (Group number 1)

## Notes for Group (Group number 1)

The model is recursive.

Sample size = 2279

## Variable Summary (Group number 1)

## Your model contains the following variables (Group number 1)

Observed, endogenous variables

EE\_1

EE\_2

EE\_3

EE\_4

EE\_5

EE\_6

EE\_7

EE\_8

EE\_9

EE\_10

EE\_11

EE\_12

EE\_13

EE\_14

EE\_15

EE\_16

EE\_17

EE\_18

EE\_19

EE\_20

ESE\_1

ESE\_2

ESE\_3

ESE\_4

ESE\_5

ESE\_6

ESE\_7

ESE\_8

ESE\_9

ESE\_10

AISE\_1

AISE\_2

AISE\_3

AISE\_4

AISE\_5

AISE\_6

EOE\_1

EOE\_2

EOE\_3

EOE\_4

EOE\_5

EOE\_6

EOE\_7

EOE\_8

EOE\_9

Unobserved, exogenous variables

GEN

e1

e2

e3

e4

e5

e6

e7

e8

e9

e10

e11

e12

e13

e14

e15

e16

e17

e18

e19

e20

e21

e22

e23

e24

e25

e26

e27

e28

e29

e30

e31

e32

e33

e34

e35

e36

e37

e38

e39

e40

e41

e42

e43

e44

e45

## Variable counts (Group number 1)

|  |  |
| --- | --- |
| Number of variables in your model: | 91 |
| Number of observed variables: | 45 |
| Number of unobserved variables: | 46 |
| Number of exogenous variables: | 46 |
| Number of endogenous variables: | 45 |

## Parameter Summary (Group number 1)

|  | Weights | Covariances | Variances | Means | Intercepts | Total |
| --- | --- | --- | --- | --- | --- | --- |
| Fixed | 46 | 0 | 0 | 0 | 0 | 46 |
| Labeled | 0 | 0 | 0 | 0 | 0 | 0 |
| Unlabeled | 44 | 0 | 46 | 0 | 0 | 90 |
| Total | 90 | 0 | 46 | 0 | 0 | 136 |

## Models

## Default model (Default model)

## Notes for Model (Default model)

## Computation of degrees of freedom (Default model)

|  |  |
| --- | --- |
| Number of distinct sample moments: | 1035 |
| Number of distinct parameters to be estimated: | 90 |
| Degrees of freedom (1035 - 90): | 945 |

## Result (Default model)

Minimum was achieved

Chi-square = 23193.606

Degrees of freedom = 945

Probability level = .000

## Group number 1 (Group number 1 - Default model)

## Estimates (Group number 1 - Default model)

## Scalar Estimates (Group number 1 - Default model)

## Maximum Likelihood Estimates

## Regression Weights: (Group number 1 - Default model)

|  |  |  | Estimate | S.E. | C.R. | P | Label |
| --- | --- | --- | --- | --- | --- | --- | --- |
| EE\_1 | <--- | GEN | 1.000 |  |  |  |  |
| EE\_2 | <--- | GEN | 1.021 | .047 | 21.877 | \*\*\* |  |
| EE\_3 | <--- | GEN | 1.047 | .047 | 22.313 | \*\*\* |  |
| EE\_4 | <--- | GEN | .977 | .046 | 21.379 | \*\*\* |  |
| EE\_5 | <--- | GEN | .997 | .046 | 21.492 | \*\*\* |  |
| EE\_6 | <--- | GEN | 1.037 | .047 | 22.264 | \*\*\* |  |
| EE\_7 | <--- | GEN | 1.021 | .047 | 21.947 | \*\*\* |  |
| EE\_8 | <--- | GEN | .983 | .046 | 21.489 | \*\*\* |  |
| EE\_9 | <--- | GEN | 1.018 | .046 | 22.014 | \*\*\* |  |
| EE\_10 | <--- | GEN | 1.038 | .047 | 22.250 | \*\*\* |  |
| EE\_11 | <--- | GEN | .990 | .046 | 21.572 | \*\*\* |  |
| EE\_12 | <--- | GEN | 1.039 | .046 | 22.449 | \*\*\* |  |
| EE\_13 | <--- | GEN | 1.039 | .046 | 22.456 | \*\*\* |  |
| EE\_14 | <--- | GEN | 1.005 | .046 | 22.025 | \*\*\* |  |
| EE\_15 | <--- | GEN | 1.010 | .046 | 22.151 | \*\*\* |  |
| EE\_16 | <--- | GEN | .989 | .046 | 21.727 | \*\*\* |  |
| EE\_17 | <--- | GEN | .988 | .045 | 21.893 | \*\*\* |  |
| EE\_18 | <--- | GEN | .907 | .043 | 20.964 | \*\*\* |  |
| EE\_19 | <--- | GEN | .918 | .044 | 21.076 | \*\*\* |  |
| EE\_20 | <--- | GEN | .898 | .043 | 20.650 | \*\*\* |  |
| ESE\_1 | <--- | GEN | .794 | .043 | 18.273 | \*\*\* |  |
| ESE\_2 | <--- | GEN | .819 | .044 | 18.774 | \*\*\* |  |
| ESE\_3 | <--- | GEN | .779 | .043 | 18.292 | \*\*\* |  |
| ESE\_4 | <--- | GEN | .836 | .044 | 18.936 | \*\*\* |  |
| ESE\_5 | <--- | GEN | .825 | .044 | 18.928 | \*\*\* |  |
| ESE\_6 | <--- | GEN | .833 | .044 | 18.762 | \*\*\* |  |
| ESE\_7 | <--- | GEN | .749 | .042 | 17.693 | \*\*\* |  |
| ESE\_8 | <--- | GEN | .805 | .043 | 18.732 | \*\*\* |  |
| ESE\_9 | <--- | GEN | .746 | .042 | 17.881 | \*\*\* |  |
| ESE\_10 | <--- | GEN | .813 | .043 | 18.991 | \*\*\* |  |
| AISE\_1 | <--- | GEN | 1.338 | .064 | 20.912 | \*\*\* |  |
| AISE\_2 | <--- | GEN | 1.306 | .061 | 21.559 | \*\*\* |  |
| AISE\_3 | <--- | GEN | 1.290 | .061 | 21.314 | \*\*\* |  |
| AISE\_4 | <--- | GEN | 1.332 | .062 | 21.544 | \*\*\* |  |
| AISE\_5 | <--- | GEN | 1.284 | .059 | 21.827 | \*\*\* |  |
| AISE\_6 | <--- | GEN | 1.323 | .062 | 21.420 | \*\*\* |  |
| EOE\_1 | <--- | GEN | .899 | .043 | 21.082 | \*\*\* |  |
| EOE\_2 | <--- | GEN | .904 | .043 | 21.119 | \*\*\* |  |
| EOE\_3 | <--- | GEN | .902 | .043 | 21.123 | \*\*\* |  |
| EOE\_4 | <--- | GEN | .924 | .043 | 21.702 | \*\*\* |  |
| EOE\_5 | <--- | GEN | .923 | .043 | 21.385 | \*\*\* |  |
| EOE\_6 | <--- | GEN | .876 | .042 | 20.739 | \*\*\* |  |
| EOE\_7 | <--- | GEN | .937 | .043 | 21.812 | \*\*\* |  |
| EOE\_8 | <--- | GEN | .912 | .043 | 21.107 | \*\*\* |  |
| EOE\_9 | <--- | GEN | .906 | .043 | 21.151 | \*\*\* |  |

## Standardized Regression Weights: (Group number 1 - Default model)

|  |  |  | Estimate |
| --- | --- | --- | --- |
| EE\_1 | <--- | GEN | .554 |
| EE\_2 | <--- | GEN | .556 |
| EE\_3 | <--- | GEN | .572 |
| EE\_4 | <--- | GEN | .539 |
| EE\_5 | <--- | GEN | .543 |
| EE\_6 | <--- | GEN | .570 |
| EE\_7 | <--- | GEN | .559 |
| EE\_8 | <--- | GEN | .543 |
| EE\_9 | <--- | GEN | .561 |
| EE\_10 | <--- | GEN | .570 |
| EE\_11 | <--- | GEN | .546 |
| EE\_12 | <--- | GEN | .577 |
| EE\_13 | <--- | GEN | .577 |
| EE\_14 | <--- | GEN | .562 |
| EE\_15 | <--- | GEN | .566 |
| EE\_16 | <--- | GEN | .551 |
| EE\_17 | <--- | GEN | .557 |
| EE\_18 | <--- | GEN | .525 |
| EE\_19 | <--- | GEN | .528 |
| EE\_20 | <--- | GEN | .514 |
| ESE\_1 | <--- | GEN | .439 |
| ESE\_2 | <--- | GEN | .454 |
| ESE\_3 | <--- | GEN | .440 |
| ESE\_4 | <--- | GEN | .459 |
| ESE\_5 | <--- | GEN | .459 |
| ESE\_6 | <--- | GEN | .454 |
| ESE\_7 | <--- | GEN | .422 |
| ESE\_8 | <--- | GEN | .453 |
| ESE\_9 | <--- | GEN | .428 |
| ESE\_10 | <--- | GEN | .461 |
| AISE\_1 | <--- | GEN | .523 |
| AISE\_2 | <--- | GEN | .545 |
| AISE\_3 | <--- | GEN | .537 |
| AISE\_4 | <--- | GEN | .545 |
| AISE\_5 | <--- | GEN | .554 |
| AISE\_6 | <--- | GEN | .540 |
| EOE\_1 | <--- | GEN | .529 |
| EOE\_2 | <--- | GEN | .530 |
| EOE\_3 | <--- | GEN | .530 |
| EOE\_4 | <--- | GEN | .550 |
| EOE\_5 | <--- | GEN | .539 |
| EOE\_6 | <--- | GEN | .517 |
| EOE\_7 | <--- | GEN | .554 |
| EOE\_8 | <--- | GEN | .530 |
| EOE\_9 | <--- | GEN | .531 |

## Variances: (Group number 1 - Default model)

|  |  |  | Estimate | S.E. | C.R. | P | Label |
| --- | --- | --- | --- | --- | --- | --- | --- |
| GEN |  |  | .183 | .013 | 14.035 | \*\*\* |  |
| e1 |  |  | .414 | .013 | 32.892 | \*\*\* |  |
| e2 |  |  | .426 | .013 | 32.882 | \*\*\* |  |
| e3 |  |  | .413 | .013 | 32.809 | \*\*\* |  |
| e4 |  |  | .427 | .013 | 32.957 | \*\*\* |  |
| e5 |  |  | .436 | .013 | 32.941 | \*\*\* |  |
| e6 |  |  | .409 | .012 | 32.817 | \*\*\* |  |
| e7 |  |  | .421 | .013 | 32.871 | \*\*\* |  |
| e8 |  |  | .424 | .013 | 32.942 | \*\*\* |  |
| e9 |  |  | .413 | .013 | 32.860 | \*\*\* |  |
| e10 |  |  | .411 | .013 | 32.820 | \*\*\* |  |
| e11 |  |  | .424 | .013 | 32.929 | \*\*\* |  |
| e12 |  |  | .397 | .012 | 32.784 | \*\*\* |  |
| e13 |  |  | .396 | .012 | 32.783 | \*\*\* |  |
| e14 |  |  | .402 | .012 | 32.858 | \*\*\* |  |
| e15 |  |  | .396 | .012 | 32.837 | \*\*\* |  |
| e16 |  |  | .411 | .012 | 32.906 | \*\*\* |  |
| e17 |  |  | .398 | .012 | 32.880 | \*\*\* |  |
| e18 |  |  | .397 | .012 | 33.014 | \*\*\* |  |
| e19 |  |  | .398 | .012 | 32.999 | \*\*\* |  |
| e20 |  |  | .411 | .012 | 33.054 | \*\*\* |  |
| e21 |  |  | .483 | .015 | 33.287 | \*\*\* |  |
| e22 |  |  | .472 | .014 | 33.246 | \*\*\* |  |
| e23 |  |  | .464 | .014 | 33.285 | \*\*\* |  |
| e24 |  |  | .479 | .014 | 33.232 | \*\*\* |  |
| e25 |  |  | .467 | .014 | 33.232 | \*\*\* |  |
| e26 |  |  | .489 | .015 | 33.247 | \*\*\* |  |
| e27 |  |  | .474 | .014 | 33.330 | \*\*\* |  |
| e28 |  |  | .460 | .014 | 33.249 | \*\*\* |  |
| e29 |  |  | .456 | .014 | 33.316 | \*\*\* |  |
| e30 |  |  | .449 | .014 | 33.227 | \*\*\* |  |
| e31 |  |  | .871 | .026 | 33.021 | \*\*\* |  |
| e32 |  |  | .739 | .022 | 32.931 | \*\*\* |  |
| e33 |  |  | .754 | .023 | 32.967 | \*\*\* |  |
| e34 |  |  | .771 | .023 | 32.933 | \*\*\* |  |
| e35 |  |  | .680 | .021 | 32.890 | \*\*\* |  |
| e36 |  |  | .778 | .024 | 32.952 | \*\*\* |  |
| e37 |  |  | .382 | .012 | 32.999 | \*\*\* |  |
| e38 |  |  | .383 | .012 | 32.994 | \*\*\* |  |
| e39 |  |  | .381 | .012 | 32.993 | \*\*\* |  |
| e40 |  |  | .361 | .011 | 32.910 | \*\*\* |  |
| e41 |  |  | .381 | .012 | 32.957 | \*\*\* |  |
| e42 |  |  | .385 | .012 | 33.043 | \*\*\* |  |
| e43 |  |  | .363 | .011 | 32.892 | \*\*\* |  |
| e44 |  |  | .391 | .012 | 32.995 | \*\*\* |  |
| e45 |  |  | .383 | .012 | 32.989 | \*\*\* |  |

## Modification Indices (Group number 1 - Default model)

## Covariances: (Group number 1 - Default model)

|  |  |  | M.I. | Par Change |
| --- | --- | --- | --- | --- |
| e44 | <--> | e45 | 266.338 | .135 |
| e43 | <--> | e45 | 315.830 | .142 |
| e43 | <--> | e44 | 315.398 | .143 |
| e42 | <--> | e45 | 301.189 | .143 |
| e42 | <--> | e44 | 287.951 | .141 |
| e42 | <--> | e43 | 326.505 | .145 |
| e41 | <--> | e45 | 323.865 | .147 |
| e41 | <--> | e44 | 317.438 | .147 |
| e41 | <--> | e43 | 313.502 | .141 |
| e41 | <--> | e42 | 337.710 | .151 |
| e40 | <--> | e45 | 186.569 | .109 |
| e40 | <--> | e44 | 244.445 | .126 |
| e40 | <--> | e43 | 248.743 | .122 |
| e40 | <--> | e42 | 207.743 | .115 |
| e40 | <--> | e41 | 273.367 | .131 |
| e39 | <--> | e45 | 255.515 | .131 |
| e39 | <--> | e44 | 255.646 | .132 |
| e39 | <--> | e43 | 248.416 | .126 |
| e39 | <--> | e42 | 251.924 | .130 |
| e39 | <--> | e41 | 224.246 | .122 |
| e39 | <--> | e40 | 303.328 | .138 |
| e38 | <--> | e45 | 232.384 | .125 |
| e38 | <--> | e44 | 258.972 | .133 |
| e38 | <--> | e43 | 276.889 | .133 |
| e38 | <--> | e42 | 231.787 | .125 |
| e38 | <--> | e41 | 277.021 | .136 |
| e38 | <--> | e40 | 322.954 | .143 |
| e38 | <--> | e39 | 323.089 | .147 |
| e37 | <--> | e45 | 235.470 | .126 |
| e37 | <--> | e44 | 282.182 | .139 |
| e37 | <--> | e43 | 259.698 | .129 |
| e37 | <--> | e42 | 250.837 | .130 |
| e37 | <--> | e41 | 235.487 | .125 |
| e37 | <--> | e40 | 267.387 | .130 |
| e37 | <--> | e39 | 301.561 | .142 |
| e37 | <--> | e38 | 321.456 | .147 |
| e35 | <--> | e36 | 416.579 | .319 |
| e34 | <--> | e41 | 4.365 | .024 |
| e34 | <--> | e36 | 333.704 | .303 |
| e34 | <--> | e35 | 352.803 | .292 |
| e33 | <--> | e45 | 4.601 | .025 |
| e33 | <--> | e43 | 5.457 | .026 |
| e33 | <--> | e36 | 294.508 | .282 |
| e33 | <--> | e35 | 294.647 | .264 |
| e33 | <--> | e34 | 337.332 | .300 |
| e32 | <--> | e36 | 343.340 | .301 |
| e32 | <--> | e35 | 398.228 | .304 |
| e32 | <--> | e34 | 322.672 | .291 |
| e32 | <--> | e33 | 346.709 | .298 |
| e31 | <--> | e36 | 332.023 | .321 |
| e31 | <--> | e35 | 256.621 | .264 |
| e31 | <--> | e34 | 307.978 | .308 |
| e31 | <--> | e33 | 316.030 | .308 |
| e31 | <--> | e32 | 325.535 | .310 |
| e30 | <--> | e43 | 5.891 | -.021 |
| e30 | <--> | e41 | 5.483 | -.021 |
| e30 | <--> | e39 | 5.435 | -.021 |
| e30 | <--> | e36 | 11.007 | .042 |
| e30 | <--> | e33 | 4.185 | .025 |
| e30 | <--> | e32 | 4.204 | .025 |
| e30 | <--> | e31 | 10.860 | .044 |
| e29 | <--> | e30 | 371.137 | .185 |
| e28 | <--> | e43 | 4.080 | -.018 |
| e28 | <--> | e42 | 7.405 | -.024 |
| e28 | <--> | e41 | 11.061 | -.030 |
| e28 | <--> | e39 | 7.033 | -.024 |
| e28 | <--> | e36 | 7.496 | .035 |
| e28 | <--> | e31 | 7.200 | .036 |
| e28 | <--> | e30 | 421.088 | .198 |
| e28 | <--> | e29 | 435.107 | .203 |
| e27 | <--> | e30 | 444.132 | .206 |
| e27 | <--> | e29 | 455.091 | .210 |
| e27 | <--> | e28 | 481.031 | .217 |
| e26 | <--> | e30 | 93.766 | .096 |
| e26 | <--> | e29 | 59.751 | .078 |
| e26 | <--> | e28 | 60.366 | .078 |
| e26 | <--> | e27 | 75.774 | .089 |
| e25 | <--> | e43 | 4.150 | -.018 |
| e25 | <--> | e36 | 6.502 | .033 |
| e25 | <--> | e33 | 6.455 | .032 |
| e25 | <--> | e30 | 87.464 | .091 |
| e25 | <--> | e29 | 53.378 | .072 |
| e25 | <--> | e28 | 103.975 | .100 |
| e25 | <--> | e27 | 102.198 | .101 |
| e25 | <--> | e26 | 387.224 | .200 |
| e24 | <--> | e30 | 96.032 | .097 |
| e24 | <--> | e29 | 67.130 | .081 |
| e24 | <--> | e28 | 66.958 | .082 |
| e24 | <--> | e27 | 77.684 | .089 |
| e24 | <--> | e26 | 376.819 | .200 |
| e24 | <--> | e25 | 403.567 | .202 |
| e23 | <--> | e45 | 5.847 | -.022 |
| e23 | <--> | e30 | 53.851 | .071 |
| e23 | <--> | e29 | 54.274 | .072 |
| e23 | <--> | e28 | 57.831 | .075 |
| e23 | <--> | e27 | 80.240 | .089 |
| e23 | <--> | e26 | 368.194 | .194 |
| e23 | <--> | e25 | 395.794 | .197 |
| e23 | <--> | e24 | 425.756 | .207 |
| e22 | <--> | e43 | 5.915 | -.022 |
| e22 | <--> | e30 | 70.640 | .082 |
| e22 | <--> | e29 | 71.808 | .083 |
| e22 | <--> | e28 | 77.412 | .087 |
| e22 | <--> | e27 | 94.335 | .098 |
| e22 | <--> | e26 | 407.244 | .206 |
| e22 | <--> | e25 | 422.366 | .205 |
| e22 | <--> | e24 | 398.822 | .202 |
| e22 | <--> | e23 | 360.939 | .189 |
| e21 | <--> | e36 | 5.033 | .029 |
| e21 | <--> | e31 | 4.511 | .029 |
| e21 | <--> | e30 | 82.274 | .090 |
| e21 | <--> | e29 | 49.908 | .070 |
| e21 | <--> | e28 | 44.859 | .067 |
| e21 | <--> | e27 | 75.289 | .088 |
| e21 | <--> | e26 | 342.434 | .191 |
| e21 | <--> | e25 | 384.115 | .198 |
| e21 | <--> | e24 | 410.892 | .207 |
| e21 | <--> | e23 | 338.552 | .185 |
| e21 | <--> | e22 | 391.612 | .201 |
| e20 | <--> | e45 | 13.720 | -.031 |
| e20 | <--> | e44 | 30.981 | -.048 |
| e20 | <--> | e43 | 21.455 | -.038 |
| e20 | <--> | e42 | 30.787 | -.047 |
| e20 | <--> | e41 | 29.859 | -.046 |
| e20 | <--> | e40 | 22.504 | -.039 |
| e20 | <--> | e39 | 23.431 | -.041 |
| e20 | <--> | e38 | 17.240 | -.035 |
| e20 | <--> | e37 | 35.854 | -.051 |
| e20 | <--> | e36 | 10.616 | -.039 |
| e20 | <--> | e35 | 17.631 | -.048 |
| e20 | <--> | e34 | 13.090 | -.044 |
| e20 | <--> | e33 | 20.643 | -.054 |
| e20 | <--> | e32 | 14.849 | -.045 |
| e20 | <--> | e31 | 15.761 | -.051 |
| e20 | <--> | e30 | 18.077 | -.039 |
| e20 | <--> | e28 | 12.199 | -.032 |
| e20 | <--> | e27 | 14.397 | -.036 |
| e20 | <--> | e26 | 24.555 | -.047 |
| e20 | <--> | e25 | 26.318 | -.048 |
| e20 | <--> | e24 | 25.360 | -.048 |
| e20 | <--> | e23 | 11.472 | -.031 |
| e20 | <--> | e22 | 22.176 | -.044 |
| e20 | <--> | e21 | 39.520 | -.060 |
| e19 | <--> | e45 | 9.093 | -.025 |
| e19 | <--> | e44 | 20.115 | -.038 |
| e19 | <--> | e43 | 16.048 | -.033 |
| e19 | <--> | e42 | 9.485 | -.026 |
| e19 | <--> | e41 | 10.704 | -.027 |
| e19 | <--> | e40 | 14.676 | -.031 |
| e19 | <--> | e39 | 26.538 | -.043 |
| e19 | <--> | e38 | 16.149 | -.034 |
| e19 | <--> | e37 | 19.829 | -.037 |
| e19 | <--> | e36 | 15.297 | -.047 |
| e19 | <--> | e35 | 25.209 | -.056 |
| e19 | <--> | e34 | 13.139 | -.043 |
| e19 | <--> | e33 | 26.801 | -.061 |
| e19 | <--> | e32 | 14.561 | -.044 |
| e19 | <--> | e31 | 23.245 | -.061 |
| e19 | <--> | e30 | 16.278 | -.036 |
| e19 | <--> | e29 | 19.385 | -.040 |
| e19 | <--> | e28 | 14.147 | -.034 |
| e19 | <--> | e27 | 16.325 | -.037 |
| e19 | <--> | e26 | 16.843 | -.039 |
| e19 | <--> | e25 | 19.832 | -.041 |
| e19 | <--> | e24 | 25.068 | -.047 |
| e19 | <--> | e23 | 15.204 | -.036 |
| e19 | <--> | e22 | 10.503 | -.030 |
| e19 | <--> | e21 | 13.244 | -.034 |
| e19 | <--> | e20 | 266.944 | .141 |
| e18 | <--> | e45 | 21.654 | -.039 |
| e18 | <--> | e44 | 32.187 | -.048 |
| e18 | <--> | e43 | 26.308 | -.042 |
| e18 | <--> | e42 | 31.873 | -.047 |
| e18 | <--> | e41 | 26.334 | -.043 |
| e18 | <--> | e40 | 31.705 | -.046 |
| e18 | <--> | e39 | 34.546 | -.049 |
| e18 | <--> | e38 | 17.871 | -.035 |
| e18 | <--> | e37 | 36.606 | -.050 |
| e18 | <--> | e36 | 24.145 | -.058 |
| e18 | <--> | e35 | 29.555 | -.061 |
| e18 | <--> | e34 | 14.916 | -.046 |
| e18 | <--> | e33 | 25.310 | -.059 |
| e18 | <--> | e32 | 23.897 | -.057 |
| e18 | <--> | e31 | 16.852 | -.052 |
| e18 | <--> | e30 | 7.092 | -.024 |
| e18 | <--> | e29 | 11.952 | -.031 |
| e18 | <--> | e28 | 6.284 | -.023 |
| e18 | <--> | e27 | 17.208 | -.038 |
| e18 | <--> | e26 | 15.795 | -.037 |
| e18 | <--> | e25 | 22.767 | -.044 |
| e18 | <--> | e24 | 29.843 | -.051 |
| e18 | <--> | e23 | 27.776 | -.048 |
| e18 | <--> | e22 | 23.873 | -.045 |
| e18 | <--> | e21 | 28.268 | -.050 |
| e18 | <--> | e20 | 325.880 | .156 |
| e18 | <--> | e19 | 270.540 | .140 |
| e17 | <--> | e45 | 27.354 | -.044 |
| e17 | <--> | e44 | 33.895 | -.049 |
| e17 | <--> | e43 | 25.971 | -.042 |
| e17 | <--> | e42 | 40.391 | -.053 |
| e17 | <--> | e41 | 41.166 | -.054 |
| e17 | <--> | e40 | 64.934 | -.066 |
| e17 | <--> | e39 | 34.123 | -.049 |
| e17 | <--> | e38 | 29.588 | -.046 |
| e17 | <--> | e37 | 33.449 | -.048 |
| e17 | <--> | e36 | 20.023 | -.053 |
| e17 | <--> | e35 | 17.514 | -.047 |
| e17 | <--> | e34 | 41.420 | -.076 |
| e17 | <--> | e33 | 31.999 | -.066 |
| e17 | <--> | e32 | 37.640 | -.071 |
| e17 | <--> | e31 | 23.724 | -.061 |
| e17 | <--> | e30 | 20.451 | -.041 |
| e17 | <--> | e29 | 21.134 | -.042 |
| e17 | <--> | e28 | 17.993 | -.039 |
| e17 | <--> | e27 | 25.057 | -.046 |
| e17 | <--> | e26 | 26.794 | -.049 |
| e17 | <--> | e25 | 36.809 | -.056 |
| e17 | <--> | e24 | 33.414 | -.054 |
| e17 | <--> | e23 | 33.903 | -.053 |
| e17 | <--> | e22 | 29.170 | -.050 |
| e17 | <--> | e21 | 40.386 | -.059 |
| e17 | <--> | e20 | 20.850 | .040 |
| e17 | <--> | e19 | 8.046 | .024 |
| e17 | <--> | e18 | 15.375 | .033 |
| e16 | <--> | e45 | 41.781 | -.055 |
| e16 | <--> | e44 | 50.181 | -.061 |
| e16 | <--> | e43 | 47.699 | -.057 |
| e16 | <--> | e42 | 38.090 | -.053 |
| e16 | <--> | e41 | 33.563 | -.049 |
| e16 | <--> | e40 | 49.068 | -.058 |
| e16 | <--> | e39 | 32.306 | -.048 |
| e16 | <--> | e38 | 39.733 | -.054 |
| e16 | <--> | e37 | 41.641 | -.055 |
| e16 | <--> | e36 | 47.893 | -.084 |
| e16 | <--> | e35 | 32.395 | -.065 |
| e16 | <--> | e34 | 79.695 | -.108 |
| e16 | <--> | e33 | 34.672 | -.070 |
| e16 | <--> | e32 | 36.617 | -.072 |
| e16 | <--> | e31 | 42.309 | -.083 |
| e16 | <--> | e30 | 8.500 | -.027 |
| e16 | <--> | e29 | 18.378 | -.040 |
| e16 | <--> | e28 | 29.790 | -.051 |
| e16 | <--> | e27 | 15.272 | -.037 |
| e16 | <--> | e26 | 19.688 | -.042 |
| e16 | <--> | e25 | 15.032 | -.036 |
| e16 | <--> | e24 | 18.923 | -.041 |
| e16 | <--> | e23 | 16.159 | -.037 |
| e16 | <--> | e22 | 20.618 | -.043 |
| e16 | <--> | e21 | 22.804 | -.045 |
| e16 | <--> | e20 | 7.955 | .025 |
| e16 | <--> | e19 | 14.360 | .033 |
| e16 | <--> | e18 | 11.772 | .030 |
| e16 | <--> | e17 | 317.336 | .155 |
| e15 | <--> | e45 | 49.879 | -.059 |
| e15 | <--> | e44 | 40.812 | -.054 |
| e15 | <--> | e43 | 35.156 | -.048 |
| e15 | <--> | e42 | 35.039 | -.050 |
| e15 | <--> | e41 | 45.911 | -.056 |
| e15 | <--> | e40 | 50.784 | -.058 |
| e15 | <--> | e39 | 27.726 | -.044 |
| e15 | <--> | e38 | 26.782 | -.043 |
| e15 | <--> | e37 | 43.988 | -.055 |
| e15 | <--> | e36 | 38.170 | -.074 |
| e15 | <--> | e35 | 23.304 | -.054 |
| e15 | <--> | e34 | 39.666 | -.075 |
| e15 | <--> | e33 | 20.382 | -.053 |
| e15 | <--> | e32 | 28.147 | -.062 |
| e15 | <--> | e31 | 21.827 | -.059 |
| e15 | <--> | e30 | 24.759 | -.045 |
| e15 | <--> | e29 | 23.655 | -.044 |
| e15 | <--> | e28 | 17.931 | -.039 |
| e15 | <--> | e27 | 25.293 | -.047 |
| e15 | <--> | e26 | 51.167 | -.067 |
| e15 | <--> | e25 | 39.749 | -.058 |
| e15 | <--> | e24 | 36.790 | -.056 |
| e15 | <--> | e23 | 46.742 | -.063 |
| e15 | <--> | e22 | 38.760 | -.058 |
| e15 | <--> | e21 | 49.867 | -.066 |
| e15 | <--> | e20 | 18.069 | .037 |
| e15 | <--> | e19 | 35.459 | .051 |
| e15 | <--> | e18 | 26.613 | .044 |
| e15 | <--> | e17 | 300.428 | .148 |
| e15 | <--> | e16 | 283.569 | .146 |
| e14 | <--> | e45 | 40.727 | -.054 |
| e14 | <--> | e44 | 49.966 | -.060 |
| e14 | <--> | e43 | 43.194 | -.054 |
| e14 | <--> | e42 | 45.221 | -.057 |
| e14 | <--> | e41 | 33.860 | -.049 |
| e14 | <--> | e40 | 48.817 | -.057 |
| e14 | <--> | e39 | 39.373 | -.053 |
| e14 | <--> | e38 | 50.931 | -.060 |
| e14 | <--> | e37 | 44.298 | -.056 |
| e14 | <--> | e36 | 12.537 | -.042 |
| e14 | <--> | e35 | 11.452 | -.038 |
| e14 | <--> | e34 | 19.108 | -.052 |
| e14 | <--> | e33 | 19.197 | -.052 |
| e14 | <--> | e32 | 14.939 | -.045 |
| e14 | <--> | e31 | 18.910 | -.055 |
| e14 | <--> | e30 | 10.532 | -.029 |
| e14 | <--> | e29 | 23.072 | -.044 |
| e14 | <--> | e28 | 20.421 | -.041 |
| e14 | <--> | e27 | 25.092 | -.047 |
| e14 | <--> | e26 | 19.191 | -.041 |
| e14 | <--> | e25 | 40.068 | -.059 |
| e14 | <--> | e24 | 31.995 | -.053 |
| e14 | <--> | e23 | 17.574 | -.039 |
| e14 | <--> | e22 | 19.689 | -.041 |
| e14 | <--> | e21 | 25.516 | -.048 |
| e14 | <--> | e20 | 11.087 | .029 |
| e14 | <--> | e19 | 4.035 | .017 |
| e14 | <--> | e18 | 6.530 | .022 |
| e14 | <--> | e17 | 267.863 | .141 |
| e14 | <--> | e16 | 252.336 | .139 |
| e14 | <--> | e15 | 261.526 | .139 |
| e13 | <--> | e45 | 38.871 | -.052 |
| e13 | <--> | e44 | 48.847 | -.059 |
| e13 | <--> | e43 | 43.854 | -.054 |
| e13 | <--> | e42 | 34.138 | -.049 |
| e13 | <--> | e41 | 60.520 | -.065 |
| e13 | <--> | e40 | 51.749 | -.058 |
| e13 | <--> | e39 | 45.448 | -.056 |
| e13 | <--> | e38 | 48.858 | -.058 |
| e13 | <--> | e37 | 34.856 | -.049 |
| e13 | <--> | e36 | 34.246 | -.070 |
| e13 | <--> | e35 | 24.166 | -.055 |
| e13 | <--> | e34 | 45.544 | -.080 |
| e13 | <--> | e33 | 31.127 | -.065 |
| e13 | <--> | e32 | 25.802 | -.059 |
| e13 | <--> | e31 | 26.323 | -.065 |
| e13 | <--> | e30 | 20.308 | -.041 |
| e13 | <--> | e29 | 17.284 | -.038 |
| e13 | <--> | e28 | 24.184 | -.045 |
| e13 | <--> | e27 | 19.224 | -.041 |
| e13 | <--> | e26 | 19.675 | -.042 |
| e13 | <--> | e25 | 13.068 | -.033 |
| e13 | <--> | e24 | 13.062 | -.034 |
| e13 | <--> | e23 | 10.980 | -.030 |
| e13 | <--> | e22 | 29.976 | -.051 |
| e13 | <--> | e21 | 27.381 | -.049 |
| e13 | <--> | e20 | 19.764 | .038 |
| e13 | <--> | e19 | 23.472 | .041 |
| e13 | <--> | e18 | 24.740 | .042 |
| e13 | <--> | e17 | 240.139 | .132 |
| e13 | <--> | e16 | 274.698 | .144 |
| e13 | <--> | e15 | 287.693 | .145 |
| e13 | <--> | e14 | 306.597 | .150 |
| e12 | <--> | e45 | 44.908 | -.056 |
| e12 | <--> | e44 | 58.027 | -.064 |
| e12 | <--> | e43 | 30.078 | -.045 |
| e12 | <--> | e42 | 41.418 | -.054 |
| e12 | <--> | e41 | 35.309 | -.050 |
| e12 | <--> | e40 | 51.915 | -.059 |
| e12 | <--> | e39 | 29.576 | -.045 |
| e12 | <--> | e38 | 41.321 | -.054 |
| e12 | <--> | e37 | 28.553 | -.045 |
| e12 | <--> | e36 | 43.304 | -.079 |
| e12 | <--> | e35 | 30.492 | -.062 |
| e12 | <--> | e34 | 20.192 | -.053 |
| e12 | <--> | e33 | 34.484 | -.069 |
| e12 | <--> | e32 | 15.276 | -.045 |
| e12 | <--> | e31 | 26.989 | -.066 |
| e12 | <--> | e30 | 25.406 | -.046 |
| e12 | <--> | e29 | 22.947 | -.044 |
| e12 | <--> | e28 | 6.764 | -.024 |
| e12 | <--> | e27 | 25.554 | -.047 |
| e12 | <--> | e26 | 17.407 | -.039 |
| e12 | <--> | e25 | 29.951 | -.050 |
| e12 | <--> | e24 | 37.476 | -.057 |
| e12 | <--> | e23 | 32.512 | -.052 |
| e12 | <--> | e22 | 24.274 | -.046 |
| e12 | <--> | e21 | 40.659 | -.060 |
| e12 | <--> | e20 | 16.827 | .036 |
| e12 | <--> | e19 | 8.756 | .025 |
| e12 | <--> | e18 | 17.177 | .035 |
| e12 | <--> | e17 | 9.448 | .026 |
| e12 | <--> | e16 | 19.588 | .038 |
| e12 | <--> | e15 | 19.129 | .037 |
| e12 | <--> | e14 | 7.054 | .023 |
| e12 | <--> | e13 | 8.636 | .025 |
| e11 | <--> | e45 | 66.922 | -.071 |
| e11 | <--> | e44 | 65.522 | -.071 |
| e11 | <--> | e43 | 40.534 | -.054 |
| e11 | <--> | e42 | 37.183 | -.053 |
| e11 | <--> | e41 | 75.019 | -.075 |
| e11 | <--> | e40 | 48.273 | -.058 |
| e11 | <--> | e39 | 53.164 | -.063 |
| e11 | <--> | e38 | 51.478 | -.062 |
| e11 | <--> | e37 | 40.999 | -.055 |
| e11 | <--> | e36 | 23.943 | -.060 |
| e11 | <--> | e35 | 35.940 | -.069 |
| e11 | <--> | e34 | 36.163 | -.074 |
| e11 | <--> | e33 | 30.670 | -.067 |
| e11 | <--> | e32 | 28.640 | -.064 |
| e11 | <--> | e31 | 28.279 | -.069 |
| e11 | <--> | e30 | 16.966 | -.038 |
| e11 | <--> | e29 | 17.574 | -.039 |
| e11 | <--> | e28 | 14.525 | -.036 |
| e11 | <--> | e27 | 24.298 | -.047 |
| e11 | <--> | e26 | 45.946 | -.066 |
| e11 | <--> | e25 | 41.322 | -.061 |
| e11 | <--> | e24 | 28.355 | -.051 |
| e11 | <--> | e23 | 41.078 | -.061 |
| e11 | <--> | e22 | 43.773 | -.063 |
| e11 | <--> | e21 | 41.149 | -.062 |
| e11 | <--> | e20 | 14.123 | .034 |
| e11 | <--> | e19 | 5.091 | .020 |
| e11 | <--> | e18 | 29.398 | .048 |
| e11 | <--> | e17 | 28.616 | .047 |
| e11 | <--> | e16 | 18.987 | .039 |
| e11 | <--> | e15 | 14.894 | .034 |
| e11 | <--> | e14 | 16.991 | .037 |
| e11 | <--> | e13 | 23.141 | .042 |
| e11 | <--> | e12 | 238.962 | .136 |
| e10 | <--> | e45 | 48.550 | -.059 |
| e10 | <--> | e44 | 60.141 | -.067 |
| e10 | <--> | e43 | 43.618 | -.055 |
| e10 | <--> | e42 | 40.784 | -.054 |
| e10 | <--> | e41 | 65.947 | -.069 |
| e10 | <--> | e40 | 35.628 | -.049 |
| e10 | <--> | e39 | 41.813 | -.055 |
| e10 | <--> | e38 | 51.745 | -.061 |
| e10 | <--> | e37 | 44.265 | -.057 |
| e10 | <--> | e36 | 21.041 | -.056 |
| e10 | <--> | e35 | 27.174 | -.059 |
| e10 | <--> | e34 | 33.198 | -.070 |
| e10 | <--> | e33 | 29.189 | -.065 |
| e10 | <--> | e32 | 27.687 | -.062 |
| e10 | <--> | e31 | 19.605 | -.057 |
| e10 | <--> | e30 | 32.021 | -.052 |
| e10 | <--> | e29 | 17.050 | -.038 |
| e10 | <--> | e28 | 12.958 | -.033 |
| e10 | <--> | e27 | 22.837 | -.045 |
| e10 | <--> | e26 | 25.549 | -.048 |
| e10 | <--> | e25 | 25.818 | -.048 |
| e10 | <--> | e24 | 21.776 | -.044 |
| e10 | <--> | e23 | 23.673 | -.045 |
| e10 | <--> | e22 | 22.933 | -.045 |
| e10 | <--> | e21 | 21.932 | -.045 |
| e10 | <--> | e20 | 11.902 | .030 |
| e10 | <--> | e18 | 19.704 | .038 |
| e10 | <--> | e17 | 17.215 | .036 |
| e10 | <--> | e16 | 11.661 | .030 |
| e10 | <--> | e15 | 20.489 | .039 |
| e10 | <--> | e14 | 5.075 | .020 |
| e10 | <--> | e13 | 15.760 | .034 |
| e10 | <--> | e12 | 231.599 | .132 |
| e10 | <--> | e11 | 279.268 | .150 |
| e9 | <--> | e45 | 42.113 | -.055 |
| e9 | <--> | e44 | 37.923 | -.053 |
| e9 | <--> | e43 | 50.564 | -.059 |
| e9 | <--> | e42 | 34.432 | -.050 |
| e9 | <--> | e41 | 66.757 | -.070 |
| e9 | <--> | e40 | 32.413 | -.047 |
| e9 | <--> | e39 | 61.598 | -.067 |
| e9 | <--> | e38 | 47.180 | -.059 |
| e9 | <--> | e37 | 35.690 | -.051 |
| e9 | <--> | e36 | 46.583 | -.083 |
| e9 | <--> | e35 | 44.074 | -.076 |
| e9 | <--> | e34 | 46.193 | -.082 |
| e9 | <--> | e33 | 47.858 | -.083 |
| e9 | <--> | e32 | 43.485 | -.078 |
| e9 | <--> | e31 | 27.727 | -.068 |
| e9 | <--> | e30 | 23.917 | -.045 |
| e9 | <--> | e29 | 26.184 | -.047 |
| e9 | <--> | e28 | 24.090 | -.046 |
| e9 | <--> | e27 | 21.570 | -.044 |
| e9 | <--> | e26 | 43.802 | -.064 |
| e9 | <--> | e25 | 33.254 | -.054 |
| e9 | <--> | e24 | 22.196 | -.045 |
| e9 | <--> | e23 | 31.604 | -.053 |
| e9 | <--> | e22 | 26.878 | -.049 |
| e9 | <--> | e21 | 26.788 | -.049 |
| e9 | <--> | e20 | 16.430 | .036 |
| e9 | <--> | e19 | 13.187 | .032 |
| e9 | <--> | e18 | 27.152 | .045 |
| e9 | <--> | e17 | 14.977 | .034 |
| e9 | <--> | e16 | 18.446 | .038 |
| e9 | <--> | e15 | 23.387 | .042 |
| e9 | <--> | e14 | 12.749 | .031 |
| e9 | <--> | e13 | 25.401 | .044 |
| e9 | <--> | e12 | 249.207 | .137 |
| e9 | <--> | e11 | 257.707 | .144 |
| e9 | <--> | e10 | 275.114 | .147 |
| e8 | <--> | e45 | 29.108 | -.047 |
| e8 | <--> | e44 | 55.363 | -.065 |
| e8 | <--> | e43 | 33.012 | -.048 |
| e8 | <--> | e42 | 24.659 | -.043 |
| e8 | <--> | e41 | 46.559 | -.059 |
| e8 | <--> | e40 | 59.100 | -.064 |
| e8 | <--> | e39 | 52.288 | -.062 |
| e8 | <--> | e38 | 42.365 | -.056 |
| e8 | <--> | e37 | 33.682 | -.050 |
| e8 | <--> | e36 | 31.916 | -.070 |
| e8 | <--> | e35 | 18.385 | -.049 |
| e8 | <--> | e34 | 38.177 | -.076 |
| e8 | <--> | e33 | 35.197 | -.072 |
| e8 | <--> | e32 | 24.829 | -.060 |
| e8 | <--> | e31 | 31.563 | -.073 |
| e8 | <--> | e30 | 9.553 | -.029 |
| e8 | <--> | e29 | 8.638 | -.028 |
| e8 | <--> | e28 | 10.752 | -.031 |
| e8 | <--> | e27 | 17.090 | -.040 |
| e8 | <--> | e26 | 30.338 | -.054 |
| e8 | <--> | e25 | 22.555 | -.045 |
| e8 | <--> | e24 | 12.059 | -.033 |
| e8 | <--> | e23 | 31.882 | -.053 |
| e8 | <--> | e22 | 20.421 | -.043 |
| e8 | <--> | e21 | 37.046 | -.059 |
| e8 | <--> | e20 | 7.402 | .024 |
| e8 | <--> | e18 | 8.143 | .025 |
| e8 | <--> | e17 | 18.754 | .038 |
| e8 | <--> | e16 | 32.738 | .051 |
| e8 | <--> | e15 | 16.772 | .036 |
| e8 | <--> | e14 | 11.717 | .030 |
| e8 | <--> | e13 | 16.891 | .036 |
| e8 | <--> | e12 | 234.087 | .135 |
| e8 | <--> | e11 | 287.325 | .154 |
| e8 | <--> | e10 | 216.315 | .132 |
| e8 | <--> | e9 | 257.547 | .144 |
| e7 | <--> | e45 | 36.910 | -.052 |
| e7 | <--> | e44 | 53.024 | -.063 |
| e7 | <--> | e43 | 53.775 | -.062 |
| e7 | <--> | e42 | 35.911 | -.052 |
| e7 | <--> | e41 | 49.623 | -.061 |
| e7 | <--> | e40 | 30.219 | -.046 |
| e7 | <--> | e39 | 39.478 | -.054 |
| e7 | <--> | e38 | 22.559 | -.041 |
| e7 | <--> | e37 | 30.873 | -.048 |
| e7 | <--> | e36 | 25.751 | -.062 |
| e7 | <--> | e35 | 32.012 | -.065 |
| e7 | <--> | e34 | 23.838 | -.060 |
| e7 | <--> | e33 | 39.899 | -.076 |
| e7 | <--> | e32 | 28.901 | -.064 |
| e7 | <--> | e31 | 24.243 | -.064 |
| e7 | <--> | e30 | 12.290 | -.033 |
| e7 | <--> | e29 | 20.421 | -.042 |
| e7 | <--> | e28 | 16.988 | -.039 |
| e7 | <--> | e27 | 21.870 | -.045 |
| e7 | <--> | e26 | 30.071 | -.053 |
| e7 | <--> | e25 | 30.537 | -.052 |
| e7 | <--> | e24 | 20.559 | -.044 |
| e7 | <--> | e23 | 28.412 | -.050 |
| e7 | <--> | e22 | 15.178 | -.037 |
| e7 | <--> | e21 | 17.929 | -.041 |
| e7 | <--> | e20 | 5.812 | .021 |
| e7 | <--> | e19 | 14.807 | .034 |
| e7 | <--> | e18 | 17.288 | .036 |
| e7 | <--> | e17 | 14.303 | .033 |
| e7 | <--> | e16 | 22.723 | .043 |
| e7 | <--> | e15 | 9.666 | .027 |
| e7 | <--> | e14 | 7.579 | .024 |
| e7 | <--> | e13 | 14.289 | .033 |
| e7 | <--> | e12 | 240.213 | .136 |
| e7 | <--> | e11 | 288.647 | .154 |
| e7 | <--> | e10 | 245.601 | .140 |
| e7 | <--> | e9 | 251.343 | .142 |
| e7 | <--> | e8 | 226.758 | .136 |
| e6 | <--> | e45 | 56.366 | -.064 |
| e6 | <--> | e44 | 57.700 | -.065 |
| e6 | <--> | e43 | 66.652 | -.068 |
| e6 | <--> | e42 | 46.762 | -.058 |
| e6 | <--> | e41 | 54.381 | -.062 |
| e6 | <--> | e40 | 47.609 | -.057 |
| e6 | <--> | e39 | 46.305 | -.058 |
| e6 | <--> | e38 | 64.546 | -.068 |
| e6 | <--> | e37 | 49.453 | -.060 |
| e6 | <--> | e36 | 18.336 | -.052 |
| e6 | <--> | e35 | 29.857 | -.062 |
| e6 | <--> | e34 | 19.175 | -.053 |
| e6 | <--> | e33 | 37.936 | -.073 |
| e6 | <--> | e32 | 19.582 | -.052 |
| e6 | <--> | e31 | 20.332 | -.058 |
| e6 | <--> | e30 | 16.162 | -.037 |
| e6 | <--> | e29 | 22.425 | -.044 |
| e6 | <--> | e28 | 21.156 | -.043 |
| e6 | <--> | e27 | 25.721 | -.048 |
| e6 | <--> | e26 | 27.440 | -.050 |
| e6 | <--> | e25 | 32.396 | -.053 |
| e6 | <--> | e24 | 31.906 | -.053 |
| e6 | <--> | e23 | 29.219 | -.050 |
| e6 | <--> | e22 | 23.399 | -.045 |
| e6 | <--> | e21 | 25.996 | -.048 |
| e6 | <--> | e20 | 12.054 | .031 |
| e6 | <--> | e19 | 11.749 | .030 |
| e6 | <--> | e18 | 15.122 | .034 |
| e6 | <--> | e17 | 18.019 | .037 |
| e6 | <--> | e16 | 13.879 | .033 |
| e6 | <--> | e15 | 12.525 | .031 |
| e6 | <--> | e14 | 18.429 | .037 |
| e6 | <--> | e13 | 10.305 | .028 |
| e6 | <--> | e12 | 6.338 | .022 |
| e6 | <--> | e11 | 27.771 | .047 |
| e6 | <--> | e10 | 15.150 | .034 |
| e6 | <--> | e9 | 10.611 | .029 |
| e6 | <--> | e8 | 13.649 | .033 |
| e6 | <--> | e7 | 4.722 | .019 |
| e5 | <--> | e45 | 41.204 | -.056 |
| e5 | <--> | e44 | 45.111 | -.059 |
| e5 | <--> | e43 | 47.674 | -.059 |
| e5 | <--> | e42 | 48.253 | -.061 |
| e5 | <--> | e41 | 45.507 | -.059 |
| e5 | <--> | e40 | 26.415 | -.044 |
| e5 | <--> | e39 | 31.959 | -.049 |
| e5 | <--> | e38 | 43.001 | -.057 |
| e5 | <--> | e37 | 37.583 | -.054 |
| e5 | <--> | e36 | 33.221 | -.072 |
| e5 | <--> | e35 | 32.052 | -.066 |
| e5 | <--> | e34 | 19.911 | -.055 |
| e5 | <--> | e33 | 19.816 | -.055 |
| e5 | <--> | e32 | 38.241 | -.075 |
| e5 | <--> | e31 | 32.342 | -.075 |
| e5 | <--> | e30 | 22.267 | -.045 |
| e5 | <--> | e29 | 10.912 | -.031 |
| e5 | <--> | e28 | 18.679 | -.041 |
| e5 | <--> | e27 | 22.426 | -.046 |
| e5 | <--> | e26 | 36.016 | -.059 |
| e5 | <--> | e25 | 45.638 | -.065 |
| e5 | <--> | e24 | 39.259 | -.061 |
| e5 | <--> | e23 | 42.503 | -.063 |
| e5 | <--> | e22 | 42.144 | -.063 |
| e5 | <--> | e21 | 52.567 | -.071 |
| e5 | <--> | e20 | 25.635 | .046 |
| e5 | <--> | e19 | 29.438 | .048 |
| e5 | <--> | e18 | 33.776 | .052 |
| e5 | <--> | e17 | 5.175 | .020 |
| e5 | <--> | e16 | 9.449 | .028 |
| e5 | <--> | e15 | 10.265 | .029 |
| e5 | <--> | e14 | 7.875 | .025 |
| e5 | <--> | e13 | 5.254 | .020 |
| e5 | <--> | e12 | 16.845 | .037 |
| e5 | <--> | e11 | 6.923 | .024 |
| e5 | <--> | e10 | 6.078 | .022 |
| e5 | <--> | e9 | 16.474 | .037 |
| e5 | <--> | e7 | 8.447 | .027 |
| e5 | <--> | e6 | 226.037 | .136 |
| e4 | <--> | e45 | 36.799 | -.053 |
| e4 | <--> | e44 | 56.566 | -.066 |
| e4 | <--> | e43 | 23.081 | -.041 |
| e4 | <--> | e42 | 53.988 | -.064 |
| e4 | <--> | e41 | 53.144 | -.063 |
| e4 | <--> | e40 | 25.255 | -.042 |
| e4 | <--> | e39 | 45.541 | -.058 |
| e4 | <--> | e38 | 64.782 | -.070 |
| e4 | <--> | e37 | 56.818 | -.065 |
| e4 | <--> | e36 | 9.050 | -.037 |
| e4 | <--> | e35 | 23.187 | -.056 |
| e4 | <--> | e34 | 9.174 | -.037 |
| e4 | <--> | e33 | 17.452 | -.051 |
| e4 | <--> | e32 | 19.643 | -.053 |
| e4 | <--> | e31 | 8.144 | -.037 |
| e4 | <--> | e30 | 26.120 | -.048 |
| e4 | <--> | e29 | 31.718 | -.053 |
| e4 | <--> | e28 | 24.018 | -.046 |
| e4 | <--> | e27 | 29.519 | -.052 |
| e4 | <--> | e26 | 22.107 | -.046 |
| e4 | <--> | e25 | 34.453 | -.056 |
| e4 | <--> | e24 | 39.353 | -.061 |
| e4 | <--> | e23 | 31.355 | -.053 |
| e4 | <--> | e22 | 26.880 | -.050 |
| e4 | <--> | e21 | 43.325 | -.064 |
| e4 | <--> | e20 | 16.235 | .036 |
| e4 | <--> | e19 | 20.166 | .040 |
| e4 | <--> | e18 | 21.750 | .041 |
| e4 | <--> | e16 | 4.952 | .020 |
| e4 | <--> | e15 | 11.786 | .030 |
| e4 | <--> | e14 | 6.323 | .022 |
| e4 | <--> | e11 | 7.786 | .025 |
| e4 | <--> | e10 | 7.023 | .024 |
| e4 | <--> | e9 | 7.650 | .025 |
| e4 | <--> | e6 | 283.461 | .151 |
| e4 | <--> | e5 | 287.141 | .157 |
| e3 | <--> | e45 | 44.725 | -.057 |
| e3 | <--> | e44 | 53.563 | -.063 |
| e3 | <--> | e43 | 50.521 | -.059 |
| e3 | <--> | e42 | 35.557 | -.051 |
| e3 | <--> | e41 | 55.078 | -.063 |
| e3 | <--> | e40 | 38.111 | -.051 |
| e3 | <--> | e39 | 35.258 | -.051 |
| e3 | <--> | e38 | 78.829 | -.076 |
| e3 | <--> | e37 | 40.665 | -.054 |
| e3 | <--> | e36 | 26.991 | -.063 |
| e3 | <--> | e35 | 36.270 | -.069 |
| e3 | <--> | e34 | 28.091 | -.064 |
| e3 | <--> | e33 | 23.087 | -.058 |
| e3 | <--> | e32 | 27.494 | -.062 |
| e3 | <--> | e31 | 21.394 | -.060 |
| e3 | <--> | e30 | 11.435 | -.031 |
| e3 | <--> | e29 | 15.493 | -.037 |
| e3 | <--> | e27 | 7.804 | -.026 |
| e3 | <--> | e26 | 36.353 | -.058 |
| e3 | <--> | e25 | 28.433 | -.050 |
| e3 | <--> | e24 | 47.989 | -.066 |
| e3 | <--> | e23 | 23.919 | -.046 |
| e3 | <--> | e22 | 29.181 | -.051 |
| e3 | <--> | e21 | 48.151 | -.066 |
| e3 | <--> | e20 | 23.936 | .043 |
| e3 | <--> | e19 | 8.794 | .026 |
| e3 | <--> | e18 | 22.543 | .041 |
| e3 | <--> | e17 | 17.503 | .036 |
| e3 | <--> | e16 | 20.745 | .040 |
| e3 | <--> | e15 | 9.307 | .027 |
| e3 | <--> | e14 | 20.174 | .039 |
| e3 | <--> | e13 | 7.095 | .023 |
| e3 | <--> | e12 | 5.003 | .019 |
| e3 | <--> | e11 | 6.687 | .023 |
| e3 | <--> | e10 | 14.193 | .033 |
| e3 | <--> | e9 | 4.192 | .018 |
| e3 | <--> | e7 | 5.141 | .020 |
| e3 | <--> | e6 | 244.838 | .138 |
| e3 | <--> | e5 | 290.209 | .155 |
| e3 | <--> | e4 | 275.746 | .150 |
| e2 | <--> | e45 | 20.742 | -.039 |
| e2 | <--> | e44 | 34.527 | -.051 |
| e2 | <--> | e43 | 20.937 | -.039 |
| e2 | <--> | e42 | 47.983 | -.060 |
| e2 | <--> | e41 | 21.855 | -.040 |
| e2 | <--> | e40 | 28.521 | -.045 |
| e2 | <--> | e39 | 35.507 | -.051 |
| e2 | <--> | e38 | 24.564 | -.043 |
| e2 | <--> | e37 | 29.458 | -.047 |
| e2 | <--> | e36 | 29.056 | -.067 |
| e2 | <--> | e35 | 27.417 | -.061 |
| e2 | <--> | e34 | 27.811 | -.065 |
| e2 | <--> | e33 | 61.218 | -.095 |
| e2 | <--> | e32 | 35.408 | -.072 |
| e2 | <--> | e31 | 32.482 | -.074 |
| e2 | <--> | e30 | 19.873 | -.042 |
| e2 | <--> | e29 | 12.779 | -.034 |
| e2 | <--> | e28 | 13.116 | -.034 |
| e2 | <--> | e27 | 23.374 | -.046 |
| e2 | <--> | e26 | 17.936 | -.041 |
| e2 | <--> | e25 | 22.223 | -.045 |
| e2 | <--> | e24 | 31.571 | -.054 |
| e2 | <--> | e23 | 29.753 | -.052 |
| e2 | <--> | e22 | 24.797 | -.048 |
| e2 | <--> | e21 | 49.982 | -.068 |
| e2 | <--> | e20 | 9.294 | .027 |
| e2 | <--> | e19 | 10.006 | .028 |
| e2 | <--> | e18 | 20.180 | .040 |
| e2 | <--> | e17 | 6.101 | .022 |
| e2 | <--> | e16 | 9.760 | .028 |
| e2 | <--> | e15 | 10.173 | .028 |
| e2 | <--> | e14 | 15.461 | .035 |
| e2 | <--> | e13 | 8.903 | .026 |
| e2 | <--> | e12 | 6.787 | .023 |
| e2 | <--> | e11 | 5.300 | .021 |
| e2 | <--> | e10 | 4.010 | .018 |
| e2 | <--> | e9 | 11.014 | .030 |
| e2 | <--> | e6 | 262.483 | .145 |
| e2 | <--> | e5 | 250.426 | .146 |
| e2 | <--> | e4 | 283.387 | .154 |
| e2 | <--> | e3 | 212.671 | .132 |
| e1 | <--> | e45 | 59.042 | -.066 |
| e1 | <--> | e44 | 36.077 | -.052 |
| e1 | <--> | e43 | 42.043 | -.054 |
| e1 | <--> | e42 | 42.353 | -.056 |
| e1 | <--> | e41 | 66.132 | -.069 |
| e1 | <--> | e40 | 45.411 | -.056 |
| e1 | <--> | e39 | 48.721 | -.059 |
| e1 | <--> | e38 | 47.160 | -.059 |
| e1 | <--> | e37 | 46.725 | -.058 |
| e1 | <--> | e36 | 23.650 | -.059 |
| e1 | <--> | e35 | 25.176 | -.057 |
| e1 | <--> | e34 | 19.558 | -.054 |
| e1 | <--> | e33 | 26.077 | -.061 |
| e1 | <--> | e32 | 32.384 | -.067 |
| e1 | <--> | e31 | 21.273 | -.059 |
| e1 | <--> | e30 | 23.358 | -.044 |
| e1 | <--> | e29 | 19.015 | -.040 |
| e1 | <--> | e28 | 11.215 | -.031 |
| e1 | <--> | e27 | 12.872 | -.034 |
| e1 | <--> | e26 | 24.793 | -.048 |
| e1 | <--> | e25 | 22.082 | -.044 |
| e1 | <--> | e24 | 22.184 | -.045 |
| e1 | <--> | e23 | 18.074 | -.040 |
| e1 | <--> | e22 | 22.367 | -.045 |
| e1 | <--> | e21 | 13.848 | -.036 |
| e1 | <--> | e20 | 19.000 | .038 |
| e1 | <--> | e19 | 13.395 | .032 |
| e1 | <--> | e18 | 6.378 | .022 |
| e1 | <--> | e17 | 9.147 | .026 |
| e1 | <--> | e16 | 11.278 | .030 |
| e1 | <--> | e15 | 18.048 | .037 |
| e1 | <--> | e14 | 14.255 | .033 |
| e1 | <--> | e13 | 16.848 | .036 |
| e1 | <--> | e12 | 9.794 | .027 |
| e1 | <--> | e11 | 6.918 | .024 |
| e1 | <--> | e10 | 5.502 | .021 |
| e1 | <--> | e9 | 16.340 | .036 |
| e1 | <--> | e6 | 241.556 | .137 |
| e1 | <--> | e5 | 228.825 | .138 |
| e1 | <--> | e4 | 252.363 | .143 |
| e1 | <--> | e3 | 264.002 | .144 |
| e1 | <--> | e2 | 197.680 | .127 |

## Variances: (Group number 1 - Default model)

|  |  |  | M.I. | Par Change |
| --- | --- | --- | --- | --- |

## Regression Weights: (Group number 1 - Default model)

|  |  |  | M.I. | Par Change |
| --- | --- | --- | --- | --- |
| EOE\_9 | <--- | EOE\_8 | 187.693 | .244 |
| EOE\_9 | <--- | EOE\_7 | 213.767 | .265 |
| EOE\_9 | <--- | EOE\_6 | 216.378 | .266 |
| EOE\_9 | <--- | EOE\_5 | 224.760 | .268 |
| EOE\_9 | <--- | EOE\_4 | 127.119 | .206 |
| EOE\_9 | <--- | EOE\_3 | 179.909 | .241 |
| EOE\_9 | <--- | EOE\_2 | 163.659 | .230 |
| EOE\_9 | <--- | EOE\_1 | 166.168 | .232 |
| EOE\_9 | <--- | ESE\_3 | 4.656 | -.037 |
| EOE\_9 | <--- | EE\_20 | 9.901 | -.055 |
| EOE\_9 | <--- | EE\_19 | 6.419 | -.045 |
| EOE\_9 | <--- | EE\_18 | 15.377 | -.069 |
| EOE\_9 | <--- | EE\_17 | 18.422 | -.074 |
| EOE\_9 | <--- | EE\_16 | 28.425 | -.091 |
| EOE\_9 | <--- | EE\_15 | 33.050 | -.099 |
| EOE\_9 | <--- | EE\_14 | 27.204 | -.089 |
| EOE\_9 | <--- | EE\_13 | 25.238 | -.085 |
| EOE\_9 | <--- | EE\_12 | 29.172 | -.092 |
| EOE\_9 | <--- | EE\_11 | 45.948 | -.114 |
| EOE\_9 | <--- | EE\_10 | 31.963 | -.095 |
| EOE\_9 | <--- | EE\_9 | 28.149 | -.090 |
| EOE\_9 | <--- | EE\_8 | 20.082 | -.076 |
| EOE\_9 | <--- | EE\_7 | 24.776 | -.083 |
| EOE\_9 | <--- | EE\_6 | 37.074 | -.103 |
| EOE\_9 | <--- | EE\_5 | 28.422 | -.089 |
| EOE\_9 | <--- | EE\_4 | 25.547 | -.085 |
| EOE\_9 | <--- | EE\_3 | 29.321 | -.091 |
| EOE\_9 | <--- | EE\_2 | 13.983 | -.062 |
| EOE\_9 | <--- | EE\_1 | 39.953 | -.107 |
| EOE\_8 | <--- | EOE\_9 | 187.247 | .248 |
| EOE\_8 | <--- | EOE\_7 | 213.473 | .267 |
| EOE\_8 | <--- | EOE\_6 | 206.867 | .263 |
| EOE\_8 | <--- | EOE\_5 | 220.299 | .268 |
| EOE\_8 | <--- | EOE\_4 | 166.553 | .238 |
| EOE\_8 | <--- | EOE\_3 | 180.001 | .244 |
| EOE\_8 | <--- | EOE\_2 | 182.383 | .245 |
| EOE\_8 | <--- | EOE\_1 | 199.132 | .257 |
| EOE\_8 | <--- | EE\_20 | 22.356 | -.084 |
| EOE\_8 | <--- | EE\_19 | 14.199 | -.067 |
| EOE\_8 | <--- | EE\_18 | 22.856 | -.086 |
| EOE\_8 | <--- | EE\_17 | 22.827 | -.083 |
| EOE\_8 | <--- | EE\_16 | 34.139 | -.101 |
| EOE\_8 | <--- | EE\_15 | 27.042 | -.090 |
| EOE\_8 | <--- | EE\_14 | 33.375 | -.100 |
| EOE\_8 | <--- | EE\_13 | 31.716 | -.097 |
| EOE\_8 | <--- | EE\_12 | 37.693 | -.105 |
| EOE\_8 | <--- | EE\_11 | 44.987 | -.114 |
| EOE\_8 | <--- | EE\_10 | 39.594 | -.107 |
| EOE\_8 | <--- | EE\_9 | 25.348 | -.086 |
| EOE\_8 | <--- | EE\_8 | 38.195 | -.106 |
| EOE\_8 | <--- | EE\_7 | 35.592 | -.101 |
| EOE\_8 | <--- | EE\_6 | 37.951 | -.105 |
| EOE\_8 | <--- | EE\_5 | 31.117 | -.094 |
| EOE\_8 | <--- | EE\_4 | 39.270 | -.107 |
| EOE\_8 | <--- | EE\_3 | 35.114 | -.100 |
| EOE\_8 | <--- | EE\_2 | 23.277 | -.081 |
| EOE\_8 | <--- | EE\_1 | 24.412 | -.085 |
| EOE\_7 | <--- | EOE\_9 | 222.056 | .261 |
| EOE\_7 | <--- | EOE\_8 | 222.280 | .258 |
| EOE\_7 | <--- | EOE\_6 | 234.578 | .270 |
| EOE\_7 | <--- | EOE\_5 | 217.582 | .257 |
| EOE\_7 | <--- | EOE\_4 | 169.493 | .231 |
| EOE\_7 | <--- | EOE\_3 | 174.921 | .232 |
| EOE\_7 | <--- | EOE\_2 | 195.014 | .244 |
| EOE\_7 | <--- | EOE\_1 | 183.277 | .238 |
| EOE\_7 | <--- | ESE\_10 | 4.573 | -.036 |
| EOE\_7 | <--- | ESE\_2 | 4.629 | -.036 |
| EOE\_7 | <--- | EE\_20 | 15.483 | -.067 |
| EOE\_7 | <--- | EE\_19 | 11.329 | -.058 |
| EOE\_7 | <--- | EE\_18 | 18.682 | -.075 |
| EOE\_7 | <--- | EE\_17 | 17.492 | -.070 |
| EOE\_7 | <--- | EE\_16 | 32.453 | -.095 |
| EOE\_7 | <--- | EE\_15 | 23.296 | -.081 |
| EOE\_7 | <--- | EE\_14 | 28.854 | -.090 |
| EOE\_7 | <--- | EE\_13 | 28.476 | -.089 |
| EOE\_7 | <--- | EE\_12 | 19.540 | -.073 |
| EOE\_7 | <--- | EE\_11 | 27.833 | -.087 |
| EOE\_7 | <--- | EE\_10 | 28.718 | -.088 |
| EOE\_7 | <--- | EE\_9 | 33.800 | -.096 |
| EOE\_7 | <--- | EE\_8 | 22.776 | -.079 |
| EOE\_7 | <--- | EE\_7 | 36.099 | -.098 |
| EOE\_7 | <--- | EE\_6 | 43.843 | -.109 |
| EOE\_7 | <--- | EE\_5 | 32.887 | -.093 |
| EOE\_7 | <--- | EE\_4 | 16.025 | -.066 |
| EOE\_7 | <--- | EE\_3 | 33.123 | -.094 |
| EOE\_7 | <--- | EE\_2 | 14.116 | -.061 |
| EOE\_7 | <--- | EE\_1 | 28.451 | -.088 |
| EOE\_6 | <--- | EOE\_9 | 211.742 | .262 |
| EOE\_6 | <--- | EOE\_8 | 202.918 | .254 |
| EOE\_6 | <--- | EOE\_7 | 220.983 | .270 |
| EOE\_6 | <--- | EOE\_5 | 234.360 | .274 |
| EOE\_6 | <--- | EOE\_4 | 141.541 | .217 |
| EOE\_6 | <--- | EOE\_3 | 177.375 | .240 |
| EOE\_6 | <--- | EOE\_2 | 163.233 | .230 |
| EOE\_6 | <--- | EOE\_1 | 177.007 | .240 |
| EOE\_6 | <--- | ESE\_8 | 5.804 | -.042 |
| EOE\_6 | <--- | EE\_20 | 22.216 | -.083 |
| EOE\_6 | <--- | EE\_19 | 6.695 | -.046 |
| EOE\_6 | <--- | EE\_18 | 22.632 | -.084 |
| EOE\_6 | <--- | EE\_17 | 27.201 | -.090 |
| EOE\_6 | <--- | EE\_16 | 25.912 | -.087 |
| EOE\_6 | <--- | EE\_15 | 23.216 | -.083 |
| EOE\_6 | <--- | EE\_14 | 30.205 | -.094 |
| EOE\_6 | <--- | EE\_13 | 22.165 | -.080 |
| EOE\_6 | <--- | EE\_12 | 26.903 | -.088 |
| EOE\_6 | <--- | EE\_11 | 25.529 | -.085 |
| EOE\_6 | <--- | EE\_10 | 26.849 | -.087 |
| EOE\_6 | <--- | EE\_9 | 23.014 | -.081 |
| EOE\_6 | <--- | EE\_8 | 17.011 | -.070 |
| EOE\_6 | <--- | EE\_7 | 24.104 | -.082 |
| EOE\_6 | <--- | EE\_6 | 30.756 | -.094 |
| EOE\_6 | <--- | EE\_5 | 33.283 | -.096 |
| EOE\_6 | <--- | EE\_4 | 37.479 | -.104 |
| EOE\_6 | <--- | EE\_3 | 23.309 | -.081 |
| EOE\_6 | <--- | EE\_2 | 32.346 | -.095 |
| EOE\_6 | <--- | EE\_1 | 28.658 | -.091 |
| EOE\_5 | <--- | EOE\_9 | 227.696 | .270 |
| EOE\_5 | <--- | EOE\_8 | 223.709 | .265 |
| EOE\_5 | <--- | EOE\_7 | 212.196 | .263 |
| EOE\_5 | <--- | EOE\_6 | 242.620 | .281 |
| EOE\_5 | <--- | EOE\_4 | 186.263 | .248 |
| EOE\_5 | <--- | EOE\_3 | 157.896 | .226 |
| EOE\_5 | <--- | EOE\_2 | 195.099 | .250 |
| EOE\_5 | <--- | EOE\_1 | 166.184 | .232 |
| EOE\_5 | <--- | ESE\_10 | 4.256 | -.036 |
| EOE\_5 | <--- | ESE\_8 | 8.670 | -.051 |
| EOE\_5 | <--- | EE\_20 | 21.547 | -.081 |
| EOE\_5 | <--- | EE\_19 | 7.556 | -.048 |
| EOE\_5 | <--- | EE\_18 | 18.701 | -.076 |
| EOE\_5 | <--- | EE\_17 | 27.725 | -.091 |
| EOE\_5 | <--- | EE\_16 | 22.834 | -.081 |
| EOE\_5 | <--- | EE\_15 | 30.422 | -.094 |
| EOE\_5 | <--- | EE\_14 | 22.618 | -.081 |
| EOE\_5 | <--- | EE\_13 | 39.296 | -.106 |
| EOE\_5 | <--- | EE\_12 | 22.937 | -.081 |
| EOE\_5 | <--- | EE\_11 | 51.509 | -.121 |
| EOE\_5 | <--- | EE\_10 | 43.417 | -.110 |
| EOE\_5 | <--- | EE\_9 | 44.622 | -.112 |
| EOE\_5 | <--- | EE\_8 | 32.122 | -.096 |
| EOE\_5 | <--- | EE\_7 | 33.310 | -.096 |
| EOE\_5 | <--- | EE\_6 | 35.769 | -.100 |
| EOE\_5 | <--- | EE\_5 | 31.391 | -.093 |
| EOE\_5 | <--- | EE\_4 | 36.895 | -.102 |
| EOE\_5 | <--- | EE\_3 | 36.108 | -.100 |
| EOE\_5 | <--- | EE\_2 | 14.734 | -.064 |
| EOE\_5 | <--- | EE\_1 | 44.751 | -.113 |
| EOE\_4 | <--- | EOE\_9 | 131.173 | .200 |
| EOE\_4 | <--- | EOE\_8 | 172.274 | .227 |
| EOE\_4 | <--- | EOE\_7 | 168.369 | .228 |
| EOE\_4 | <--- | EOE\_6 | 149.252 | .214 |
| EOE\_4 | <--- | EOE\_5 | 189.724 | .239 |
| EOE\_4 | <--- | EOE\_3 | 213.585 | .256 |
| EOE\_4 | <--- | EOE\_2 | 227.456 | .263 |
| EOE\_4 | <--- | EOE\_1 | 188.701 | .240 |
| EOE\_4 | <--- | EE\_20 | 16.240 | -.069 |
| EOE\_4 | <--- | EE\_19 | 10.360 | -.055 |
| EOE\_4 | <--- | EE\_18 | 22.515 | -.082 |
| EOE\_4 | <--- | EE\_17 | 43.734 | -.111 |
| EOE\_4 | <--- | EE\_16 | 33.384 | -.096 |
| EOE\_4 | <--- | EE\_15 | 33.652 | -.097 |
| EOE\_4 | <--- | EE\_14 | 32.610 | -.095 |
| EOE\_4 | <--- | EE\_13 | 33.602 | -.096 |
| EOE\_4 | <--- | EE\_12 | 33.726 | -.096 |
| EOE\_4 | <--- | EE\_11 | 33.146 | -.094 |
| EOE\_4 | <--- | EE\_10 | 23.457 | -.079 |
| EOE\_4 | <--- | EE\_9 | 21.666 | -.076 |
| EOE\_4 | <--- | EE\_8 | 40.775 | -.105 |
| EOE\_4 | <--- | EE\_7 | 20.286 | -.073 |
| EOE\_4 | <--- | EE\_6 | 31.316 | -.092 |
| EOE\_4 | <--- | EE\_5 | 18.222 | -.069 |
| EOE\_4 | <--- | EE\_4 | 17.534 | -.069 |
| EOE\_4 | <--- | EE\_3 | 24.986 | -.081 |
| EOE\_4 | <--- | EE\_2 | 19.228 | -.071 |
| EOE\_4 | <--- | EE\_1 | 30.730 | -.091 |
| EOE\_3 | <--- | EOE\_9 | 179.638 | .240 |
| EOE\_3 | <--- | EOE\_8 | 180.158 | .238 |
| EOE\_3 | <--- | EOE\_7 | 168.137 | .234 |
| EOE\_3 | <--- | EOE\_6 | 180.985 | .243 |
| EOE\_3 | <--- | EOE\_5 | 155.625 | .223 |
| EOE\_3 | <--- | EOE\_4 | 206.673 | .261 |
| EOE\_3 | <--- | EOE\_2 | 227.538 | .270 |
| EOE\_3 | <--- | EOE\_1 | 212.807 | .262 |
| EOE\_3 | <--- | ESE\_10 | 4.218 | -.036 |
| EOE\_3 | <--- | ESE\_8 | 5.512 | -.040 |
| EOE\_3 | <--- | EE\_20 | 16.908 | -.072 |
| EOE\_3 | <--- | EE\_19 | 18.733 | -.076 |
| EOE\_3 | <--- | EE\_18 | 24.532 | -.088 |
| EOE\_3 | <--- | EE\_17 | 22.981 | -.083 |
| EOE\_3 | <--- | EE\_16 | 21.979 | -.080 |
| EOE\_3 | <--- | EE\_15 | 18.371 | -.073 |
| EOE\_3 | <--- | EE\_14 | 26.300 | -.088 |
| EOE\_3 | <--- | EE\_13 | 29.509 | -.092 |
| EOE\_3 | <--- | EE\_12 | 19.212 | -.074 |
| EOE\_3 | <--- | EE\_11 | 36.503 | -.102 |
| EOE\_3 | <--- | EE\_10 | 27.527 | -.088 |
| EOE\_3 | <--- | EE\_9 | 41.172 | -.108 |
| EOE\_3 | <--- | EE\_8 | 36.073 | -.101 |
| EOE\_3 | <--- | EE\_7 | 26.499 | -.086 |
| EOE\_3 | <--- | EE\_6 | 30.456 | -.093 |
| EOE\_3 | <--- | EE\_5 | 22.045 | -.078 |
| EOE\_3 | <--- | EE\_4 | 31.616 | -.095 |
| EOE\_3 | <--- | EE\_3 | 23.114 | -.080 |
| EOE\_3 | <--- | EE\_2 | 23.937 | -.081 |
| EOE\_3 | <--- | EE\_1 | 32.968 | -.097 |
| EOE\_2 | <--- | EOE\_9 | 163.376 | .229 |
| EOE\_2 | <--- | EOE\_8 | 182.502 | .240 |
| EOE\_2 | <--- | EOE\_7 | 187.409 | .248 |
| EOE\_2 | <--- | EOE\_6 | 166.518 | .233 |
| EOE\_2 | <--- | EOE\_5 | 192.250 | .248 |
| EOE\_2 | <--- | EOE\_4 | 220.045 | .271 |
| EOE\_2 | <--- | EOE\_3 | 227.488 | .272 |
| EOE\_2 | <--- | EOE\_1 | 226.847 | .271 |
| EOE\_2 | <--- | EE\_20 | 12.441 | -.062 |
| EOE\_2 | <--- | EE\_19 | 11.400 | -.060 |
| EOE\_2 | <--- | EE\_18 | 12.691 | -.063 |
| EOE\_2 | <--- | EE\_17 | 19.927 | -.077 |
| EOE\_2 | <--- | EE\_16 | 27.031 | -.089 |
| EOE\_2 | <--- | EE\_15 | 17.746 | -.072 |
| EOE\_2 | <--- | EE\_14 | 34.020 | -.100 |
| EOE\_2 | <--- | EE\_13 | 31.723 | -.096 |
| EOE\_2 | <--- | EE\_12 | 26.842 | -.088 |
| EOE\_2 | <--- | EE\_11 | 35.345 | -.100 |
| EOE\_2 | <--- | EE\_10 | 34.066 | -.098 |
| EOE\_2 | <--- | EE\_9 | 31.535 | -.095 |
| EOE\_2 | <--- | EE\_8 | 29.227 | -.091 |
| EOE\_2 | <--- | EE\_7 | 15.142 | -.065 |
| EOE\_2 | <--- | EE\_6 | 42.454 | -.110 |
| EOE\_2 | <--- | EE\_5 | 29.662 | -.091 |
| EOE\_2 | <--- | EE\_4 | 44.974 | -.113 |
| EOE\_2 | <--- | EE\_3 | 51.678 | -.120 |
| EOE\_2 | <--- | EE\_2 | 16.560 | -.068 |
| EOE\_2 | <--- | EE\_1 | 31.912 | -.096 |
| EOE\_1 | <--- | EOE\_9 | 165.545 | .230 |
| EOE\_1 | <--- | EOE\_8 | 198.857 | .250 |
| EOE\_1 | <--- | EOE\_7 | 175.773 | .240 |
| EOE\_1 | <--- | EOE\_6 | 180.203 | .242 |
| EOE\_1 | <--- | EOE\_5 | 163.425 | .228 |
| EOE\_1 | <--- | EOE\_4 | 182.184 | .246 |
| EOE\_1 | <--- | EOE\_3 | 212.329 | .262 |
| EOE\_1 | <--- | EOE\_2 | 226.388 | .270 |
| EOE\_1 | <--- | EE\_20 | 25.873 | -.089 |
| EOE\_1 | <--- | EE\_19 | 13.998 | -.066 |
| EOE\_1 | <--- | EE\_18 | 25.994 | -.090 |
| EOE\_1 | <--- | EE\_17 | 22.527 | -.082 |
| EOE\_1 | <--- | EE\_16 | 28.329 | -.091 |
| EOE\_1 | <--- | EE\_15 | 29.147 | -.093 |
| EOE\_1 | <--- | EE\_14 | 29.589 | -.093 |
| EOE\_1 | <--- | EE\_13 | 22.631 | -.081 |
| EOE\_1 | <--- | EE\_12 | 18.548 | -.073 |
| EOE\_1 | <--- | EE\_11 | 28.150 | -.089 |
| EOE\_1 | <--- | EE\_10 | 29.141 | -.091 |
| EOE\_1 | <--- | EE\_9 | 23.855 | -.082 |
| EOE\_1 | <--- | EE\_8 | 23.237 | -.081 |
| EOE\_1 | <--- | EE\_7 | 20.724 | -.076 |
| EOE\_1 | <--- | EE\_6 | 32.527 | -.096 |
| EOE\_1 | <--- | EE\_5 | 25.924 | -.085 |
| EOE\_1 | <--- | EE\_4 | 39.445 | -.106 |
| EOE\_1 | <--- | EE\_3 | 26.659 | -.086 |
| EOE\_1 | <--- | EE\_2 | 19.859 | -.074 |
| EOE\_1 | <--- | EE\_1 | 31.618 | -.095 |
| AISE\_6 | <--- | AISE\_5 | 281.720 | .317 |
| AISE\_6 | <--- | AISE\_4 | 229.497 | .271 |
| AISE\_6 | <--- | AISE\_3 | 205.211 | .260 |
| AISE\_6 | <--- | AISE\_2 | 235.919 | .280 |
| AISE\_6 | <--- | AISE\_1 | 236.419 | .262 |
| AISE\_6 | <--- | ESE\_10 | 8.544 | .072 |
| AISE\_6 | <--- | ESE\_8 | 5.876 | .060 |
| AISE\_6 | <--- | ESE\_5 | 5.059 | .055 |
| AISE\_6 | <--- | ESE\_1 | 4.010 | .048 |
| AISE\_6 | <--- | EE\_20 | 7.661 | -.069 |
| AISE\_6 | <--- | EE\_19 | 10.799 | -.083 |
| AISE\_6 | <--- | EE\_18 | 17.146 | -.105 |
| AISE\_6 | <--- | EE\_17 | 13.485 | -.090 |
| AISE\_6 | <--- | EE\_16 | 32.584 | -.139 |
| AISE\_6 | <--- | EE\_15 | 25.292 | -.123 |
| AISE\_6 | <--- | EE\_14 | 8.375 | -.071 |
| AISE\_6 | <--- | EE\_13 | 22.236 | -.114 |
| AISE\_6 | <--- | EE\_12 | 28.131 | -.129 |
| AISE\_6 | <--- | EE\_11 | 16.440 | -.098 |
| AISE\_6 | <--- | EE\_10 | 13.853 | -.089 |
| AISE\_6 | <--- | EE\_9 | 31.138 | -.134 |
| AISE\_6 | <--- | EE\_8 | 22.019 | -.113 |
| AISE\_6 | <--- | EE\_7 | 17.286 | -.099 |
| AISE\_6 | <--- | EE\_6 | 12.061 | -.083 |
| AISE\_6 | <--- | EE\_5 | 22.916 | -.114 |
| AISE\_6 | <--- | EE\_4 | 6.283 | -.060 |
| AISE\_6 | <--- | EE\_3 | 17.695 | -.100 |
| AISE\_6 | <--- | EE\_2 | 19.589 | -.105 |
| AISE\_6 | <--- | EE\_1 | 16.004 | -.097 |
| AISE\_5 | <--- | AISE\_6 | 288.558 | .283 |
| AISE\_5 | <--- | AISE\_4 | 242.642 | .260 |
| AISE\_5 | <--- | AISE\_3 | 205.316 | .244 |
| AISE\_5 | <--- | AISE\_2 | 273.646 | .282 |
| AISE\_5 | <--- | AISE\_1 | 182.736 | .216 |
| AISE\_5 | <--- | EE\_20 | 12.723 | -.083 |
| AISE\_5 | <--- | EE\_19 | 17.796 | -.099 |
| AISE\_5 | <--- | EE\_18 | 20.988 | -.108 |
| AISE\_5 | <--- | EE\_17 | 11.796 | -.079 |
| AISE\_5 | <--- | EE\_16 | 22.040 | -.107 |
| AISE\_5 | <--- | EE\_15 | 15.443 | -.090 |
| AISE\_5 | <--- | EE\_14 | 7.650 | -.063 |
| AISE\_5 | <--- | EE\_13 | 15.692 | -.090 |
| AISE\_5 | <--- | EE\_12 | 19.809 | -.101 |
| AISE\_5 | <--- | EE\_11 | 24.678 | -.112 |
| AISE\_5 | <--- | EE\_10 | 17.891 | -.095 |
| AISE\_5 | <--- | EE\_9 | 29.462 | -.122 |
| AISE\_5 | <--- | EE\_8 | 12.685 | -.080 |
| AISE\_5 | <--- | EE\_7 | 21.490 | -.104 |
| AISE\_5 | <--- | EE\_6 | 19.640 | -.100 |
| AISE\_5 | <--- | EE\_5 | 22.111 | -.105 |
| AISE\_5 | <--- | EE\_4 | 16.098 | -.090 |
| AISE\_5 | <--- | EE\_3 | 23.779 | -.109 |
| AISE\_5 | <--- | EE\_2 | 18.485 | -.096 |
| AISE\_5 | <--- | EE\_1 | 17.037 | -.093 |
| AISE\_4 | <--- | AISE\_6 | 231.145 | .270 |
| AISE\_4 | <--- | AISE\_5 | 238.593 | .290 |
| AISE\_4 | <--- | AISE\_3 | 235.053 | .277 |
| AISE\_4 | <--- | AISE\_2 | 221.720 | .270 |
| AISE\_4 | <--- | AISE\_1 | 219.300 | .252 |
| AISE\_4 | <--- | EE\_20 | 9.446 | -.077 |
| AISE\_4 | <--- | EE\_19 | 9.275 | -.076 |
| AISE\_4 | <--- | EE\_18 | 10.592 | -.082 |
| AISE\_4 | <--- | EE\_17 | 27.897 | -.129 |
| AISE\_4 | <--- | EE\_16 | 54.220 | -.178 |
| AISE\_4 | <--- | EE\_15 | 26.284 | -.125 |
| AISE\_4 | <--- | EE\_14 | 12.764 | -.087 |
| AISE\_4 | <--- | EE\_13 | 29.573 | -.131 |
| AISE\_4 | <--- | EE\_12 | 13.117 | -.087 |
| AISE\_4 | <--- | EE\_11 | 24.831 | -.119 |
| AISE\_4 | <--- | EE\_10 | 21.857 | -.112 |
| AISE\_4 | <--- | EE\_9 | 30.877 | -.133 |
| AISE\_4 | <--- | EE\_8 | 26.339 | -.123 |
| AISE\_4 | <--- | EE\_7 | 16.002 | -.095 |
| AISE\_4 | <--- | EE\_6 | 12.613 | -.085 |
| AISE\_4 | <--- | EE\_5 | 13.735 | -.088 |
| AISE\_4 | <--- | EE\_4 | 6.369 | -.061 |
| AISE\_4 | <--- | EE\_3 | 18.417 | -.102 |
| AISE\_4 | <--- | EE\_2 | 18.750 | -.103 |
| AISE\_4 | <--- | EE\_1 | 13.235 | -.088 |
| AISE\_3 | <--- | AISE\_6 | 203.991 | .251 |
| AISE\_3 | <--- | AISE\_5 | 199.259 | .262 |
| AISE\_3 | <--- | AISE\_4 | 231.990 | .268 |
| AISE\_3 | <--- | AISE\_2 | 238.232 | .277 |
| AISE\_3 | <--- | AISE\_1 | 225.029 | .252 |
| AISE\_3 | <--- | ESE\_5 | 5.022 | .054 |
| AISE\_3 | <--- | EE\_20 | 14.897 | -.095 |
| AISE\_3 | <--- | EE\_19 | 18.919 | -.108 |
| AISE\_3 | <--- | EE\_18 | 17.973 | -.105 |
| AISE\_3 | <--- | EE\_17 | 21.551 | -.112 |
| AISE\_3 | <--- | EE\_16 | 23.588 | -.116 |
| AISE\_3 | <--- | EE\_15 | 13.505 | -.089 |
| AISE\_3 | <--- | EE\_14 | 12.823 | -.086 |
| AISE\_3 | <--- | EE\_13 | 20.211 | -.107 |
| AISE\_3 | <--- | EE\_12 | 22.401 | -.113 |
| AISE\_3 | <--- | EE\_11 | 21.058 | -.109 |
| AISE\_3 | <--- | EE\_10 | 19.217 | -.103 |
| AISE\_3 | <--- | EE\_9 | 31.989 | -.134 |
| AISE\_3 | <--- | EE\_8 | 24.282 | -.117 |
| AISE\_3 | <--- | EE\_7 | 26.783 | -.122 |
| AISE\_3 | <--- | EE\_6 | 24.952 | -.118 |
| AISE\_3 | <--- | EE\_5 | 13.669 | -.086 |
| AISE\_3 | <--- | EE\_4 | 12.116 | -.082 |
| AISE\_3 | <--- | EE\_3 | 15.136 | -.091 |
| AISE\_3 | <--- | EE\_2 | 41.271 | -.150 |
| AISE\_3 | <--- | EE\_1 | 17.646 | -.100 |
| AISE\_2 | <--- | AISE\_6 | 237.820 | .268 |
| AISE\_2 | <--- | AISE\_5 | 269.314 | .302 |
| AISE\_2 | <--- | AISE\_4 | 221.913 | .259 |
| AISE\_2 | <--- | AISE\_3 | 241.587 | .275 |
| AISE\_2 | <--- | AISE\_1 | 231.803 | .253 |
| AISE\_2 | <--- | EE\_20 | 10.716 | -.080 |
| AISE\_2 | <--- | EE\_19 | 10.279 | -.079 |
| AISE\_2 | <--- | EE\_18 | 16.970 | -.101 |
| AISE\_2 | <--- | EE\_17 | 25.351 | -.121 |
| AISE\_2 | <--- | EE\_16 | 24.913 | -.118 |
| AISE\_2 | <--- | EE\_15 | 18.651 | -.103 |
| AISE\_2 | <--- | EE\_14 | 9.979 | -.075 |
| AISE\_2 | <--- | EE\_13 | 16.754 | -.097 |
| AISE\_2 | <--- | EE\_12 | 9.924 | -.074 |
| AISE\_2 | <--- | EE\_11 | 19.665 | -.104 |
| AISE\_2 | <--- | EE\_10 | 18.229 | -.100 |
| AISE\_2 | <--- | EE\_9 | 29.067 | -.126 |
| AISE\_2 | <--- | EE\_8 | 17.130 | -.097 |
| AISE\_2 | <--- | EE\_7 | 19.401 | -.103 |
| AISE\_2 | <--- | EE\_6 | 12.881 | -.084 |
| AISE\_2 | <--- | EE\_5 | 26.379 | -.119 |
| AISE\_2 | <--- | EE\_4 | 13.638 | -.087 |
| AISE\_2 | <--- | EE\_3 | 18.026 | -.099 |
| AISE\_2 | <--- | EE\_2 | 23.871 | -.113 |
| AISE\_2 | <--- | EE\_1 | 21.914 | -.110 |
| AISE\_1 | <--- | AISE\_6 | 229.967 | .286 |
| AISE\_1 | <--- | AISE\_5 | 173.536 | .263 |
| AISE\_1 | <--- | AISE\_4 | 211.795 | .275 |
| AISE\_1 | <--- | AISE\_3 | 220.197 | .285 |
| AISE\_1 | <--- | AISE\_2 | 223.674 | .288 |
| AISE\_1 | <--- | ESE\_10 | 8.429 | .076 |
| AISE\_1 | <--- | ESE\_8 | 5.644 | .062 |
| AISE\_1 | <--- | EE\_20 | 11.374 | -.089 |
| AISE\_1 | <--- | EE\_19 | 16.408 | -.108 |
| AISE\_1 | <--- | EE\_18 | 11.967 | -.092 |
| AISE\_1 | <--- | EE\_17 | 15.977 | -.104 |
| AISE\_1 | <--- | EE\_16 | 28.783 | -.138 |
| AISE\_1 | <--- | EE\_15 | 14.462 | -.098 |
| AISE\_1 | <--- | EE\_14 | 12.631 | -.092 |
| AISE\_1 | <--- | EE\_13 | 17.091 | -.106 |
| AISE\_1 | <--- | EE\_12 | 17.532 | -.107 |
| AISE\_1 | <--- | EE\_11 | 19.416 | -.112 |
| AISE\_1 | <--- | EE\_10 | 12.906 | -.091 |
| AISE\_1 | <--- | EE\_9 | 18.533 | -.110 |
| AISE\_1 | <--- | EE\_8 | 21.775 | -.119 |
| AISE\_1 | <--- | EE\_7 | 16.273 | -.102 |
| AISE\_1 | <--- | EE\_6 | 13.373 | -.093 |
| AISE\_1 | <--- | EE\_5 | 22.309 | -.119 |
| AISE\_1 | <--- | EE\_4 | 5.654 | -.061 |
| AISE\_1 | <--- | EE\_3 | 14.025 | -.094 |
| AISE\_1 | <--- | EE\_2 | 21.897 | -.118 |
| AISE\_1 | <--- | EE\_1 | 14.395 | -.097 |
| ESE\_10 | <--- | AISE\_6 | 7.623 | .037 |
| ESE\_10 | <--- | AISE\_1 | 7.731 | .036 |
| ESE\_10 | <--- | ESE\_9 | 299.649 | .328 |
| ESE\_10 | <--- | ESE\_8 | 330.016 | .338 |
| ESE\_10 | <--- | ESE\_7 | 360.773 | .354 |
| ESE\_10 | <--- | ESE\_6 | 73.403 | .154 |
| ESE\_10 | <--- | ESE\_5 | 68.044 | .152 |
| ESE\_10 | <--- | ESE\_4 | 74.684 | .157 |
| ESE\_10 | <--- | ESE\_3 | 42.878 | .122 |
| ESE\_10 | <--- | ESE\_2 | 55.275 | .136 |
| ESE\_10 | <--- | ESE\_1 | 65.551 | .148 |
| ESE\_10 | <--- | EE\_20 | 13.043 | -.068 |
| ESE\_10 | <--- | EE\_19 | 11.489 | -.064 |
| ESE\_10 | <--- | EE\_18 | 5.036 | -.043 |
| ESE\_10 | <--- | EE\_17 | 13.771 | -.069 |
| ESE\_10 | <--- | EE\_16 | 5.782 | -.044 |
| ESE\_10 | <--- | EE\_15 | 16.403 | -.075 |
| ESE\_10 | <--- | EE\_14 | 7.034 | -.049 |
| ESE\_10 | <--- | EE\_13 | 13.183 | -.067 |
| ESE\_10 | <--- | EE\_12 | 16.500 | -.075 |
| ESE\_10 | <--- | EE\_11 | 11.647 | -.062 |
| ESE\_10 | <--- | EE\_10 | 21.077 | -.083 |
| ESE\_10 | <--- | EE\_9 | 15.984 | -.073 |
| ESE\_10 | <--- | EE\_8 | 6.590 | -.047 |
| ESE\_10 | <--- | EE\_7 | 8.248 | -.052 |
| ESE\_10 | <--- | EE\_6 | 10.628 | -.059 |
| ESE\_10 | <--- | EE\_5 | 15.357 | -.070 |
| ESE\_10 | <--- | EE\_4 | 18.130 | -.078 |
| ESE\_10 | <--- | EE\_3 | 7.495 | -.049 |
| ESE\_10 | <--- | EE\_2 | 13.395 | -.066 |
| ESE\_10 | <--- | EE\_1 | 15.803 | -.073 |
| ESE\_9 | <--- | ESE\_10 | 288.020 | .320 |
| ESE\_9 | <--- | ESE\_8 | 340.991 | .346 |
| ESE\_9 | <--- | ESE\_7 | 369.663 | .360 |
| ESE\_9 | <--- | ESE\_6 | 46.773 | .124 |
| ESE\_9 | <--- | ESE\_5 | 41.524 | .119 |
| ESE\_9 | <--- | ESE\_4 | 52.205 | .132 |
| ESE\_9 | <--- | ESE\_3 | 43.213 | .123 |
| ESE\_9 | <--- | ESE\_2 | 56.187 | .138 |
| ESE\_9 | <--- | ESE\_1 | 39.763 | .116 |
| ESE\_9 | <--- | EE\_19 | 13.681 | -.071 |
| ESE\_9 | <--- | EE\_18 | 8.486 | -.056 |
| ESE\_9 | <--- | EE\_17 | 14.230 | -.071 |
| ESE\_9 | <--- | EE\_16 | 12.500 | -.066 |
| ESE\_9 | <--- | EE\_15 | 15.670 | -.074 |
| ESE\_9 | <--- | EE\_14 | 15.408 | -.073 |
| ESE\_9 | <--- | EE\_13 | 11.220 | -.062 |
| ESE\_9 | <--- | EE\_12 | 14.902 | -.071 |
| ESE\_9 | <--- | EE\_11 | 12.064 | -.064 |
| ESE\_9 | <--- | EE\_10 | 11.222 | -.061 |
| ESE\_9 | <--- | EE\_9 | 17.497 | -.077 |
| ESE\_9 | <--- | EE\_8 | 5.958 | -.045 |
| ESE\_9 | <--- | EE\_7 | 13.704 | -.067 |
| ESE\_9 | <--- | EE\_6 | 14.746 | -.070 |
| ESE\_9 | <--- | EE\_5 | 7.525 | -.050 |
| ESE\_9 | <--- | EE\_4 | 22.015 | -.086 |
| ESE\_9 | <--- | EE\_3 | 10.154 | -.058 |
| ESE\_9 | <--- | EE\_2 | 8.613 | -.053 |
| ESE\_9 | <--- | EE\_1 | 12.864 | -.066 |
| ESE\_8 | <--- | EOE\_6 | 5.319 | -.045 |
| ESE\_8 | <--- | EOE\_5 | 7.675 | -.054 |
| ESE\_8 | <--- | EOE\_3 | 4.951 | -.044 |
| ESE\_8 | <--- | AISE\_6 | 5.191 | .031 |
| ESE\_8 | <--- | AISE\_1 | 5.126 | .030 |
| ESE\_8 | <--- | ESE\_10 | 326.794 | .342 |
| ESE\_8 | <--- | ESE\_9 | 351.294 | .359 |
| ESE\_8 | <--- | ESE\_7 | 390.742 | .372 |
| ESE\_8 | <--- | ESE\_6 | 47.256 | .125 |
| ESE\_8 | <--- | ESE\_5 | 80.887 | .167 |
| ESE\_8 | <--- | ESE\_4 | 52.072 | .132 |
| ESE\_8 | <--- | ESE\_3 | 46.046 | .128 |
| ESE\_8 | <--- | ESE\_2 | 60.573 | .144 |
| ESE\_8 | <--- | ESE\_1 | 35.740 | .111 |
| ESE\_8 | <--- | EE\_20 | 8.802 | -.057 |
| ESE\_8 | <--- | EE\_19 | 9.985 | -.061 |
| ESE\_8 | <--- | EE\_18 | 4.462 | -.041 |
| ESE\_8 | <--- | EE\_17 | 12.116 | -.066 |
| ESE\_8 | <--- | EE\_16 | 20.263 | -.084 |
| ESE\_8 | <--- | EE\_15 | 11.879 | -.065 |
| ESE\_8 | <--- | EE\_14 | 13.638 | -.069 |
| ESE\_8 | <--- | EE\_13 | 15.699 | -.074 |
| ESE\_8 | <--- | EE\_12 | 4.393 | -.039 |
| ESE\_8 | <--- | EE\_11 | 9.971 | -.058 |
| ESE\_8 | <--- | EE\_10 | 8.529 | -.054 |
| ESE\_8 | <--- | EE\_9 | 16.099 | -.074 |
| ESE\_8 | <--- | EE\_8 | 7.417 | -.050 |
| ESE\_8 | <--- | EE\_7 | 11.401 | -.062 |
| ESE\_8 | <--- | EE\_6 | 13.912 | -.069 |
| ESE\_8 | <--- | EE\_5 | 12.882 | -.065 |
| ESE\_8 | <--- | EE\_4 | 16.671 | -.075 |
| ESE\_8 | <--- | EE\_2 | 8.840 | -.054 |
| ESE\_8 | <--- | EE\_1 | 7.587 | -.051 |
| ESE\_7 | <--- | ESE\_10 | 344.667 | .357 |
| ESE\_7 | <--- | ESE\_9 | 367.418 | .372 |
| ESE\_7 | <--- | ESE\_8 | 376.979 | .370 |
| ESE\_7 | <--- | ESE\_6 | 59.316 | .142 |
| ESE\_7 | <--- | ESE\_5 | 79.502 | .168 |
| ESE\_7 | <--- | ESE\_4 | 60.412 | .145 |
| ESE\_7 | <--- | ESE\_3 | 63.887 | .153 |
| ESE\_7 | <--- | ESE\_2 | 73.813 | .162 |
| ESE\_7 | <--- | ESE\_1 | 59.983 | .145 |
| ESE\_7 | <--- | EE\_20 | 10.387 | -.063 |
| ESE\_7 | <--- | EE\_19 | 11.522 | -.066 |
| ESE\_7 | <--- | EE\_18 | 12.217 | -.069 |
| ESE\_7 | <--- | EE\_17 | 16.872 | -.078 |
| ESE\_7 | <--- | EE\_16 | 10.387 | -.061 |
| ESE\_7 | <--- | EE\_15 | 16.755 | -.078 |
| ESE\_7 | <--- | EE\_14 | 16.756 | -.078 |
| ESE\_7 | <--- | EE\_13 | 12.479 | -.067 |
| ESE\_7 | <--- | EE\_12 | 16.595 | -.077 |
| ESE\_7 | <--- | EE\_11 | 16.679 | -.076 |
| ESE\_7 | <--- | EE\_10 | 15.031 | -.072 |
| ESE\_7 | <--- | EE\_9 | 14.414 | -.071 |
| ESE\_7 | <--- | EE\_8 | 11.788 | -.064 |
| ESE\_7 | <--- | EE\_7 | 14.677 | -.071 |
| ESE\_7 | <--- | EE\_6 | 16.913 | -.077 |
| ESE\_7 | <--- | EE\_5 | 15.466 | -.073 |
| ESE\_7 | <--- | EE\_4 | 20.489 | -.085 |
| ESE\_7 | <--- | EE\_3 | 5.115 | -.042 |
| ESE\_7 | <--- | EE\_2 | 15.754 | -.073 |
| ESE\_7 | <--- | EE\_1 | 8.708 | -.055 |
| ESE\_6 | <--- | ESE\_10 | 72.769 | .167 |
| ESE\_6 | <--- | ESE\_9 | 48.241 | .137 |
| ESE\_6 | <--- | ESE\_8 | 47.310 | .133 |
| ESE\_6 | <--- | ESE\_7 | 61.552 | .152 |
| ESE\_6 | <--- | ESE\_5 | 301.242 | .333 |
| ESE\_6 | <--- | ESE\_4 | 293.048 | .324 |
| ESE\_6 | <--- | ESE\_3 | 293.167 | .333 |
| ESE\_6 | <--- | ESE\_2 | 318.660 | .342 |
| ESE\_6 | <--- | ESE\_1 | 272.829 | .315 |
| ESE\_6 | <--- | EE\_20 | 17.717 | -.083 |
| ESE\_6 | <--- | EE\_19 | 11.887 | -.068 |
| ESE\_6 | <--- | EE\_18 | 11.214 | -.067 |
| ESE\_6 | <--- | EE\_17 | 18.042 | -.083 |
| ESE\_6 | <--- | EE\_16 | 13.392 | -.070 |
| ESE\_6 | <--- | EE\_15 | 33.897 | -.113 |
| ESE\_6 | <--- | EE\_14 | 12.816 | -.069 |
| ESE\_6 | <--- | EE\_13 | 12.772 | -.068 |
| ESE\_6 | <--- | EE\_12 | 11.305 | -.064 |
| ESE\_6 | <--- | EE\_11 | 31.541 | -.107 |
| ESE\_6 | <--- | EE\_10 | 16.817 | -.078 |
| ESE\_6 | <--- | EE\_9 | 29.272 | -.103 |
| ESE\_6 | <--- | EE\_8 | 20.927 | -.087 |
| ESE\_6 | <--- | EE\_7 | 20.182 | -.085 |
| ESE\_6 | <--- | EE\_6 | 18.045 | -.081 |
| ESE\_6 | <--- | EE\_5 | 24.839 | -.094 |
| ESE\_6 | <--- | EE\_4 | 15.345 | -.074 |
| ESE\_6 | <--- | EE\_3 | 23.827 | -.092 |
| ESE\_6 | <--- | EE\_2 | 12.089 | -.065 |
| ESE\_6 | <--- | EE\_1 | 16.774 | -.078 |
| ESE\_5 | <--- | AISE\_6 | 4.503 | .029 |
| ESE\_5 | <--- | AISE\_3 | 4.497 | .030 |
| ESE\_5 | <--- | ESE\_10 | 67.879 | .157 |
| ESE\_5 | <--- | ESE\_9 | 43.096 | .127 |
| ESE\_5 | <--- | ESE\_8 | 81.487 | .171 |
| ESE\_5 | <--- | ESE\_7 | 83.016 | .173 |
| ESE\_5 | <--- | ESE\_6 | 303.131 | .320 |
| ESE\_5 | <--- | ESE\_4 | 313.851 | .328 |
| ESE\_5 | <--- | ESE\_3 | 315.145 | .338 |
| ESE\_5 | <--- | ESE\_2 | 330.495 | .340 |
| ESE\_5 | <--- | ESE\_1 | 306.039 | .326 |
| ESE\_5 | <--- | EE\_20 | 18.989 | -.084 |
| ESE\_5 | <--- | EE\_19 | 13.998 | -.073 |
| ESE\_5 | <--- | EE\_18 | 16.165 | -.078 |
| ESE\_5 | <--- | EE\_17 | 24.786 | -.095 |
| ESE\_5 | <--- | EE\_16 | 10.225 | -.060 |
| ESE\_5 | <--- | EE\_15 | 26.333 | -.097 |
| ESE\_5 | <--- | EE\_14 | 26.759 | -.097 |
| ESE\_5 | <--- | EE\_13 | 8.483 | -.055 |
| ESE\_5 | <--- | EE\_12 | 19.452 | -.083 |
| ESE\_5 | <--- | EE\_11 | 28.367 | -.099 |
| ESE\_5 | <--- | EE\_10 | 16.994 | -.076 |
| ESE\_5 | <--- | EE\_9 | 22.223 | -.088 |
| ESE\_5 | <--- | EE\_8 | 15.558 | -.073 |
| ESE\_5 | <--- | EE\_7 | 20.494 | -.083 |
| ESE\_5 | <--- | EE\_6 | 21.304 | -.086 |
| ESE\_5 | <--- | EE\_5 | 31.476 | -.103 |
| ESE\_5 | <--- | EE\_4 | 23.915 | -.091 |
| ESE\_5 | <--- | EE\_3 | 18.637 | -.079 |
| ESE\_5 | <--- | EE\_2 | 14.979 | -.071 |
| ESE\_5 | <--- | EE\_1 | 14.940 | -.072 |
| ESE\_4 | <--- | ESE\_10 | 74.529 | .167 |
| ESE\_4 | <--- | ESE\_9 | 54.200 | .144 |
| ESE\_4 | <--- | ESE\_8 | 52.476 | .139 |
| ESE\_4 | <--- | ESE\_7 | 63.103 | .153 |
| ESE\_4 | <--- | ESE\_6 | 294.986 | .320 |
| ESE\_4 | <--- | ESE\_5 | 313.958 | .336 |
| ESE\_4 | <--- | ESE\_3 | 339.001 | .355 |
| ESE\_4 | <--- | ESE\_2 | 312.072 | .335 |
| ESE\_4 | <--- | ESE\_1 | 327.374 | .342 |
| ESE\_4 | <--- | EE\_20 | 18.298 | -.084 |
| ESE\_4 | <--- | EE\_19 | 17.693 | -.083 |
| ESE\_4 | <--- | EE\_18 | 21.189 | -.091 |
| ESE\_4 | <--- | EE\_17 | 22.500 | -.091 |
| ESE\_4 | <--- | EE\_16 | 12.871 | -.068 |
| ESE\_4 | <--- | EE\_15 | 24.373 | -.094 |
| ESE\_4 | <--- | EE\_14 | 21.368 | -.088 |
| ESE\_4 | <--- | EE\_13 | 8.480 | -.055 |
| ESE\_4 | <--- | EE\_12 | 24.339 | -.093 |
| ESE\_4 | <--- | EE\_11 | 19.465 | -.083 |
| ESE\_4 | <--- | EE\_10 | 14.334 | -.071 |
| ESE\_4 | <--- | EE\_9 | 14.833 | -.072 |
| ESE\_4 | <--- | EE\_8 | 8.318 | -.054 |
| ESE\_4 | <--- | EE\_7 | 13.798 | -.069 |
| ESE\_4 | <--- | EE\_6 | 20.982 | -.086 |
| ESE\_4 | <--- | EE\_5 | 27.076 | -.097 |
| ESE\_4 | <--- | EE\_4 | 27.316 | -.098 |
| ESE\_4 | <--- | EE\_3 | 31.455 | -.105 |
| ESE\_4 | <--- | EE\_2 | 21.280 | -.086 |
| ESE\_4 | <--- | EE\_1 | 15.009 | -.073 |
| ESE\_3 | <--- | EOE\_9 | 4.110 | -.040 |
| ESE\_3 | <--- | ESE\_10 | 41.792 | .123 |
| ESE\_3 | <--- | ESE\_9 | 43.819 | .127 |
| ESE\_3 | <--- | ESE\_8 | 45.322 | .127 |
| ESE\_3 | <--- | ESE\_7 | 65.178 | .153 |
| ESE\_3 | <--- | ESE\_6 | 288.227 | .311 |
| ESE\_3 | <--- | ESE\_5 | 307.904 | .328 |
| ESE\_3 | <--- | ESE\_4 | 331.100 | .335 |
| ESE\_3 | <--- | ESE\_2 | 282.423 | .313 |
| ESE\_3 | <--- | ESE\_1 | 269.732 | .305 |
| ESE\_3 | <--- | EE\_20 | 8.277 | -.055 |
| ESE\_3 | <--- | EE\_19 | 10.730 | -.063 |
| ESE\_3 | <--- | EE\_18 | 19.721 | -.086 |
| ESE\_3 | <--- | EE\_17 | 22.828 | -.090 |
| ESE\_3 | <--- | EE\_16 | 10.991 | -.062 |
| ESE\_3 | <--- | EE\_15 | 30.965 | -.105 |
| ESE\_3 | <--- | EE\_14 | 11.736 | -.064 |
| ESE\_3 | <--- | EE\_13 | 7.128 | -.050 |
| ESE\_3 | <--- | EE\_12 | 21.114 | -.086 |
| ESE\_3 | <--- | EE\_11 | 28.198 | -.098 |
| ESE\_3 | <--- | EE\_10 | 15.582 | -.073 |
| ESE\_3 | <--- | EE\_9 | 21.120 | -.085 |
| ESE\_3 | <--- | EE\_8 | 21.991 | -.087 |
| ESE\_3 | <--- | EE\_7 | 19.068 | -.080 |
| ESE\_3 | <--- | EE\_6 | 19.214 | -.081 |
| ESE\_3 | <--- | EE\_5 | 29.312 | -.099 |
| ESE\_3 | <--- | EE\_4 | 21.763 | -.086 |
| ESE\_3 | <--- | EE\_3 | 15.677 | -.073 |
| ESE\_3 | <--- | EE\_2 | 20.054 | -.082 |
| ESE\_3 | <--- | EE\_1 | 12.228 | -.065 |
| ESE\_2 | <--- | EOE\_7 | 4.003 | -.040 |
| ESE\_2 | <--- | ESE\_10 | 54.821 | .142 |
| ESE\_2 | <--- | ESE\_9 | 57.976 | .148 |
| ESE\_2 | <--- | ESE\_8 | 60.669 | .149 |
| ESE\_2 | <--- | ESE\_7 | 76.629 | .167 |
| ESE\_2 | <--- | ESE\_6 | 318.801 | .330 |
| ESE\_2 | <--- | ESE\_5 | 328.581 | .342 |
| ESE\_2 | <--- | ESE\_4 | 310.159 | .328 |
| ESE\_2 | <--- | ESE\_3 | 287.390 | .324 |
| ESE\_2 | <--- | ESE\_1 | 312.011 | .331 |
| ESE\_2 | <--- | EE\_20 | 16.000 | -.078 |
| ESE\_2 | <--- | EE\_19 | 7.413 | -.053 |
| ESE\_2 | <--- | EE\_18 | 16.950 | -.081 |
| ESE\_2 | <--- | EE\_17 | 19.642 | -.085 |
| ESE\_2 | <--- | EE\_16 | 14.024 | -.071 |
| ESE\_2 | <--- | EE\_15 | 25.678 | -.096 |
| ESE\_2 | <--- | EE\_14 | 13.149 | -.069 |
| ESE\_2 | <--- | EE\_13 | 19.459 | -.083 |
| ESE\_2 | <--- | EE\_12 | 15.765 | -.075 |
| ESE\_2 | <--- | EE\_11 | 30.049 | -.102 |
| ESE\_2 | <--- | EE\_10 | 15.095 | -.072 |
| ESE\_2 | <--- | EE\_9 | 17.962 | -.079 |
| ESE\_2 | <--- | EE\_8 | 14.086 | -.070 |
| ESE\_2 | <--- | EE\_7 | 10.186 | -.059 |
| ESE\_2 | <--- | EE\_6 | 15.387 | -.073 |
| ESE\_2 | <--- | EE\_5 | 29.065 | -.099 |
| ESE\_2 | <--- | EE\_4 | 18.658 | -.081 |
| ESE\_2 | <--- | EE\_3 | 19.126 | -.081 |
| ESE\_2 | <--- | EE\_2 | 16.714 | -.075 |
| ESE\_2 | <--- | EE\_1 | 15.133 | -.073 |
| ESE\_1 | <--- | ESE\_10 | 63.850 | .155 |
| ESE\_1 | <--- | ESE\_9 | 40.294 | .125 |
| ESE\_1 | <--- | ESE\_8 | 35.156 | .114 |
| ESE\_1 | <--- | ESE\_7 | 61.156 | .151 |
| ESE\_1 | <--- | ESE\_6 | 268.062 | .306 |
| ESE\_1 | <--- | ESE\_5 | 298.818 | .329 |
| ESE\_1 | <--- | ESE\_4 | 319.541 | .336 |
| ESE\_1 | <--- | ESE\_3 | 269.561 | .317 |
| ESE\_1 | <--- | ESE\_2 | 306.424 | .333 |
| ESE\_1 | <--- | EE\_20 | 28.514 | -.105 |
| ESE\_1 | <--- | EE\_19 | 9.347 | -.060 |
| ESE\_1 | <--- | EE\_18 | 20.070 | -.089 |
| ESE\_1 | <--- | EE\_17 | 27.194 | -.101 |
| ESE\_1 | <--- | EE\_16 | 15.511 | -.075 |
| ESE\_1 | <--- | EE\_15 | 33.035 | -.110 |
| ESE\_1 | <--- | EE\_14 | 17.040 | -.079 |
| ESE\_1 | <--- | EE\_13 | 17.774 | -.080 |
| ESE\_1 | <--- | EE\_12 | 26.405 | -.098 |
| ESE\_1 | <--- | EE\_11 | 28.247 | -.100 |
| ESE\_1 | <--- | EE\_10 | 14.436 | -.071 |
| ESE\_1 | <--- | EE\_9 | 17.901 | -.080 |
| ESE\_1 | <--- | EE\_8 | 25.553 | -.096 |
| ESE\_1 | <--- | EE\_7 | 12.032 | -.065 |
| ESE\_1 | <--- | EE\_6 | 17.095 | -.078 |
| ESE\_1 | <--- | EE\_5 | 36.253 | -.112 |
| ESE\_1 | <--- | EE\_4 | 30.072 | -.104 |
| ESE\_1 | <--- | EE\_3 | 31.559 | -.105 |
| ESE\_1 | <--- | EE\_2 | 33.688 | -.108 |
| ESE\_1 | <--- | EE\_1 | 9.369 | -.058 |
| EE\_20 | <--- | EOE\_9 | 9.646 | -.058 |
| EE\_20 | <--- | EOE\_8 | 21.832 | -.086 |
| EE\_20 | <--- | EOE\_7 | 14.521 | -.071 |
| EE\_20 | <--- | EOE\_6 | 22.117 | -.088 |
| EE\_20 | <--- | EOE\_5 | 20.721 | -.084 |
| EE\_20 | <--- | EOE\_4 | 15.333 | -.074 |
| EE\_20 | <--- | EOE\_3 | 16.497 | -.076 |
| EE\_20 | <--- | EOE\_2 | 12.141 | -.065 |
| EE\_20 | <--- | EOE\_1 | 25.301 | -.094 |
| EE\_20 | <--- | AISE\_6 | 7.353 | -.035 |
| EE\_20 | <--- | AISE\_5 | 11.922 | -.047 |
| EE\_20 | <--- | AISE\_4 | 9.002 | -.039 |
| EE\_20 | <--- | AISE\_3 | 14.383 | -.050 |
| EE\_20 | <--- | AISE\_2 | 10.203 | -.042 |
| EE\_20 | <--- | AISE\_1 | 11.222 | -.041 |
| EE\_20 | <--- | ESE\_10 | 14.031 | -.067 |
| EE\_20 | <--- | ESE\_8 | 9.561 | -.055 |
| EE\_20 | <--- | ESE\_7 | 11.696 | -.061 |
| EE\_20 | <--- | ESE\_6 | 19.224 | -.076 |
| EE\_20 | <--- | ESE\_5 | 20.476 | -.080 |
| EE\_20 | <--- | ESE\_4 | 19.724 | -.077 |
| EE\_20 | <--- | ESE\_3 | 9.135 | -.054 |
| EE\_20 | <--- | ESE\_2 | 17.354 | -.073 |
| EE\_20 | <--- | ESE\_1 | 31.489 | -.098 |
| EE\_20 | <--- | EE\_19 | 188.429 | .250 |
| EE\_20 | <--- | EE\_18 | 231.401 | .279 |
| EE\_20 | <--- | EE\_17 | 14.041 | .067 |
| EE\_20 | <--- | EE\_16 | 5.411 | .041 |
| EE\_20 | <--- | EE\_15 | 11.972 | .061 |
| EE\_20 | <--- | EE\_14 | 7.405 | .048 |
| EE\_20 | <--- | EE\_13 | 12.832 | .063 |
| EE\_20 | <--- | EE\_12 | 10.930 | .058 |
| EE\_20 | <--- | EE\_11 | 9.696 | .054 |
| EE\_20 | <--- | EE\_10 | 7.836 | .049 |
| EE\_20 | <--- | EE\_9 | 10.982 | .058 |
| EE\_20 | <--- | EE\_8 | 5.106 | .040 |
| EE\_20 | <--- | EE\_6 | 7.928 | .049 |
| EE\_20 | <--- | EE\_5 | 17.682 | .073 |
| EE\_20 | <--- | EE\_4 | 11.271 | .059 |
| EE\_20 | <--- | EE\_3 | 15.691 | .069 |
| EE\_20 | <--- | EE\_2 | 6.265 | .043 |
| EE\_20 | <--- | EE\_1 | 12.856 | .063 |
| EE\_19 | <--- | EOE\_9 | 6.393 | -.046 |
| EE\_19 | <--- | EOE\_8 | 14.175 | -.068 |
| EE\_19 | <--- | EOE\_7 | 10.862 | -.061 |
| EE\_19 | <--- | EOE\_6 | 6.814 | -.048 |
| EE\_19 | <--- | EOE\_5 | 7.428 | -.050 |
| EE\_19 | <--- | EOE\_4 | 9.999 | -.059 |
| EE\_19 | <--- | EOE\_3 | 18.685 | -.079 |
| EE\_19 | <--- | EOE\_2 | 11.373 | -.062 |
| EE\_19 | <--- | EOE\_1 | 13.993 | -.069 |
| EE\_19 | <--- | AISE\_6 | 10.595 | -.041 |
| EE\_19 | <--- | AISE\_5 | 17.048 | -.056 |
| EE\_19 | <--- | AISE\_4 | 9.036 | -.038 |
| EE\_19 | <--- | AISE\_3 | 18.674 | -.056 |
| EE\_19 | <--- | AISE\_2 | 10.005 | -.041 |
| EE\_19 | <--- | AISE\_1 | 16.551 | -.050 |
| EE\_19 | <--- | ESE\_10 | 12.635 | -.063 |
| EE\_19 | <--- | ESE\_9 | 15.652 | -.071 |
| EE\_19 | <--- | ESE\_8 | 11.088 | -.059 |
| EE\_19 | <--- | ESE\_7 | 13.262 | -.064 |
| EE\_19 | <--- | ESE\_6 | 13.186 | -.062 |
| EE\_19 | <--- | ESE\_5 | 15.430 | -.068 |
| EE\_19 | <--- | ESE\_4 | 19.497 | -.076 |
| EE\_19 | <--- | ESE\_3 | 12.107 | -.061 |
| EE\_19 | <--- | ESE\_2 | 8.219 | -.050 |
| EE\_19 | <--- | ESE\_1 | 10.553 | -.056 |
| EE\_19 | <--- | EE\_20 | 192.631 | .248 |
| EE\_19 | <--- | EE\_18 | 192.112 | .250 |
| EE\_19 | <--- | EE\_17 | 5.419 | .041 |
| EE\_19 | <--- | EE\_16 | 9.770 | .054 |
| EE\_19 | <--- | EE\_15 | 23.495 | .085 |
| EE\_19 | <--- | EE\_13 | 15.240 | .068 |
| EE\_19 | <--- | EE\_12 | 5.688 | .041 |
| EE\_19 | <--- | EE\_9 | 8.814 | .051 |
| EE\_19 | <--- | EE\_7 | 9.939 | .054 |
| EE\_19 | <--- | EE\_6 | 7.728 | .048 |
| EE\_19 | <--- | EE\_5 | 20.306 | .077 |
| EE\_19 | <--- | EE\_4 | 14.000 | .064 |
| EE\_19 | <--- | EE\_3 | 5.765 | .041 |
| EE\_19 | <--- | EE\_2 | 6.745 | .044 |
| EE\_19 | <--- | EE\_1 | 9.064 | .052 |
| EE\_18 | <--- | EOE\_9 | 15.224 | -.071 |
| EE\_18 | <--- | EOE\_8 | 22.682 | -.086 |
| EE\_18 | <--- | EOE\_7 | 17.806 | -.078 |
| EE\_18 | <--- | EOE\_6 | 22.897 | -.088 |
| EE\_18 | <--- | EOE\_5 | 18.276 | -.078 |
| EE\_18 | <--- | EOE\_4 | 21.602 | -.086 |
| EE\_18 | <--- | EOE\_3 | 24.324 | -.090 |
| EE\_18 | <--- | EOE\_2 | 12.586 | -.065 |
| EE\_18 | <--- | EOE\_1 | 25.832 | -.093 |
| EE\_18 | <--- | AISE\_6 | 16.724 | -.052 |
| EE\_18 | <--- | AISE\_5 | 19.986 | -.060 |
| EE\_18 | <--- | AISE\_4 | 10.258 | -.041 |
| EE\_18 | <--- | AISE\_3 | 17.635 | -.054 |
| EE\_18 | <--- | AISE\_2 | 16.419 | -.053 |
| EE\_18 | <--- | AISE\_1 | 11.999 | -.042 |
| EE\_18 | <--- | ESE\_10 | 5.505 | -.041 |
| EE\_18 | <--- | ESE\_9 | 9.651 | -.055 |
| EE\_18 | <--- | ESE\_8 | 4.925 | -.039 |
| EE\_18 | <--- | ESE\_7 | 13.979 | -.066 |
| EE\_18 | <--- | ESE\_6 | 12.366 | -.060 |
| EE\_18 | <--- | ESE\_5 | 17.714 | -.073 |
| EE\_18 | <--- | ESE\_4 | 23.211 | -.082 |
| EE\_18 | <--- | ESE\_3 | 22.118 | -.083 |
| EE\_18 | <--- | ESE\_2 | 18.682 | -.075 |
| EE\_18 | <--- | ESE\_1 | 22.524 | -.082 |
| EE\_18 | <--- | EE\_20 | 235.158 | .274 |
| EE\_18 | <--- | EE\_19 | 190.973 | .248 |
| EE\_18 | <--- | EE\_17 | 10.354 | .057 |
| EE\_18 | <--- | EE\_16 | 8.008 | .049 |
| EE\_18 | <--- | EE\_15 | 17.634 | .073 |
| EE\_18 | <--- | EE\_14 | 4.362 | .036 |
| EE\_18 | <--- | EE\_13 | 16.063 | .069 |
| EE\_18 | <--- | EE\_12 | 11.158 | .058 |
| EE\_18 | <--- | EE\_11 | 20.184 | .077 |
| EE\_18 | <--- | EE\_10 | 12.972 | .062 |
| EE\_18 | <--- | EE\_9 | 18.148 | .073 |
| EE\_18 | <--- | EE\_8 | 5.618 | .041 |
| EE\_18 | <--- | EE\_7 | 11.604 | .058 |
| EE\_18 | <--- | EE\_6 | 9.946 | .054 |
| EE\_18 | <--- | EE\_5 | 23.298 | .082 |
| EE\_18 | <--- | EE\_4 | 15.099 | .067 |
| EE\_18 | <--- | EE\_3 | 14.779 | .065 |
| EE\_18 | <--- | EE\_2 | 13.604 | .063 |
| EE\_18 | <--- | EE\_1 | 4.316 | .036 |
| EE\_17 | <--- | EOE\_9 | 19.232 | -.080 |
| EE\_17 | <--- | EOE\_8 | 23.888 | -.089 |
| EE\_17 | <--- | EOE\_7 | 17.580 | -.078 |
| EE\_17 | <--- | EOE\_6 | 29.019 | -.099 |
| EE\_17 | <--- | EOE\_5 | 28.571 | -.098 |
| EE\_17 | <--- | EOE\_4 | 44.246 | -.124 |
| EE\_17 | <--- | EOE\_3 | 24.028 | -.090 |
| EE\_17 | <--- | EOE\_2 | 20.839 | -.084 |
| EE\_17 | <--- | EOE\_1 | 23.606 | -.089 |
| EE\_17 | <--- | AISE\_6 | 13.870 | -.048 |
| EE\_17 | <--- | AISE\_5 | 11.845 | -.046 |
| EE\_17 | <--- | AISE\_4 | 28.487 | -.068 |
| EE\_17 | <--- | AISE\_3 | 22.298 | -.061 |
| EE\_17 | <--- | AISE\_2 | 25.865 | -.066 |
| EE\_17 | <--- | AISE\_1 | 16.893 | -.050 |
| EE\_17 | <--- | ESE\_10 | 15.874 | -.071 |
| EE\_17 | <--- | ESE\_9 | 17.065 | -.074 |
| EE\_17 | <--- | ESE\_8 | 14.103 | -.066 |
| EE\_17 | <--- | ESE\_7 | 20.357 | -.079 |
| EE\_17 | <--- | ESE\_6 | 20.978 | -.078 |
| EE\_17 | <--- | ESE\_5 | 28.640 | -.093 |
| EE\_17 | <--- | ESE\_4 | 25.990 | -.088 |
| EE\_17 | <--- | ESE\_3 | 26.999 | -.092 |
| EE\_17 | <--- | ESE\_2 | 22.828 | -.083 |
| EE\_17 | <--- | ESE\_1 | 32.182 | -.098 |
| EE\_17 | <--- | EE\_20 | 15.046 | .069 |
| EE\_17 | <--- | EE\_19 | 5.680 | .043 |
| EE\_17 | <--- | EE\_18 | 10.918 | .060 |
| EE\_17 | <--- | EE\_16 | 215.907 | .256 |
| EE\_17 | <--- | EE\_15 | 199.082 | .247 |
| EE\_17 | <--- | EE\_14 | 178.937 | .234 |
| EE\_17 | <--- | EE\_13 | 155.934 | .217 |
| EE\_17 | <--- | EE\_12 | 6.138 | .043 |
| EE\_17 | <--- | EE\_11 | 19.649 | .076 |
| EE\_17 | <--- | EE\_10 | 11.334 | .058 |
| EE\_17 | <--- | EE\_9 | 10.012 | .055 |
| EE\_17 | <--- | EE\_8 | 12.939 | .062 |
| EE\_17 | <--- | EE\_7 | 9.602 | .053 |
| EE\_17 | <--- | EE\_6 | 11.853 | .059 |
| EE\_17 | <--- | EE\_3 | 11.476 | .058 |
| EE\_17 | <--- | EE\_2 | 4.113 | .035 |
| EE\_17 | <--- | EE\_1 | 6.190 | .043 |
| EE\_16 | <--- | EOE\_9 | 29.375 | -.101 |
| EE\_16 | <--- | EOE\_8 | 35.365 | -.110 |
| EE\_16 | <--- | EOE\_7 | 32.287 | -.107 |
| EE\_16 | <--- | EOE\_6 | 27.366 | -.098 |
| EE\_16 | <--- | EOE\_5 | 23.294 | -.090 |
| EE\_16 | <--- | EOE\_4 | 33.435 | -.109 |
| EE\_16 | <--- | EOE\_3 | 22.748 | -.089 |
| EE\_16 | <--- | EOE\_2 | 27.984 | -.098 |
| EE\_16 | <--- | EOE\_1 | 29.387 | -.101 |
| EE\_16 | <--- | AISE\_6 | 33.174 | -.075 |
| EE\_16 | <--- | AISE\_5 | 21.908 | -.064 |
| EE\_16 | <--- | AISE\_4 | 54.810 | -.096 |
| EE\_16 | <--- | AISE\_3 | 24.160 | -.065 |
| EE\_16 | <--- | AISE\_2 | 25.162 | -.067 |
| EE\_16 | <--- | AISE\_1 | 30.127 | -.068 |
| EE\_16 | <--- | ESE\_10 | 6.597 | -.046 |
| EE\_16 | <--- | ESE\_9 | 14.840 | -.070 |
| EE\_16 | <--- | ESE\_8 | 23.350 | -.086 |
| EE\_16 | <--- | ESE\_7 | 12.407 | -.063 |
| EE\_16 | <--- | ESE\_6 | 15.414 | -.068 |
| EE\_16 | <--- | ESE\_5 | 11.696 | -.060 |
| EE\_16 | <--- | ESE\_4 | 14.718 | -.067 |
| EE\_16 | <--- | ESE\_3 | 12.868 | -.064 |
| EE\_16 | <--- | ESE\_2 | 16.135 | -.071 |
| EE\_16 | <--- | ESE\_1 | 18.171 | -.075 |
| EE\_16 | <--- | EE\_20 | 5.740 | .044 |
| EE\_16 | <--- | EE\_19 | 10.138 | .058 |
| EE\_16 | <--- | EE\_18 | 8.360 | .053 |
| EE\_16 | <--- | EE\_17 | 213.733 | .262 |
| EE\_16 | <--- | EE\_15 | 187.906 | .244 |
| EE\_16 | <--- | EE\_14 | 168.561 | .230 |
| EE\_16 | <--- | EE\_13 | 178.371 | .236 |
| EE\_16 | <--- | EE\_12 | 12.725 | .063 |
| EE\_16 | <--- | EE\_11 | 13.037 | .063 |
| EE\_16 | <--- | EE\_10 | 7.677 | .048 |
| EE\_16 | <--- | EE\_9 | 12.330 | .061 |
| EE\_16 | <--- | EE\_8 | 22.587 | .083 |
| EE\_16 | <--- | EE\_7 | 15.254 | .068 |
| EE\_16 | <--- | EE\_6 | 9.129 | .053 |
| EE\_16 | <--- | EE\_5 | 6.518 | .044 |
| EE\_16 | <--- | EE\_3 | 13.601 | .064 |
| EE\_16 | <--- | EE\_2 | 6.580 | .044 |
| EE\_16 | <--- | EE\_1 | 7.632 | .049 |
| EE\_15 | <--- | EOE\_9 | 35.071 | -.108 |
| EE\_15 | <--- | EOE\_8 | 28.764 | -.097 |
| EE\_15 | <--- | EOE\_7 | 23.797 | -.090 |
| EE\_15 | <--- | EOE\_6 | 25.174 | -.092 |
| EE\_15 | <--- | EOE\_5 | 31.865 | -.103 |
| EE\_15 | <--- | EOE\_4 | 34.605 | -.109 |
| EE\_15 | <--- | EOE\_3 | 19.524 | -.081 |
| EE\_15 | <--- | EOE\_2 | 18.863 | -.079 |
| EE\_15 | <--- | EOE\_1 | 31.045 | -.102 |
| EE\_15 | <--- | AISE\_6 | 26.441 | -.066 |
| EE\_15 | <--- | AISE\_5 | 15.761 | -.054 |
| EE\_15 | <--- | AISE\_4 | 27.281 | -.067 |
| EE\_15 | <--- | AISE\_3 | 14.203 | -.049 |
| EE\_15 | <--- | AISE\_2 | 19.342 | -.057 |
| EE\_15 | <--- | AISE\_1 | 15.543 | -.048 |
| EE\_15 | <--- | ESE\_10 | 19.219 | -.078 |
| EE\_15 | <--- | ESE\_9 | 19.101 | -.078 |
| EE\_15 | <--- | ESE\_8 | 14.055 | -.066 |
| EE\_15 | <--- | ESE\_7 | 20.549 | -.080 |
| EE\_15 | <--- | ESE\_6 | 40.062 | -.108 |
| EE\_15 | <--- | ESE\_5 | 30.929 | -.097 |
| EE\_15 | <--- | ESE\_4 | 28.616 | -.092 |
| EE\_15 | <--- | ESE\_3 | 37.223 | -.108 |
| EE\_15 | <--- | ESE\_2 | 30.334 | -.095 |
| EE\_15 | <--- | ESE\_1 | 39.737 | -.109 |
| EE\_15 | <--- | EE\_20 | 13.040 | .065 |
| EE\_15 | <--- | EE\_19 | 25.033 | .090 |
| EE\_15 | <--- | EE\_18 | 18.900 | .078 |
| EE\_15 | <--- | EE\_17 | 202.355 | .250 |
| EE\_15 | <--- | EE\_16 | 192.939 | .242 |
| EE\_15 | <--- | EE\_14 | 174.709 | .231 |
| EE\_15 | <--- | EE\_13 | 186.820 | .237 |
| EE\_15 | <--- | EE\_12 | 12.427 | .061 |
| EE\_15 | <--- | EE\_11 | 10.227 | .055 |
| EE\_15 | <--- | EE\_10 | 13.491 | .063 |
| EE\_15 | <--- | EE\_9 | 15.634 | .068 |
| EE\_15 | <--- | EE\_8 | 11.572 | .059 |
| EE\_15 | <--- | EE\_7 | 6.489 | .044 |
| EE\_15 | <--- | EE\_6 | 8.239 | .049 |
| EE\_15 | <--- | EE\_5 | 7.082 | .045 |
| EE\_15 | <--- | EE\_4 | 8.183 | .049 |
| EE\_15 | <--- | EE\_3 | 6.102 | .042 |
| EE\_15 | <--- | EE\_2 | 6.859 | .045 |
| EE\_15 | <--- | EE\_1 | 12.214 | .060 |
| EE\_14 | <--- | EOE\_9 | 28.635 | -.099 |
| EE\_14 | <--- | EOE\_8 | 35.215 | -.108 |
| EE\_14 | <--- | EOE\_7 | 29.238 | -.100 |
| EE\_14 | <--- | EOE\_6 | 32.490 | -.106 |
| EE\_14 | <--- | EOE\_5 | 23.501 | -.089 |
| EE\_14 | <--- | EOE\_4 | 33.265 | -.108 |
| EE\_14 | <--- | EOE\_3 | 27.725 | -.097 |
| EE\_14 | <--- | EOE\_2 | 35.872 | -.110 |
| EE\_14 | <--- | EOE\_1 | 31.263 | -.103 |
| EE\_14 | <--- | AISE\_6 | 8.684 | -.038 |
| EE\_14 | <--- | AISE\_5 | 7.745 | -.038 |
| EE\_14 | <--- | AISE\_4 | 13.142 | -.047 |
| EE\_14 | <--- | AISE\_3 | 13.377 | -.048 |
| EE\_14 | <--- | AISE\_2 | 10.266 | -.042 |
| EE\_14 | <--- | AISE\_1 | 13.466 | -.045 |
| EE\_14 | <--- | ESE\_10 | 8.175 | -.051 |
| EE\_14 | <--- | ESE\_9 | 18.631 | -.078 |
| EE\_14 | <--- | ESE\_8 | 16.007 | -.071 |
| EE\_14 | <--- | ESE\_7 | 20.385 | -.080 |
| EE\_14 | <--- | ESE\_6 | 15.025 | -.066 |
| EE\_14 | <--- | ESE\_5 | 31.176 | -.098 |
| EE\_14 | <--- | ESE\_4 | 24.887 | -.086 |
| EE\_14 | <--- | ESE\_3 | 13.995 | -.066 |
| EE\_14 | <--- | ESE\_2 | 15.409 | -.068 |
| EE\_14 | <--- | ESE\_1 | 20.332 | -.078 |
| EE\_14 | <--- | EE\_20 | 8.001 | .051 |
| EE\_14 | <--- | EE\_18 | 4.638 | .039 |
| EE\_14 | <--- | EE\_17 | 180.418 | .238 |
| EE\_14 | <--- | EE\_16 | 171.686 | .229 |
| EE\_14 | <--- | EE\_15 | 173.306 | .232 |
| EE\_14 | <--- | EE\_13 | 199.092 | .246 |
| EE\_14 | <--- | EE\_12 | 4.583 | .037 |
| EE\_14 | <--- | EE\_11 | 11.667 | .059 |
| EE\_14 | <--- | EE\_9 | 8.522 | .051 |
| EE\_14 | <--- | EE\_8 | 8.085 | .049 |
| EE\_14 | <--- | EE\_7 | 5.088 | .039 |
| EE\_14 | <--- | EE\_6 | 12.123 | .060 |
| EE\_14 | <--- | EE\_5 | 5.432 | .040 |
| EE\_14 | <--- | EE\_4 | 4.390 | .036 |
| EE\_14 | <--- | EE\_3 | 13.227 | .062 |
| EE\_14 | <--- | EE\_2 | 10.424 | .055 |
| EE\_14 | <--- | EE\_1 | 9.647 | .054 |
| EE\_13 | <--- | EOE\_9 | 27.331 | -.096 |
| EE\_13 | <--- | EOE\_8 | 34.428 | -.106 |
| EE\_13 | <--- | EOE\_7 | 29.686 | -.101 |
| EE\_13 | <--- | EOE\_6 | 24.528 | -.091 |
| EE\_13 | <--- | EOE\_5 | 42.006 | -.118 |
| EE\_13 | <--- | EOE\_4 | 35.264 | -.110 |
| EE\_13 | <--- | EOE\_3 | 32.004 | -.104 |
| EE\_13 | <--- | EOE\_2 | 34.413 | -.107 |
| EE\_13 | <--- | EOE\_1 | 24.600 | -.091 |
| EE\_13 | <--- | AISE\_6 | 23.723 | -.062 |
| EE\_13 | <--- | AISE\_5 | 16.344 | -.055 |
| EE\_13 | <--- | AISE\_4 | 31.326 | -.071 |
| EE\_13 | <--- | AISE\_3 | 21.691 | -.061 |
| EE\_13 | <--- | AISE\_2 | 17.731 | -.055 |
| EE\_13 | <--- | AISE\_1 | 18.745 | -.053 |
| EE\_13 | <--- | ESE\_10 | 15.763 | -.070 |
| EE\_13 | <--- | ESE\_9 | 13.957 | -.067 |
| EE\_13 | <--- | ESE\_8 | 18.957 | -.077 |
| EE\_13 | <--- | ESE\_7 | 15.618 | -.070 |
| EE\_13 | <--- | ESE\_6 | 15.405 | -.067 |
| EE\_13 | <--- | ESE\_5 | 10.168 | -.055 |
| EE\_13 | <--- | ESE\_4 | 10.160 | -.055 |
| EE\_13 | <--- | ESE\_3 | 8.744 | -.052 |
| EE\_13 | <--- | ESE\_2 | 23.460 | -.084 |
| EE\_13 | <--- | ESE\_1 | 21.819 | -.081 |
| EE\_13 | <--- | EE\_20 | 14.263 | .068 |
| EE\_13 | <--- | EE\_19 | 16.571 | .073 |
| EE\_13 | <--- | EE\_18 | 17.570 | .076 |
| EE\_13 | <--- | EE\_17 | 161.754 | .224 |
| EE\_13 | <--- | EE\_16 | 186.911 | .238 |
| EE\_13 | <--- | EE\_15 | 190.657 | .242 |
| EE\_13 | <--- | EE\_14 | 204.826 | .250 |
| EE\_13 | <--- | EE\_12 | 5.611 | .041 |
| EE\_13 | <--- | EE\_11 | 15.891 | .069 |
| EE\_13 | <--- | EE\_10 | 10.378 | .055 |
| EE\_13 | <--- | EE\_9 | 16.981 | .071 |
| EE\_13 | <--- | EE\_8 | 11.655 | .059 |
| EE\_13 | <--- | EE\_7 | 9.593 | .053 |
| EE\_13 | <--- | EE\_6 | 6.779 | .045 |
| EE\_13 | <--- | EE\_3 | 4.652 | .037 |
| EE\_13 | <--- | EE\_2 | 6.003 | .042 |
| EE\_13 | <--- | EE\_1 | 11.402 | .058 |
| EE\_12 | <--- | EOE\_9 | 31.576 | -.103 |
| EE\_12 | <--- | EOE\_8 | 40.898 | -.116 |
| EE\_12 | <--- | EOE\_7 | 20.361 | -.083 |
| EE\_12 | <--- | EOE\_6 | 29.759 | -.101 |
| EE\_12 | <--- | EOE\_5 | 24.508 | -.090 |
| EE\_12 | <--- | EOE\_4 | 35.378 | -.111 |
| EE\_12 | <--- | EOE\_3 | 20.827 | -.084 |
| EE\_12 | <--- | EOE\_2 | 29.105 | -.099 |
| EE\_12 | <--- | EOE\_1 | 20.152 | -.082 |
| EE\_12 | <--- | AISE\_6 | 29.998 | -.070 |
| EE\_12 | <--- | AISE\_5 | 20.623 | -.061 |
| EE\_12 | <--- | AISE\_4 | 13.888 | -.048 |
| EE\_12 | <--- | AISE\_3 | 24.031 | -.064 |
| EE\_12 | <--- | AISE\_2 | 10.498 | -.042 |
| EE\_12 | <--- | AISE\_1 | 19.220 | -.054 |
| EE\_12 | <--- | ESE\_10 | 19.721 | -.079 |
| EE\_12 | <--- | ESE\_9 | 18.530 | -.077 |
| EE\_12 | <--- | ESE\_8 | 5.302 | -.041 |
| EE\_12 | <--- | ESE\_7 | 20.761 | -.080 |
| EE\_12 | <--- | ESE\_6 | 13.629 | -.063 |
| EE\_12 | <--- | ESE\_5 | 23.305 | -.084 |
| EE\_12 | <--- | ESE\_4 | 29.151 | -.093 |
| EE\_12 | <--- | ESE\_3 | 25.891 | -.090 |
| EE\_12 | <--- | ESE\_2 | 18.997 | -.076 |
| EE\_12 | <--- | ESE\_1 | 32.400 | -.098 |
| EE\_12 | <--- | EE\_20 | 12.144 | .062 |
| EE\_12 | <--- | EE\_19 | 6.182 | .045 |
| EE\_12 | <--- | EE\_18 | 12.199 | .063 |
| EE\_12 | <--- | EE\_17 | 6.364 | .044 |
| EE\_12 | <--- | EE\_16 | 13.328 | .064 |
| EE\_12 | <--- | EE\_15 | 12.677 | .062 |
| EE\_12 | <--- | EE\_14 | 4.713 | .038 |
| EE\_12 | <--- | EE\_13 | 5.609 | .041 |
| EE\_12 | <--- | EE\_11 | 164.095 | .220 |
| EE\_12 | <--- | EE\_10 | 152.497 | .212 |
| EE\_12 | <--- | EE\_9 | 166.598 | .222 |
| EE\_12 | <--- | EE\_8 | 161.518 | .219 |
| EE\_12 | <--- | EE\_7 | 161.267 | .217 |
| EE\_12 | <--- | EE\_6 | 4.169 | .035 |
| EE\_12 | <--- | EE\_5 | 11.621 | .058 |
| EE\_12 | <--- | EE\_2 | 4.576 | .036 |
| EE\_12 | <--- | EE\_1 | 6.628 | .045 |
| EE\_11 | <--- | EOE\_9 | 47.051 | -.130 |
| EE\_11 | <--- | EOE\_8 | 46.176 | -.127 |
| EE\_11 | <--- | EOE\_7 | 27.437 | -.100 |
| EE\_11 | <--- | EOE\_6 | 26.713 | -.098 |
| EE\_11 | <--- | EOE\_5 | 52.065 | -.136 |
| EE\_11 | <--- | EOE\_4 | 32.892 | -.110 |
| EE\_11 | <--- | EOE\_3 | 37.435 | -.116 |
| EE\_11 | <--- | EOE\_2 | 36.255 | -.114 |
| EE\_11 | <--- | EOE\_1 | 28.934 | -.102 |
| EE\_11 | <--- | AISE\_6 | 16.585 | -.054 |
| EE\_11 | <--- | AISE\_5 | 24.306 | -.069 |
| EE\_11 | <--- | AISE\_4 | 24.871 | -.066 |
| EE\_11 | <--- | AISE\_3 | 21.371 | -.062 |
| EE\_11 | <--- | AISE\_2 | 19.680 | -.060 |
| EE\_11 | <--- | AISE\_1 | 20.136 | -.057 |
| EE\_11 | <--- | ESE\_10 | 13.169 | -.066 |
| EE\_11 | <--- | ESE\_9 | 14.191 | -.070 |
| EE\_11 | <--- | ESE\_8 | 11.385 | -.061 |
| EE\_11 | <--- | ESE\_7 | 19.740 | -.081 |
| EE\_11 | <--- | ESE\_6 | 35.973 | -.105 |
| EE\_11 | <--- | ESE\_5 | 32.151 | -.102 |
| EE\_11 | <--- | ESE\_4 | 22.055 | -.083 |
| EE\_11 | <--- | ESE\_3 | 32.711 | -.104 |
| EE\_11 | <--- | ESE\_2 | 34.256 | -.105 |
| EE\_11 | <--- | ESE\_1 | 32.789 | -.102 |
| EE\_11 | <--- | EE\_20 | 10.192 | .059 |
| EE\_11 | <--- | EE\_18 | 20.876 | .085 |
| EE\_11 | <--- | EE\_17 | 19.273 | .080 |
| EE\_11 | <--- | EE\_16 | 12.918 | .065 |
| EE\_11 | <--- | EE\_15 | 9.869 | .057 |
| EE\_11 | <--- | EE\_14 | 11.350 | .061 |
| EE\_11 | <--- | EE\_13 | 15.026 | .069 |
| EE\_11 | <--- | EE\_12 | 155.235 | .223 |
| EE\_11 | <--- | EE\_10 | 183.864 | .240 |
| EE\_11 | <--- | EE\_9 | 172.262 | .233 |
| EE\_11 | <--- | EE\_8 | 198.232 | .251 |
| EE\_11 | <--- | EE\_7 | 193.763 | .246 |
| EE\_11 | <--- | EE\_6 | 18.267 | .076 |
| EE\_11 | <--- | EE\_5 | 4.775 | .038 |
| EE\_11 | <--- | EE\_4 | 5.405 | .041 |
| EE\_11 | <--- | EE\_3 | 4.384 | .037 |
| EE\_11 | <--- | EE\_1 | 4.681 | .039 |
| EE\_10 | <--- | EOE\_9 | 34.137 | -.109 |
| EE\_10 | <--- | EOE\_8 | 42.387 | -.120 |
| EE\_10 | <--- | EOE\_7 | 29.526 | -.102 |
| EE\_10 | <--- | EOE\_6 | 29.303 | -.102 |
| EE\_10 | <--- | EOE\_5 | 45.772 | -.126 |
| EE\_10 | <--- | EOE\_4 | 24.278 | -.093 |
| EE\_10 | <--- | EOE\_3 | 29.444 | -.101 |
| EE\_10 | <--- | EOE\_2 | 36.446 | -.113 |
| EE\_10 | <--- | EOE\_1 | 31.240 | -.104 |
| EE\_10 | <--- | AISE\_6 | 14.575 | -.050 |
| EE\_10 | <--- | AISE\_5 | 18.379 | -.059 |
| EE\_10 | <--- | AISE\_4 | 22.833 | -.062 |
| EE\_10 | <--- | AISE\_3 | 20.340 | -.060 |
| EE\_10 | <--- | AISE\_2 | 19.027 | -.058 |
| EE\_10 | <--- | AISE\_1 | 13.961 | -.046 |
| EE\_10 | <--- | ESE\_10 | 24.855 | -.090 |
| EE\_10 | <--- | ESE\_9 | 13.768 | -.068 |
| EE\_10 | <--- | ESE\_8 | 10.157 | -.057 |
| EE\_10 | <--- | ESE\_7 | 18.553 | -.077 |
| EE\_10 | <--- | ESE\_6 | 20.004 | -.078 |
| EE\_10 | <--- | ESE\_5 | 20.088 | -.079 |
| EE\_10 | <--- | ESE\_4 | 16.938 | -.072 |
| EE\_10 | <--- | ESE\_3 | 18.852 | -.078 |
| EE\_10 | <--- | ESE\_2 | 17.948 | -.075 |
| EE\_10 | <--- | ESE\_1 | 17.477 | -.074 |
| EE\_10 | <--- | EE\_20 | 8.590 | .053 |
| EE\_10 | <--- | EE\_18 | 13.994 | .069 |
| EE\_10 | <--- | EE\_17 | 11.595 | .061 |
| EE\_10 | <--- | EE\_16 | 7.934 | .050 |
| EE\_10 | <--- | EE\_15 | 13.578 | .066 |
| EE\_10 | <--- | EE\_13 | 10.235 | .056 |
| EE\_10 | <--- | EE\_12 | 150.465 | .217 |
| EE\_10 | <--- | EE\_11 | 191.768 | .242 |
| EE\_10 | <--- | EE\_9 | 183.913 | .238 |
| EE\_10 | <--- | EE\_8 | 149.252 | .214 |
| EE\_10 | <--- | EE\_7 | 164.880 | .223 |
| EE\_10 | <--- | EE\_6 | 9.966 | .055 |
| EE\_10 | <--- | EE\_5 | 4.193 | .035 |
| EE\_10 | <--- | EE\_4 | 4.876 | .039 |
| EE\_10 | <--- | EE\_3 | 9.306 | .053 |
| EE\_9 | <--- | EOE\_9 | 29.610 | -.102 |
| EE\_9 | <--- | EOE\_8 | 26.727 | -.096 |
| EE\_9 | <--- | EOE\_7 | 34.227 | -.110 |
| EE\_9 | <--- | EOE\_6 | 24.738 | -.094 |
| EE\_9 | <--- | EOE\_5 | 46.333 | -.127 |
| EE\_9 | <--- | EOE\_4 | 22.087 | -.089 |
| EE\_9 | <--- | EOE\_3 | 43.375 | -.123 |
| EE\_9 | <--- | EOE\_2 | 33.229 | -.108 |
| EE\_9 | <--- | EOE\_1 | 25.188 | -.094 |
| EE\_9 | <--- | AISE\_6 | 32.268 | -.074 |
| EE\_9 | <--- | AISE\_5 | 29.808 | -.075 |
| EE\_9 | <--- | AISE\_4 | 31.770 | -.073 |
| EE\_9 | <--- | AISE\_3 | 33.349 | -.077 |
| EE\_9 | <--- | AISE\_2 | 29.881 | -.073 |
| EE\_9 | <--- | AISE\_1 | 19.744 | -.055 |
| EE\_9 | <--- | ESE\_10 | 18.565 | -.078 |
| EE\_9 | <--- | ESE\_9 | 21.143 | -.084 |
| EE\_9 | <--- | ESE\_8 | 18.883 | -.078 |
| EE\_9 | <--- | ESE\_7 | 17.524 | -.075 |
| EE\_9 | <--- | ESE\_6 | 34.295 | -.102 |
| EE\_9 | <--- | ESE\_5 | 25.874 | -.090 |
| EE\_9 | <--- | ESE\_4 | 17.265 | -.073 |
| EE\_9 | <--- | ESE\_3 | 25.167 | -.090 |
| EE\_9 | <--- | ESE\_2 | 21.035 | -.081 |
| EE\_9 | <--- | ESE\_1 | 21.346 | -.081 |
| EE\_9 | <--- | EE\_20 | 11.857 | .063 |
| EE\_9 | <--- | EE\_19 | 9.310 | .056 |
| EE\_9 | <--- | EE\_18 | 19.282 | .081 |
| EE\_9 | <--- | EE\_17 | 10.088 | .057 |
| EE\_9 | <--- | EE\_16 | 12.550 | .063 |
| EE\_9 | <--- | EE\_15 | 15.498 | .070 |
| EE\_9 | <--- | EE\_14 | 8.516 | .052 |
| EE\_9 | <--- | EE\_13 | 16.494 | .072 |
| EE\_9 | <--- | EE\_12 | 161.899 | .225 |
| EE\_9 | <--- | EE\_11 | 176.958 | .233 |
| EE\_9 | <--- | EE\_10 | 181.139 | .235 |
| EE\_9 | <--- | EE\_8 | 177.696 | .235 |
| EE\_9 | <--- | EE\_7 | 168.730 | .226 |
| EE\_9 | <--- | EE\_6 | 6.980 | .046 |
| EE\_9 | <--- | EE\_5 | 11.364 | .058 |
| EE\_9 | <--- | EE\_4 | 5.311 | .040 |
| EE\_9 | <--- | EE\_2 | 7.426 | .047 |
| EE\_9 | <--- | EE\_1 | 11.058 | .059 |
| EE\_8 | <--- | EOE\_9 | 20.465 | -.085 |
| EE\_8 | <--- | EOE\_8 | 39.017 | -.117 |
| EE\_8 | <--- | EOE\_7 | 22.344 | -.090 |
| EE\_8 | <--- | EOE\_6 | 17.716 | -.080 |
| EE\_8 | <--- | EOE\_5 | 32.313 | -.107 |
| EE\_8 | <--- | EOE\_4 | 40.269 | -.122 |
| EE\_8 | <--- | EOE\_3 | 36.817 | -.115 |
| EE\_8 | <--- | EOE\_2 | 29.837 | -.103 |
| EE\_8 | <--- | EOE\_1 | 23.770 | -.092 |
| EE\_8 | <--- | AISE\_6 | 22.107 | -.062 |
| EE\_8 | <--- | AISE\_5 | 12.434 | -.049 |
| EE\_8 | <--- | AISE\_4 | 26.255 | -.068 |
| EE\_8 | <--- | AISE\_3 | 24.525 | -.066 |
| EE\_8 | <--- | AISE\_2 | 17.061 | -.056 |
| EE\_8 | <--- | AISE\_1 | 22.475 | -.060 |
| EE\_8 | <--- | ESE\_10 | 7.415 | -.050 |
| EE\_8 | <--- | ESE\_9 | 6.975 | -.049 |
| EE\_8 | <--- | ESE\_8 | 8.428 | -.053 |
| EE\_8 | <--- | ESE\_7 | 13.884 | -.068 |
| EE\_8 | <--- | ESE\_6 | 23.753 | -.086 |
| EE\_8 | <--- | ESE\_5 | 17.549 | -.075 |
| EE\_8 | <--- | ESE\_4 | 9.380 | -.054 |
| EE\_8 | <--- | ESE\_3 | 25.388 | -.092 |
| EE\_8 | <--- | ESE\_2 | 15.981 | -.072 |
| EE\_8 | <--- | ESE\_1 | 29.519 | -.097 |
| EE\_8 | <--- | EE\_20 | 5.342 | .043 |
| EE\_8 | <--- | EE\_18 | 5.782 | .045 |
| EE\_8 | <--- | EE\_17 | 12.631 | .065 |
| EE\_8 | <--- | EE\_16 | 22.273 | .085 |
| EE\_8 | <--- | EE\_15 | 11.113 | .060 |
| EE\_8 | <--- | EE\_14 | 7.827 | .050 |
| EE\_8 | <--- | EE\_13 | 10.968 | .059 |
| EE\_8 | <--- | EE\_12 | 152.066 | .221 |
| EE\_8 | <--- | EE\_11 | 197.284 | .249 |
| EE\_8 | <--- | EE\_10 | 142.416 | .211 |
| EE\_8 | <--- | EE\_9 | 172.153 | .233 |
| EE\_8 | <--- | EE\_7 | 152.216 | .218 |
| EE\_8 | <--- | EE\_6 | 8.978 | .053 |
| EE\_7 | <--- | EOE\_9 | 25.952 | -.096 |
| EE\_7 | <--- | EOE\_8 | 37.370 | -.114 |
| EE\_7 | <--- | EOE\_7 | 36.400 | -.115 |
| EE\_7 | <--- | EOE\_6 | 25.801 | -.096 |
| EE\_7 | <--- | EOE\_5 | 34.440 | -.110 |
| EE\_7 | <--- | EOE\_4 | 20.591 | -.087 |
| EE\_7 | <--- | EOE\_3 | 27.799 | -.100 |
| EE\_7 | <--- | EOE\_2 | 15.888 | -.075 |
| EE\_7 | <--- | EOE\_1 | 21.789 | -.088 |
| EE\_7 | <--- | AISE\_6 | 17.838 | -.055 |
| EE\_7 | <--- | AISE\_5 | 21.650 | -.065 |
| EE\_7 | <--- | AISE\_4 | 16.395 | -.053 |
| EE\_7 | <--- | AISE\_3 | 27.803 | -.071 |
| EE\_7 | <--- | AISE\_2 | 19.860 | -.060 |
| EE\_7 | <--- | AISE\_1 | 17.263 | -.052 |
| EE\_7 | <--- | ESE\_10 | 9.539 | -.056 |
| EE\_7 | <--- | ESE\_9 | 16.489 | -.075 |
| EE\_7 | <--- | ESE\_8 | 13.316 | -.066 |
| EE\_7 | <--- | ESE\_7 | 17.768 | -.076 |
| EE\_7 | <--- | ESE\_6 | 23.544 | -.085 |
| EE\_7 | <--- | ESE\_5 | 23.760 | -.087 |
| EE\_7 | <--- | ESE\_4 | 15.991 | -.071 |
| EE\_7 | <--- | ESE\_3 | 22.626 | -.086 |
| EE\_7 | <--- | ESE\_2 | 11.878 | -.061 |
| EE\_7 | <--- | ESE\_1 | 14.287 | -.067 |
| EE\_7 | <--- | EE\_20 | 4.194 | .038 |
| EE\_7 | <--- | EE\_19 | 10.453 | .060 |
| EE\_7 | <--- | EE\_18 | 12.277 | .065 |
| EE\_7 | <--- | EE\_17 | 9.634 | .056 |
| EE\_7 | <--- | EE\_16 | 15.460 | .070 |
| EE\_7 | <--- | EE\_15 | 6.405 | .046 |
| EE\_7 | <--- | EE\_14 | 5.063 | .040 |
| EE\_7 | <--- | EE\_13 | 9.279 | .054 |
| EE\_7 | <--- | EE\_12 | 156.055 | .223 |
| EE\_7 | <--- | EE\_11 | 198.201 | .249 |
| EE\_7 | <--- | EE\_10 | 161.706 | .224 |
| EE\_7 | <--- | EE\_9 | 168.016 | .230 |
| EE\_7 | <--- | EE\_8 | 156.452 | .222 |
| EE\_7 | <--- | EE\_5 | 5.827 | .042 |
| EE\_6 | <--- | EOE\_9 | 39.632 | -.117 |
| EE\_6 | <--- | EOE\_8 | 40.667 | -.117 |
| EE\_6 | <--- | EOE\_7 | 45.118 | -.126 |
| EE\_6 | <--- | EOE\_6 | 33.598 | -.109 |
| EE\_6 | <--- | EOE\_5 | 37.744 | -.114 |
| EE\_6 | <--- | EOE\_4 | 32.442 | -.108 |
| EE\_6 | <--- | EOE\_3 | 32.607 | -.106 |
| EE\_6 | <--- | EOE\_2 | 45.462 | -.125 |
| EE\_6 | <--- | EOE\_1 | 34.902 | -.110 |
| EE\_6 | <--- | AISE\_6 | 12.702 | -.046 |
| EE\_6 | <--- | AISE\_5 | 20.193 | -.062 |
| EE\_6 | <--- | AISE\_4 | 13.188 | -.047 |
| EE\_6 | <--- | AISE\_3 | 26.436 | -.068 |
| EE\_6 | <--- | AISE\_2 | 13.457 | -.049 |
| EE\_6 | <--- | AISE\_1 | 14.479 | -.047 |
| EE\_6 | <--- | ESE\_10 | 12.545 | -.064 |
| EE\_6 | <--- | ESE\_9 | 18.108 | -.077 |
| EE\_6 | <--- | ESE\_8 | 16.583 | -.073 |
| EE\_6 | <--- | ESE\_7 | 20.896 | -.082 |
| EE\_6 | <--- | ESE\_6 | 21.484 | -.080 |
| EE\_6 | <--- | ESE\_5 | 25.207 | -.089 |
| EE\_6 | <--- | ESE\_4 | 24.817 | -.087 |
| EE\_6 | <--- | ESE\_3 | 23.269 | -.086 |
| EE\_6 | <--- | ESE\_2 | 18.313 | -.075 |
| EE\_6 | <--- | ESE\_1 | 20.716 | -.080 |
| EE\_6 | <--- | EE\_20 | 8.699 | .054 |
| EE\_6 | <--- | EE\_19 | 8.295 | .053 |
| EE\_6 | <--- | EE\_18 | 10.739 | .060 |
| EE\_6 | <--- | EE\_17 | 12.137 | .062 |
| EE\_6 | <--- | EE\_16 | 9.443 | .054 |
| EE\_6 | <--- | EE\_15 | 8.300 | .051 |
| EE\_6 | <--- | EE\_14 | 12.312 | .062 |
| EE\_6 | <--- | EE\_13 | 6.692 | .046 |
| EE\_6 | <--- | EE\_12 | 4.118 | .036 |
| EE\_6 | <--- | EE\_11 | 19.070 | .076 |
| EE\_6 | <--- | EE\_10 | 9.976 | .055 |
| EE\_6 | <--- | EE\_9 | 7.093 | .047 |
| EE\_6 | <--- | EE\_8 | 9.418 | .054 |
| EE\_6 | <--- | EE\_5 | 155.935 | .216 |
| EE\_6 | <--- | EE\_4 | 196.811 | .245 |
| EE\_6 | <--- | EE\_3 | 160.532 | .220 |
| EE\_6 | <--- | EE\_2 | 176.975 | .230 |
| EE\_6 | <--- | EE\_1 | 163.476 | .225 |
| EE\_5 | <--- | EOE\_9 | 28.969 | -.103 |
| EE\_5 | <--- | EOE\_8 | 31.791 | -.107 |
| EE\_5 | <--- | EOE\_7 | 32.269 | -.110 |
| EE\_5 | <--- | EOE\_6 | 34.667 | -.114 |
| EE\_5 | <--- | EOE\_5 | 31.583 | -.107 |
| EE\_5 | <--- | EOE\_4 | 17.998 | -.083 |
| EE\_5 | <--- | EOE\_3 | 22.503 | -.091 |
| EE\_5 | <--- | EOE\_2 | 30.285 | -.105 |
| EE\_5 | <--- | EOE\_1 | 26.523 | -.099 |
| EE\_5 | <--- | AISE\_6 | 23.011 | -.064 |
| EE\_5 | <--- | AISE\_5 | 21.676 | -.066 |
| EE\_5 | <--- | AISE\_4 | 13.693 | -.049 |
| EE\_5 | <--- | AISE\_3 | 13.808 | -.051 |
| EE\_5 | <--- | AISE\_2 | 26.277 | -.070 |
| EE\_5 | <--- | AISE\_1 | 23.030 | -.061 |
| EE\_5 | <--- | ESE\_10 | 17.283 | -.077 |
| EE\_5 | <--- | ESE\_9 | 8.811 | -.056 |
| EE\_5 | <--- | ESE\_8 | 14.641 | -.070 |
| EE\_5 | <--- | ESE\_7 | 18.219 | -.079 |
| EE\_5 | <--- | ESE\_6 | 28.198 | -.095 |
| EE\_5 | <--- | ESE\_5 | 35.509 | -.108 |
| EE\_5 | <--- | ESE\_4 | 30.535 | -.099 |
| EE\_5 | <--- | ESE\_3 | 33.846 | -.107 |
| EE\_5 | <--- | ESE\_2 | 32.981 | -.104 |
| EE\_5 | <--- | ESE\_1 | 41.887 | -.117 |
| EE\_5 | <--- | EE\_20 | 18.499 | .081 |
| EE\_5 | <--- | EE\_19 | 20.781 | .086 |
| EE\_5 | <--- | EE\_18 | 23.985 | .093 |
| EE\_5 | <--- | EE\_16 | 6.429 | .046 |
| EE\_5 | <--- | EE\_15 | 6.802 | .048 |
| EE\_5 | <--- | EE\_14 | 5.260 | .042 |
| EE\_5 | <--- | EE\_12 | 10.943 | .060 |
| EE\_5 | <--- | EE\_11 | 4.753 | .039 |
| EE\_5 | <--- | EE\_10 | 4.001 | .036 |
| EE\_5 | <--- | EE\_9 | 11.012 | .060 |
| EE\_5 | <--- | EE\_7 | 5.670 | .043 |
| EE\_5 | <--- | EE\_6 | 148.679 | .219 |
| EE\_5 | <--- | EE\_4 | 199.350 | .255 |
| EE\_5 | <--- | EE\_3 | 190.261 | .246 |
| EE\_5 | <--- | EE\_2 | 168.831 | .231 |
| EE\_5 | <--- | EE\_1 | 154.846 | .225 |
| EE\_4 | <--- | EOE\_9 | 25.872 | -.096 |
| EE\_4 | <--- | EOE\_8 | 39.864 | -.119 |
| EE\_4 | <--- | EOE\_7 | 15.623 | -.076 |
| EE\_4 | <--- | EOE\_6 | 38.786 | -.119 |
| EE\_4 | <--- | EOE\_5 | 36.882 | -.115 |
| EE\_4 | <--- | EOE\_4 | 17.208 | -.080 |
| EE\_4 | <--- | EOE\_3 | 32.066 | -.108 |
| EE\_4 | <--- | EOE\_2 | 45.624 | -.128 |
| EE\_4 | <--- | EOE\_1 | 40.096 | -.121 |
| EE\_4 | <--- | AISE\_6 | 6.269 | -.033 |
| EE\_4 | <--- | AISE\_5 | 15.681 | -.055 |
| EE\_4 | <--- | AISE\_4 | 6.309 | -.033 |
| EE\_4 | <--- | AISE\_3 | 12.161 | -.047 |
| EE\_4 | <--- | AISE\_2 | 13.498 | -.050 |
| EE\_4 | <--- | AISE\_1 | 5.799 | -.030 |
| EE\_4 | <--- | ESE\_10 | 20.273 | -.083 |
| EE\_4 | <--- | ESE\_9 | 25.611 | -.094 |
| EE\_4 | <--- | ESE\_8 | 18.825 | -.079 |
| EE\_4 | <--- | ESE\_7 | 23.981 | -.089 |
| EE\_4 | <--- | ESE\_6 | 17.308 | -.073 |
| EE\_4 | <--- | ESE\_5 | 26.806 | -.093 |
| EE\_4 | <--- | ESE\_4 | 30.608 | -.098 |
| EE\_4 | <--- | ESE\_3 | 24.968 | -.091 |
| EE\_4 | <--- | ESE\_2 | 21.035 | -.082 |
| EE\_4 | <--- | ESE\_1 | 34.522 | -.105 |
| EE\_4 | <--- | EE\_20 | 11.716 | .063 |
| EE\_4 | <--- | EE\_19 | 14.235 | .070 |
| EE\_4 | <--- | EE\_18 | 15.445 | .074 |
| EE\_4 | <--- | EE\_15 | 7.809 | .051 |
| EE\_4 | <--- | EE\_14 | 4.224 | .037 |
| EE\_4 | <--- | EE\_11 | 5.346 | .041 |
| EE\_4 | <--- | EE\_10 | 4.624 | .038 |
| EE\_4 | <--- | EE\_9 | 5.113 | .040 |
| EE\_4 | <--- | EE\_6 | 186.447 | .243 |
| EE\_4 | <--- | EE\_5 | 198.070 | .248 |
| EE\_4 | <--- | EE\_3 | 180.777 | .238 |
| EE\_4 | <--- | EE\_2 | 191.049 | .244 |
| EE\_4 | <--- | EE\_1 | 170.772 | .234 |
| EE\_3 | <--- | EOE\_9 | 31.447 | -.105 |
| EE\_3 | <--- | EOE\_8 | 37.751 | -.114 |
| EE\_3 | <--- | EOE\_7 | 34.199 | -.110 |
| EE\_3 | <--- | EOE\_6 | 25.547 | -.095 |
| EE\_3 | <--- | EOE\_5 | 38.228 | -.115 |
| EE\_3 | <--- | EOE\_4 | 25.970 | -.097 |
| EE\_3 | <--- | EOE\_3 | 24.828 | -.093 |
| EE\_3 | <--- | EOE\_2 | 55.522 | -.139 |
| EE\_3 | <--- | EOE\_1 | 28.700 | -.100 |
| EE\_3 | <--- | AISE\_6 | 18.697 | -.056 |
| EE\_3 | <--- | AISE\_5 | 24.531 | -.068 |
| EE\_3 | <--- | AISE\_4 | 19.321 | -.057 |
| EE\_3 | <--- | AISE\_3 | 16.089 | -.053 |
| EE\_3 | <--- | AISE\_2 | 18.894 | -.058 |
| EE\_3 | <--- | AISE\_1 | 15.235 | -.049 |
| EE\_3 | <--- | ESE\_10 | 8.876 | -.054 |
| EE\_3 | <--- | ESE\_9 | 12.511 | -.065 |
| EE\_3 | <--- | ESE\_7 | 6.340 | -.045 |
| EE\_3 | <--- | ESE\_6 | 28.463 | -.093 |
| EE\_3 | <--- | ESE\_5 | 22.124 | -.083 |
| EE\_3 | <--- | ESE\_4 | 37.328 | -.107 |
| EE\_3 | <--- | ESE\_3 | 19.048 | -.079 |
| EE\_3 | <--- | ESE\_2 | 22.838 | -.085 |
| EE\_3 | <--- | ESE\_1 | 38.370 | -.109 |
| EE\_3 | <--- | EE\_20 | 17.274 | .076 |
| EE\_3 | <--- | EE\_19 | 6.209 | .046 |
| EE\_3 | <--- | EE\_18 | 16.010 | .074 |
| EE\_3 | <--- | EE\_17 | 11.790 | .062 |
| EE\_3 | <--- | EE\_16 | 14.115 | .067 |
| EE\_3 | <--- | EE\_15 | 6.168 | .044 |
| EE\_3 | <--- | EE\_14 | 13.477 | .065 |
| EE\_3 | <--- | EE\_13 | 4.607 | .038 |
| EE\_3 | <--- | EE\_11 | 4.592 | .038 |
| EE\_3 | <--- | EE\_10 | 9.345 | .054 |
| EE\_3 | <--- | EE\_6 | 161.062 | .223 |
| EE\_3 | <--- | EE\_5 | 200.207 | .246 |
| EE\_3 | <--- | EE\_4 | 191.456 | .243 |
| EE\_3 | <--- | EE\_2 | 143.391 | .208 |
| EE\_3 | <--- | EE\_1 | 178.667 | .236 |
| EE\_2 | <--- | EOE\_9 | 14.583 | -.072 |
| EE\_2 | <--- | EOE\_8 | 24.334 | -.093 |
| EE\_2 | <--- | EOE\_7 | 14.172 | -.072 |
| EE\_2 | <--- | EOE\_6 | 34.474 | -.112 |
| EE\_2 | <--- | EOE\_5 | 15.168 | -.074 |
| EE\_2 | <--- | EOE\_4 | 19.434 | -.085 |
| EE\_2 | <--- | EOE\_3 | 25.003 | -.095 |
| EE\_2 | <--- | EOE\_2 | 17.301 | -.079 |
| EE\_2 | <--- | EOE\_1 | 20.789 | -.087 |
| EE\_2 | <--- | AISE\_6 | 20.127 | -.059 |
| EE\_2 | <--- | AISE\_5 | 18.542 | -.060 |
| EE\_2 | <--- | AISE\_4 | 19.127 | -.058 |
| EE\_2 | <--- | AISE\_3 | 42.658 | -.088 |
| EE\_2 | <--- | AISE\_2 | 24.331 | -.067 |
| EE\_2 | <--- | AISE\_1 | 23.130 | -.061 |
| EE\_2 | <--- | ESE\_10 | 15.425 | -.072 |
| EE\_2 | <--- | ESE\_9 | 10.319 | -.060 |
| EE\_2 | <--- | ESE\_8 | 10.281 | -.058 |
| EE\_2 | <--- | ESE\_7 | 18.989 | -.079 |
| EE\_2 | <--- | ESE\_6 | 14.043 | -.066 |
| EE\_2 | <--- | ESE\_5 | 17.291 | -.075 |
| EE\_2 | <--- | ESE\_4 | 24.557 | -.088 |
| EE\_2 | <--- | ESE\_3 | 23.694 | -.089 |
| EE\_2 | <--- | ESE\_2 | 19.406 | -.079 |
| EE\_2 | <--- | ESE\_1 | 39.828 | -.113 |
| EE\_2 | <--- | EE\_20 | 6.707 | .048 |
| EE\_2 | <--- | EE\_19 | 7.064 | .050 |
| EE\_2 | <--- | EE\_18 | 14.331 | .071 |
| EE\_2 | <--- | EE\_17 | 4.109 | .037 |
| EE\_2 | <--- | EE\_16 | 6.641 | .046 |
| EE\_2 | <--- | EE\_15 | 6.741 | .047 |
| EE\_2 | <--- | EE\_14 | 10.328 | .058 |
| EE\_2 | <--- | EE\_13 | 5.781 | .043 |
| EE\_2 | <--- | EE\_12 | 4.409 | .038 |
| EE\_2 | <--- | EE\_9 | 7.363 | .048 |
| EE\_2 | <--- | EE\_6 | 172.659 | .234 |
| EE\_2 | <--- | EE\_5 | 172.753 | .232 |
| EE\_2 | <--- | EE\_4 | 196.751 | .250 |
| EE\_2 | <--- | EE\_3 | 139.433 | .209 |
| EE\_2 | <--- | EE\_1 | 133.776 | .207 |
| EE\_1 | <--- | EOE\_9 | 41.512 | -.120 |
| EE\_1 | <--- | EOE\_8 | 25.426 | -.093 |
| EE\_1 | <--- | EOE\_7 | 28.458 | -.101 |
| EE\_1 | <--- | EOE\_6 | 30.429 | -.104 |
| EE\_1 | <--- | EOE\_5 | 45.898 | -.126 |
| EE\_1 | <--- | EOE\_4 | 30.943 | -.106 |
| EE\_1 | <--- | EOE\_3 | 34.307 | -.110 |
| EE\_1 | <--- | EOE\_2 | 33.215 | -.108 |
| EE\_1 | <--- | EOE\_1 | 32.975 | -.108 |
| EE\_1 | <--- | AISE\_6 | 16.382 | -.053 |
| EE\_1 | <--- | AISE\_5 | 17.026 | -.057 |
| EE\_1 | <--- | AISE\_4 | 13.451 | -.048 |
| EE\_1 | <--- | AISE\_3 | 18.171 | -.057 |
| EE\_1 | <--- | AISE\_2 | 22.253 | -.063 |
| EE\_1 | <--- | AISE\_1 | 15.148 | -.048 |
| EE\_1 | <--- | ESE\_10 | 18.131 | -.077 |
| EE\_1 | <--- | ESE\_9 | 15.354 | -.072 |
| EE\_1 | <--- | ESE\_8 | 8.790 | -.053 |
| EE\_1 | <--- | ESE\_7 | 10.458 | -.058 |
| EE\_1 | <--- | ESE\_6 | 19.412 | -.077 |
| EE\_1 | <--- | ESE\_5 | 17.181 | -.073 |
| EE\_1 | <--- | ESE\_4 | 17.255 | -.073 |
| EE\_1 | <--- | ESE\_3 | 14.393 | -.068 |
| EE\_1 | <--- | ESE\_2 | 17.505 | -.074 |
| EE\_1 | <--- | ESE\_1 | 11.035 | -.059 |
| EE\_1 | <--- | EE\_20 | 13.711 | .068 |
| EE\_1 | <--- | EE\_19 | 9.456 | .056 |
| EE\_1 | <--- | EE\_18 | 4.529 | .039 |
| EE\_1 | <--- | EE\_17 | 6.161 | .045 |
| EE\_1 | <--- | EE\_16 | 7.673 | .049 |
| EE\_1 | <--- | EE\_15 | 11.960 | .062 |
| EE\_1 | <--- | EE\_14 | 9.522 | .055 |
| EE\_1 | <--- | EE\_13 | 10.940 | .059 |
| EE\_1 | <--- | EE\_12 | 6.362 | .045 |
| EE\_1 | <--- | EE\_11 | 4.750 | .038 |
| EE\_1 | <--- | EE\_9 | 10.923 | .058 |
| EE\_1 | <--- | EE\_6 | 158.893 | .221 |
| EE\_1 | <--- | EE\_5 | 157.851 | .218 |
| EE\_1 | <--- | EE\_4 | 175.211 | .233 |
| EE\_1 | <--- | EE\_3 | 173.087 | .229 |
| EE\_1 | <--- | EE\_2 | 133.275 | .200 |

## Minimization History (Default model)

| Iteration |  | Negative eigenvalues | Condition # | Smallest eigenvalue | Diameter | F | NTries | Ratio |
| --- | --- | --- | --- | --- | --- | --- | --- | --- |
| 0 | e | 2 |  | -1.119 | 9999.000 | 43454.765 | 0 | 9999.000 |
| 1 | e | 2 |  | -.038 | 3.419 | 25786.156 | 18 | .250 |
| 2 | e\* | 0 | 22.773 |  | 2.079 | 23841.856 | 8 | .706 |
| 3 | e | 0 | 46.429 |  | 1.033 | 23492.718 | 1 | .869 |
| 4 | e | 0 | 81.370 |  | .676 | 23242.601 | 1 | 1.169 |
| 5 | e | 0 | 200.845 |  | .603 | 23201.924 | 1 | 1.215 |
| 6 | e | 0 | 379.516 |  | .397 | 23194.197 | 1 | 1.144 |
| 7 | e | 0 | 465.091 |  | .128 | 23193.612 | 1 | 1.062 |
| 8 | e | 0 | 472.306 |  | .015 | 23193.606 | 1 | 1.007 |
| 9 | e | 0 | 468.096 |  | .000 | 23193.606 | 1 | 1.000 |

## Model Fit Summary

## CMIN

| Model | NPAR | CMIN | DF | P | CMIN/DF |
| --- | --- | --- | --- | --- | --- |
| Default model | 90 | 23193.606 | 945 | .000 | 24.543 |
| Saturated model | 1035 | .000 | 0 |  |  |
| Independence model | 45 | 49963.739 | 990 | .000 | 50.468 |

## RMR, GFI

| Model | RMR | GFI | AGFI | PGFI |
| --- | --- | --- | --- | --- |
| Default model | .070 | .497 | .449 | .454 |
| Saturated model | .000 | 1.000 |  |  |
| Independence model | .190 | .204 | .168 | .195 |

## Baseline Comparisons

| Model | NFI Delta1 | RFI rho1 | IFI Delta2 | TLI rho2 | CFI |
| --- | --- | --- | --- | --- | --- |
| Default model | .536 | .514 | .546 | .524 | .546 |
| Saturated model | 1.000 |  | 1.000 |  | 1.000 |
| Independence model | .000 | .000 | .000 | .000 | .000 |

## Parsimony-Adjusted Measures

| Model | PRATIO | PNFI | PCFI |
| --- | --- | --- | --- |
| Default model | .955 | .511 | .521 |
| Saturated model | .000 | .000 | .000 |
| Independence model | 1.000 | .000 | .000 |

## NCP

| Model | NCP | LO 90 | HI 90 |
| --- | --- | --- | --- |
| Default model | 22248.606 | 21755.921 | 22747.659 |
| Saturated model | .000 | .000 | .000 |
| Independence model | 48973.739 | 48245.220 | 49708.577 |

## FMIN

| Model | FMIN | F0 | LO 90 | HI 90 |
| --- | --- | --- | --- | --- |
| Default model | 10.182 | 9.767 | 9.550 | 9.986 |
| Saturated model | .000 | .000 | .000 | .000 |
| Independence model | 21.933 | 21.499 | 21.179 | 21.821 |

## RMSEA

| Model | RMSEA | LO 90 | HI 90 | PCLOSE |
| --- | --- | --- | --- | --- |
| Default model | .102 | .101 | .103 | .000 |
| Independence model | .147 | .146 | .148 | .000 |

## AIC

| Model | AIC | BCC | BIC | CAIC |
| --- | --- | --- | --- | --- |
| Default model | 23373.606 | 23377.316 | 23889.441 | 23979.441 |
| Saturated model | 2070.000 | 2112.661 | 8002.094 | 9037.094 |
| Independence model | 50053.739 | 50055.594 | 50311.656 | 50356.656 |

## ECVI

| Model | ECVI | LO 90 | HI 90 | MECVI |
| --- | --- | --- | --- | --- |
| Default model | 10.261 | 10.044 | 10.480 | 10.262 |
| Saturated model | .909 | .909 | .909 | .927 |
| Independence model | 21.973 | 21.653 | 22.295 | 21.973 |

## HOELTER

| Model | HOELTER .05 | HOELTER .01 |
| --- | --- | --- |
| Default model | 100 | 104 |
| Independence model | 49 | 50 |

## Execution time summary

|  |  |
| --- | --- |
| Minimization: | .071 |
| Miscellaneous: | .731 |
| Bootstrap: | .000 |
| Total: | .802 |
