# Supplementary material for: AI-contextualized self-efficacy and exercise engagement among vocational college students in China: a cross-sectional serial mediation analysis of exercise self-efficacy and exercise outcome expectations
Source: Front Psychol. 2026 Oct 6;17:1933716. doi: 10.3389/fpsyg.2026.1933716 (PMC13680253; doi:10.3389/fpsyg.2026.1933716)
Supplement: Supplementary file 2 [file Data_Sheet_1.zip › Revision_Package_1933716/Single_Factor_Model_2/Single_Factor_Model_2.AmosOutput]

单因子模型2.amw


# D:\系统默认\桌面\danzi\2025年9月份单子\361.数据分析-220元\单因子模型2.amw

## Analysis Summary

## Date and Time

Date: 2026年9月13日

Time: 18:12:56

## Title

单因子模型2: 2026年9月13日 18:12

## Groups

## Group number 1 (Group number 1)

## Notes for Group (Group number 1)

The model is recursive.

Sample size = 2279

## Variable Summary (Group number 1)

## Your model contains the following variables (Group number 1)

Observed, endogenous variables

EOE\_1

EOE\_2

EOE\_3

EOE\_4

EOE\_5

EOE\_6

EOE\_7

EOE\_8

EOE\_9

EE\_13

EE\_14

EE\_15

EE\_16

EE\_17

Unobserved, exogenous variables

GEN

e1

e2

e3

e4

e5

e6

e7

e8

e9

e10

e11

e12

e13

e14

## Variable counts (Group number 1)

|  |  |
| --- | --- |
| Number of variables in your model: | 29 |
| Number of observed variables: | 14 |
| Number of unobserved variables: | 15 |
| Number of exogenous variables: | 15 |
| Number of endogenous variables: | 14 |

## Parameter Summary (Group number 1)

|  | Weights | Covariances | Variances | Means | Intercepts | Total |
| --- | --- | --- | --- | --- | --- | --- |
| Fixed | 15 | 0 | 0 | 0 | 0 | 15 |
| Labeled | 0 | 0 | 0 | 0 | 0 | 0 |
| Unlabeled | 13 | 0 | 15 | 0 | 0 | 28 |
| Total | 28 | 0 | 15 | 0 | 0 | 43 |

## Models

## Default model (Default model)

## Notes for Model (Default model)

## Computation of degrees of freedom (Default model)

|  |  |
| --- | --- |
| Number of distinct sample moments: | 105 |
| Number of distinct parameters to be estimated: | 28 |
| Degrees of freedom (105 - 28): | 77 |

## Result (Default model)

Minimum was achieved

Chi-square = 3889.447

Degrees of freedom = 77

Probability level = .000

## Group number 1 (Group number 1 - Default model)

## Estimates (Group number 1 - Default model)

## Scalar Estimates (Group number 1 - Default model)

## Maximum Likelihood Estimates

## Regression Weights: (Group number 1 - Default model)

|  |  |  | Estimate | S.E. | C.R. | P | Label |
| --- | --- | --- | --- | --- | --- | --- | --- |
| EOE\_1 | <--- | GEN | 1.000 |  |  |  |  |
| EOE\_2 | <--- | GEN | 1.014 | .031 | 32.886 | \*\*\* |  |
| EOE\_3 | <--- | GEN | 1.004 | .031 | 32.633 | \*\*\* |  |
| EOE\_4 | <--- | GEN | .989 | .030 | 32.557 | \*\*\* |  |
| EOE\_5 | <--- | GEN | 1.030 | .031 | 33.284 | \*\*\* |  |
| EOE\_6 | <--- | GEN | .992 | .031 | 32.386 | \*\*\* |  |
| EOE\_7 | <--- | GEN | 1.032 | .031 | 33.743 | \*\*\* |  |
| EOE\_8 | <--- | GEN | 1.021 | .031 | 32.783 | \*\*\* |  |
| EOE\_9 | <--- | GEN | 1.001 | .031 | 32.459 | \*\*\* |  |
| EE\_13 | <--- | GEN | .547 | .033 | 16.807 | \*\*\* |  |
| EE\_14 | <--- | GEN | .526 | .032 | 16.285 | \*\*\* |  |
| EE\_15 | <--- | GEN | .541 | .032 | 16.790 | \*\*\* |  |
| EE\_16 | <--- | GEN | .521 | .032 | 16.080 | \*\*\* |  |
| EE\_17 | <--- | GEN | .534 | .032 | 16.651 | \*\*\* |  |

## Standardized Regression Weights: (Group number 1 - Default model)

|  |  |  | Estimate |
| --- | --- | --- | --- |
| EOE\_1 | <--- | GEN | .714 |
| EOE\_2 | <--- | GEN | .722 |
| EOE\_3 | <--- | GEN | .716 |
| EOE\_4 | <--- | GEN | .715 |
| EOE\_5 | <--- | GEN | .731 |
| EOE\_6 | <--- | GEN | .711 |
| EOE\_7 | <--- | GEN | .741 |
| EOE\_8 | <--- | GEN | .720 |
| EOE\_9 | <--- | GEN | .712 |
| EE\_13 | <--- | GEN | .369 |
| EE\_14 | <--- | GEN | .357 |
| EE\_15 | <--- | GEN | .368 |
| EE\_16 | <--- | GEN | .353 |
| EE\_17 | <--- | GEN | .365 |

## Variances: (Group number 1 - Default model)

|  |  |  | Estimate | S.E. | C.R. | P | Label |
| --- | --- | --- | --- | --- | --- | --- | --- |
| GEN |  |  | .270 | .014 | 19.044 | \*\*\* |  |
| e1 |  |  | .260 | .009 | 30.379 | \*\*\* |  |
| e2 |  |  | .255 | .008 | 30.221 | \*\*\* |  |
| e3 |  |  | .258 | .009 | 30.334 | \*\*\* |  |
| e4 |  |  | .253 | .008 | 30.366 | \*\*\* |  |
| e5 |  |  | .250 | .008 | 30.035 | \*\*\* |  |
| e6 |  |  | .260 | .009 | 30.439 | \*\*\* |  |
| e7 |  |  | .236 | .008 | 29.802 | \*\*\* |  |
| e8 |  |  | .262 | .009 | 30.268 | \*\*\* |  |
| e9 |  |  | .263 | .009 | 30.408 | \*\*\* |  |
| e10 |  |  | .513 | .015 | 33.242 | \*\*\* |  |
| e11 |  |  | .512 | .015 | 33.277 | \*\*\* |  |
| e12 |  |  | .504 | .015 | 33.243 | \*\*\* |  |
| e13 |  |  | .517 | .016 | 33.291 | \*\*\* |  |
| e14 |  |  | .500 | .015 | 33.253 | \*\*\* |  |

## Modification Indices (Group number 1 - Default model)

## Covariances: (Group number 1 - Default model)

|  |  |  | M.I. | Par Change |
| --- | --- | --- | --- | --- |
| e13 | <--> | e14 | 571.193 | .258 |
| e12 | <--> | e14 | 559.776 | .252 |
| e12 | <--> | e13 | 541.967 | .252 |
| e11 | <--> | e14 | 525.512 | .246 |
| e11 | <--> | e13 | 508.416 | .246 |
| e11 | <--> | e12 | 525.729 | .247 |
| e10 | <--> | e14 | 503.622 | .241 |
| e10 | <--> | e13 | 541.837 | .254 |
| e10 | <--> | e12 | 563.460 | .256 |
| e10 | <--> | e11 | 584.327 | .263 |
| e9 | <--> | e14 | 9.418 | -.025 |
| e9 | <--> | e13 | 16.040 | -.033 |
| e9 | <--> | e12 | 23.405 | -.039 |
| e9 | <--> | e11 | 13.491 | -.030 |
| e9 | <--> | e10 | 11.714 | -.028 |
| e8 | <--> | e14 | 16.402 | -.032 |
| e8 | <--> | e13 | 25.217 | -.041 |
| e8 | <--> | e12 | 19.594 | -.036 |
| e8 | <--> | e11 | 22.651 | -.039 |
| e8 | <--> | e10 | 21.004 | -.037 |
| e7 | <--> | e14 | 7.681 | -.021 |
| e7 | <--> | e13 | 19.152 | -.034 |
| e7 | <--> | e12 | 11.884 | -.027 |
| e7 | <--> | e11 | 13.902 | -.029 |
| e7 | <--> | e10 | 13.586 | -.029 |
| e7 | <--> | e9 | 6.675 | .015 |
| e6 | <--> | e14 | 24.333 | -.039 |
| e6 | <--> | e13 | 18.377 | -.035 |
| e6 | <--> | e12 | 17.621 | -.034 |
| e6 | <--> | e11 | 21.844 | -.038 |
| e6 | <--> | e10 | 13.158 | -.029 |
| e6 | <--> | e9 | 7.874 | .017 |
| e6 | <--> | e7 | 7.009 | .015 |
| e5 | <--> | e14 | 21.708 | -.037 |
| e5 | <--> | e13 | 12.150 | -.028 |
| e5 | <--> | e12 | 22.999 | -.038 |
| e5 | <--> | e11 | 10.699 | -.026 |
| e5 | <--> | e10 | 29.055 | -.043 |
| e5 | <--> | e9 | 10.087 | .019 |
| e5 | <--> | e8 | 5.243 | .014 |
| e5 | <--> | e6 | 11.594 | .020 |
| e4 | <--> | e14 | 28.451 | -.042 |
| e4 | <--> | e13 | 14.084 | -.030 |
| e4 | <--> | e12 | 16.245 | -.032 |
| e4 | <--> | e11 | 12.049 | -.028 |
| e4 | <--> | e10 | 12.776 | -.028 |
| e4 | <--> | e9 | 4.075 | -.012 |
| e3 | <--> | e14 | 15.424 | -.031 |
| e3 | <--> | e13 | 10.953 | -.027 |
| e3 | <--> | e12 | 9.183 | -.024 |
| e3 | <--> | e11 | 13.920 | -.030 |
| e3 | <--> | e10 | 17.310 | -.034 |
| e3 | <--> | e5 | 4.084 | -.012 |
| e3 | <--> | e4 | 12.056 | .020 |
| e2 | <--> | e14 | 13.541 | -.029 |
| e2 | <--> | e13 | 17.669 | -.034 |
| e2 | <--> | e12 | 9.786 | -.025 |
| e2 | <--> | e11 | 23.994 | -.039 |
| e2 | <--> | e10 | 21.603 | -.037 |
| e2 | <--> | e4 | 15.505 | .023 |
| e2 | <--> | e3 | 9.882 | .019 |
| e1 | <--> | e14 | 14.816 | -.031 |
| e1 | <--> | e13 | 17.284 | -.034 |
| e1 | <--> | e12 | 20.564 | -.036 |
| e1 | <--> | e11 | 17.159 | -.033 |
| e1 | <--> | e10 | 10.431 | -.026 |
| e1 | <--> | e3 | 7.308 | .016 |
| e1 | <--> | e2 | 10.185 | .019 |

## Variances: (Group number 1 - Default model)

|  |  |  | M.I. | Par Change |
| --- | --- | --- | --- | --- |

## Regression Weights: (Group number 1 - Default model)

|  |  |  | M.I. | Par Change |
| --- | --- | --- | --- | --- |
| EE\_17 | <--- | EE\_16 | 494.060 | .431 |
| EE\_17 | <--- | EE\_15 | 477.360 | .427 |
| EE\_17 | <--- | EE\_14 | 452.724 | .414 |
| EE\_17 | <--- | EE\_13 | 429.328 | .401 |
| EE\_17 | <--- | EOE\_9 | 4.226 | -.042 |
| EE\_17 | <--- | EOE\_8 | 7.178 | -.054 |
| EE\_17 | <--- | EOE\_6 | 10.981 | -.068 |
| EE\_17 | <--- | EOE\_5 | 9.122 | -.061 |
| EE\_17 | <--- | EOE\_4 | 12.673 | -.074 |
| EE\_17 | <--- | EOE\_3 | 6.830 | -.054 |
| EE\_17 | <--- | EOE\_2 | 5.878 | -.050 |
| EE\_17 | <--- | EOE\_1 | 6.615 | -.053 |
| EE\_16 | <--- | EE\_17 | 488.475 | .441 |
| EE\_16 | <--- | EE\_15 | 462.166 | .427 |
| EE\_16 | <--- | EE\_14 | 437.989 | .414 |
| EE\_16 | <--- | EE\_13 | 461.898 | .423 |
| EE\_16 | <--- | EOE\_9 | 7.197 | -.056 |
| EE\_16 | <--- | EOE\_8 | 11.035 | -.068 |
| EE\_16 | <--- | EOE\_7 | 7.735 | -.058 |
| EE\_16 | <--- | EOE\_6 | 8.292 | -.060 |
| EE\_16 | <--- | EOE\_5 | 5.105 | -.047 |
| EE\_16 | <--- | EOE\_4 | 6.273 | -.053 |
| EE\_16 | <--- | EOE\_3 | 4.850 | -.046 |
| EE\_16 | <--- | EOE\_2 | 7.669 | -.057 |
| EE\_16 | <--- | EOE\_1 | 7.716 | -.058 |
| EE\_15 | <--- | EE\_17 | 478.720 | .431 |
| EE\_15 | <--- | EE\_16 | 468.782 | .422 |
| EE\_15 | <--- | EE\_14 | 452.913 | .416 |
| EE\_15 | <--- | EE\_13 | 480.340 | .426 |
| EE\_15 | <--- | EOE\_9 | 10.504 | -.066 |
| EE\_15 | <--- | EOE\_8 | 8.576 | -.059 |
| EE\_15 | <--- | EOE\_7 | 4.800 | -.045 |
| EE\_15 | <--- | EOE\_6 | 7.952 | -.058 |
| EE\_15 | <--- | EOE\_5 | 9.664 | -.064 |
| EE\_15 | <--- | EOE\_4 | 7.236 | -.056 |
| EE\_15 | <--- | EOE\_3 | 4.067 | -.041 |
| EE\_15 | <--- | EOE\_2 | 4.248 | -.042 |
| EE\_15 | <--- | EOE\_1 | 9.181 | -.062 |
| EE\_14 | <--- | EE\_17 | 449.412 | .421 |
| EE\_14 | <--- | EE\_16 | 439.756 | .412 |
| EE\_14 | <--- | EE\_15 | 448.322 | .418 |
| EE\_14 | <--- | EE\_13 | 498.123 | .437 |
| EE\_14 | <--- | EOE\_9 | 6.054 | -.051 |
| EE\_14 | <--- | EOE\_8 | 9.912 | -.064 |
| EE\_14 | <--- | EOE\_7 | 5.615 | -.049 |
| EE\_14 | <--- | EOE\_6 | 9.857 | -.065 |
| EE\_14 | <--- | EOE\_5 | 4.495 | -.044 |
| EE\_14 | <--- | EOE\_4 | 5.367 | -.049 |
| EE\_14 | <--- | EOE\_3 | 6.164 | -.051 |
| EE\_14 | <--- | EOE\_2 | 10.414 | -.067 |
| EE\_14 | <--- | EOE\_1 | 7.660 | -.057 |
| EE\_13 | <--- | EE\_17 | 430.697 | .413 |
| EE\_13 | <--- | EE\_16 | 468.670 | .426 |
| EE\_13 | <--- | EE\_15 | 480.504 | .434 |
| EE\_13 | <--- | EE\_14 | 503.395 | .443 |
| EE\_13 | <--- | EOE\_9 | 5.257 | -.047 |
| EE\_13 | <--- | EOE\_8 | 9.192 | -.062 |
| EE\_13 | <--- | EOE\_7 | 5.488 | -.049 |
| EE\_13 | <--- | EOE\_6 | 5.938 | -.051 |
| EE\_13 | <--- | EOE\_5 | 12.209 | -.072 |
| EE\_13 | <--- | EOE\_4 | 5.691 | -.050 |
| EE\_13 | <--- | EOE\_3 | 7.665 | -.057 |
| EE\_13 | <--- | EOE\_2 | 9.377 | -.063 |
| EE\_13 | <--- | EOE\_1 | 4.657 | -.045 |
| EOE\_9 | <--- | EE\_17 | 8.063 | -.042 |
| EOE\_9 | <--- | EE\_16 | 13.888 | -.055 |
| EOE\_9 | <--- | EE\_15 | 19.983 | -.066 |
| EOE\_9 | <--- | EE\_14 | 11.635 | -.050 |
| EOE\_9 | <--- | EE\_13 | 9.998 | -.046 |
| EOE\_9 | <--- | EOE\_5 | 4.278 | .032 |
| EOE\_8 | <--- | EE\_17 | 14.044 | -.056 |
| EOE\_8 | <--- | EE\_16 | 21.836 | -.069 |
| EOE\_8 | <--- | EE\_15 | 16.730 | -.060 |
| EOE\_8 | <--- | EE\_14 | 19.536 | -.065 |
| EOE\_8 | <--- | EE\_13 | 17.928 | -.062 |
| EOE\_7 | <--- | EE\_17 | 6.578 | -.036 |
| EOE\_7 | <--- | EE\_16 | 16.588 | -.057 |
| EOE\_7 | <--- | EE\_15 | 10.149 | -.045 |
| EOE\_7 | <--- | EE\_14 | 11.992 | -.049 |
| EOE\_7 | <--- | EE\_13 | 11.599 | -.048 |
| EOE\_6 | <--- | EE\_17 | 20.834 | -.067 |
| EOE\_6 | <--- | EE\_16 | 15.912 | -.058 |
| EOE\_6 | <--- | EE\_15 | 15.044 | -.057 |
| EOE\_6 | <--- | EE\_14 | 18.839 | -.063 |
| EOE\_6 | <--- | EE\_13 | 11.230 | -.049 |
| EOE\_6 | <--- | EOE\_5 | 4.917 | .034 |
| EOE\_5 | <--- | EE\_17 | 18.589 | -.063 |
| EOE\_5 | <--- | EE\_16 | 10.522 | -.047 |
| EOE\_5 | <--- | EE\_15 | 19.639 | -.064 |
| EOE\_5 | <--- | EE\_14 | 9.228 | -.044 |
| EOE\_5 | <--- | EE\_13 | 24.803 | -.071 |
| EOE\_5 | <--- | EOE\_9 | 4.570 | .032 |
| EOE\_5 | <--- | EOE\_6 | 5.282 | .035 |
| EOE\_4 | <--- | EE\_17 | 24.360 | -.072 |
| EOE\_4 | <--- | EE\_16 | 12.195 | -.050 |
| EOE\_4 | <--- | EE\_15 | 13.870 | -.054 |
| EOE\_4 | <--- | EE\_14 | 10.392 | -.047 |
| EOE\_4 | <--- | EE\_13 | 10.904 | -.047 |
| EOE\_4 | <--- | EOE\_3 | 5.385 | .035 |
| EOE\_4 | <--- | EOE\_2 | 6.791 | .039 |
| EOE\_3 | <--- | EE\_17 | 13.206 | -.053 |
| EOE\_3 | <--- | EE\_16 | 9.484 | -.045 |
| EOE\_3 | <--- | EE\_15 | 7.840 | -.041 |
| EOE\_3 | <--- | EE\_14 | 12.006 | -.051 |
| EOE\_3 | <--- | EE\_13 | 14.774 | -.056 |
| EOE\_3 | <--- | EOE\_4 | 5.417 | .036 |
| EOE\_3 | <--- | EOE\_2 | 4.328 | .032 |
| EOE\_2 | <--- | EE\_17 | 11.595 | -.050 |
| EOE\_2 | <--- | EE\_16 | 15.301 | -.057 |
| EOE\_2 | <--- | EE\_15 | 8.356 | -.042 |
| EOE\_2 | <--- | EE\_14 | 20.695 | -.066 |
| EOE\_2 | <--- | EE\_13 | 18.439 | -.062 |
| EOE\_2 | <--- | EOE\_4 | 6.969 | .041 |
| EOE\_2 | <--- | EOE\_3 | 4.416 | .032 |
| EOE\_2 | <--- | EOE\_1 | 4.588 | .033 |
| EOE\_1 | <--- | EE\_17 | 12.685 | -.053 |
| EOE\_1 | <--- | EE\_16 | 14.967 | -.056 |
| EOE\_1 | <--- | EE\_15 | 17.557 | -.061 |
| EOE\_1 | <--- | EE\_14 | 14.799 | -.056 |
| EOE\_1 | <--- | EE\_13 | 8.903 | -.043 |
| EOE\_1 | <--- | EOE\_2 | 4.461 | .032 |

## Minimization History (Default model)

| Iteration |  | Negative eigenvalues | Condition # | Smallest eigenvalue | Diameter | F | NTries | Ratio |
| --- | --- | --- | --- | --- | --- | --- | --- | --- |
| 0 | e | 3 |  | -1.041 | 9999.000 | 13717.494 | 0 | 9999.000 |
| 1 | e | 1 |  | -.058 | 2.532 | 6142.997 | 19 | .264 |
| 2 | e | 1 |  | -.022 | .912 | 4359.362 | 5 | .781 |
| 3 | e | 0 | 18.596 |  | .580 | 4000.671 | 5 | .885 |
| 4 | e | 0 | 15.335 |  | .326 | 3916.637 | 2 | .000 |
| 5 | e | 0 | 21.012 |  | .246 | 3891.173 | 1 | 1.142 |
| 6 | e | 0 | 24.753 |  | .081 | 3889.461 | 1 | 1.058 |
| 7 | e | 0 | 25.253 |  | .008 | 3889.447 | 1 | 1.006 |
| 8 | e | 0 | 25.174 |  | .000 | 3889.447 | 1 | 1.000 |

## Model Fit Summary

## CMIN

| Model | NPAR | CMIN | DF | P | CMIN/DF |
| --- | --- | --- | --- | --- | --- |
| Default model | 28 | 3889.447 | 77 | .000 | 50.512 |
| Saturated model | 105 | .000 | 0 |  |  |
| Independence model | 14 | 14934.693 | 91 | .000 | 164.118 |

## RMR, GFI

| Model | RMR | GFI | AGFI | PGFI |
| --- | --- | --- | --- | --- |
| Default model | .078 | .724 | .624 | .531 |
| Saturated model | .000 | 1.000 |  |  |
| Independence model | .206 | .318 | .213 | .275 |

## Baseline Comparisons

| Model | NFI Delta1 | RFI rho1 | IFI Delta2 | TLI rho2 | CFI |
| --- | --- | --- | --- | --- | --- |
| Default model | .740 | .692 | .743 | .696 | .743 |
| Saturated model | 1.000 |  | 1.000 |  | 1.000 |
| Independence model | .000 | .000 | .000 | .000 | .000 |

## Parsimony-Adjusted Measures

| Model | PRATIO | PNFI | PCFI |
| --- | --- | --- | --- |
| Default model | .846 | .626 | .629 |
| Saturated model | .000 | .000 | .000 |
| Independence model | 1.000 | .000 | .000 |

## NCP

| Model | NCP | LO 90 | HI 90 |
| --- | --- | --- | --- |
| Default model | 3812.447 | 3611.857 | 4020.308 |
| Saturated model | .000 | .000 | .000 |
| Independence model | 14843.693 | 14445.430 | 15248.244 |

## FMIN

| Model | FMIN | F0 | LO 90 | HI 90 |
| --- | --- | --- | --- | --- |
| Default model | 1.707 | 1.674 | 1.586 | 1.765 |
| Saturated model | .000 | .000 | .000 | .000 |
| Independence model | 6.556 | 6.516 | 6.341 | 6.694 |

## RMSEA

| Model | RMSEA | LO 90 | HI 90 | PCLOSE |
| --- | --- | --- | --- | --- |
| Default model | .147 | .143 | .151 | .000 |
| Independence model | .268 | .264 | .271 | .000 |

## AIC

| Model | AIC | BCC | BIC | CAIC |
| --- | --- | --- | --- | --- |
| Default model | 3945.447 | 3945.818 | 4105.929 | 4133.929 |
| Saturated model | 210.000 | 211.392 | 811.807 | 916.807 |
| Independence model | 14962.693 | 14962.879 | 15042.934 | 15056.934 |

## ECVI

| Model | ECVI | LO 90 | HI 90 | MECVI |
| --- | --- | --- | --- | --- |
| Default model | 1.732 | 1.644 | 1.823 | 1.732 |
| Saturated model | .092 | .092 | .092 | .093 |
| Independence model | 6.568 | 6.394 | 6.746 | 6.568 |

## HOELTER

| Model | HOELTER .05 | HOELTER .01 |
| --- | --- | --- |
| Default model | 58 | 64 |
| Independence model | 18 | 20 |

## Execution time summary

|  |  |
| --- | --- |
| Minimization: | .065 |
| Miscellaneous: | .342 |
| Bootstrap: | .000 |
| Total: | .407 |
